# Therapeutic Efficiency of an External Chinese Herbal Formula of Mammary Precancerous Lesions by BATMAN-TCM Online Bioinformatics Analysis Tool and Experimental Validation

**DOI:** 10.1155/2019/2795010

**Published:** 2019-02-17

**Authors:** Guijuan Zhang, Xuefeng Jiang, Yusheng Liu, Xiaoqian Hao, Yurong Wang, Xianxin Yan, Naijun Yuan, Yi Ma, Min Ma

**Affiliations:** ^1^The First Affiliated Hospital of Jinan University, Guangzhou, Guangdong 510630, China; ^2^College of Traditional Chinese Medicine of Jinan University, Guangzhou, Guangdong 510632, China; ^3^Institute of Biomedicine and Department of Cellular Biology, Jinan University, Guangzhou, Guangdong 510632, China

## Abstract

Ruyan Neixiao Cream (RYNXC), a patented Chinese herbal formula, was reported to have the effect of treating mammary precancerous disease. In this study, we predicted the potential targets, pathways, and diseases of the ingredients contained in each herbal of RYNXC and constructed an ingredients-targets-diseases network. Then, we analyzed molecular mechanisms of this Chinese herbal formula by MCF-10AT cells and model rats of breast precancerous lesions. BATMAN-TCM prediction showed that ESR1, PGR, PTGS2, EGFR, and Src were mRNA targets of RYNXC. Our results suggested that RYNXC transdermal fluid downregulated ESR1, PGR, PTGS2, EGFR, and Src expression at gene and protein level in MCF-10AT cells. In the rat breast precancerous lesions model, high and low dose RYNXC could also significantly reduce genes and proteins expression of ESR1, PGR, PTGS2, EGFR, and Src. Taken together these data indicate that RYNXC targets multiple molecules responsible for breast precancerous lesion and is an effective Chinese herbal formula. So RYNXC may be a promising external drug for breast precancerous lesions.

## 1. Introduction

Breast cancer was the most commonly diagnosed cancer among women in the vast majority of countries worldwide (140/184), representing a quarter of all cancers diagnosed in women. It was also the leading cause of cancer-related deaths among women [1].The American Cancer Society (ACS) estimated that there would be 249,260 new cases (246,660 female; 2,600 male) of breast cancer and 40450 died from the disease in the United States by 2016 [2]. With advances in the multidisciplinary treatment of breast cancer, surgery, radiotherapy, chemotherapy, and endocrine therapy remained the primary treatment methods. However, chemotherapy, endocrine therapy, and other methods must have some toxic side effects [3–5]. For the current research and development of targeted drugs, there were more and more targeted drugs for different signal pathways, but most targeted drugs could only inhibit part of the signaling pathway, while cancer cells had almost a wide range of capabilities to respond to individual molecular-targeted drugs. And the toxicity and expensive features of most targeted drugs, in the clinical feasibility, were not high of the combination of drugs [6].

At the end of the 20th century, researchers proposed a “multistage development model for breast cancer.” The development of breast cancer had experienced the continuous process of “usual ductal hyperplasia (UDH)-atypical ductal epithelial hyperplasia (ADH)-ductal carcinoma in situ (DCIS) - invasive breast cancer (IBC)” [7]. WHO reported that precancerous lesions were diseases in which the risks developing into cancer were more than 20%. It referred to the presence of atypical hyperplasia before the appearance of malignant neoplasms, but it did not yet have malignant characteristic changes or certain lesions that are easily developed into cancer [8, 9]. It was known that breast precancerous lesions mainly include ADH, DCIS, ductal carcinoma, papillary tumor, and resulted in approximately 50% probability of breast cancer occurring [10]. Precancerous lesion, the essential stage of breast cancer progression, could be blocked or reversed via specific drug intervention. Thereby, the incidence of breast cancer could be effectively decreased by blocking the tumor development process.

Traditional Chinese medicine (TCM) had been widely used in china and proved effective in the prevention and treatment of various diseases. Some natural products from TCM were well characterized at the molecular level and had the well-understood mechanism of action. However, the molecular mechanism of action for many TCMs remained unclear due to the complexity of many ingredients and their interactions with biological receptors. This created difficulties in identifying the essential active ingredients and quality control. TCM theory inspired Tu Yo Yo to discover artemisinin, an antimalarial drug, who won the Nobel Prize in physiology or medicine in 2015. The Dantonic (T89) capsule had demonstrated positive results in phase III clinical trial to prevent and treat stable angina and had a great chance of being approved by the US Food and Drug Administration (FDA). Ban Lan Gen, a well-known Chinese cold and flu remedy, had been approved by UK Medicines and Healthcare Products Regulatory Agency (MHRA)[11]. Along with the increasing number of successful developments and modernization of TCM products, TCM had gained more attention both from academia and industries.

Ruyan Neixiao Cream (RYNXC) were modified from the well-known traditional Chinese prescription “Yindu Neixiao Pulvis”. And RYNXC had been used for a long time in the external treatment of surgical diseases, including breast cancer and precancerous lesion. The preparation process and parameters of RYNXC had obtained national invention patent (ZL201110029344.1)[12]. RYNXC consisted of 9 traditional Chinese herbs, including clove, rhubarb, frankincense, myrrh, borneol, rhizoma corydalis, cowherb seed, Rosae rugosae, Garden balsam stem. Above with the use of herbs could invigorate the circulation of qi and dissipate phlegm and resolve mass. At present, modern pharmacological research had shown that Activating blood, removing phlegm and dredging the liver to use qi medicine had their unique antitumor mechanism[13, 14]. Therefore, pharmacological actions and drug targets of RYNXC were required so as to provide experimental data for developing new drugs.

In recent years, more and more evidence indicated that combining target prediction of herbal ingredients and subsequent network pharmacology analyses was a feasible and powerful way to analyze the molecular mechanism of TCM. The curative effect of compound prescription of traditional Chinese medicine depends on the result of the interaction between various pharmacodynamic substances and macromolecules of the body. BATMAN-TCM (http://bionet.ncpsb.org/batman-tcm/) is an online bioinformatics analysis tool, and the core idea of this method is ranking potential drug-target interactions based on their similarity to the known drug-target interactions. It also supports users to simultaneously input multiple TCMs, which is typically used to simultaneously analyze multiple compositive herbs of a formula, helping understand this combinational principle of a formula from molecular and systematic level, which will help to reveal the occurrence and development of diseases from the systems biology and biological network balance, and understand the interactions between drugs and the body from the holistic perspective of improving and restoring biological balance. In the present study, we applied batman-TCM Online database to predict the potential targets, pathways, and diseases of the ingredients contained in each herbal of RYNXC, which were collected from several databases. Then we constructed an ingredients-targets-diseases network to predict its efficiency on breast cancer. Finally, combined with subsequent cytology and animal experiments validation preliminarily elucidated the mechanism of action of RYNXC.

## 2. Materials and Methods

### 2.1. Target Prediction

Analysis of network pharmacology based on Bioinformatics Analysis Tool for Molecular mechanism of TCM (BATMAN-TCM, http://bionet.ncpsb.org/batman-tcm/). RYNXC was composed of 9 herbs including Syzygium aromaticum (Dingxiang), Rheum officinale (Dahuang), Olibanum (Ruxiang), myrrh (Moyao), Borneol (Bingpian), Rhizoma corydalis (Yanhusuo), Semen vaccariae (Wangbuliuxing), Flos rosae rugosae (Meiguihua), Garden balsam stem (Tougucao), which respectively act as “emperor”, “minister”, “adjuvant” and “courier” in the formula. 9 herbs of RYNXC were submitted to BATMAN-TCM. The predicted candidate targets (including known targets) with scores not smaller than Score cutoff = 20 for each ingredient were presented. The known and predicted targets were obtained by the retrieval in the integrated known drug-target interaction dataset and the prediction respectively and used for further bioinformatics analyses (the data source: DrugBank (https://www.drugbank.ca/), Kyoto Encyclopedia of Genes and Genomes (KEGG, https://www.genome.jp/kegg/pathway.html) and Therapeutic Target Database (TTD, http//en.wikipedia.org/wiki/therapeutic-targets-database)). Significantly enriched KEGG pathways/GO terms/TTD diseases with adjusted P-value smaller than adjusted P-value cutoff = 0.05 were highlighted in the results (The data source: Gene Ontology (GO, http://geneontology.org/), KEGG and TTD). Then we constructed an ingredients-targets-diseases network to predict its efficiency on breast cancer.

### 2.2. Experimental Validation

#### 2.2.1. Reagents and Antibodies

All herbs were provided by the First Affiliated Hospital of Jinan University (China). Blank matrix and Ruyan Neixiao Cream (Per 1g of high or low dose RYNXC, respectively, contained 4g or 2g herbal drugs) was prepared in School of traditional Chinese medicine, Jinan University. Dulbecco's modified Eagle medium (DMEM), Nutrient Mixture F-12 (DMEM/F12) media, horse serum, trypsin and penicillin/streptomycin were purchased from Life Technologies, Gibco BRL Products (Rockville, MD, USA). 7,12-Dimethylbenz[a]anthracene (DMBA) was purchased from Tokyo Chemical Industry Co., Ltd (Tokyo, Japan). Estradiol Benzoate injection was from Shanghai GM Pharmaceutical Co., Ltd (China), and progesterone injection was from Zhejiang Xianju Pharmaceutical Co., Ltd (China). Tamoxifen was purchased from Wuhan Chi-Fei Chemical Co., Ltd. (China). ER-*α*, PR, EGFR, COX2, Src and *β*-actin were all obtained from Cell Signaling Technology (Beverly, MA, USA). RNAiso plus, prime script™ RT reagent Kit with gDNA Eraser, and SYBR Premix EX Taq™ were obtained from Takara (Tokyo, Japan).

#### 2.2.2. Medicine Disposition

DMBA solution: The DMBA was precisely weighed, and completely dissolved in sesame oil by 7 mg/ml ratio.

Tamoxifen cream: 99.5 g blank matrix was heated to melting, 0.5 g Tam was slowly added under constant stirring and preserved at room temperature.

Ruyan Neixiao transdermal fluid (RYNXT): The permeation experiments were performed using Nude mouse skin human skin on modified Franz Diffusion Cell System for In Vitro. RYNXC was weighed 2g, added to the supply room. Normal saline was added accept the room, placed in a thermostatic water bath (37°C, 24h). Finally, RYNXT was filtered by 0.22 *μ*m sterile filter and stored at -20°C.

Blank matrix transdermal fluid: 2g blank matrix was added to the supply room. The other steps were the same as above.

Tamoxifen solution: Tamoxifen in powder form was dissolved in normal saline at a concentration of 10^−3^mol/L and stored at -20°C. It was used concentration of 200 *μ*M.

#### 2.2.3. Cell Lines

MCF10A, MCF-7, and MDA-MB-231 cell lines were purchased from the American Type Culture Collection (ATCC) and cultured according to manufacturer's directions. MCF10AT cell lines were obtained from American Karmanos cancer research institute (KCI). The MCF-10AT cell line were cultured in DMEM/F12 (1:1) supplemented with 5% horse serum, 10 *μ*g/ml insulin, 50 *μ*g/ml hydrocortisone, 20 ng/ml recombinant epidermal growth factor, 100 units/ml penicillin, and 0.1 mg/mL streptomycin, at 37°C, in a humidified 5% CO2 atmosphere. And cells at logarithmic growth phase were used in the cytological experiments.

#### 2.2.4. Cell Viability Assay

The cell viability was measured using a 3-(4,5-Dimethylthiazol-2-yl)-2,5-diphenyltetrazolium bromide (MTT) assay. Cells (5×10^3^cells/well) were seeded onto a 96-well plates overnight. Then the medium was replaced by fresh medium containing various concentrations of RYNCT. After a 24h incubation, 20 *μ*l MTT(5 mg/mL) was added in each well and incubated for 4h away from light. The medium was removed and the formazan blue was dissolved in 100 *μ*L of DMSO. Cell proliferation was measured 570nm using a microplate reader. The results are expressed as a ratio compared to control.

#### 2.2.5. Animal Groups and Treatment

60 Healthy female SD rats (SPF, Certified No. SCXK (Guangdong)2013-0002) were kept in Laboratory Animal Center SPF grade animal room in Jinan University (indoor temperature 25°C, natural lighting, free diet). All animals were allowed to acclimatize for a week on normal diet before grouping. The rats, in accordance with the random number table, were divided into normal blank control group, disease model group, blank matrix group, TAM-treated group, high dose RYNXC-treated group, and low dose RYNXC-treated group. The breast precancerous lesion rat model was induced using DMBA in combination with estrogen and progesterone. 1ml/100g DMBA sesame oil were required for one-off gavage experiment in rats. The next 5 days acted as a cycle, and 0.5 mg/kg benzoate estradiol was injected in the medial muscles of hind legs from the 1 day to the 3 day. Then 4 mg/kg progesterone was injected on the 4 day. The rats were observed on the 5 day. Continuous 12 cycles were performed. From the first day for injection, Rat's breasts in each treatment group were covered with 0.2g The drug cream And massaged for a minute every day. The rats were sacrificed and assayed in the 14th week.

#### 2.2.6. Quantitative Reverse Transcription PCR

The mRNA expression of ESR1, PGR, PTGS2, EGFR, and Src were measured by real-time fluorescence quantitative PCR (qPCR). Total RNA was extracted from cells and tissues by Trizol (Takara, Japan). Then, the total RNA concentration and purity were measured using Micronucleic acid spectrophotometer. After quality control, reverse transcription was performed according to the instructions of the Prime Script RT reagent kits (Takara, Japan), and the obtained cDNA was subjected to PCR amplification using the primers detailed in Table 1. The PCR conditions were as follows: predenaturation at 95°C for 1 min, denaturation at 95°C for 5 s, annealing and extension at 60°C for 30 s, and after 40 cycles. The relative quantification results were performed using the formula 2^−ΔΔCt^.

#### 2.2.7. Western Blot Analysis

Total protein was extracted according to the protein extraction kit instructions. Protein concentration was determined using the BCA assay(Beyotime, China).After electrophoresis, the protein was transferred to PVDF membranes (Millipore, Germany), and blocked with 5% skimmed milk powder for 2h at room temperature. The membranes were incubated with primary antibody (diluted according to the manufacturer's instructions) overnight at 4°C. The next day, the membranes were incubated with 1:5000 dilution of horseradish peroxidase-conjugated secondary antibody for 1h at room temperature. Afer extensive washes with TBST, the PVDF membranes were detected by the gel automatic imaging system for exposure. Western blotting was performed using Image-Pro Plus software for grey value analysis.

#### 2.2.8. Hematoxylin-Eosin (HE) Staining

The breast tissues were fixed in 10% buffered formalin and embedded in paraffin. Then, the paraffin samples were serially sectioned (4 *μ*m), conventionally baked, dewaxed, hydrated, and stained. Finally, the histological changes were observed under light microscopes.

All animal experiments were carried out according to the approved guidelines specified from the Laboratory Animal Ethics Committee of Jinan University. Animal welfare and experimental procedures were approved by the Guide for the Care and Use of Laboratory Animals and related ethical regulations of Jinan University.

#### 2.2.9. Statistical Analysis

Statistical analysis of the experimental data was performed using SPSS 17.0 software. The experimental data were expressed as mean ± SD and differences between groups were analyzed using the One-Way ANOVA Test.* P*< 0.05 was considered statistically significant.

## 3. Results

### 3.1. To Predict the Potential Action Target of Ingredient Pharmaceutical Compound for RYNXC

RYNXC included 9 herbs, such as Syzygium aromaticum (Dingxiang), Rheum officinale (Dahuang), Olibanum (Ruxiang), myrrh (Moyao), Borneol (Bingpian), Rhizoma corydalis (Yanhusuo), Semen vaccariae (Wangbuliuxing), Flos rosae rugosae (Meiguihua), Garden balsam stem (Tougucao), which contained 382 compounds (among which 167 compounds did not have structural information and thus their targets could not be predicted) through searching the BATMAN-TCM. A total of 1626 potential targets were predicted from 215 compounds (Supplementary Table S1).

**Figure 1:**
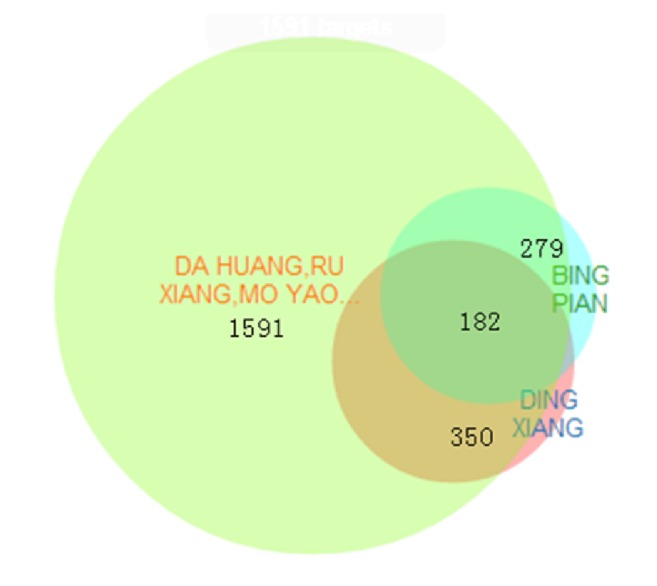
Venn diagram of overlapping potential targets of the nine herbs of RYNXC predicted by BATMAN-TCM.

Clove was the principle drug and 7 herbs were the minister drug covering rhubarb, frankincense, myrrh, rhizoma corydalis, cowherb seed, Rosae rugosae, Garden balsam stem, while borneol was the assistant drug and the envoy drug of this decoction. Clove had 350 potential targets, the minister drug had 1591 potential targets, borneol 279 potential targets. All targets were intersected to obtain 182 main active sites (Figure 1, Supplementary Table S2).

### 3.2. Biological Pathway Enrichment Analysis

1626 candidate potential targets in RYNXC were predicted bioinformatically. In order to further characterize the potential functional pathways altered by RYNXC, we performed GO and KEGG enrichment analysis for targets. Potential targets were mainly enriched in the cell-cell signaling [15], response to stress [16], small molecule metabolic process[17], cell proliferation, and biosynthetic process. We found that biological pathway enrichment analysis revealed many RYNXC's potential target pathways, many of which were known to play important roles in mammary gland-related diseases. For example, The endocrine system plays important roles in the development and progress of breast disease, and endocrine therapy has been widely used in the mastopathy clinical therapeutics [18–20]; deregulation of calcium homeostasis and signaling is associated with mammary gland pathophysiology [21, 22]; cross-talk among tyrosine kinase signaling pathways regulates breast cancer proliferation, protection from cell death, and metastasis [23, 24] (Supplementary Table S3).

### 3.3. Disease Enrichment Analysis

**Figure 2:**
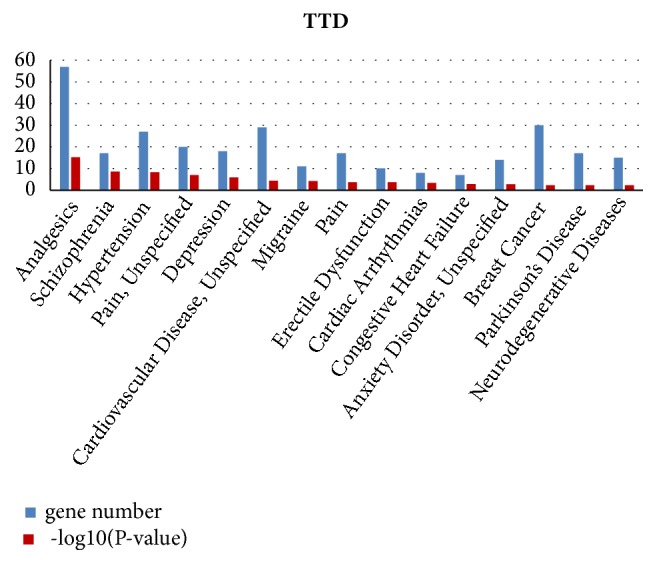
Disease enrichment analysis of potential targets of RYNXC (TOP 15).

**Figure 3:**
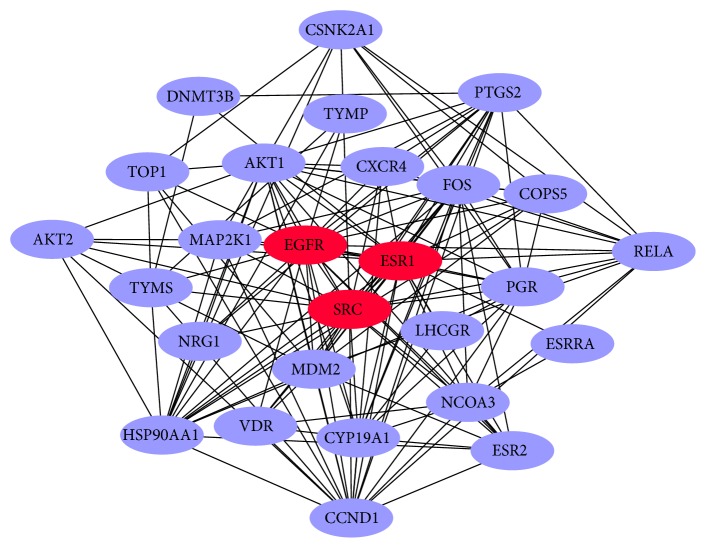
The gene interaction network of potential targets for breast cancer. Each node represents a target, and an edge between two nodes represents their relationship. The key targets are indicated as red ellipses.

Disease enrichment analysis result based on disease-gene associations from Therapeutic Target Database (TTD). Disease analysis indicated that RYNXC had the potential therapeutic effects of Pain, Depression, Cardiovascular Disease, Breast Cancer, Neurodegenerative Diseases and so on (Figure 2, Supplementary Table S3). In the course of clinical use, RYNXC had a great effect to the UDH, ADH, DCIS and other breast diseases. Therefore, 30 targets associated with breast cancer by searching the Therapeutic Target Database. And the gene interaction network of potential targets for breast cancer was constructed by using STRING online software. In the gene interaction network, each node represents a target, and an edge between two nodes represents their relationship. In the end, it contained 27 nodes (AKT1, AKT2, CCND1, COPS5, CSNK2A1, CXCR4, CYP19A1, DNMT3B, EGFR, ESR1, ESR2, ESRRA, FOS,HSP90AA1, LHCGR, MAP2K1, MDM2, NCOA3, NRG1, PGR, PTGS2, RELA, SRC, TOP1, TYMP, TYMS, and VDR) and 147 edges in the gene interaction network, and three targets (ER1, EGFR, and SRC) were the strongest effector for the relationship. we would mark them as red nodes (Figure 3).

### 3.4. The “Ingredient-Target-Pathway/Disease” Association Network

**Figure 4:**
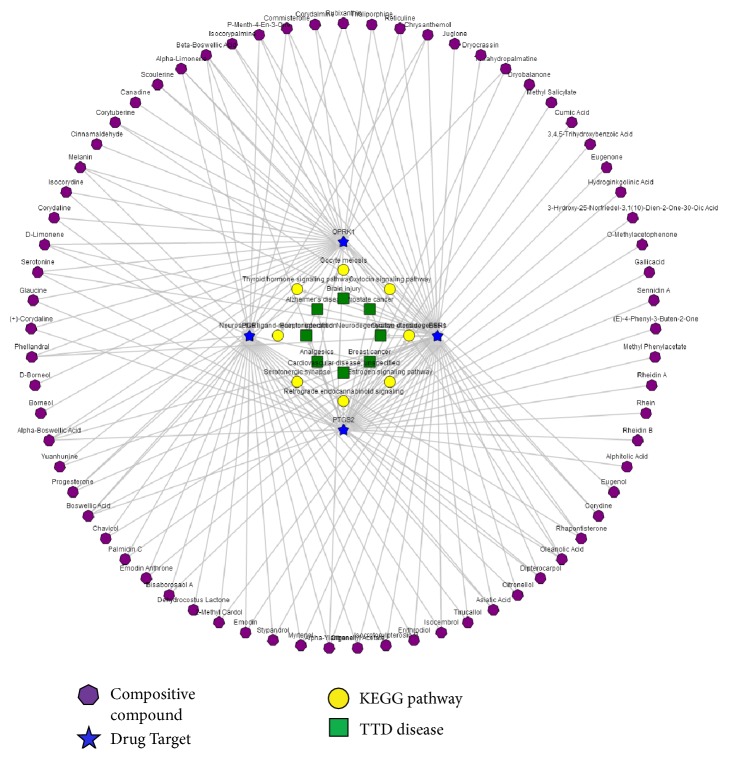
The ingredient-target-pathway/disease association network.

In the view of ingredient-target-pathway/disease association network, there are four kinds of nodes distinguished by different shapes and colors including TCM's ingredients, targets, biological pathways and OMIM/TTD diseases and three types of edges including ingredient-target association, target-pathway association, and target-disease association. Based on the comprehensive analysis of BATMAN-TCM's results, we found that biological pathway enrichment analyses revealed many RYNXC's potential target pathways, many of which were known to play important roles in breast cancer physiology and pathology. The results of network analysis show that RYNXC treat breast cancer by regulating the ESR1, PGR and PTGS2 (Figure 4). Here for the predicted the mechanism of RYNXC for breast cancer, we performed further experimental validation.

### 3.5. RYNXC Might Significantly Inhibit MCF-10AT Cells Proliferation

**Figure 5:**
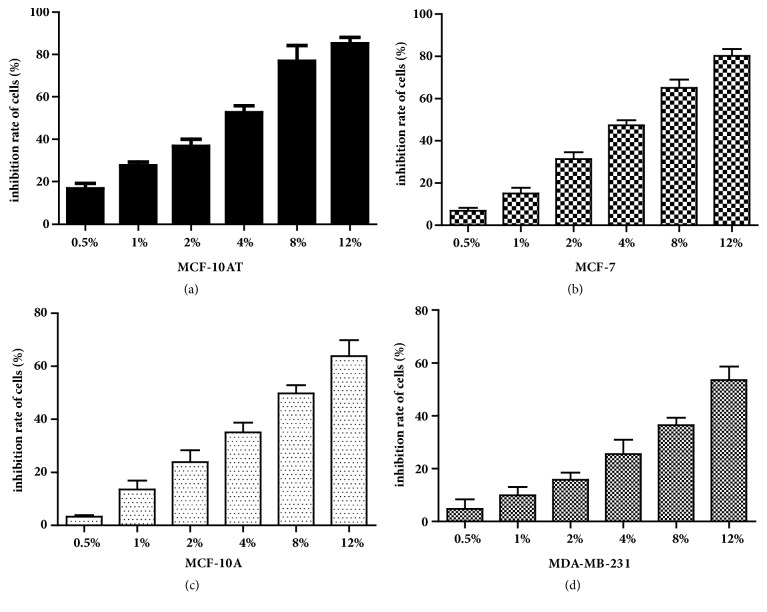
Effects of RYNXT on proliferation of MCF-10A, MCF-10AT, MCF-7, and MDA-MB-231 cells. Values represent the means ± SD. Data are representative of 3 independent experiments.

The MCF-10A, MCF-10AT, MCF-7, and MDA-MB-231 cells in logarithmic growth phase were treated by different concentrations of RYNXT (0.5%, 1%, 2%, 4%, 8% and 12%) for 24h, and the cell proliferation was determined with the MTT assay. RYNXT significantly inhibited MCF10AT cell growth in a dose-dependent manner, the 50% inhibitory concentration (IC50) of RYNXT on MCF-10AT and MCF-7 cells were about 2.96% and 4.23%. However, the IC50 of RYNXT on MCF-10A and MDA-MB-231 cells were 7.31% and 11.90%, respectively (Figure 5). These results showed that RYNXT might inhibit MCF-10AT and MCF-7 cells, but it had a more significant inhibitory effect on MCF-10AT cells. However, it had weak inhibitory effect on MCF-10A and MDA-MB-231 cells.

### 3.6. Effect of RYNXT on the mRNA Expressions of ESR1, PGR, EGFR, PTGS2, and Src mRNA in MCF-10AT Cells

**Figure 6:**
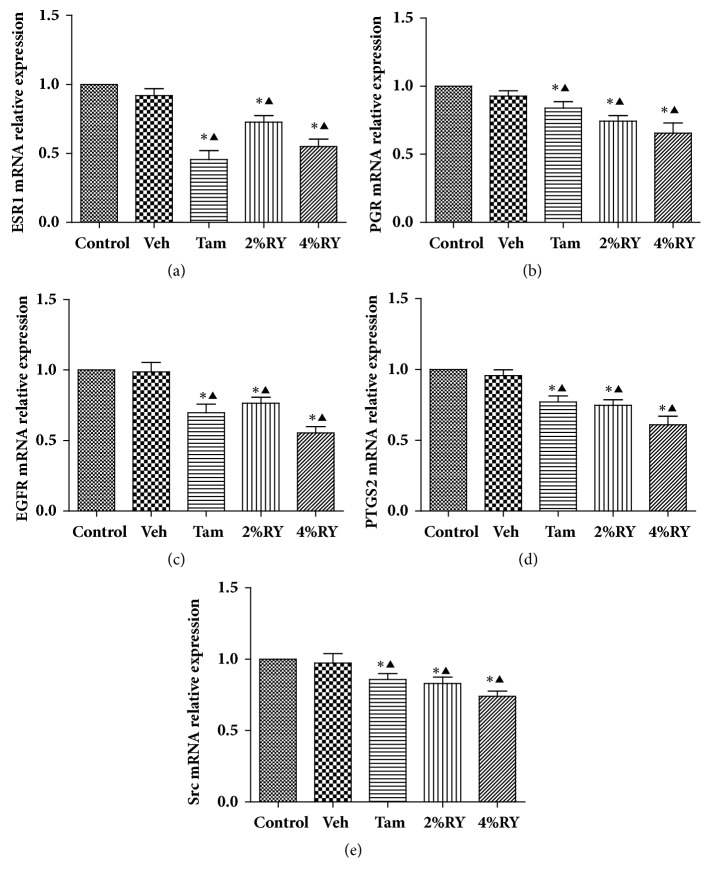
Relative mRNA expression of ESR1(a), PGR(b), EGFR(c), PTGS2(d), and Src(e) at 24h after vehicle, TAM, or various concentrations of RYNXT treatment in MCF-10AT cells. *∗P*<0.05, RYNXT (2%, 4%), TAM, or Veh group versus control group; ^▲^*P*<0.05, RYNXT (2%, 4%), TAM, or control group versus Veh group. Data are representative of 3 independent experiments.

The effect of RYNXT on ESR1, PGR, EGFR, PTGS2, and Src mRNA expressions in MCF-10AT cells was examined by RT-PCR. As shown in Figure 6, ESR1, PGR, EGFR, PTGS2, and Src mRNA expression was no difference between the control group and vehicle control group(*P*> 0.05). Compared with the vehicle control group, the mRNA expression of ESR1, PGR, EGFR, PTGS2 and Src in MCF-10AT cells were decreased in the TAM-, 2% RYNXT – and 4% RYNXT -treated group (*P*<0.05). In contrast, the expression of ESR1, PGR, EGFR, PTGS2 and Src mRNA were lowest in 4% RYNXT -treated group.

### 3.7. Effect of RYNXT on the Protein Expressions of ER-*α*, PR, EGFR, COX2, and Src in MCF-10AT Cells

**Figure 7:**
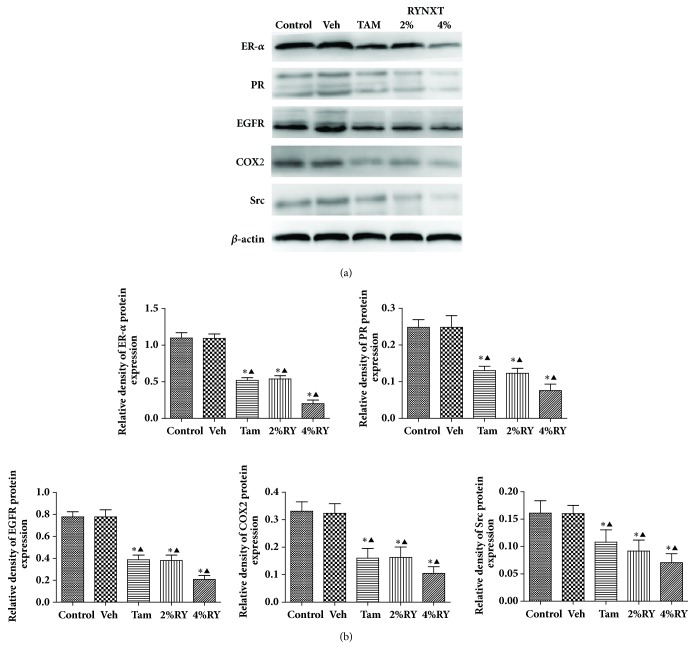
RYNXT significantly inhibited the protein expression of ER-*α*, PR, EGFR, COX2, and Src in MCF-10AT cells. (a) Western-blot assays of key protein expression at 24 h after vehicle, TAM, or various concentrations of RYNXT treatment in MCF-10AT cells. (b) Relative protein expression of ER-*α*, PR, EGFR, COX2, and Src in (a). *∗P*<0.05, RYNXT (2%; 4%), TAM, or Veh versus group control group; ^▲^*P*<0.05, RYNXT (2%; 4%), TAM, or control group versus Veh group. Data are representative of 3 independent experiments.

MCF-10AT that were treated with RYNXT for 24h had reduced expression of ER-*α*, PR, EGFR, COX2, and Src at the protein level.  Figure 7 shows that ER-*α*, PR, EGFR, COX2, and Src protein expression almost showed no difference between the control group and vehicle control group(*P*> 0.05). Compared with control group or vehicle control group, protein expression of ER-*α*, PR, EGFR, COX2, and Src was significantly decreased in TAM-treated group and different doses of RYNXT-treated groups(*P*<0.05), and RYNXT had a dose-dependent effect.

### 3.8. Morphological Observation of Breast Tissue in Rats

**Figure 8:**
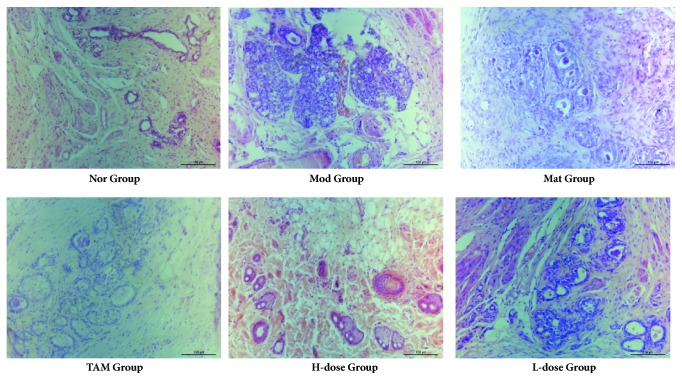
Morphological observation in rats breast tissue of Nor, Mod, Mat, TAM, high dose and low dose RYNXC group (stain ×200).

According to pathological morphological characteristics, the precancerous lesions and invasive carcinoma did not occur in the normal blank control group rats breast tissue. But disease model group, blank matrix group, TAM-, high dose RYNXC and low dose RYNXC-treated group found varying degrees of precancerous lesions and invasive carcinoma (Figure 8). Compared with normal control group, the disease model group and blank matrix group showed typical precancerous lesion characteristics (*P*<0.05). Compared with disease model group, the incidence of precancerous lesions and invasive carcinomas were decreased in the TAM-, high dose RYNXC and low dose RYNXC-treated group (*P*<0.05), and the occurrence rates of breast precancer were respectively decreased by 35.7%, 53.0%, 23.4% (Table 2 and Figure 8). The histological diagnostic results suggest that (1) DMBA combined with estrogen and progesterone could successfully replicate breast precancerous lesions rat model, (2) TAM and RYNXC could alleviate the pathological changes of breast tissue in rats with breast precancerous lesions induced by DMBA with estrogen and progesterone for sequential 5 days. To some extent, the occurrence and development of breast precancerous lesions may be blocked or reversed by RYNXC and TAM.

### 3.9. Effects of RYNXC on mRNA Expression of ESR1, PGR, EGFR, PTGS2, and Src mRNA in Breast Precancerous Lesion Model Rats

**Figure 9:**
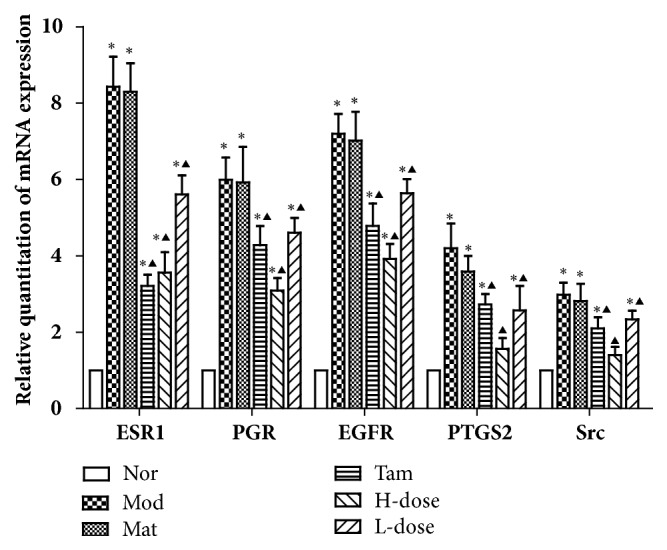
Relative mRNA expression of ESR1, PGR, EGFR, PTGS2 and Src in normal rats or breast precancerous lesion model rats without treatment or continuously treated with blank matrix, TAM, high dose RYNXC, and low dose RYNXC for 14 weeks. *∗P*<0.05, disease model, blank matrix, TAM, or RYNXC (high or low dose) rats versus normal control rats; ^▲^*P*<0.05, blank matrix, TAM, or RYNXC (high or low dose) rats versus disease model rats (n=10 in each group).

In order to detect the therapeutic intervention effect of RYNXC on the progression of precancerous breast cancer, SD rats were smeared with different concentrations of RYNXC every day for 14 weeks. The results of RT-PCR showed that compared with the normal blank control group, the mRNA expression of ESR1, PGR, EGFR, PTGS2 and Src were significantly increased in the disease model group and blank matrix group (*P*<0.05). Compared with the disease model group, the mRNA expression of ESR1, PGR, EGFR, PTGS2, and Src were decreased in the TAM- and RYNXC-treated (high or low dose) group (*P*<0.05) (Figure 9).

### 3.10. Effects of RYNXC on Protein Expression of ER-*α*, PR, EGFR, COX2, and Src in Breast Precancerous Lesion Model Rats

**Figure 10:**
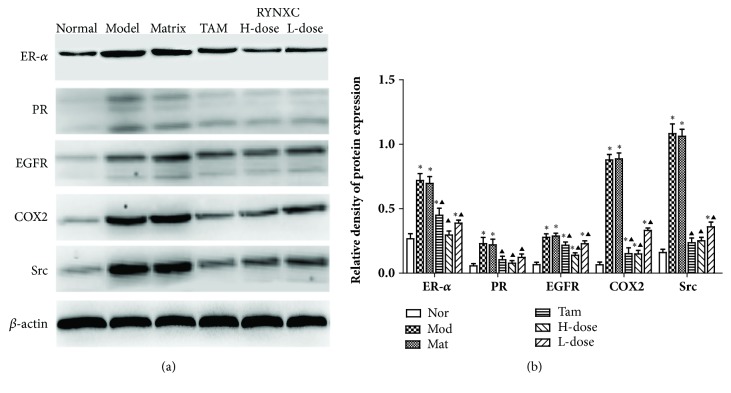
Effects of RYNXC on protein expression of ER-*α*, PR, EGFR, COX2, and Src in breast precancerous lesion model rats. (a) Western-blot assays of key protein expression in normal rats or breast precancerous lesion model rats without treatment or continuously treated with blank matrix, TAM, high dose RYNXC, and low dose RYNXC for 14 weeks.(b) Relative protein expression of ER-*α*, PR, EGFR, COX2, and Src in (a). *∗P*<0.05, disease model, blank matrix, TAM, or RYNXC (high or low dose) rats versus normal control rats; ^▲^*P*<0.05, normal, blank matrix, TAM, or RYNXC (high or low dose) rats versus disease model rats (n=10 in each group).

The effect of RYNXT on expression of ER-*α*, PR, EGFR, COX2, and Src proteins were detected using western blot analysis. The experiment results showed that, compared with the normal control group, the protein expression of ER-*α*, PR, EGFR, COX2, and Src was upregulated in the disease model group and blank matrix group (*P*<0.05). Compared with the disease model group, the protein expression of ER-*α*, PR, EGFR, COX2, and Src in the TAM-, high dose RYNXC-, and low dose RYNXC-treated group was significantly decreased (*P*<0.05) (Figure 10). High dose RYNXC-treated group had a similar efficacy rate with TAM-treated group on the expressions of these key proteins (*P*> 0.05).

## 4. Discussion

Chinese materia medica (CMM) is the typical “multicomponent, multitarget, and multipathway” agent, which coincided with the theory of network pharmacology. Therefore, applying network pharmacology to CMM researches will be helpful to explain the effects of CMM in the treatment of complex diseases holistically and systematically. Bioinformatics Analysis Tool for Molecular mechANism of TCM (BATMAN-TCM, http://bionet.ncpsb.org/batman-tcm/) was the first online bioinformatics analysis tool specially designed for the research of molecular mechanism of TCM, mainly based on TCM ingredients' target prediction and the following network pharmacology analyses of the potential targets, aiming to contribute to the understanding of the “multicomponent, multitargets, and multipathway” combinational therapeutic mechanism of TCM and to provide clues for the following experimental validation [25, 26]. Therefore, this study used BATMAN-TCM online analysis tool to predict the therapeutic efficiency of RYNXC on prebreast cancer and primarily testified by experiment.

The study of substance basis and functional mechanism of Chinese Material Medica is the research focus of current CMM study. Our previous research has shown that herbs in RYNXC contain multiple bioactive constituents. The fifteen compounds from the ingredient herbs of RYNXC were characterized according to HPLC-MS data, including gallic acid, cianidanol, chlorogenic acid, tetrahydropalmatine, rosmarinic acid, quercetin, luteolin, eugenol, kaempferol, apigenin, aloe-emodin, rhein, emodin, 11-keto-*β*-boswellic acid, and 3-acetyl-11-keto-*β*-boswellic acid. The compounds can be classified as organic acids, tannin, alkaloid, volatile oil, anthraquinones, and flavonoids. Moreover, we also identified these compounds in RYNXT [12]. Research at home and abroad indicated that the active ingredient pharmaceutical compounds for RYNXC had the effect of treating mammary gland-related diseases. Eugenol inhibited oxidative phosphorylation and fatty acid oxidation by downregulation of c-Myc/PGC-1*β*/ERR*α* signaling pathway in MCF10AT cells [27] and could alleviate breast precancerous lesions through HER2/PI3K-AKT pathway-induced cell apoptosis and S-phase arrest [28]. Emodin and aloe-emodin could suppress breast cancer cell proliferation through ER*α* inhibition aside from its anti-inflammatory activity [29, 30]. Boswellic acid as a potent anticancer agent acted against multiple intracellular targets that affect angiogenesis (VEGF), inflammation (TNF-*α*, IL-12), apoptosis (caspase-3 and caspase-9), and antioxidant (SOD and CAT) based anticarcinogenic mechanisms. It inhibited MCF-7 cells proliferation and potentiated the cell death induced by VEGF antibody [31]. Myrrh could kill breast cancer cells without harming healthy cells by inactivating a protein called Bcl-2 [32, 33]. Borneol could induce cancer cells apoptosis, such as breast cancer, lung squamous cell carcinoma, lung epithelial cancer, and rhinitis cancer [34]. However, there have been no relevant reports based on the theory of TCM on the treatment of precancerous breast cancer by TCM compound prescription.

Unlike western medicines, most herbal compounds have unknown targets. The significantly enriched KEGG biological pathways, Gene Ontology (GO) functional terms (including biological process, molecular function, and cellular component) and OMIM/TTD disease phenotypes may play crucial roles for TCM's therapeutic effects, providing direct clues for further experimental validation of molecular mechanism of TCM. RYNXC included 9 traditional Chinese herbs, such as clove, rhubarb, olibanum, myrrh, and borneol. For the 382 ingredients (215 compounds have structural information) we obtained 1626 potential targets adopting similarity-based target prediction method. Then enrichment analysis was implemented for them. We found that biological pathway enrichment analyses revealed many RYNXC's potential target pathways. The endocrine system [28], calcium signaling pathway [29], signal transduction [30], and tyrosine metabolism [31] play important roles in the occurrence and progression of breast cancer. Significantly enriched disease phenotypes included analgesics, schizophrenia, depression, cardiovascular disease, breast cancer, and neurodegenerative diseases, many of which are RYNXC's known indications and other suggest RYNXC's potential therapeutic effects. We built gene interaction network for 30 targets associated with breast cancer and observed ER1, EGFR, and SRC were the strongest effector for the relationship. In addition, we constructed an ingredients-targets-diseases network that show that RYNXC treated breast cancer by regulating the ESR1, PGR, and PTGS2. And we found that ESR1, PGR, PTGS2, and Src were common mRNA targets of RYNXC. The results indicated that ESR1, PGR, EGFR, PTGS2, and Src may be the potential therapeutic target of RYNXC for the treatment of breast cancer. We performed further experimental validation.

The MCF10-AT cells derived from Xenotransplantation of MCF10A-ras cells and generated carcinomas in about 25% of xenografts, representing the transition from normal epithelium to malignant cancer [35]. RYNXC could significantly inhibit proliferation of breast precancerous lesion MCF-10AT cells, but there was almost no significant inhibitory effect on MCF-10A or MDA-MB-231 cells. In this study, RYNXC could significantly decrease ESR1, PGR, EGFR, PTGS2, and Src mRNA expressions in a dose-dependent manner in MCF-10AT cells or breast precancerous lesion model rats, which could further inhibit cell proliferation and tumor progression. In the current diagnosis and treatment of breast cancer, the most specific markers are estrogen receptor (ER), progesterone receptor (PR), and human epidermal growth factor receptor 2 (HER2). These receptors communicate prognostic information and serve as individualized therapeutic targets [36]. Studies showed that PR was not merely an ER*α*-induced gene target, but it was also an ER*α*-associated protein that modulates its behaviour [37]. EGFR is one of the members in the 4 epidermal growth factor receptor family members and is a transmembrane cell surface distribution sensor with tyrosine kinase activity [38]. EGFR is a monomer in the nonactive state, once EGFR and its ligand bound, the homologous or heterologous dimers will be formed, and receptor in intracellular region will be phosphorylated and self-start a series of intracellular signaling cascade. Ras, MAPK, Src, STAT3/5, PKC, PI3K, etc. are associated with the above signal transmission protein [39, 40]. EGFR also plays an important role in cell derivatization; the interrelated study has revealed that EGFR offered significance contribution in the cancer development process, such as metastasis of tumor cells, adhesion, apoptosis, and angiogenesis [41, 42]. COX (cyclooxygenase), a prostaglandin H synthetase (PGHS), is composed of two distinct isoenzymes, cyclooxygenase-1 (COX-1) and COX-2. These enzymes form part of the prostaglandin synthetase complex of enzymes, which catalyzes the rate-limiting step in the conversion of arachidonic acid into prostaglandin G2 (PGG2) [43]. In the last decade numerous studies have indicated a link between the pathogenesis of breast cancer and the expression of cyclooxygenases, particularly cyclooxygenase-2 (COX-2) [44]. Elevated expression of COX-2 could promote the growth and resistance of tumor obviously and is a negative prognostic factor for disease free survival and overall survival in patients [45, 46]. Src is the regulatory protein. Its upstream pathway includes EGFR, VEGF, HER2, PDGF, etc. [47]. Under the action of upstream factors, Src will be phosphorylated and affect downstream signaling pathways, such as PI3K/Akt, MAPK/ERK, and FAK signaling. It plays a key role not only in cell differentiation, motility, invasion, proliferation, and cell survival, but also in the advancement and metastasis of solid tumors [48, 49]. So RYNXC may effectively inhibit the progression of breast precancerous lesion through blocking protein expression of ER-*α*, PR, EGFR, COX2, and Src in the endocrine system and tyrosine metabolism.

One of the strengths of our research is that we predicted the therapeutic targets, pathways, and diseases of the ingredients of RYNXC and built the ingredients-targets-diseases network. We have also further identify ESR1, PGR, EGFR, PTGS2, and Src targets using cells and rat models, which may represent a promising therapeutic target in the future. However, the mechanisms of action underlying the beneficial effects of RYNXC on breast precancerous lesions are complex. This study also had some limitations including the fact that RYNXC is an external used cream, unlike the oral medicines. Our previous studies identified 15 compounds in RYNXC and RYNXT. We were not sure what kind of compounds in this herbal formula is more inclined to get through the skin to exert the therapeutic effects. Meanwhile, we only predicted potential targets, and we did not study the signaling pathway and the specific mechanism. Our next study will involve the biological process and signaling pathway against such targets and find out which compounds in this herbal formula are more inclined to get through the skin to exert the therapeutic effects to further explore the mechanism of RYNXC therapy for prebreast cancer.

## 5. Conclusion

By analysis ingredients and potential targets of RYNXC based on network pharmacology, it provided evidence for RYNXC treatment of precancerous lesions. RYNXC could effectively inhibit the progress of precancerous lesions; it might be achieved by inhibiting cell proliferation, downregulating ESR1, PGR, EGFR, PTGS2, and Src mRNA expression levels, and regulating protein expression of ER-*α*, PR, EGFR, COX2, and Src. Thus it may be one of the important mechanisms of RYNXC. The results indicated RYNXC may be a promising external application drug to prevent or treat breast precancerous lesions.

## Figures and Tables

**Table 1:** Sequence of forward and reverse primers used for PCR.

Gene name	Species	Forward primer (5′->3′)	Reverse primer (5′->3′)
ESR1	human	GGAATGCGATGAAGTAGAGCC	ATGAAGTGCAAGAACGTGGTG
PGR	human	TTCATCCGCTGTTCATTTAGT	CTGACACCTCCAGTTCTTTGC
PTGS2	human	ATAAAGCGTTTGCGGTACTCA	GGTTGCTGGTGGTAGGAATGT
EGFR	human	GGGCACGGTAGAAGTTGGAGT	GCTGGATGATAGACGCAGATAGT
Src	human	GGTGCGGGAGGTGATGTAGAA	GCGAGAAAGTGAGACCACGAAA
*β*-actin	human	GGACCTGACCTGCCGTCTAG	GTAGCCCAGGATGCCCTTGA
ESR1	Rat	CTCCTGTTTGCTCCTAACTTGCTCT	CATGCGGAATCGACTTGACGT
PGR	Rat	GCAGCAATAACTTCAGACATCAT	CCAGTTCACAACGCTTCTATC
PTGS2	Rat	TGAAATATCAGGTCATCGGTGGAG	CATACATCATCAGACCCGGCAC
EGFR	Rat	CAGTTTTCTCTGGCGGTTGTCG	TGCAGTCCTTTTCAGCTCTGTTGTT
Src	Rat	TCTGACATCCACCTTCCTCGT	GCTTCAACTCCTCGGACACTG
*β*-actin	Rat	CCGTAAAGACCTCTATGCCAACA	CTAGGAGCCAGGGCAGTAATCTC

**Table 2:** Comparison of the pathological changes of the mammary gland tissue of rats in each treatment group.

Groups	breast number	No hyperplasia	General hyperplasia	Precancerous lesions	Invasive carcinoma
Normal	120	115^▲^	5^▲^	0^▲^	0^▲^
Model	120	0*∗*	13*∗*	98*∗*	9*∗*
Matrix	120	0*∗*	14*∗*	96*∗*	10*∗*
TAM	120	18*∗*^▲^	36*∗*^▲^	63*∗*^▲^	3*∗*^▲^
high dose RYNXC	120	29*∗*^▲^	45*∗*^▲^	46*∗*^▲^	0^▲^
low dose RYNXC	120	11*∗*^▲^	33*∗*^▲^	75*∗*^▲^	1*∗*^▲^

There are 10 rats in every group and each has 6 pairs of nipples. *∗P*<0.05 compared with the normal blank control group; ^▲^*P*<0.05 compared with the disease model group.

## Data Availability

Most of the data (Figures 1–10 and Tables 1 and 2) used to support the findings of this study are included within the article. Some of the data (Supplementary Tables 1–3) used to support the findings of this study are included within the supplementary information file.

## Supplementary Materials

Supplementary Materials Supplementary Tables 1: target prediction result for each ingredient. Supplementary Tables 2: all targets were intersected to obtain 182 main active sites. Supplementary Tables 3: bioinformatics analysis of potential targets.
