# Supplementary material for: Therapeutic Efficiency of an External Chinese Herbal Formula of Mammary Precancerous Lesions by BATMAN-TCM Online Bioinformatics Analysis Tool and Experimental Validation
Source: Evid Based Complement Alternat Med. 2019 Feb 17;2019:2795010. doi: 10.1155/2019/2795010 (PMC6398062; doi:10.1155/2019/2795010)
Supplement: Supplementary Materials — Supplementary Tables 1: target prediction result for each ingredient. Supplementary Tables 2: all targets were intersected to obtain 182 main active sites. Supplementary Tables 3: bioinformatics analysis of potential targets. [file 2795010.f1.zip › Supplementary table. S2.docx]

Supplementary table. S2: All targets were intersected to obtain 182 main active sites.

ACSL4, ACY1, ACY3, ADH1A, ADH1B, ADH1C, ADH4, ADH7, ADORA1, ADRA2A, AKR1C1, AKR1C2, ALDH1A1, ALDH1B1, ALDH2, ALDH3B1, ALDH3B2, ANK3, ANXA1, APOE, AQP8, AREG, ARX, ASPA, ATP1A1, CACNA1D, CAT, CD34, CES1, CFTR, CNTNAP4, COX1, COX2, COX3, COX4I1, COX5A, COX5B, COX6A2, COX6B1, COX6C, COX7A1, COX7B, COX7C, COX8A, CRLF1, CYGB, CYP11A1, CYP17A1, CYP2E1, DAB2IP, DARS, DGKI, DLG4, EDN1, ELOVL4, **ESR1**, ESR2, ESRRG, F12, FABP6, FADD, FADS1, FADS2, FAS, FBP1, FECH, FGFR2, GABRB3, GAMT, GATM, GJA5, GLRA3, GPER1, GPX7, GRIN2A, GUCY1B3, HMGCR, IGF1, IL1B, IYD, KCNA1, KCNA10, KCNA2, KCNA3, KCNA4, KCNA5, KCNA6, KCNA7, KCNB1, KCNB2, KCNC1, KCNC2, KCNC3, KCND1, KCND2, KCND3, KCNE5, KCNIP2, KCNK4, KCNMA1, KCNQ1, LEF1, LEP, LRRC4B, MAGI2, MC4R, MED1, MIP, NEDD4, NFIB, NKX2-1, NKX3-1, NLGN1, NPPA, NR1H4, NR3C1, NR3C2, NRXN1, NRXN2, NRXN3, OPRK1, OXT, OXTR, PAX7, PAXBP1, PDE4B, PDE4D, PDGFB, **PGR**, PHB, PLA2G1B, PPARD, PPARG, PRKAB1, PRKCA, PRLR, PTGER1, PTGER2, PTGER3, PTGER4, PTGS1, **PTGS2**, RAB3A, RAPGEF2, RNASE1, RNASE2, RNASE4, RNASE8, RYR3, SCGB1A1, SCN10A, SCN5A, SERPINB3, SHANK3, SHH, SLC17A7, SLC6A4, SLC8A1, SNTG2, SORCS3, SOX15, SOX9, SPARC, **SRC**, SRD5A1, STX1A, TFAP2C, TGFB1, TNFSF11, TOX3, TPO, TRIM24, TRPA1, TRPM8, TRPV1, TRPV3, TYR, UBR5, VDR, WNT4, WNT5A, ZPR1
