# Supplementary material for: Therapeutic Efficiency of an External Chinese Herbal Formula of Mammary Precancerous Lesions by BATMAN-TCM Online Bioinformatics Analysis Tool and Experimental Validation
Source: Evid Based Complement Alternat Med. 2019 Feb 17;2019:2795010. doi: 10.1155/2019/2795010 (PMC6398062; doi:10.1155/2019/2795010)
Supplement: Supplementary Materials — Supplementary Tables 1: target prediction result for each ingredient. Supplementary Tables 2: all targets were intersected to obtain 182 main active sites. Supplementary Tables 3: bioinformatics analysis of potential targets. [file 2795010.f1.zip › Supplementary table. S1.doc]

Supplementary table. S1: Target Prediction Result for each ingredient.

| **Compound** | **Predicted targets [Gene Symbol] ranked according to the decreasing ([score](http://bionet.ncpsb.org/batman-tcm/index.php/Home/document/index#Algorithm))** |
| --- | --- |
| [Octyl Acetate](http://www.megabionet.org/tcmid/ingredient/24879/) | [NPR1](http://www.ncbi.nlm.nih.gov/gene/?term=4881)(80.882)[AR](http://www.ncbi.nlm.nih.gov/gene/?term=367)(48.000)[NPR3](http://www.ncbi.nlm.nih.gov/gene/?term=4883)(22.373)[NPR2](http://www.ncbi.nlm.nih.gov/gene/?term=4882)(22.373) |
| [Phrymarolin I](http://www.megabionet.org/tcmid/ingredient/17188/) | [TOP2A](http://www.ncbi.nlm.nih.gov/gene/?term=7153)(48.000)[TOP2B](http://www.ncbi.nlm.nih.gov/gene/?term=7155)(48.000) |
| [Juglanin](http://www.megabionet.org/tcmid/ingredient/11894/) | This compound doesn't have any potential target with score larger than 20. |
| [Sennoside C](http://www.megabionet.org/tcmid/ingredient/19736/) | This compound doesn't have any potential target with score larger than 20. |
| [Nopinone](http://www.megabionet.org/tcmid/ingredient/15697/) | [AR](http://www.ncbi.nlm.nih.gov/gene/?term=367)(48.000) |
| [3-Hydroxy-25-Norfriedel-3,1(10)-Dien-2-One-30-Oic Acid](http://www.megabionet.org/tcmid/ingredient/31241/) | [SLC8A1](http://www.ncbi.nlm.nih.gov/gene/?term=6546)(48.000)[NR0B1](http://www.ncbi.nlm.nih.gov/gene/?term=190)(48.000)[TRPV1](http://www.ncbi.nlm.nih.gov/gene/?term=7442)(48.000)[NOS2](http://www.ncbi.nlm.nih.gov/gene/?term=4843)(48.000)[AR](http://www.ncbi.nlm.nih.gov/gene/?term=367)(48.000) [NR3C1](http://www.ncbi.nlm.nih.gov/gene/?term=2908)(48.000)[FADS2](http://www.ncbi.nlm.nih.gov/gene/?term=9415)(48.000)[FADS1](http://www.ncbi.nlm.nih.gov/gene/?term=3992)(48.000)[PTGS1](http://www.ncbi.nlm.nih.gov/gene/?term=5742)(48.000)[PTGS2](http://www.ncbi.nlm.nih.gov/gene/?term=5743)(48.000) [ELOVL4](http://www.ncbi.nlm.nih.gov/gene/?term=6785)(48.000)[HSD11B1](http://www.ncbi.nlm.nih.gov/gene/?term=3290)(48.000)[ANXA1](http://www.ncbi.nlm.nih.gov/gene/?term=301)(48.000)[CYP19A1](http://www.ncbi.nlm.nih.gov/gene/?term=1588)(48.000)[FOLR2](http://www.ncbi.nlm.nih.gov/gene/?term=2350)(23.000) [SHMT1](http://www.ncbi.nlm.nih.gov/gene/?term=6470)(23.000)[GART](http://www.ncbi.nlm.nih.gov/gene/?term=2618)(23.000)[MTHFD2](http://www.ncbi.nlm.nih.gov/gene/?term=10797)(23.000)[MTR](http://www.ncbi.nlm.nih.gov/gene/?term=4548)(23.000)[MTHFD1](http://www.ncbi.nlm.nih.gov/gene/?term=4522)(23.000) [ALDH1L1](http://www.ncbi.nlm.nih.gov/gene/?term=10840)(23.000)[AMT](http://www.ncbi.nlm.nih.gov/gene/?term=275)(23.000)[MTFMT](http://www.ncbi.nlm.nih.gov/gene/?term=123263)(23.000)[ATIC](http://www.ncbi.nlm.nih.gov/gene/?term=471)(23.000)[SHMT2](http://www.ncbi.nlm.nih.gov/gene/?term=6472)(23.000) [DHFR](http://www.ncbi.nlm.nih.gov/gene/?term=1719)(23.000)[FOLR3](http://www.ncbi.nlm.nih.gov/gene/?term=2352)(23.000)[FTCD](http://www.ncbi.nlm.nih.gov/gene/?term=10841)(23.000)[TYMS](http://www.ncbi.nlm.nih.gov/gene/?term=7298)(23.000)[MTHFR](http://www.ncbi.nlm.nih.gov/gene/?term=4524)(23.000) |
| [2,3-Diphenyl-2-Cyclopropen-1-One](http://www.megabionet.org/tcmid/ingredient/6484/) | This compound doesn't have any potential target with score larger than 20. |
| [Palmidin B](http://www.megabionet.org/tcmid/ingredient/16546/) | [CHRM3](http://www.ncbi.nlm.nih.gov/gene/?term=1131)(23.000)[CHRM1](http://www.ncbi.nlm.nih.gov/gene/?term=1128)(23.000)[CHRM2](http://www.ncbi.nlm.nih.gov/gene/?term=1129)(23.000)[CHRM4](http://www.ncbi.nlm.nih.gov/gene/?term=1132)(23.000) |
| [Dipterocarpol](http://www.megabionet.org/tcmid/ingredient/6501/) | [AR](http://www.ncbi.nlm.nih.gov/gene/?term=367)(80.882)[SLC8A1](http://www.ncbi.nlm.nih.gov/gene/?term=6546)(48.000)[F12](http://www.ncbi.nlm.nih.gov/gene/?term=2161)(48.000)[ESR1](http://www.ncbi.nlm.nih.gov/gene/?term=2099)(48.000)[TRPV1](http://www.ncbi.nlm.nih.gov/gene/?term=7442)(48.000) [PTGER1](http://www.ncbi.nlm.nih.gov/gene/?term=5731)(48.000)[PGR](http://www.ncbi.nlm.nih.gov/gene/?term=5241)(48.000)[NR3C1](http://www.ncbi.nlm.nih.gov/gene/?term=2908)(48.000)[FADS2](http://www.ncbi.nlm.nih.gov/gene/?term=9415)(48.000)[FADS1](http://www.ncbi.nlm.nih.gov/gene/?term=3992)(48.000) [PTGER2](http://www.ncbi.nlm.nih.gov/gene/?term=5732)(48.000)[PTGS1](http://www.ncbi.nlm.nih.gov/gene/?term=5742)(48.000)[PTGS2](http://www.ncbi.nlm.nih.gov/gene/?term=5743)(48.000)[ELOVL4](http://www.ncbi.nlm.nih.gov/gene/?term=6785)(48.000)[ANXA1](http://www.ncbi.nlm.nih.gov/gene/?term=301)(48.000) [SRD5A1](http://www.ncbi.nlm.nih.gov/gene/?term=6715)(23.000)[HSD11B1](http://www.ncbi.nlm.nih.gov/gene/?term=3290)(23.000)[COL5A1](http://www.ncbi.nlm.nih.gov/gene/?term=1289)(22.373)[NOX1](http://www.ncbi.nlm.nih.gov/gene/?term=27035)(22.373)[NKX3-1](http://www.ncbi.nlm.nih.gov/gene/?term=4824)(22.373) [SHBG](http://www.ncbi.nlm.nih.gov/gene/?term=6462)(22.373)[TGFB2](http://www.ncbi.nlm.nih.gov/gene/?term=7042)(22.373)[NR1I3](http://www.ncbi.nlm.nih.gov/gene/?term=9970)(22.373)[ALDH1A1](http://www.ncbi.nlm.nih.gov/gene/?term=216)(22.373)[TSPO](http://www.ncbi.nlm.nih.gov/gene/?term=706)(22.373) [S100A9](http://www.ncbi.nlm.nih.gov/gene/?term=6280)(22.373) |
| [Strictosamide](http://www.megabionet.org/tcmid/ingredient/20374/) | This compound doesn't have any potential target with score larger than 20. |
| [Rugosin D](http://www.megabionet.org/tcmid/ingredient/19042/) | This compound doesn't have any potential target with score larger than 20. |
| [Isobrucine](http://www.megabionet.org/tcmid/ingredient/11262/) | This compound doesn't have any potential target with score larger than 20. |
| [Corydamine](http://www.megabionet.org/tcmid/ingredient/4105/) | [CHRNA4](http://www.ncbi.nlm.nih.gov/gene/?term=1137)(23.000)[CHRNA7](http://www.ncbi.nlm.nih.gov/gene/?term=1139)(23.000)[HRH3](http://www.ncbi.nlm.nih.gov/gene/?term=11255)(23.000)[CHRNA3](http://www.ncbi.nlm.nih.gov/gene/?term=1136)(23.000)[HRH1](http://www.ncbi.nlm.nih.gov/gene/?term=3269)(23.000) [CHRNA6](http://www.ncbi.nlm.nih.gov/gene/?term=8973)(23.000)[CHRFAM7A](http://www.ncbi.nlm.nih.gov/gene/?term=89832)(23.000) |
| [Anthraglycoside B](http://www.megabionet.org/tcmid/ingredient/1363/) | This compound doesn't have any potential target with score larger than 20. |
| [Asiatic Acid](http://www.megabionet.org/tcmid/ingredient/24347/) | [SRD5A1](http://www.ncbi.nlm.nih.gov/gene/?term=6715)(48.000)[SLC8A1](http://www.ncbi.nlm.nih.gov/gene/?term=6546)(48.000)[F12](http://www.ncbi.nlm.nih.gov/gene/?term=2161)(48.000)[PRLR](http://www.ncbi.nlm.nih.gov/gene/?term=5618)(48.000)[ESR1](http://www.ncbi.nlm.nih.gov/gene/?term=2099)(48.000) [TRPV1](http://www.ncbi.nlm.nih.gov/gene/?term=7442)(48.000)[PTGER1](http://www.ncbi.nlm.nih.gov/gene/?term=5731)(48.000)[PTGER4](http://www.ncbi.nlm.nih.gov/gene/?term=5734)(48.000)[PGR](http://www.ncbi.nlm.nih.gov/gene/?term=5241)(48.000)[AR](http://www.ncbi.nlm.nih.gov/gene/?term=367)(48.000) [NR3C1](http://www.ncbi.nlm.nih.gov/gene/?term=2908)(48.000)[PTGIR](http://www.ncbi.nlm.nih.gov/gene/?term=5739)(48.000)[FADS2](http://www.ncbi.nlm.nih.gov/gene/?term=9415)(48.000)[FADS1](http://www.ncbi.nlm.nih.gov/gene/?term=3992)(48.000)[PTGER2](http://www.ncbi.nlm.nih.gov/gene/?term=5732)(48.000) [PTGS1](http://www.ncbi.nlm.nih.gov/gene/?term=5742)(48.000)[PTGER3](http://www.ncbi.nlm.nih.gov/gene/?term=5733)(48.000)[PTGS2](http://www.ncbi.nlm.nih.gov/gene/?term=5743)(48.000)[ELOVL4](http://www.ncbi.nlm.nih.gov/gene/?term=6785)(48.000)[PTGFR](http://www.ncbi.nlm.nih.gov/gene/?term=5737)(48.000) [ANXA1](http://www.ncbi.nlm.nih.gov/gene/?term=301)(48.000)[NR3C2](http://www.ncbi.nlm.nih.gov/gene/?term=4306)(48.000) |
| [Dryocrassin](http://www.megabionet.org/tcmid/ingredient/6610/) | [ESR1](http://www.ncbi.nlm.nih.gov/gene/?term=2099)(23.000)[CFTR](http://www.ncbi.nlm.nih.gov/gene/?term=1080)(23.000) |
| [Chrysanthemol](http://www.megabionet.org/tcmid/ingredient/3595/) | [VDR](http://www.ncbi.nlm.nih.gov/gene/?term=7421)(80.882)[GC](http://www.ncbi.nlm.nih.gov/gene/?term=2638)(55.444)[TRPV3](http://www.ncbi.nlm.nih.gov/gene/?term=162514)(48.000)[OPRK1](http://www.ncbi.nlm.nih.gov/gene/?term=4986)(48.000)[TRPM8](http://www.ncbi.nlm.nih.gov/gene/?term=79054)(48.000) [TRPA1](http://www.ncbi.nlm.nih.gov/gene/?term=8989)(48.000)[GPBAR1](http://www.ncbi.nlm.nih.gov/gene/?term=151306)(22.373)[MED1](http://www.ncbi.nlm.nih.gov/gene/?term=5469)(22.373)[CALB1](http://www.ncbi.nlm.nih.gov/gene/?term=793)(22.373)[ESR1](http://www.ncbi.nlm.nih.gov/gene/?term=2099)(22.373) [FGF23](http://www.ncbi.nlm.nih.gov/gene/?term=8074)(22.373)[PGR](http://www.ncbi.nlm.nih.gov/gene/?term=5241)(22.373)[WNT4](http://www.ncbi.nlm.nih.gov/gene/?term=54361)(22.373)[TCF3](http://www.ncbi.nlm.nih.gov/gene/?term=6929)(22.373)[BAX](http://www.ncbi.nlm.nih.gov/gene/?term=581)(22.373) [KL](http://www.ncbi.nlm.nih.gov/gene/?term=9365)(22.373)[PML](http://www.ncbi.nlm.nih.gov/gene/?term=5371)(22.373)[B4GALT1](http://www.ncbi.nlm.nih.gov/gene/?term=2683)(22.373)[S100G](http://www.ncbi.nlm.nih.gov/gene/?term=795)(22.373)[IRX5](http://www.ncbi.nlm.nih.gov/gene/?term=10265)(22.373) [RXRA](http://www.ncbi.nlm.nih.gov/gene/?term=6256)(22.373)[NR1H4](http://www.ncbi.nlm.nih.gov/gene/?term=9971)(22.373)[CYP27B1](http://www.ncbi.nlm.nih.gov/gene/?term=1594)(22.373) |
| [Na](http://www.megabionet.org/tcmid/ingredient/24237/) | This compound doesn't have any potential target with score larger than 20. |
| [Gallic Acid-4-O-(6'-O-Galloyl)-Glucoside](http://www.megabionet.org/tcmid/ingredient/30996/) | This compound doesn't have any potential target with score larger than 20. |
| [Vaccarin](http://www.megabionet.org/tcmid/ingredient/22286/) | This compound doesn't have any potential target with score larger than 20. |
| [Rhein](http://www.megabionet.org/tcmid/ingredient/18744/) | [PPARG](http://www.ncbi.nlm.nih.gov/gene/?term=5468)(23.000)[FPGS](http://www.ncbi.nlm.nih.gov/gene/?term=2356)(23.000)[PTGS1](http://www.ncbi.nlm.nih.gov/gene/?term=5742)(23.000)[PTGS2](http://www.ncbi.nlm.nih.gov/gene/?term=5743)(23.000)[ALOX5](http://www.ncbi.nlm.nih.gov/gene/?term=240)(23.000) [MPL](http://www.ncbi.nlm.nih.gov/gene/?term=4352)(23.000)[TYMS](http://www.ncbi.nlm.nih.gov/gene/?term=7298)(23.000)[APOA2](http://www.ncbi.nlm.nih.gov/gene/?term=336)(22.373)[SHMT1](http://www.ncbi.nlm.nih.gov/gene/?term=6470)(22.373)[SERPINE1](http://www.ncbi.nlm.nih.gov/gene/?term=5054)(22.373) [IL1B](http://www.ncbi.nlm.nih.gov/gene/?term=3553)(22.373)[IL13](http://www.ncbi.nlm.nih.gov/gene/?term=3596)(22.373)[GGH](http://www.ncbi.nlm.nih.gov/gene/?term=8836)(22.373)[SERPINB7](http://www.ncbi.nlm.nih.gov/gene/?term=8710)(22.373) |
| [Dryobalanone](http://www.megabionet.org/tcmid/ingredient/6609/) | [SLC8A1](http://www.ncbi.nlm.nih.gov/gene/?term=6546)(48.000)[F12](http://www.ncbi.nlm.nih.gov/gene/?term=2161)(48.000)[TRPV1](http://www.ncbi.nlm.nih.gov/gene/?term=7442)(48.000)[AR](http://www.ncbi.nlm.nih.gov/gene/?term=367)(48.000)[NR3C1](http://www.ncbi.nlm.nih.gov/gene/?term=2908)(48.000) [FADS2](http://www.ncbi.nlm.nih.gov/gene/?term=9415)(48.000)[FADS1](http://www.ncbi.nlm.nih.gov/gene/?term=3992)(48.000)[PTGS1](http://www.ncbi.nlm.nih.gov/gene/?term=5742)(48.000)[PTGS2](http://www.ncbi.nlm.nih.gov/gene/?term=5743)(48.000)[ELOVL4](http://www.ncbi.nlm.nih.gov/gene/?term=6785)(48.000) [ANXA1](http://www.ncbi.nlm.nih.gov/gene/?term=301)(48.000)[HSD11B1](http://www.ncbi.nlm.nih.gov/gene/?term=3290)(23.000)[NOTCH2](http://www.ncbi.nlm.nih.gov/gene/?term=4853)(22.373)[HSD11B2](http://www.ncbi.nlm.nih.gov/gene/?term=3291)(22.373) |
| [Pinene](http://www.megabionet.org/tcmid/ingredient/24585/) | [KCND1](http://www.ncbi.nlm.nih.gov/gene/?term=3750)(80.882)[KCNA3](http://www.ncbi.nlm.nih.gov/gene/?term=3738)(80.882)[PRKAB1](http://www.ncbi.nlm.nih.gov/gene/?term=5564)(80.882)[ADH1A](http://www.ncbi.nlm.nih.gov/gene/?term=124)(80.882)[KCNA10](http://www.ncbi.nlm.nih.gov/gene/?term=3744)(80.882) [GAMT](http://www.ncbi.nlm.nih.gov/gene/?term=2593)(80.882)[KCNC3](http://www.ncbi.nlm.nih.gov/gene/?term=3748)(80.882)[KCNA1](http://www.ncbi.nlm.nih.gov/gene/?term=3736)(80.882)[KCNA2](http://www.ncbi.nlm.nih.gov/gene/?term=3737)(80.882)[TPO](http://www.ncbi.nlm.nih.gov/gene/?term=7173)(80.882) [CAT](http://www.ncbi.nlm.nih.gov/gene/?term=847)(80.882)[KCNB1](http://www.ncbi.nlm.nih.gov/gene/?term=3745)(80.882)[ADH1B](http://www.ncbi.nlm.nih.gov/gene/?term=125)(80.882)[DLG4](http://www.ncbi.nlm.nih.gov/gene/?term=1742)(80.882)[KCNC2](http://www.ncbi.nlm.nih.gov/gene/?term=3747)(80.882) [KCNC1](http://www.ncbi.nlm.nih.gov/gene/?term=3746)(80.882)[ADH1C](http://www.ncbi.nlm.nih.gov/gene/?term=126)(80.882)[KCNB2](http://www.ncbi.nlm.nih.gov/gene/?term=9312)(80.882)[KCNA5](http://www.ncbi.nlm.nih.gov/gene/?term=3741)(80.882)[RNASE1](http://www.ncbi.nlm.nih.gov/gene/?term=6035)(80.882) [KCND2](http://www.ncbi.nlm.nih.gov/gene/?term=3751)(80.882)[ALDH2](http://www.ncbi.nlm.nih.gov/gene/?term=217)(80.882)[KCNA4](http://www.ncbi.nlm.nih.gov/gene/?term=3739)(80.882)[KCNA7](http://www.ncbi.nlm.nih.gov/gene/?term=3743)(80.882)[KCNA6](http://www.ncbi.nlm.nih.gov/gene/?term=3742)(80.882) [KCND3](http://www.ncbi.nlm.nih.gov/gene/?term=3752)(80.882)[GUCY1B3](http://www.ncbi.nlm.nih.gov/gene/?term=2983)(55.444)[GATM](http://www.ncbi.nlm.nih.gov/gene/?term=2628)(55.444)[KCNK4](http://www.ncbi.nlm.nih.gov/gene/?term=50801)(55.444)[IYD](http://www.ncbi.nlm.nih.gov/gene/?term=389434)(55.444) [KCNQ1](http://www.ncbi.nlm.nih.gov/gene/?term=3784)(55.444)[FBP1](http://www.ncbi.nlm.nih.gov/gene/?term=2203)(22.373)[DGKI](http://www.ncbi.nlm.nih.gov/gene/?term=9162)(22.373)[ADH7](http://www.ncbi.nlm.nih.gov/gene/?term=131)(22.373)[NFIB](http://www.ncbi.nlm.nih.gov/gene/?term=4781)(22.373) [CYGB](http://www.ncbi.nlm.nih.gov/gene/?term=114757)(22.373)[NRXN3](http://www.ncbi.nlm.nih.gov/gene/?term=9369)(22.373)[LRRC4B](http://www.ncbi.nlm.nih.gov/gene/?term=94030)(22.373)[AQP8](http://www.ncbi.nlm.nih.gov/gene/?term=343)(22.373)[ARX](http://www.ncbi.nlm.nih.gov/gene/?term=170302)(22.373) [IGF1](http://www.ncbi.nlm.nih.gov/gene/?term=3479)(22.373)[SHANK3](http://www.ncbi.nlm.nih.gov/gene/?term=85358)(22.373)[ACY3](http://www.ncbi.nlm.nih.gov/gene/?term=91703)(22.373)[ASPA](http://www.ncbi.nlm.nih.gov/gene/?term=443)(22.373)[NRXN1](http://www.ncbi.nlm.nih.gov/gene/?term=9378)(22.373) [EDN1](http://www.ncbi.nlm.nih.gov/gene/?term=1906)(22.373)[OXT](http://www.ncbi.nlm.nih.gov/gene/?term=5020)(22.373)[MAGI2](http://www.ncbi.nlm.nih.gov/gene/?term=9863)(22.373)[APOE](http://www.ncbi.nlm.nih.gov/gene/?term=348)(22.373)[SOX15](http://www.ncbi.nlm.nih.gov/gene/?term=6665)(22.373) [CNTNAP4](http://www.ncbi.nlm.nih.gov/gene/?term=85445)(22.373)[RNASE4](http://www.ncbi.nlm.nih.gov/gene/?term=6038)(22.373)[RAB3A](http://www.ncbi.nlm.nih.gov/gene/?term=5864)(22.373)[MC4R](http://www.ncbi.nlm.nih.gov/gene/?term=4160)(22.373)[IL1B](http://www.ncbi.nlm.nih.gov/gene/?term=3553)(22.373) [GRIN2A](http://www.ncbi.nlm.nih.gov/gene/?term=2903)(22.373)[SLC17A7](http://www.ncbi.nlm.nih.gov/gene/?term=57030)(22.373)[CYP11A1](http://www.ncbi.nlm.nih.gov/gene/?term=1583)(22.373)[SCN5A](http://www.ncbi.nlm.nih.gov/gene/?term=6331)(22.373)[SCN10A](http://www.ncbi.nlm.nih.gov/gene/?term=6336)(22.373) [SPARC](http://www.ncbi.nlm.nih.gov/gene/?term=6678)(22.373)[SORCS3](http://www.ncbi.nlm.nih.gov/gene/?term=22986)(22.373)[RNASE8](http://www.ncbi.nlm.nih.gov/gene/?term=122665)(22.373)[KCNIP2](http://www.ncbi.nlm.nih.gov/gene/?term=30819)(22.373)[GPX7](http://www.ncbi.nlm.nih.gov/gene/?term=2882)(22.373) [CRLF1](http://www.ncbi.nlm.nih.gov/gene/?term=9244)(22.373)[FADD](http://www.ncbi.nlm.nih.gov/gene/?term=8772)(22.373)[SNTG2](http://www.ncbi.nlm.nih.gov/gene/?term=54221)(22.373)[SCGB1A1](http://www.ncbi.nlm.nih.gov/gene/?term=7356)(22.373)[OXTR](http://www.ncbi.nlm.nih.gov/gene/?term=5021)(22.373) [ALDH1B1](http://www.ncbi.nlm.nih.gov/gene/?term=219)(22.373)[LEP](http://www.ncbi.nlm.nih.gov/gene/?term=3952)(22.373)[NLGN1](http://www.ncbi.nlm.nih.gov/gene/?term=22871)(22.373)[CACNA1D](http://www.ncbi.nlm.nih.gov/gene/?term=776)(22.373)[RAPGEF2](http://www.ncbi.nlm.nih.gov/gene/?term=9693)(22.373) [DARS](http://www.ncbi.nlm.nih.gov/gene/?term=1615)(22.373)[ANK3](http://www.ncbi.nlm.nih.gov/gene/?term=288)(22.373)[KCNE5](http://www.ncbi.nlm.nih.gov/gene/?term=23630)(22.373)[PPARD](http://www.ncbi.nlm.nih.gov/gene/?term=5467)(22.373)[NKX2-1](http://www.ncbi.nlm.nih.gov/gene/?term=7080)(22.373) [MIP](http://www.ncbi.nlm.nih.gov/gene/?term=4284)(22.373)[RYR3](http://www.ncbi.nlm.nih.gov/gene/?term=6263)(22.373)[ZPR1](http://www.ncbi.nlm.nih.gov/gene/?term=8882)(22.373)[ALDH3B1](http://www.ncbi.nlm.nih.gov/gene/?term=221)(22.373)[NPPA](http://www.ncbi.nlm.nih.gov/gene/?term=4878)(22.373) [STX1A](http://www.ncbi.nlm.nih.gov/gene/?term=6804)(22.373)[PAXBP1](http://www.ncbi.nlm.nih.gov/gene/?term=94104)(22.373)[PAX7](http://www.ncbi.nlm.nih.gov/gene/?term=5081)(22.373)[ALDH3B2](http://www.ncbi.nlm.nih.gov/gene/?term=222)(22.373)[RNASE2](http://www.ncbi.nlm.nih.gov/gene/?term=6036)(22.373) [NRXN2](http://www.ncbi.nlm.nih.gov/gene/?term=9379)(22.373)[ADORA1](http://www.ncbi.nlm.nih.gov/gene/?term=134)(22.373)[ADH4](http://www.ncbi.nlm.nih.gov/gene/?term=127)(22.373)[GJA5](http://www.ncbi.nlm.nih.gov/gene/?term=2702)(22.373)[FAS](http://www.ncbi.nlm.nih.gov/gene/?term=355)(22.373) [DAB2IP](http://www.ncbi.nlm.nih.gov/gene/?term=153090)(22.373)[CYP2E1](http://www.ncbi.nlm.nih.gov/gene/?term=1571)(22.373)[ACY1](http://www.ncbi.nlm.nih.gov/gene/?term=95)(22.373) |
| [Vanillin](http://www.megabionet.org/tcmid/ingredient/22320/) | This compound doesn't have any potential target with score larger than 20. |
| [Isocorypalmine](http://www.megabionet.org/tcmid/ingredient/11345/) | [ACHE](http://www.ncbi.nlm.nih.gov/gene/?term=43)(122.778)[CHRNA2](http://www.ncbi.nlm.nih.gov/gene/?term=1135)(122.778)[HTR3A](http://www.ncbi.nlm.nih.gov/gene/?term=3359)(122.778)[CHRM2](http://www.ncbi.nlm.nih.gov/gene/?term=1129)(122.778)[CHRM3](http://www.ncbi.nlm.nih.gov/gene/?term=1131)(48.000) [DRD2](http://www.ncbi.nlm.nih.gov/gene/?term=1813)(48.000)[SLC18A2](http://www.ncbi.nlm.nih.gov/gene/?term=6571)(48.000)[BCHE](http://www.ncbi.nlm.nih.gov/gene/?term=590)(48.000)[ESR2](http://www.ncbi.nlm.nih.gov/gene/?term=2100)(23.000)[OPRD1](http://www.ncbi.nlm.nih.gov/gene/?term=4985)(23.000) [ESR1](http://www.ncbi.nlm.nih.gov/gene/?term=2099)(23.000)[OPRK1](http://www.ncbi.nlm.nih.gov/gene/?term=4986)(23.000)[OPRM1](http://www.ncbi.nlm.nih.gov/gene/?term=4988)(23.000) |
| [Rubricauloside](http://www.megabionet.org/tcmid/ingredient/19025/) | This compound doesn't have any potential target with score larger than 20. |
| [2-Tridecanone](http://www.megabionet.org/tcmid/ingredient/21596/) | [SCN11A](http://www.ncbi.nlm.nih.gov/gene/?term=11280)(48.000)[SCN2B](http://www.ncbi.nlm.nih.gov/gene/?term=6327)(48.000)[ABAT](http://www.ncbi.nlm.nih.gov/gene/?term=18)(48.000)[SCN1A](http://www.ncbi.nlm.nih.gov/gene/?term=6323)(48.000)[SCN3B](http://www.ncbi.nlm.nih.gov/gene/?term=55800)(48.000) [SCN3A](http://www.ncbi.nlm.nih.gov/gene/?term=6328)(48.000)[SCN7A](http://www.ncbi.nlm.nih.gov/gene/?term=6332)(48.000)[SCN5A](http://www.ncbi.nlm.nih.gov/gene/?term=6331)(48.000)[SCN10A](http://www.ncbi.nlm.nih.gov/gene/?term=6336)(48.000)[AKR1D1](http://www.ncbi.nlm.nih.gov/gene/?term=6718)(48.000) [ALDH5A1](http://www.ncbi.nlm.nih.gov/gene/?term=7915)(48.000)[SCN2A](http://www.ncbi.nlm.nih.gov/gene/?term=6326)(48.000)[HDAC9](http://www.ncbi.nlm.nih.gov/gene/?term=9734)(48.000)[TYR](http://www.ncbi.nlm.nih.gov/gene/?term=7299)(48.000)[SCN9A](http://www.ncbi.nlm.nih.gov/gene/?term=6335)(48.000) [SCN4A](http://www.ncbi.nlm.nih.gov/gene/?term=6329)(48.000)[SCN4B](http://www.ncbi.nlm.nih.gov/gene/?term=6330)(48.000)[SRD5A2](http://www.ncbi.nlm.nih.gov/gene/?term=6716)(48.000)[ACADSB](http://www.ncbi.nlm.nih.gov/gene/?term=36)(48.000)[SCN8A](http://www.ncbi.nlm.nih.gov/gene/?term=6334)(48.000) [SCN1B](http://www.ncbi.nlm.nih.gov/gene/?term=6324)(48.000)[OGDH](http://www.ncbi.nlm.nih.gov/gene/?term=4967)(48.000)[HDAC2](http://www.ncbi.nlm.nih.gov/gene/?term=3066)(48.000) |
| [Vicianin](http://www.megabionet.org/tcmid/ingredient/22449/) | This compound doesn't have any potential target with score larger than 20. |
| [Tetrahydrocyperaguinone](http://www.megabionet.org/tcmid/ingredient/21047/) | This compound doesn't have any potential target with score larger than 20. |
| [6-O-Galloyl-Glucose](http://www.megabionet.org/tcmid/ingredient/8108/) | This compound doesn't have any potential target with score larger than 20. |
| [2-O-Cinnamoyl-Glucogallin](http://www.megabionet.org/tcmid/ingredient/3703/) | This compound doesn't have any potential target with score larger than 20. |
| [Allocryptopine](http://www.megabionet.org/tcmid/ingredient/930/) | [CACNB1](http://www.ncbi.nlm.nih.gov/gene/?term=782)(23.000)[ADRA1A](http://www.ncbi.nlm.nih.gov/gene/?term=148)(23.000)[SCN1A](http://www.ncbi.nlm.nih.gov/gene/?term=6323)(23.000)[CACNA1F](http://www.ncbi.nlm.nih.gov/gene/?term=778)(23.000)[HTR2A](http://www.ncbi.nlm.nih.gov/gene/?term=3356)(23.000) [OPRD1](http://www.ncbi.nlm.nih.gov/gene/?term=4985)(23.000)[ADRA2A](http://www.ncbi.nlm.nih.gov/gene/?term=150)(23.000)[KCNK2](http://www.ncbi.nlm.nih.gov/gene/?term=3776)(23.000)[SCN5A](http://www.ncbi.nlm.nih.gov/gene/?term=6331)(23.000)[SCN10A](http://www.ncbi.nlm.nih.gov/gene/?term=6336)(23.000) [CACNA1S](http://www.ncbi.nlm.nih.gov/gene/?term=779)(23.000)[ADRB1](http://www.ncbi.nlm.nih.gov/gene/?term=153)(23.000)[CACNB4](http://www.ncbi.nlm.nih.gov/gene/?term=785)(23.000)[ADRA1D](http://www.ncbi.nlm.nih.gov/gene/?term=146)(23.000)[DRD2](http://www.ncbi.nlm.nih.gov/gene/?term=1813)(23.000) [CACNA1C](http://www.ncbi.nlm.nih.gov/gene/?term=775)(23.000)[CACNA1D](http://www.ncbi.nlm.nih.gov/gene/?term=776)(23.000)[OPRM1](http://www.ncbi.nlm.nih.gov/gene/?term=4988)(23.000)[ACHE](http://www.ncbi.nlm.nih.gov/gene/?term=43)(23.000)[CACNB3](http://www.ncbi.nlm.nih.gov/gene/?term=784)(23.000) [KCNH2](http://www.ncbi.nlm.nih.gov/gene/?term=3757)(23.000)[SLC18A2](http://www.ncbi.nlm.nih.gov/gene/?term=6571)(23.000)[ADRA1B](http://www.ncbi.nlm.nih.gov/gene/?term=147)(23.000)[ADRA2C](http://www.ncbi.nlm.nih.gov/gene/?term=152)(23.000)[CACNB2](http://www.ncbi.nlm.nih.gov/gene/?term=783)(23.000) [ADRA2B](http://www.ncbi.nlm.nih.gov/gene/?term=151)(23.000)[SLC17A7](http://www.ncbi.nlm.nih.gov/gene/?term=57030)(22.373)[HTR2C](http://www.ncbi.nlm.nih.gov/gene/?term=3358)(22.373)[COLQ](http://www.ncbi.nlm.nih.gov/gene/?term=8292)(22.373)[CNR1](http://www.ncbi.nlm.nih.gov/gene/?term=1268)(22.373) [SCN1B](http://www.ncbi.nlm.nih.gov/gene/?term=6324)(22.373)[WLS](http://www.ncbi.nlm.nih.gov/gene/?term=79971)(22.373)[BCHE](http://www.ncbi.nlm.nih.gov/gene/?term=590)(22.373)[CD34](http://www.ncbi.nlm.nih.gov/gene/?term=947)(22.373) |
| [Geranyl Acetate](http://www.megabionet.org/tcmid/ingredient/8312/) | This compound doesn't have any potential target with score larger than 20. |
| [Methyl Leptol B](http://www.megabionet.org/tcmid/ingredient/14543/) | [ADRA1A](http://www.ncbi.nlm.nih.gov/gene/?term=148)(23.000)[ADRA1D](http://www.ncbi.nlm.nih.gov/gene/?term=146)(23.000)[KCNH2](http://www.ncbi.nlm.nih.gov/gene/?term=3757)(23.000)[ADRA1B](http://www.ncbi.nlm.nih.gov/gene/?term=147)(23.000) |
| [2-Cinnamoyl-Glucose](http://www.megabionet.org/tcmid/ingredient/3706/) | This compound doesn't have any potential target with score larger than 20. |
| [(+)-Catechin-5-O-Glucoside](http://www.megabionet.org/tcmid/ingredient/3315/) | This compound doesn't have any potential target with score larger than 20. |
| [Rhamnocitrin](http://www.megabionet.org/tcmid/ingredient/18667/) | This compound doesn't have any potential target with score larger than 20. |
| [Yuanhunine](http://www.megabionet.org/tcmid/ingredient/22918/) | [ACHE](http://www.ncbi.nlm.nih.gov/gene/?term=43)(122.778)[CHRNA2](http://www.ncbi.nlm.nih.gov/gene/?term=1135)(122.778)[HTR3A](http://www.ncbi.nlm.nih.gov/gene/?term=3359)(122.778)[CHRM2](http://www.ncbi.nlm.nih.gov/gene/?term=1129)(122.778)[CHRM3](http://www.ncbi.nlm.nih.gov/gene/?term=1131)(48.000) [DRD2](http://www.ncbi.nlm.nih.gov/gene/?term=1813)(48.000)[SLC18A2](http://www.ncbi.nlm.nih.gov/gene/?term=6571)(48.000)[BCHE](http://www.ncbi.nlm.nih.gov/gene/?term=590)(48.000)[ESR2](http://www.ncbi.nlm.nih.gov/gene/?term=2100)(23.000)[OPRD1](http://www.ncbi.nlm.nih.gov/gene/?term=4985)(23.000) [ESR1](http://www.ncbi.nlm.nih.gov/gene/?term=2099)(23.000)[OPRK1](http://www.ncbi.nlm.nih.gov/gene/?term=4986)(23.000)[OPRM1](http://www.ncbi.nlm.nih.gov/gene/?term=4988)(23.000) |
| [Borneol](http://www.megabionet.org/tcmid/ingredient/2550/) | [TRPV3](http://www.ncbi.nlm.nih.gov/gene/?term=162514)(122.778)[OPRK1](http://www.ncbi.nlm.nih.gov/gene/?term=4986)(122.778)[TRPM8](http://www.ncbi.nlm.nih.gov/gene/?term=79054)(122.778)[TRPA1](http://www.ncbi.nlm.nih.gov/gene/?term=8989)(122.778)[KCNK4](http://www.ncbi.nlm.nih.gov/gene/?term=50801)(55.444) [ESRRG](http://www.ncbi.nlm.nih.gov/gene/?term=2104)(48.000)[COX6C](http://www.ncbi.nlm.nih.gov/gene/?term=1345)(48.000)[COX5B](http://www.ncbi.nlm.nih.gov/gene/?term=1329)(48.000)[COX7C](http://www.ncbi.nlm.nih.gov/gene/?term=1350)(48.000)[COX1](http://www.ncbi.nlm.nih.gov/gene/?term=4512)(48.000) [AKR1C2](http://www.ncbi.nlm.nih.gov/gene/?term=1646)(48.000)[COX5A](http://www.ncbi.nlm.nih.gov/gene/?term=9377)(48.000)[COX3](http://www.ncbi.nlm.nih.gov/gene/?term=4514)(48.000)[AR](http://www.ncbi.nlm.nih.gov/gene/?term=367)(48.000)[COX7A1](http://www.ncbi.nlm.nih.gov/gene/?term=1346)(48.000) [FECH](http://www.ncbi.nlm.nih.gov/gene/?term=2235)(48.000)[COX4I1](http://www.ncbi.nlm.nih.gov/gene/?term=1327)(48.000)[PLA2G1B](http://www.ncbi.nlm.nih.gov/gene/?term=5319)(48.000)[COX6A2](http://www.ncbi.nlm.nih.gov/gene/?term=1339)(48.000)[ADH1C](http://www.ncbi.nlm.nih.gov/gene/?term=126)(48.000) [COX6B1](http://www.ncbi.nlm.nih.gov/gene/?term=1340)(48.000)[FABP6](http://www.ncbi.nlm.nih.gov/gene/?term=2172)(48.000)[CES1](http://www.ncbi.nlm.nih.gov/gene/?term=1066)(48.000)[COX7B](http://www.ncbi.nlm.nih.gov/gene/?term=1349)(48.000)[COX2](http://www.ncbi.nlm.nih.gov/gene/?term=4513)(48.000) [COX8A](http://www.ncbi.nlm.nih.gov/gene/?term=1351)(48.000)[NR1H4](http://www.ncbi.nlm.nih.gov/gene/?term=9971)(48.000)[ACTN3](http://www.ncbi.nlm.nih.gov/gene/?term=89)(22.373)[TAC1](http://www.ncbi.nlm.nih.gov/gene/?term=6863)(22.373)[NGFR](http://www.ncbi.nlm.nih.gov/gene/?term=4804)(22.373) [ADRA2A](http://www.ncbi.nlm.nih.gov/gene/?term=150)(22.373)[TRPV1](http://www.ncbi.nlm.nih.gov/gene/?term=7442)(22.373)[FGF10](http://www.ncbi.nlm.nih.gov/gene/?term=2255)(22.373)[VCP](http://www.ncbi.nlm.nih.gov/gene/?term=7415)(22.373)[HIF1A](http://www.ncbi.nlm.nih.gov/gene/?term=3091)(22.373) [BNIP3](http://www.ncbi.nlm.nih.gov/gene/?term=664)(22.373)[CRH](http://www.ncbi.nlm.nih.gov/gene/?term=1392)(22.373)[NEFL](http://www.ncbi.nlm.nih.gov/gene/?term=4747)(22.373)[UCN2](http://www.ncbi.nlm.nih.gov/gene/?term=90226)(22.373)[SMO](http://www.ncbi.nlm.nih.gov/gene/?term=6608)(22.373) [TACR2](http://www.ncbi.nlm.nih.gov/gene/?term=6865)(22.373)[AQP1](http://www.ncbi.nlm.nih.gov/gene/?term=358)(22.373)[TAC4](http://www.ncbi.nlm.nih.gov/gene/?term=255061)(22.373)[CDH3](http://www.ncbi.nlm.nih.gov/gene/?term=1001)(22.373)[CBFA2T3](http://www.ncbi.nlm.nih.gov/gene/?term=863)(22.373) [NEFH](http://www.ncbi.nlm.nih.gov/gene/?term=4744)(22.373)[CRHR2](http://www.ncbi.nlm.nih.gov/gene/?term=1395)(22.373) |
| [Kaempferol](http://www.megabionet.org/tcmid/ingredient/12017/) | [HSD17B1](http://www.ncbi.nlm.nih.gov/gene/?term=3292)(26.373)[CRYZ](http://www.ncbi.nlm.nih.gov/gene/?term=1429)(23.000)[VKORC1](http://www.ncbi.nlm.nih.gov/gene/?term=79001)(23.000)[NQO1](http://www.ncbi.nlm.nih.gov/gene/?term=1728)(23.000) |
| [Melanin](http://www.megabionet.org/tcmid/ingredient/13652/) | [DRD2](http://www.ncbi.nlm.nih.gov/gene/?term=1813)(114.143)[DRD4](http://www.ncbi.nlm.nih.gov/gene/?term=1815)(114.143)[DRD3](http://www.ncbi.nlm.nih.gov/gene/?term=1814)(114.143)[DRD5](http://www.ncbi.nlm.nih.gov/gene/?term=1816)(114.143)[DRD1](http://www.ncbi.nlm.nih.gov/gene/?term=1812)(114.143) [PDE7B](http://www.ncbi.nlm.nih.gov/gene/?term=27115)(80.882)[PDE5A](http://www.ncbi.nlm.nih.gov/gene/?term=8654)(80.882)[PDE9A](http://www.ncbi.nlm.nih.gov/gene/?term=5152)(80.882)[PDE4A](http://www.ncbi.nlm.nih.gov/gene/?term=5141)(80.882)[RYR1](http://www.ncbi.nlm.nih.gov/gene/?term=6261)(80.882) [CYP17A1](http://www.ncbi.nlm.nih.gov/gene/?term=1586)(80.882)[PDE3A](http://www.ncbi.nlm.nih.gov/gene/?term=5139)(80.882)[ADORA2A](http://www.ncbi.nlm.nih.gov/gene/?term=135)(80.882)[ITPR1](http://www.ncbi.nlm.nih.gov/gene/?term=3708)(80.882)[PDE6A](http://www.ncbi.nlm.nih.gov/gene/?term=5145)(80.882) [PRKDC](http://www.ncbi.nlm.nih.gov/gene/?term=5591)(80.882)[PIK3CD](http://www.ncbi.nlm.nih.gov/gene/?term=5293)(80.882)[ESR1](http://www.ncbi.nlm.nih.gov/gene/?term=2099)(80.882)[PDE3B](http://www.ncbi.nlm.nih.gov/gene/?term=5140)(80.882)[PGD](http://www.ncbi.nlm.nih.gov/gene/?term=5226)(80.882) [PIK3CA](http://www.ncbi.nlm.nih.gov/gene/?term=5290)(80.882)[ADORA2B](http://www.ncbi.nlm.nih.gov/gene/?term=136)(80.882)[PDE1A](http://www.ncbi.nlm.nih.gov/gene/?term=5136)(80.882)[PDE4D](http://www.ncbi.nlm.nih.gov/gene/?term=5144)(80.882)[PGR](http://www.ncbi.nlm.nih.gov/gene/?term=5241)(80.882) [PIK3CB](http://www.ncbi.nlm.nih.gov/gene/?term=5291)(80.882)[ITPR2](http://www.ncbi.nlm.nih.gov/gene/?term=3709)(80.882)[PDE1B](http://www.ncbi.nlm.nih.gov/gene/?term=5153)(80.882)[OPRK1](http://www.ncbi.nlm.nih.gov/gene/?term=4986)(80.882)[PDE7A](http://www.ncbi.nlm.nih.gov/gene/?term=5150)(80.882) [ITPR3](http://www.ncbi.nlm.nih.gov/gene/?term=3710)(80.882)[POLA2](http://www.ncbi.nlm.nih.gov/gene/?term=23649)(80.882)[PDE4C](http://www.ncbi.nlm.nih.gov/gene/?term=5143)(80.882)[PDE10A](http://www.ncbi.nlm.nih.gov/gene/?term=10846)(80.882)[PDE1C](http://www.ncbi.nlm.nih.gov/gene/?term=5137)(80.882) [ATM](http://www.ncbi.nlm.nih.gov/gene/?term=472)(80.882)[PDE6B](http://www.ncbi.nlm.nih.gov/gene/?term=5158)(80.882)[PDE4B](http://www.ncbi.nlm.nih.gov/gene/?term=5142)(80.882)[PDE2A](http://www.ncbi.nlm.nih.gov/gene/?term=5138)(80.882)[ADORA1](http://www.ncbi.nlm.nih.gov/gene/?term=134)(80.882) [CYP19A1](http://www.ncbi.nlm.nih.gov/gene/?term=1588)(80.882)[PDE8B](http://www.ncbi.nlm.nih.gov/gene/?term=8622)(80.882)[NT5E](http://www.ncbi.nlm.nih.gov/gene/?term=4907)(80.882)[HDAC2](http://www.ncbi.nlm.nih.gov/gene/?term=3066)(80.882)[PDE6C](http://www.ncbi.nlm.nih.gov/gene/?term=5146)(80.882) [PDE11A](http://www.ncbi.nlm.nih.gov/gene/?term=50940)(80.882)[PDE8A](http://www.ncbi.nlm.nih.gov/gene/?term=5151)(80.882)[NR3C2](http://www.ncbi.nlm.nih.gov/gene/?term=4306)(80.882)[RINT1](http://www.ncbi.nlm.nih.gov/gene/?term=60561)(55.444)[RIPK1](http://www.ncbi.nlm.nih.gov/gene/?term=8737)(55.444) [PIK3R1](http://www.ncbi.nlm.nih.gov/gene/?term=5295)(55.444)[WNT4](http://www.ncbi.nlm.nih.gov/gene/?term=54361)(55.444)[TACR2](http://www.ncbi.nlm.nih.gov/gene/?term=6865)(55.444)[AMPD3](http://www.ncbi.nlm.nih.gov/gene/?term=272)(55.444)[CX3CR1](http://www.ncbi.nlm.nih.gov/gene/?term=1524)(55.444) [ADA](http://www.ncbi.nlm.nih.gov/gene/?term=100)(55.444)[HAP1](http://www.ncbi.nlm.nih.gov/gene/?term=9001)(55.444)[IDNK](http://www.ncbi.nlm.nih.gov/gene/?term=414328)(55.444)[SLC6A3](http://www.ncbi.nlm.nih.gov/gene/?term=6531)(25.857)[TH](http://www.ncbi.nlm.nih.gov/gene/?term=7054)(25.857) [TAT](http://www.ncbi.nlm.nih.gov/gene/?term=6898)(25.857)[SLC6A4](http://www.ncbi.nlm.nih.gov/gene/?term=6532)(25.857)[HTR7](http://www.ncbi.nlm.nih.gov/gene/?term=3363)(25.857)[HTR1A](http://www.ncbi.nlm.nih.gov/gene/?term=3350)(25.857)[DBH](http://www.ncbi.nlm.nih.gov/gene/?term=1621)(25.857) [YARS](http://www.ncbi.nlm.nih.gov/gene/?term=8565)(25.857)[YARS2](http://www.ncbi.nlm.nih.gov/gene/?term=51067)(25.857)[SLC6A2](http://www.ncbi.nlm.nih.gov/gene/?term=6530)(25.857)[HDAC1](http://www.ncbi.nlm.nih.gov/gene/?term=3065)(23.000)[TFAP2C](http://www.ncbi.nlm.nih.gov/gene/?term=7022)(22.373) [HMGA2](http://www.ncbi.nlm.nih.gov/gene/?term=8091)(22.373)[TNFSF11](http://www.ncbi.nlm.nih.gov/gene/?term=8600)(22.373)[NT5C1A](http://www.ncbi.nlm.nih.gov/gene/?term=84618)(22.373)[ACTN3](http://www.ncbi.nlm.nih.gov/gene/?term=89)(22.373)[NOS1AP](http://www.ncbi.nlm.nih.gov/gene/?term=9722)(22.373) [TAC1](http://www.ncbi.nlm.nih.gov/gene/?term=6863)(22.373)[PHB](http://www.ncbi.nlm.nih.gov/gene/?term=5245)(22.373)[SIRT1](http://www.ncbi.nlm.nih.gov/gene/?term=23411)(22.373)[DGKI](http://www.ncbi.nlm.nih.gov/gene/?term=9162)(22.373)[FKBP1A](http://www.ncbi.nlm.nih.gov/gene/?term=2280)(22.373) [FURIN](http://www.ncbi.nlm.nih.gov/gene/?term=5045)(22.373)[TOX3](http://www.ncbi.nlm.nih.gov/gene/?term=27324)(22.373)[UTS2](http://www.ncbi.nlm.nih.gov/gene/?term=10911)(22.373)[STUB1](http://www.ncbi.nlm.nih.gov/gene/?term=10273)(22.373)[CTR9](http://www.ncbi.nlm.nih.gov/gene/?term=9646)(22.373) [G6PD](http://www.ncbi.nlm.nih.gov/gene/?term=2539)(22.373)[GNAT1](http://www.ncbi.nlm.nih.gov/gene/?term=2779)(22.373)[SIX1](http://www.ncbi.nlm.nih.gov/gene/?term=6495)(22.373)[QDPR](http://www.ncbi.nlm.nih.gov/gene/?term=5860)(22.373)[TGFB1](http://www.ncbi.nlm.nih.gov/gene/?term=7040)(22.373) [ATP11C](http://www.ncbi.nlm.nih.gov/gene/?term=286410)(22.373)[KHDRBS1](http://www.ncbi.nlm.nih.gov/gene/?term=10657)(22.373)[PPP3CB](http://www.ncbi.nlm.nih.gov/gene/?term=5532)(22.373)[SYT2](http://www.ncbi.nlm.nih.gov/gene/?term=127833)(22.373)[SIX4](http://www.ncbi.nlm.nih.gov/gene/?term=51804)(22.373) [SIRT2](http://www.ncbi.nlm.nih.gov/gene/?term=22933)(22.373)[SPI1](http://www.ncbi.nlm.nih.gov/gene/?term=6688)(22.373)[HOMER1](http://www.ncbi.nlm.nih.gov/gene/?term=9456)(22.373)[SHANK3](http://www.ncbi.nlm.nih.gov/gene/?term=85358)(22.373)[TAS1R1](http://www.ncbi.nlm.nih.gov/gene/?term=80835)(22.373) [TRIM28](http://www.ncbi.nlm.nih.gov/gene/?term=10155)(22.373)[XRCC4](http://www.ncbi.nlm.nih.gov/gene/?term=7518)(22.373)[MED1](http://www.ncbi.nlm.nih.gov/gene/?term=5469)(22.373)[NKX3-1](http://www.ncbi.nlm.nih.gov/gene/?term=4824)(22.373)[UBE2B](http://www.ncbi.nlm.nih.gov/gene/?term=7320)(22.373) [ESR2](http://www.ncbi.nlm.nih.gov/gene/?term=2100)(22.373)[HCN4](http://www.ncbi.nlm.nih.gov/gene/?term=10021)(22.373)[EDN1](http://www.ncbi.nlm.nih.gov/gene/?term=1906)(22.373)[CACNA1A](http://www.ncbi.nlm.nih.gov/gene/?term=773)(22.373)[STAT5A](http://www.ncbi.nlm.nih.gov/gene/?term=6776)(22.373) [TP53](http://www.ncbi.nlm.nih.gov/gene/?term=7157)(22.373)[GNAT3](http://www.ncbi.nlm.nih.gov/gene/?term=346562)(22.373)[HELB](http://www.ncbi.nlm.nih.gov/gene/?term=92797)(22.373)[COMT](http://www.ncbi.nlm.nih.gov/gene/?term=1312)(22.373)[SLC26A6](http://www.ncbi.nlm.nih.gov/gene/?term=65010)(22.373) [ADRA2A](http://www.ncbi.nlm.nih.gov/gene/?term=150)(22.373)[YWHAE](http://www.ncbi.nlm.nih.gov/gene/?term=7531)(22.373)[CETN2](http://www.ncbi.nlm.nih.gov/gene/?term=1069)(22.373)[POLA1](http://www.ncbi.nlm.nih.gov/gene/?term=5422)(22.373)[CRP](http://www.ncbi.nlm.nih.gov/gene/?term=1401)(22.373) [CETN1](http://www.ncbi.nlm.nih.gov/gene/?term=1068)(22.373)[NUDT9](http://www.ncbi.nlm.nih.gov/gene/?term=53343)(22.373)[PIK3CG](http://www.ncbi.nlm.nih.gov/gene/?term=5294)(22.373)[BMP2](http://www.ncbi.nlm.nih.gov/gene/?term=650)(22.373)[TNF](http://www.ncbi.nlm.nih.gov/gene/?term=7124)(22.373) [NEDD4](http://www.ncbi.nlm.nih.gov/gene/?term=4734)(22.373)[AGT](http://www.ncbi.nlm.nih.gov/gene/?term=183)(22.373)[FER](http://www.ncbi.nlm.nih.gov/gene/?term=2241)(22.373)[SHH](http://www.ncbi.nlm.nih.gov/gene/?term=6469)(22.373)[SERPINB3](http://www.ncbi.nlm.nih.gov/gene/?term=6317)(22.373) [FOXP3](http://www.ncbi.nlm.nih.gov/gene/?term=50943)(22.373)[CXCL13](http://www.ncbi.nlm.nih.gov/gene/?term=10563)(22.373)[ARID1A](http://www.ncbi.nlm.nih.gov/gene/?term=8289)(22.373)[FGF10](http://www.ncbi.nlm.nih.gov/gene/?term=2255)(22.373)[KCNB1](http://www.ncbi.nlm.nih.gov/gene/?term=3745)(22.373) [LEF1](http://www.ncbi.nlm.nih.gov/gene/?term=51176)(22.373)[PTK2B](http://www.ncbi.nlm.nih.gov/gene/?term=2185)(22.373)[LIG4](http://www.ncbi.nlm.nih.gov/gene/?term=3981)(22.373)[ADRBK1](http://www.ncbi.nlm.nih.gov/gene/?term=156)(22.373)[MAOB](http://www.ncbi.nlm.nih.gov/gene/?term=4129)(22.373) [SORCS3](http://www.ncbi.nlm.nih.gov/gene/?term=22986)(22.373)[ADRB1](http://www.ncbi.nlm.nih.gov/gene/?term=153)(22.373)[ADAM8](http://www.ncbi.nlm.nih.gov/gene/?term=101)(22.373)[VTI1A](http://www.ncbi.nlm.nih.gov/gene/?term=143187)(22.373)[SLC44A4](http://www.ncbi.nlm.nih.gov/gene/?term=80736)(22.373) [ADAP2](http://www.ncbi.nlm.nih.gov/gene/?term=55803)(22.373)[LRRC8A](http://www.ncbi.nlm.nih.gov/gene/?term=56262)(22.373)[FGFR2](http://www.ncbi.nlm.nih.gov/gene/?term=2263)(22.373)[AURKA](http://www.ncbi.nlm.nih.gov/gene/?term=6790)(22.373)[MCM3](http://www.ncbi.nlm.nih.gov/gene/?term=4172)(22.373) [VCP](http://www.ncbi.nlm.nih.gov/gene/?term=7415)(22.373)[GHRL](http://www.ncbi.nlm.nih.gov/gene/?term=51738)(22.373)[BCL11B](http://www.ncbi.nlm.nih.gov/gene/?term=64919)(22.373)[BCL2](http://www.ncbi.nlm.nih.gov/gene/?term=596)(22.373)[8-Mar](http://www.ncbi.nlm.nih.gov/gene/?term=220972)(22.373) [GAS6](http://www.ncbi.nlm.nih.gov/gene/?term=2621)(22.373)[C5](http://www.ncbi.nlm.nih.gov/gene/?term=727)(22.373)[SREBF1](http://www.ncbi.nlm.nih.gov/gene/?term=6720)(22.373)[PLN](http://www.ncbi.nlm.nih.gov/gene/?term=5350)(22.373)[DNM3](http://www.ncbi.nlm.nih.gov/gene/?term=26052)(22.373) [CHRNA3](http://www.ncbi.nlm.nih.gov/gene/?term=1136)(22.373)[TCF3](http://www.ncbi.nlm.nih.gov/gene/?term=6929)(22.373)[SRC](http://www.ncbi.nlm.nih.gov/gene/?term=6714)(22.373)[LONP1](http://www.ncbi.nlm.nih.gov/gene/?term=9361)(22.373)[XCL1](http://www.ncbi.nlm.nih.gov/gene/?term=6375)(22.373) [HSP90AB1](http://www.ncbi.nlm.nih.gov/gene/?term=3326)(22.373)[HIBADH](http://www.ncbi.nlm.nih.gov/gene/?term=11112)(22.373)[STAP1](http://www.ncbi.nlm.nih.gov/gene/?term=26228)(22.373)[HRC](http://www.ncbi.nlm.nih.gov/gene/?term=3270)(22.373)[UTS2R](http://www.ncbi.nlm.nih.gov/gene/?term=2837)(22.373) [HMGCR](http://www.ncbi.nlm.nih.gov/gene/?term=3156)(22.373)[HIPK2](http://www.ncbi.nlm.nih.gov/gene/?term=28996)(22.373)[PPP3CA](http://www.ncbi.nlm.nih.gov/gene/?term=5530)(22.373)[RAPGEF2](http://www.ncbi.nlm.nih.gov/gene/?term=9693)(22.373)[TET1](http://www.ncbi.nlm.nih.gov/gene/?term=80312)(22.373) [C2CD5](http://www.ncbi.nlm.nih.gov/gene/?term=9847)(22.373)[CSF2](http://www.ncbi.nlm.nih.gov/gene/?term=1437)(22.373)[TALDO1](http://www.ncbi.nlm.nih.gov/gene/?term=6888)(22.373)[BDKRB2](http://www.ncbi.nlm.nih.gov/gene/?term=624)(22.373)[KCNH2](http://www.ncbi.nlm.nih.gov/gene/?term=3757)(22.373) [MMP28](http://www.ncbi.nlm.nih.gov/gene/?term=79148)(22.373)[HIF1A](http://www.ncbi.nlm.nih.gov/gene/?term=3091)(22.373)[KIT](http://www.ncbi.nlm.nih.gov/gene/?term=3815)(22.373)[INS](http://www.ncbi.nlm.nih.gov/gene/?term=3630)(22.373)[MTOR](http://www.ncbi.nlm.nih.gov/gene/?term=2475)(22.373) [WNT10B](http://www.ncbi.nlm.nih.gov/gene/?term=7480)(22.373)[SLC18A3](http://www.ncbi.nlm.nih.gov/gene/?term=6572)(22.373)[ABCC4](http://www.ncbi.nlm.nih.gov/gene/?term=10257)(22.373)[BNIP3](http://www.ncbi.nlm.nih.gov/gene/?term=664)(22.373)[ZFPM1](http://www.ncbi.nlm.nih.gov/gene/?term=161882)(22.373) [GATA3](http://www.ncbi.nlm.nih.gov/gene/?term=2625)(22.373)[UBR5](http://www.ncbi.nlm.nih.gov/gene/?term=51366)(22.373)[AREG](http://www.ncbi.nlm.nih.gov/gene/?term=374)(22.373)[CRH](http://www.ncbi.nlm.nih.gov/gene/?term=1392)(22.373)[ZFP42](http://www.ncbi.nlm.nih.gov/gene/?term=132625)(22.373) [PIK3R6](http://www.ncbi.nlm.nih.gov/gene/?term=146850)(22.373)[PF4](http://www.ncbi.nlm.nih.gov/gene/?term=5196)(22.373)[ATP1A2](http://www.ncbi.nlm.nih.gov/gene/?term=477)(22.373)[AVPR2](http://www.ncbi.nlm.nih.gov/gene/?term=554)(22.373)[CAMK2D](http://www.ncbi.nlm.nih.gov/gene/?term=817)(22.373) [ADRA1B](http://www.ncbi.nlm.nih.gov/gene/?term=147)(22.373)[IL10](http://www.ncbi.nlm.nih.gov/gene/?term=3586)(22.373)[NEFL](http://www.ncbi.nlm.nih.gov/gene/?term=4747)(22.373)[UCN2](http://www.ncbi.nlm.nih.gov/gene/?term=90226)(22.373)[ATP1A1](http://www.ncbi.nlm.nih.gov/gene/?term=476)(22.373) [RYR3](http://www.ncbi.nlm.nih.gov/gene/?term=6263)(22.373)[PPP3R1](http://www.ncbi.nlm.nih.gov/gene/?term=5534)(22.373)[SPX](http://www.ncbi.nlm.nih.gov/gene/?term=80763)(22.373)[ATP2A1](http://www.ncbi.nlm.nih.gov/gene/?term=487)(22.373)[SMO](http://www.ncbi.nlm.nih.gov/gene/?term=6608)(22.373) [RAG1](http://www.ncbi.nlm.nih.gov/gene/?term=5896)(22.373)[RAB8B](http://www.ncbi.nlm.nih.gov/gene/?term=51762)(22.373)[DKK3](http://www.ncbi.nlm.nih.gov/gene/?term=27122)(22.373)[AHCYL1](http://www.ncbi.nlm.nih.gov/gene/?term=10768)(22.373)[AQP1](http://www.ncbi.nlm.nih.gov/gene/?term=358)(22.373) [TNFAIP3](http://www.ncbi.nlm.nih.gov/gene/?term=7128)(22.373)[TAC4](http://www.ncbi.nlm.nih.gov/gene/?term=255061)(22.373)[DPPA3](http://www.ncbi.nlm.nih.gov/gene/?term=359787)(22.373)[CASQ1](http://www.ncbi.nlm.nih.gov/gene/?term=844)(22.373)[FKBP1B](http://www.ncbi.nlm.nih.gov/gene/?term=2281)(22.373) [CDC42](http://www.ncbi.nlm.nih.gov/gene/?term=998)(22.373)[IL4](http://www.ncbi.nlm.nih.gov/gene/?term=3565)(22.373)[POLB](http://www.ncbi.nlm.nih.gov/gene/?term=5423)(22.373)[IDO1](http://www.ncbi.nlm.nih.gov/gene/?term=3620)(22.373)[ATP2B4](http://www.ncbi.nlm.nih.gov/gene/?term=493)(22.373) [CTNNB1](http://www.ncbi.nlm.nih.gov/gene/?term=1499)(22.373)[PGLS](http://www.ncbi.nlm.nih.gov/gene/?term=25796)(22.373)[SLC9A3R1](http://www.ncbi.nlm.nih.gov/gene/?term=9368)(22.373)[IGF2](http://www.ncbi.nlm.nih.gov/gene/?term=3481)(22.373)[HPS4](http://www.ncbi.nlm.nih.gov/gene/?term=89781)(22.373) [TKT](http://www.ncbi.nlm.nih.gov/gene/?term=7086)(22.373)[ADAP1](http://www.ncbi.nlm.nih.gov/gene/?term=11033)(22.373)[EDA](http://www.ncbi.nlm.nih.gov/gene/?term=1896)(22.373)[RYR2](http://www.ncbi.nlm.nih.gov/gene/?term=6262)(22.373)[CD34](http://www.ncbi.nlm.nih.gov/gene/?term=947)(22.373) [HSP90AA1](http://www.ncbi.nlm.nih.gov/gene/?term=3320)(22.373)[LTA](http://www.ncbi.nlm.nih.gov/gene/?term=4049)(22.373)[SPR](http://www.ncbi.nlm.nih.gov/gene/?term=6697)(22.373)[GPER1](http://www.ncbi.nlm.nih.gov/gene/?term=2852)(22.373)[CBFA2T3](http://www.ncbi.nlm.nih.gov/gene/?term=863)(22.373) [CALHM1](http://www.ncbi.nlm.nih.gov/gene/?term=255022)(22.373)[WNT5A](http://www.ncbi.nlm.nih.gov/gene/?term=7474)(22.373)[SELP](http://www.ncbi.nlm.nih.gov/gene/?term=6403)(22.373)[XRCC6BP1](http://www.ncbi.nlm.nih.gov/gene/?term=91419)(22.373)[PARP10](http://www.ncbi.nlm.nih.gov/gene/?term=84875)(22.373) [HPRT1](http://www.ncbi.nlm.nih.gov/gene/?term=3251)(22.373)[AMICA1](http://www.ncbi.nlm.nih.gov/gene/?term=120425)(22.373)[TAS1R3](http://www.ncbi.nlm.nih.gov/gene/?term=83756)(22.373)[NOS1](http://www.ncbi.nlm.nih.gov/gene/?term=4842)(22.373)[TREM1](http://www.ncbi.nlm.nih.gov/gene/?term=54210)(22.373) [TAL1](http://www.ncbi.nlm.nih.gov/gene/?term=6886)(22.373)[ALAD](http://www.ncbi.nlm.nih.gov/gene/?term=210)(22.373)[PAWR](http://www.ncbi.nlm.nih.gov/gene/?term=5074)(22.373)[SOX9](http://www.ncbi.nlm.nih.gov/gene/?term=6662)(22.373)[BMP5](http://www.ncbi.nlm.nih.gov/gene/?term=653)(22.373) [PDGFB](http://www.ncbi.nlm.nih.gov/gene/?term=5155)(22.373)[RTN2](http://www.ncbi.nlm.nih.gov/gene/?term=6253)(22.373)[CD63](http://www.ncbi.nlm.nih.gov/gene/?term=967)(22.373)[CAMK2G](http://www.ncbi.nlm.nih.gov/gene/?term=818)(22.373)[LAMP2](http://www.ncbi.nlm.nih.gov/gene/?term=3920)(22.373) [NEFH](http://www.ncbi.nlm.nih.gov/gene/?term=4744)(22.373)[GLYR1](http://www.ncbi.nlm.nih.gov/gene/?term=84656)(22.373)[VDR](http://www.ncbi.nlm.nih.gov/gene/?term=7421)(22.373)[GNAS](http://www.ncbi.nlm.nih.gov/gene/?term=2778)(22.373)[NCBP2](http://www.ncbi.nlm.nih.gov/gene/?term=22916)(22.373) [TPM1](http://www.ncbi.nlm.nih.gov/gene/?term=7168)(22.373)[PIK3R5](http://www.ncbi.nlm.nih.gov/gene/?term=23533)(22.373)[PCSK6](http://www.ncbi.nlm.nih.gov/gene/?term=5046)(22.373)[TRIM24](http://www.ncbi.nlm.nih.gov/gene/?term=8805)(22.373)[REST](http://www.ncbi.nlm.nih.gov/gene/?term=5978)(22.373) [CRHR2](http://www.ncbi.nlm.nih.gov/gene/?term=1395)(22.373)[HCN2](http://www.ncbi.nlm.nih.gov/gene/?term=610)(22.373)[RAG2](http://www.ncbi.nlm.nih.gov/gene/?term=5897)(22.373)[NFX1](http://www.ncbi.nlm.nih.gov/gene/?term=4799)(22.373)[TAS1R2](http://www.ncbi.nlm.nih.gov/gene/?term=80834)(22.373) |
| [Cinnamaldehyde](http://www.megabionet.org/tcmid/ingredient/3693/) | [TRPV3](http://www.ncbi.nlm.nih.gov/gene/?term=162514)(373.000)[OPRK1](http://www.ncbi.nlm.nih.gov/gene/?term=4986)(373.000)[TRPM8](http://www.ncbi.nlm.nih.gov/gene/?term=79054)(373.000)[TRPA1](http://www.ncbi.nlm.nih.gov/gene/?term=8989)(373.000)[CNR2](http://www.ncbi.nlm.nih.gov/gene/?term=1269)(25.857) [CNR1](http://www.ncbi.nlm.nih.gov/gene/?term=1268)(25.857) |
| [Menthiafolin](http://www.megabionet.org/tcmid/ingredient/13766/) | [ATP1A1](http://www.ncbi.nlm.nih.gov/gene/?term=476)(80.882)[ATP1A3](http://www.ncbi.nlm.nih.gov/gene/?term=478)(23.000)[ATP1A2](http://www.ncbi.nlm.nih.gov/gene/?term=477)(23.000) |
| [Dehydrocostus Lactone](http://www.megabionet.org/tcmid/ingredient/23199/) | [CHRM3](http://www.ncbi.nlm.nih.gov/gene/?term=1131)(80.882)[CHRM1](http://www.ncbi.nlm.nih.gov/gene/?term=1128)(80.882)[CHRM2](http://www.ncbi.nlm.nih.gov/gene/?term=1129)(80.882)[PGR](http://www.ncbi.nlm.nih.gov/gene/?term=5241)(48.000)[AR](http://www.ncbi.nlm.nih.gov/gene/?term=367)(48.000) [NR3C2](http://www.ncbi.nlm.nih.gov/gene/?term=4306)(48.000)[ARFGEF2](http://www.ncbi.nlm.nih.gov/gene/?term=10564)(22.373)[DOCK5](http://www.ncbi.nlm.nih.gov/gene/?term=80005)(22.373)[NTSR1](http://www.ncbi.nlm.nih.gov/gene/?term=4923)(22.373)[CHRM5](http://www.ncbi.nlm.nih.gov/gene/?term=1133)(22.373) [GNA15](http://www.ncbi.nlm.nih.gov/gene/?term=2769)(22.373)[P2RX1](http://www.ncbi.nlm.nih.gov/gene/?term=5023)(22.373)[DOCK4](http://www.ncbi.nlm.nih.gov/gene/?term=9732)(22.373)[MAP2K1](http://www.ncbi.nlm.nih.gov/gene/?term=5604)(22.373)[CHRM4](http://www.ncbi.nlm.nih.gov/gene/?term=1132)(22.373) |
| [Isosaponarin](http://www.megabionet.org/tcmid/ingredient/11692/) | This compound doesn't have any potential target with score larger than 20. |
| [Casuariin](http://www.megabionet.org/tcmid/ingredient/3302/) | This compound doesn't have any potential target with score larger than 20. |
| [Erythrodiol](http://www.megabionet.org/tcmid/ingredient/7337/) | [ESR1](http://www.ncbi.nlm.nih.gov/gene/?term=2099)(122.778)[PGR](http://www.ncbi.nlm.nih.gov/gene/?term=5241)(122.778)[AR](http://www.ncbi.nlm.nih.gov/gene/?term=367)(48.000)[NR3C1](http://www.ncbi.nlm.nih.gov/gene/?term=2908)(48.000)[ANXA1](http://www.ncbi.nlm.nih.gov/gene/?term=301)(48.000) |
| [Curzerenone](http://www.megabionet.org/tcmid/ingredient/4415/) | [PDE5A](http://www.ncbi.nlm.nih.gov/gene/?term=8654)(23.000)[PDE11A](http://www.ncbi.nlm.nih.gov/gene/?term=50940)(23.000) |
| [1-Cinnamoyl-3-Hydroxy-11-Methoxymeliacarpinin](http://www.megabionet.org/tcmid/ingredient/30786/) | This compound doesn't have any potential target with score larger than 20. |
| [Protopine](http://www.megabionet.org/tcmid/ingredient/17969/) | [PDGFRB](http://www.ncbi.nlm.nih.gov/gene/?term=5159)(23.000)[NTRK1](http://www.ncbi.nlm.nih.gov/gene/?term=4914)(23.000)[CSF1R](http://www.ncbi.nlm.nih.gov/gene/?term=1436)(23.000)[PDGFRA](http://www.ncbi.nlm.nih.gov/gene/?term=5156)(23.000)[KIT](http://www.ncbi.nlm.nih.gov/gene/?term=3815)(23.000) [ABL1](http://www.ncbi.nlm.nih.gov/gene/?term=25)(23.000)[DDR1](http://www.ncbi.nlm.nih.gov/gene/?term=780)(23.000)[HTR3A](http://www.ncbi.nlm.nih.gov/gene/?term=3359)(23.000)[CSF1](http://www.ncbi.nlm.nih.gov/gene/?term=1435)(22.373)[EPOR](http://www.ncbi.nlm.nih.gov/gene/?term=2057)(22.373) [DDR2](http://www.ncbi.nlm.nih.gov/gene/?term=4921)(22.373)[PDGFRL](http://www.ncbi.nlm.nih.gov/gene/?term=5157)(22.373)[BICD1](http://www.ncbi.nlm.nih.gov/gene/?term=636)(22.373)[ZEB2](http://www.ncbi.nlm.nih.gov/gene/?term=9839)(22.373)[MST1R](http://www.ncbi.nlm.nih.gov/gene/?term=4486)(22.373) [NRP1](http://www.ncbi.nlm.nih.gov/gene/?term=8829)(22.373)[SORT1](http://www.ncbi.nlm.nih.gov/gene/?term=6272)(22.373) |
| [Hexadecane](http://www.megabionet.org/tcmid/ingredient/9484/) | This compound doesn't have any potential target with score larger than 20. |
| [Tetrahydropiperic Acid](http://www.megabionet.org/tcmid/ingredient/32026/) | This compound doesn't have any potential target with score larger than 20. |
| [Benzaldehyde](http://www.megabionet.org/tcmid/ingredient/2222/) | This compound doesn't have any potential target with score larger than 20. |
| [Citronellol](http://www.megabionet.org/tcmid/ingredient/3768/) | [RDH11](http://www.ncbi.nlm.nih.gov/gene/?term=51109)(80.882)[RBP3](http://www.ncbi.nlm.nih.gov/gene/?term=5949)(80.882)[RETSAT](http://www.ncbi.nlm.nih.gov/gene/?term=54884)(80.882)[RDH13](http://www.ncbi.nlm.nih.gov/gene/?term=112724)(80.882)[DHRS3](http://www.ncbi.nlm.nih.gov/gene/?term=9249)(80.882) [RDH5](http://www.ncbi.nlm.nih.gov/gene/?term=5959)(80.882)[ALDH1A3](http://www.ncbi.nlm.nih.gov/gene/?term=220)(80.882)[RDH12](http://www.ncbi.nlm.nih.gov/gene/?term=145226)(80.882)[DHRS4](http://www.ncbi.nlm.nih.gov/gene/?term=10901)(80.882)[RBP1](http://www.ncbi.nlm.nih.gov/gene/?term=5947)(80.882) [ALDH1A1](http://www.ncbi.nlm.nih.gov/gene/?term=216)(80.882)[RLBP1](http://www.ncbi.nlm.nih.gov/gene/?term=6017)(80.882)[RDH14](http://www.ncbi.nlm.nih.gov/gene/?term=57665)(80.882)[RDH8](http://www.ncbi.nlm.nih.gov/gene/?term=50700)(80.882)[LRAT](http://www.ncbi.nlm.nih.gov/gene/?term=9227)(80.882) [ALDH1A2](http://www.ncbi.nlm.nih.gov/gene/?term=8854)(80.882)[ALDH3A1](http://www.ncbi.nlm.nih.gov/gene/?term=218)(55.444)[RHO](http://www.ncbi.nlm.nih.gov/gene/?term=6010)(55.444)[RS1](http://www.ncbi.nlm.nih.gov/gene/?term=6247)(55.444)[F12](http://www.ncbi.nlm.nih.gov/gene/?term=2161)(48.000) [ESR1](http://www.ncbi.nlm.nih.gov/gene/?term=2099)(48.000)[PGR](http://www.ncbi.nlm.nih.gov/gene/?term=5241)(48.000)[CTSH](http://www.ncbi.nlm.nih.gov/gene/?term=1512)(22.373)[OPN4](http://www.ncbi.nlm.nih.gov/gene/?term=94233)(22.373)[RDH10](http://www.ncbi.nlm.nih.gov/gene/?term=157506)(22.373) [ALDH8A1](http://www.ncbi.nlm.nih.gov/gene/?term=64577)(22.373)[SHBG](http://www.ncbi.nlm.nih.gov/gene/?term=6462)(22.373)[DHRS2](http://www.ncbi.nlm.nih.gov/gene/?term=10202)(22.373)[HSD17B7](http://www.ncbi.nlm.nih.gov/gene/?term=51478)(22.373)[AR](http://www.ncbi.nlm.nih.gov/gene/?term=367)(22.373) [THRA](http://www.ncbi.nlm.nih.gov/gene/?term=7067)(22.373)[PAX2](http://www.ncbi.nlm.nih.gov/gene/?term=5076)(22.373)[CBR3](http://www.ncbi.nlm.nih.gov/gene/?term=874)(22.373)[GATA3](http://www.ncbi.nlm.nih.gov/gene/?term=2625)(22.373)[ASCL1](http://www.ncbi.nlm.nih.gov/gene/?term=429)(22.373) [DLX5](http://www.ncbi.nlm.nih.gov/gene/?term=1749)(22.373)[RET](http://www.ncbi.nlm.nih.gov/gene/?term=5979)(22.373)[THRB](http://www.ncbi.nlm.nih.gov/gene/?term=7068)(22.373)[CRYM](http://www.ncbi.nlm.nih.gov/gene/?term=1428)(22.373)[TSPO](http://www.ncbi.nlm.nih.gov/gene/?term=706)(22.373) [OPN5](http://www.ncbi.nlm.nih.gov/gene/?term=221391)(22.373)[DHRS9](http://www.ncbi.nlm.nih.gov/gene/?term=10170)(22.373)[CBR1](http://www.ncbi.nlm.nih.gov/gene/?term=873)(22.373)[CYP1A1](http://www.ncbi.nlm.nih.gov/gene/?term=1543)(22.373) |
| [Alpha-Boswellic Acid](http://www.megabionet.org/tcmid/ingredient/30678/) | [SRD5A1](http://www.ncbi.nlm.nih.gov/gene/?term=6715)(48.000)[CYP17A1](http://www.ncbi.nlm.nih.gov/gene/?term=1586)(48.000)[SLC8A1](http://www.ncbi.nlm.nih.gov/gene/?term=6546)(48.000)[F12](http://www.ncbi.nlm.nih.gov/gene/?term=2161)(48.000)[ESR1](http://www.ncbi.nlm.nih.gov/gene/?term=2099)(48.000) [TRPV1](http://www.ncbi.nlm.nih.gov/gene/?term=7442)(48.000)[PTGER1](http://www.ncbi.nlm.nih.gov/gene/?term=5731)(48.000)[PTGER4](http://www.ncbi.nlm.nih.gov/gene/?term=5734)(48.000)[PGR](http://www.ncbi.nlm.nih.gov/gene/?term=5241)(48.000)[AR](http://www.ncbi.nlm.nih.gov/gene/?term=367)(48.000) [NR3C1](http://www.ncbi.nlm.nih.gov/gene/?term=2908)(48.000)[OPRK1](http://www.ncbi.nlm.nih.gov/gene/?term=4986)(48.000)[FADS2](http://www.ncbi.nlm.nih.gov/gene/?term=9415)(48.000)[FADS1](http://www.ncbi.nlm.nih.gov/gene/?term=3992)(48.000)[PTGER2](http://www.ncbi.nlm.nih.gov/gene/?term=5732)(48.000) [PTGS1](http://www.ncbi.nlm.nih.gov/gene/?term=5742)(48.000)[PTGER3](http://www.ncbi.nlm.nih.gov/gene/?term=5733)(48.000)[PTGS2](http://www.ncbi.nlm.nih.gov/gene/?term=5743)(48.000)[ELOVL4](http://www.ncbi.nlm.nih.gov/gene/?term=6785)(48.000)[ANXA1](http://www.ncbi.nlm.nih.gov/gene/?term=301)(48.000) [NR3C2](http://www.ncbi.nlm.nih.gov/gene/?term=4306)(48.000)[ESRRG](http://www.ncbi.nlm.nih.gov/gene/?term=2104)(23.000)[COX6C](http://www.ncbi.nlm.nih.gov/gene/?term=1345)(23.000)[COX5B](http://www.ncbi.nlm.nih.gov/gene/?term=1329)(23.000)[COX7C](http://www.ncbi.nlm.nih.gov/gene/?term=1350)(23.000) [COX1](http://www.ncbi.nlm.nih.gov/gene/?term=4512)(23.000)[AKR1C2](http://www.ncbi.nlm.nih.gov/gene/?term=1646)(23.000)[ADH1B](http://www.ncbi.nlm.nih.gov/gene/?term=125)(23.000)[COX5A](http://www.ncbi.nlm.nih.gov/gene/?term=9377)(23.000)[COX3](http://www.ncbi.nlm.nih.gov/gene/?term=4514)(23.000) [COX7A1](http://www.ncbi.nlm.nih.gov/gene/?term=1346)(23.000)[FECH](http://www.ncbi.nlm.nih.gov/gene/?term=2235)(23.000)[COX4I1](http://www.ncbi.nlm.nih.gov/gene/?term=1327)(23.000)[PLA2G1B](http://www.ncbi.nlm.nih.gov/gene/?term=5319)(23.000)[AKR1C1](http://www.ncbi.nlm.nih.gov/gene/?term=1645)(23.000) [COX6A2](http://www.ncbi.nlm.nih.gov/gene/?term=1339)(23.000)[HMGCR](http://www.ncbi.nlm.nih.gov/gene/?term=3156)(23.000)[ADH1C](http://www.ncbi.nlm.nih.gov/gene/?term=126)(23.000)[COX6B1](http://www.ncbi.nlm.nih.gov/gene/?term=1340)(23.000)[FABP6](http://www.ncbi.nlm.nih.gov/gene/?term=2172)(23.000) [CES1](http://www.ncbi.nlm.nih.gov/gene/?term=1066)(23.000)[COX7B](http://www.ncbi.nlm.nih.gov/gene/?term=1349)(23.000)[COX2](http://www.ncbi.nlm.nih.gov/gene/?term=4513)(23.000)[COX8A](http://www.ncbi.nlm.nih.gov/gene/?term=1351)(23.000)[NR1H4](http://www.ncbi.nlm.nih.gov/gene/?term=9971)(23.000) |
| [Emodin](http://www.megabionet.org/tcmid/ingredient/6775/) | [ESR1](http://www.ncbi.nlm.nih.gov/gene/?term=2099)(80.882)[HSD17B1](http://www.ncbi.nlm.nih.gov/gene/?term=3292)(23.000)[TFAP2C](http://www.ncbi.nlm.nih.gov/gene/?term=7022)(22.373)[TOX3](http://www.ncbi.nlm.nih.gov/gene/?term=27324)(22.373)[TGFB1](http://www.ncbi.nlm.nih.gov/gene/?term=7040)(22.373) [MED1](http://www.ncbi.nlm.nih.gov/gene/?term=5469)(22.373)[NKX3-1](http://www.ncbi.nlm.nih.gov/gene/?term=4824)(22.373)[ESR2](http://www.ncbi.nlm.nih.gov/gene/?term=2100)(22.373)[SHH](http://www.ncbi.nlm.nih.gov/gene/?term=6469)(22.373)[LEF1](http://www.ncbi.nlm.nih.gov/gene/?term=51176)(22.373) [FGFR2](http://www.ncbi.nlm.nih.gov/gene/?term=2263)(22.373)[PGR](http://www.ncbi.nlm.nih.gov/gene/?term=5241)(22.373)[WNT4](http://www.ncbi.nlm.nih.gov/gene/?term=54361)(22.373)[AREG](http://www.ncbi.nlm.nih.gov/gene/?term=374)(22.373)[GPER1](http://www.ncbi.nlm.nih.gov/gene/?term=2852)(22.373) [WNT5A](http://www.ncbi.nlm.nih.gov/gene/?term=7474)(22.373)[SOX9](http://www.ncbi.nlm.nih.gov/gene/?term=6662)(22.373)[VDR](http://www.ncbi.nlm.nih.gov/gene/?term=7421)(22.373)[TRIM24](http://www.ncbi.nlm.nih.gov/gene/?term=8805)(22.373) |
| [3,6,6-Trimethylnorpinan-2-One](http://www.megabionet.org/tcmid/ingredient/21954/) | [AR](http://www.ncbi.nlm.nih.gov/gene/?term=367)(48.000) |
| [Chrysophanol](http://www.megabionet.org/tcmid/ingredient/3615/) | [CFTR](http://www.ncbi.nlm.nih.gov/gene/?term=1080)(80.882)[TCAF1](http://www.ncbi.nlm.nih.gov/gene/?term=9747)(55.444)[KCNE2](http://www.ncbi.nlm.nih.gov/gene/?term=9992)(55.444)[HSD17B1](http://www.ncbi.nlm.nih.gov/gene/?term=3292)(23.000)[NOX1](http://www.ncbi.nlm.nih.gov/gene/?term=27035)(22.373) [CA7](http://www.ncbi.nlm.nih.gov/gene/?term=766)(22.373)[DNAJC3](http://www.ncbi.nlm.nih.gov/gene/?term=5611)(22.373)[ANO9](http://www.ncbi.nlm.nih.gov/gene/?term=338440)(22.373)[SLC26A6](http://www.ncbi.nlm.nih.gov/gene/?term=65010)(22.373)[FXYD1](http://www.ncbi.nlm.nih.gov/gene/?term=5348)(22.373) [CLN3](http://www.ncbi.nlm.nih.gov/gene/?term=1201)(22.373)[TCAF2](http://www.ncbi.nlm.nih.gov/gene/?term=285966)(22.373)[SLC26A3](http://www.ncbi.nlm.nih.gov/gene/?term=1811)(22.373)[SLC34A1](http://www.ncbi.nlm.nih.gov/gene/?term=6569)(22.373)[CA2](http://www.ncbi.nlm.nih.gov/gene/?term=760)(22.373) [SEC61B](http://www.ncbi.nlm.nih.gov/gene/?term=10952)(22.373) |
| [O-Cresol](http://www.megabionet.org/tcmid/ingredient/4232/) | [GABRA2](http://www.ncbi.nlm.nih.gov/gene/?term=2555)(80.882)[GABRD](http://www.ncbi.nlm.nih.gov/gene/?term=2563)(80.882)[PDE3A](http://www.ncbi.nlm.nih.gov/gene/?term=5139)(80.882)[GABRB1](http://www.ncbi.nlm.nih.gov/gene/?term=2560)(80.882)[GABRG3](http://www.ncbi.nlm.nih.gov/gene/?term=2567)(80.882) [TNF](http://www.ncbi.nlm.nih.gov/gene/?term=7124)(80.882)[GABRE](http://www.ncbi.nlm.nih.gov/gene/?term=2564)(80.882)[SCN2A](http://www.ncbi.nlm.nih.gov/gene/?term=6326)(80.882)[GABRA3](http://www.ncbi.nlm.nih.gov/gene/?term=2556)(80.882)[GABRG1](http://www.ncbi.nlm.nih.gov/gene/?term=2565)(80.882) [GABRP](http://www.ncbi.nlm.nih.gov/gene/?term=2568)(80.882)[GABRA4](http://www.ncbi.nlm.nih.gov/gene/?term=2557)(80.882)[GABRB2](http://www.ncbi.nlm.nih.gov/gene/?term=2561)(80.882)[ACHE](http://www.ncbi.nlm.nih.gov/gene/?term=43)(80.882)[SCN4A](http://www.ncbi.nlm.nih.gov/gene/?term=6329)(80.882) [GABRQ](http://www.ncbi.nlm.nih.gov/gene/?term=55879)(80.882)[GABRB3](http://www.ncbi.nlm.nih.gov/gene/?term=2562)(80.882)[GABRA5](http://www.ncbi.nlm.nih.gov/gene/?term=2558)(80.882)[GABRA6](http://www.ncbi.nlm.nih.gov/gene/?term=2559)(80.882)[GABRA1](http://www.ncbi.nlm.nih.gov/gene/?term=2554)(80.882) [PDE4B](http://www.ncbi.nlm.nih.gov/gene/?term=5142)(80.882)[BCHE](http://www.ncbi.nlm.nih.gov/gene/?term=590)(80.882)[GABRG2](http://www.ncbi.nlm.nih.gov/gene/?term=2566)(80.882)[PDE3B](http://www.ncbi.nlm.nih.gov/gene/?term=5140)(55.444)[PDE4D](http://www.ncbi.nlm.nih.gov/gene/?term=5144)(55.444) [COLQ](http://www.ncbi.nlm.nih.gov/gene/?term=8292)(55.444)[LTA](http://www.ncbi.nlm.nih.gov/gene/?term=4049)(55.444)[RXRA](http://www.ncbi.nlm.nih.gov/gene/?term=6256)(55.444)[SCN1A](http://www.ncbi.nlm.nih.gov/gene/?term=6323)(23.000)[SCN3A](http://www.ncbi.nlm.nih.gov/gene/?term=6328)(23.000) [HMGA2](http://www.ncbi.nlm.nih.gov/gene/?term=8091)(22.373)[PDE5A](http://www.ncbi.nlm.nih.gov/gene/?term=8654)(22.373)[APOA2](http://www.ncbi.nlm.nih.gov/gene/?term=336)(22.373)[SLC5A7](http://www.ncbi.nlm.nih.gov/gene/?term=60482)(22.373)[SEMA4D](http://www.ncbi.nlm.nih.gov/gene/?term=10507)(22.373) [NRG1](http://www.ncbi.nlm.nih.gov/gene/?term=3084)(22.373)[CD74](http://www.ncbi.nlm.nih.gov/gene/?term=972)(22.373)[HPX](http://www.ncbi.nlm.nih.gov/gene/?term=3263)(22.373)[SIRT2](http://www.ncbi.nlm.nih.gov/gene/?term=22933)(22.373)[MIF](http://www.ncbi.nlm.nih.gov/gene/?term=4282)(22.373) [DMGDH](http://www.ncbi.nlm.nih.gov/gene/?term=29958)(22.373)[NKX3-1](http://www.ncbi.nlm.nih.gov/gene/?term=4824)(22.373)[SIX3](http://www.ncbi.nlm.nih.gov/gene/?term=6496)(22.373)[HCN4](http://www.ncbi.nlm.nih.gov/gene/?term=10021)(22.373)[FASLG](http://www.ncbi.nlm.nih.gov/gene/?term=356)(22.373) [LGALS3](http://www.ncbi.nlm.nih.gov/gene/?term=3958)(22.373)[KLF4](http://www.ncbi.nlm.nih.gov/gene/?term=9314)(22.373)[LILRB1](http://www.ncbi.nlm.nih.gov/gene/?term=10859)(22.373)[NCKAP1L](http://www.ncbi.nlm.nih.gov/gene/?term=3071)(22.373)[CRP](http://www.ncbi.nlm.nih.gov/gene/?term=1401)(22.373) [TLR3](http://www.ncbi.nlm.nih.gov/gene/?term=7098)(22.373)[EPO](http://www.ncbi.nlm.nih.gov/gene/?term=2056)(22.373)[MAP2K5](http://www.ncbi.nlm.nih.gov/gene/?term=5607)(22.373)[EIF2AK1](http://www.ncbi.nlm.nih.gov/gene/?term=27102)(22.373)[IL1B](http://www.ncbi.nlm.nih.gov/gene/?term=3553)(22.373) [SHH](http://www.ncbi.nlm.nih.gov/gene/?term=6469)(22.373)[RIPK1](http://www.ncbi.nlm.nih.gov/gene/?term=8737)(22.373)[FGF10](http://www.ncbi.nlm.nih.gov/gene/?term=2255)(22.373)[PRSS12](http://www.ncbi.nlm.nih.gov/gene/?term=8492)(22.373)[SLC44A4](http://www.ncbi.nlm.nih.gov/gene/?term=80736)(22.373) [ADORA2B](http://www.ncbi.nlm.nih.gov/gene/?term=136)(22.373)[ALDH7A1](http://www.ncbi.nlm.nih.gov/gene/?term=501)(22.373)[FADD](http://www.ncbi.nlm.nih.gov/gene/?term=8772)(22.373)[FGFR2](http://www.ncbi.nlm.nih.gov/gene/?term=2263)(22.373)[ANGPT1](http://www.ncbi.nlm.nih.gov/gene/?term=284)(22.373) [AURKA](http://www.ncbi.nlm.nih.gov/gene/?term=6790)(22.373)[WNT2](http://www.ncbi.nlm.nih.gov/gene/?term=7472)(22.373)[YBX3](http://www.ncbi.nlm.nih.gov/gene/?term=8531)(22.373)[IL1RN](http://www.ncbi.nlm.nih.gov/gene/?term=3557)(22.373)[PLN](http://www.ncbi.nlm.nih.gov/gene/?term=5350)(22.373) [DNM3](http://www.ncbi.nlm.nih.gov/gene/?term=26052)(22.373)[FCER2](http://www.ncbi.nlm.nih.gov/gene/?term=2208)(22.373)[WNT2B](http://www.ncbi.nlm.nih.gov/gene/?term=7482)(22.373)[IFNG](http://www.ncbi.nlm.nih.gov/gene/?term=3458)(22.373)[HRC](http://www.ncbi.nlm.nih.gov/gene/?term=3270)(22.373) [SLC6A4](http://www.ncbi.nlm.nih.gov/gene/?term=6532)(22.373)[TGFB2](http://www.ncbi.nlm.nih.gov/gene/?term=7042)(22.373)[RAPGEF2](http://www.ncbi.nlm.nih.gov/gene/?term=9693)(22.373)[TIRAP](http://www.ncbi.nlm.nih.gov/gene/?term=114609)(22.373)[ROCK2](http://www.ncbi.nlm.nih.gov/gene/?term=9475)(22.373) [ADIRF](http://www.ncbi.nlm.nih.gov/gene/?term=10974)(22.373)[BDKRB2](http://www.ncbi.nlm.nih.gov/gene/?term=624)(22.373)[ASCL1](http://www.ncbi.nlm.nih.gov/gene/?term=429)(22.373)[ENPP6](http://www.ncbi.nlm.nih.gov/gene/?term=133121)(22.373)[CAMK2D](http://www.ncbi.nlm.nih.gov/gene/?term=817)(22.373) [IL10](http://www.ncbi.nlm.nih.gov/gene/?term=3586)(22.373)[CDH8](http://www.ncbi.nlm.nih.gov/gene/?term=1006)(22.373)[NCOA1](http://www.ncbi.nlm.nih.gov/gene/?term=8648)(22.373)[IL6](http://www.ncbi.nlm.nih.gov/gene/?term=3569)(22.373)[ROCK1](http://www.ncbi.nlm.nih.gov/gene/?term=6093)(22.373) [FNTA](http://www.ncbi.nlm.nih.gov/gene/?term=2339)(22.373)[HPN](http://www.ncbi.nlm.nih.gov/gene/?term=3249)(22.373)[KMT2A](http://www.ncbi.nlm.nih.gov/gene/?term=4297)(22.373)[NOD2](http://www.ncbi.nlm.nih.gov/gene/?term=64127)(22.373)[BMP4](http://www.ncbi.nlm.nih.gov/gene/?term=652)(22.373) [AHCY](http://www.ncbi.nlm.nih.gov/gene/?term=191)(22.373)[APOA1](http://www.ncbi.nlm.nih.gov/gene/?term=335)(22.373)[ARHGEF2](http://www.ncbi.nlm.nih.gov/gene/?term=9181)(22.373)[IL4](http://www.ncbi.nlm.nih.gov/gene/?term=3565)(22.373)[AGRN](http://www.ncbi.nlm.nih.gov/gene/?term=375790)(22.373) [IKBKB](http://www.ncbi.nlm.nih.gov/gene/?term=3551)(22.373)[IDO1](http://www.ncbi.nlm.nih.gov/gene/?term=3620)(22.373)[CTNNB1](http://www.ncbi.nlm.nih.gov/gene/?term=1499)(22.373)[CHKA](http://www.ncbi.nlm.nih.gov/gene/?term=1119)(22.373)[PDE2A](http://www.ncbi.nlm.nih.gov/gene/?term=5138)(22.373) [CHDH](http://www.ncbi.nlm.nih.gov/gene/?term=55349)(22.373)[SOX9](http://www.ncbi.nlm.nih.gov/gene/?term=6662)(22.373)[CAMK2G](http://www.ncbi.nlm.nih.gov/gene/?term=818)(22.373)[FAS](http://www.ncbi.nlm.nih.gov/gene/?term=355)(22.373)[GRIN1](http://www.ncbi.nlm.nih.gov/gene/?term=2902)(22.373) [HCN2](http://www.ncbi.nlm.nih.gov/gene/?term=610)(22.373) |
| [Berberine](http://www.megabionet.org/tcmid/ingredient/2303/) | This compound doesn't have any potential target with score larger than 20. |
| [Eugenol](http://www.megabionet.org/tcmid/ingredient/7520/) | [TRPV3](http://www.ncbi.nlm.nih.gov/gene/?term=162514)(114.143)[OPRK1](http://www.ncbi.nlm.nih.gov/gene/?term=4986)(114.143)[TRPM8](http://www.ncbi.nlm.nih.gov/gene/?term=79054)(114.143)[TRPA1](http://www.ncbi.nlm.nih.gov/gene/?term=8989)(114.143)[CNR2](http://www.ncbi.nlm.nih.gov/gene/?term=1269)(23.000) [TYR](http://www.ncbi.nlm.nih.gov/gene/?term=7299)(23.000)[CNR1](http://www.ncbi.nlm.nih.gov/gene/?term=1268)(23.000) |
| [Columbianadin](http://www.megabionet.org/tcmid/ingredient/3935/) | This compound doesn't have any potential target with score larger than 20. |
| [Ruine](http://www.megabionet.org/tcmid/ingredient/19047/) | This compound doesn't have any potential target with score larger than 20. |
| [Chavicol](http://www.megabionet.org/tcmid/ingredient/3488/) | [ADRA1A](http://www.ncbi.nlm.nih.gov/gene/?term=148)(80.882)[ESRRG](http://www.ncbi.nlm.nih.gov/gene/?term=2104)(80.882)[ESR2](http://www.ncbi.nlm.nih.gov/gene/?term=2100)(80.882)[ADRA2A](http://www.ncbi.nlm.nih.gov/gene/?term=150)(80.882)[ESR1](http://www.ncbi.nlm.nih.gov/gene/?term=2099)(80.882) [ADRA1D](http://www.ncbi.nlm.nih.gov/gene/?term=146)(80.882)[ADRA1B](http://www.ncbi.nlm.nih.gov/gene/?term=147)(80.882)[ADRA2C](http://www.ncbi.nlm.nih.gov/gene/?term=152)(80.882)[ADRA2B](http://www.ncbi.nlm.nih.gov/gene/?term=151)(80.882)[PHKG2](http://www.ncbi.nlm.nih.gov/gene/?term=5261)(55.444) [APLP1](http://www.ncbi.nlm.nih.gov/gene/?term=333)(55.444)[CHRNA7](http://www.ncbi.nlm.nih.gov/gene/?term=1139)(55.444)[INS](http://www.ncbi.nlm.nih.gov/gene/?term=3630)(55.444)[CALCA](http://www.ncbi.nlm.nih.gov/gene/?term=796)(55.444)[GABRA2](http://www.ncbi.nlm.nih.gov/gene/?term=2555)(48.000) [GABRD](http://www.ncbi.nlm.nih.gov/gene/?term=2563)(48.000)[GABRB1](http://www.ncbi.nlm.nih.gov/gene/?term=2560)(48.000)[GABRG3](http://www.ncbi.nlm.nih.gov/gene/?term=2567)(48.000)[GABRE](http://www.ncbi.nlm.nih.gov/gene/?term=2564)(48.000)[SCN2A](http://www.ncbi.nlm.nih.gov/gene/?term=6326)(48.000) [GABRA3](http://www.ncbi.nlm.nih.gov/gene/?term=2556)(48.000)[GABRG1](http://www.ncbi.nlm.nih.gov/gene/?term=2565)(48.000)[GABRP](http://www.ncbi.nlm.nih.gov/gene/?term=2568)(48.000)[GABRA4](http://www.ncbi.nlm.nih.gov/gene/?term=2557)(48.000)[GABRB2](http://www.ncbi.nlm.nih.gov/gene/?term=2561)(48.000) [SCN4A](http://www.ncbi.nlm.nih.gov/gene/?term=6329)(48.000)[GABRQ](http://www.ncbi.nlm.nih.gov/gene/?term=55879)(48.000)[GABRB3](http://www.ncbi.nlm.nih.gov/gene/?term=2562)(48.000)[GABRA5](http://www.ncbi.nlm.nih.gov/gene/?term=2558)(48.000)[GABRA6](http://www.ncbi.nlm.nih.gov/gene/?term=2559)(48.000) [GABRA1](http://www.ncbi.nlm.nih.gov/gene/?term=2554)(48.000)[GABRG2](http://www.ncbi.nlm.nih.gov/gene/?term=2566)(48.000)[TFAP2C](http://www.ncbi.nlm.nih.gov/gene/?term=7022)(22.373)[TOX3](http://www.ncbi.nlm.nih.gov/gene/?term=27324)(22.373)[NAV2](http://www.ncbi.nlm.nih.gov/gene/?term=89797)(22.373) [TGFB1](http://www.ncbi.nlm.nih.gov/gene/?term=7040)(22.373)[ADRB2](http://www.ncbi.nlm.nih.gov/gene/?term=154)(22.373)[MED1](http://www.ncbi.nlm.nih.gov/gene/?term=5469)(22.373)[GSK3A](http://www.ncbi.nlm.nih.gov/gene/?term=2931)(22.373)[NKX3-1](http://www.ncbi.nlm.nih.gov/gene/?term=4824)(22.373) [CETN2](http://www.ncbi.nlm.nih.gov/gene/?term=1069)(22.373)[NR0B1](http://www.ncbi.nlm.nih.gov/gene/?term=190)(22.373)[CETN1](http://www.ncbi.nlm.nih.gov/gene/?term=1068)(22.373)[TRPV1](http://www.ncbi.nlm.nih.gov/gene/?term=7442)(22.373)[SHH](http://www.ncbi.nlm.nih.gov/gene/?term=6469)(22.373) [LEF1](http://www.ncbi.nlm.nih.gov/gene/?term=51176)(22.373)[ADRBK1](http://www.ncbi.nlm.nih.gov/gene/?term=156)(22.373)[ARRB2](http://www.ncbi.nlm.nih.gov/gene/?term=409)(22.373)[ADRB1](http://www.ncbi.nlm.nih.gov/gene/?term=153)(22.373)[UCHL1](http://www.ncbi.nlm.nih.gov/gene/?term=7345)(22.373) [PDE4D](http://www.ncbi.nlm.nih.gov/gene/?term=5144)(22.373)[FGFR2](http://www.ncbi.nlm.nih.gov/gene/?term=2263)(22.373)[PGR](http://www.ncbi.nlm.nih.gov/gene/?term=5241)(22.373)[PPARGC1B](http://www.ncbi.nlm.nih.gov/gene/?term=133522)(22.373)[WNT4](http://www.ncbi.nlm.nih.gov/gene/?term=54361)(22.373) [ARRB1](http://www.ncbi.nlm.nih.gov/gene/?term=408)(22.373)[NCOA3](http://www.ncbi.nlm.nih.gov/gene/?term=8202)(22.373)[AREG](http://www.ncbi.nlm.nih.gov/gene/?term=374)(22.373)[DRD5](http://www.ncbi.nlm.nih.gov/gene/?term=1816)(22.373)[ATP2B4](http://www.ncbi.nlm.nih.gov/gene/?term=493)(22.373) [ARRDC3](http://www.ncbi.nlm.nih.gov/gene/?term=57561)(22.373)[GPER1](http://www.ncbi.nlm.nih.gov/gene/?term=2852)(22.373)[WNT5A](http://www.ncbi.nlm.nih.gov/gene/?term=7474)(22.373)[ADORA1](http://www.ncbi.nlm.nih.gov/gene/?term=134)(22.373)[ADAM17](http://www.ncbi.nlm.nih.gov/gene/?term=6868)(22.373) [ADRB3](http://www.ncbi.nlm.nih.gov/gene/?term=155)(22.373)[NOS1](http://www.ncbi.nlm.nih.gov/gene/?term=4842)(22.373)[RGS2](http://www.ncbi.nlm.nih.gov/gene/?term=5997)(22.373)[TRPM8](http://www.ncbi.nlm.nih.gov/gene/?term=79054)(22.373)[SOX9](http://www.ncbi.nlm.nih.gov/gene/?term=6662)(22.373) [VDR](http://www.ncbi.nlm.nih.gov/gene/?term=7421)(22.373)[TPM1](http://www.ncbi.nlm.nih.gov/gene/?term=7168)(22.373)[EDN2](http://www.ncbi.nlm.nih.gov/gene/?term=1907)(22.373)[TRIM24](http://www.ncbi.nlm.nih.gov/gene/?term=8805)(22.373)[TRPA1](http://www.ncbi.nlm.nih.gov/gene/?term=8989)(22.373) |
| [2-Methyl Cardol](http://www.megabionet.org/tcmid/ingredient/25514/) | [ADRA1A](http://www.ncbi.nlm.nih.gov/gene/?term=148)(80.882)[ESRRG](http://www.ncbi.nlm.nih.gov/gene/?term=2104)(80.882)[ESR2](http://www.ncbi.nlm.nih.gov/gene/?term=2100)(80.882)[ADRA2A](http://www.ncbi.nlm.nih.gov/gene/?term=150)(80.882)[ESR1](http://www.ncbi.nlm.nih.gov/gene/?term=2099)(80.882) [ADRA1D](http://www.ncbi.nlm.nih.gov/gene/?term=146)(80.882)[ADRA1B](http://www.ncbi.nlm.nih.gov/gene/?term=147)(80.882)[ADRA2C](http://www.ncbi.nlm.nih.gov/gene/?term=152)(80.882)[ADRA2B](http://www.ncbi.nlm.nih.gov/gene/?term=151)(80.882)[PHKG2](http://www.ncbi.nlm.nih.gov/gene/?term=5261)(55.444) [APLP1](http://www.ncbi.nlm.nih.gov/gene/?term=333)(55.444)[CHRNA7](http://www.ncbi.nlm.nih.gov/gene/?term=1139)(55.444)[INS](http://www.ncbi.nlm.nih.gov/gene/?term=3630)(55.444)[CALCA](http://www.ncbi.nlm.nih.gov/gene/?term=796)(55.444)[TFAP2C](http://www.ncbi.nlm.nih.gov/gene/?term=7022)(22.373) [TOX3](http://www.ncbi.nlm.nih.gov/gene/?term=27324)(22.373)[NAV2](http://www.ncbi.nlm.nih.gov/gene/?term=89797)(22.373)[TGFB1](http://www.ncbi.nlm.nih.gov/gene/?term=7040)(22.373)[ADRB2](http://www.ncbi.nlm.nih.gov/gene/?term=154)(22.373)[MED1](http://www.ncbi.nlm.nih.gov/gene/?term=5469)(22.373) [GSK3A](http://www.ncbi.nlm.nih.gov/gene/?term=2931)(22.373)[NKX3-1](http://www.ncbi.nlm.nih.gov/gene/?term=4824)(22.373)[CETN2](http://www.ncbi.nlm.nih.gov/gene/?term=1069)(22.373)[NR0B1](http://www.ncbi.nlm.nih.gov/gene/?term=190)(22.373)[CETN1](http://www.ncbi.nlm.nih.gov/gene/?term=1068)(22.373) [TRPV1](http://www.ncbi.nlm.nih.gov/gene/?term=7442)(22.373)[SHH](http://www.ncbi.nlm.nih.gov/gene/?term=6469)(22.373)[LEF1](http://www.ncbi.nlm.nih.gov/gene/?term=51176)(22.373)[ADRBK1](http://www.ncbi.nlm.nih.gov/gene/?term=156)(22.373)[ARRB2](http://www.ncbi.nlm.nih.gov/gene/?term=409)(22.373) [ADRB1](http://www.ncbi.nlm.nih.gov/gene/?term=153)(22.373)[UCHL1](http://www.ncbi.nlm.nih.gov/gene/?term=7345)(22.373)[PDE4D](http://www.ncbi.nlm.nih.gov/gene/?term=5144)(22.373)[FGFR2](http://www.ncbi.nlm.nih.gov/gene/?term=2263)(22.373)[PGR](http://www.ncbi.nlm.nih.gov/gene/?term=5241)(22.373) [PPARGC1B](http://www.ncbi.nlm.nih.gov/gene/?term=133522)(22.373)[WNT4](http://www.ncbi.nlm.nih.gov/gene/?term=54361)(22.373)[ARRB1](http://www.ncbi.nlm.nih.gov/gene/?term=408)(22.373)[NCOA3](http://www.ncbi.nlm.nih.gov/gene/?term=8202)(22.373)[AREG](http://www.ncbi.nlm.nih.gov/gene/?term=374)(22.373) [DRD5](http://www.ncbi.nlm.nih.gov/gene/?term=1816)(22.373)[ATP2B4](http://www.ncbi.nlm.nih.gov/gene/?term=493)(22.373)[ARRDC3](http://www.ncbi.nlm.nih.gov/gene/?term=57561)(22.373)[GPER1](http://www.ncbi.nlm.nih.gov/gene/?term=2852)(22.373)[WNT5A](http://www.ncbi.nlm.nih.gov/gene/?term=7474)(22.373) [ADORA1](http://www.ncbi.nlm.nih.gov/gene/?term=134)(22.373)[ADAM17](http://www.ncbi.nlm.nih.gov/gene/?term=6868)(22.373)[ADRB3](http://www.ncbi.nlm.nih.gov/gene/?term=155)(22.373)[NOS1](http://www.ncbi.nlm.nih.gov/gene/?term=4842)(22.373)[RGS2](http://www.ncbi.nlm.nih.gov/gene/?term=5997)(22.373) [TRPM8](http://www.ncbi.nlm.nih.gov/gene/?term=79054)(22.373)[SOX9](http://www.ncbi.nlm.nih.gov/gene/?term=6662)(22.373)[VDR](http://www.ncbi.nlm.nih.gov/gene/?term=7421)(22.373)[TPM1](http://www.ncbi.nlm.nih.gov/gene/?term=7168)(22.373)[EDN2](http://www.ncbi.nlm.nih.gov/gene/?term=1907)(22.373) [TRIM24](http://www.ncbi.nlm.nih.gov/gene/?term=8805)(22.373)[TRPA1](http://www.ncbi.nlm.nih.gov/gene/?term=8989)(22.373) |
| [Alpha-Limonene](http://www.megabionet.org/tcmid/ingredient/31444/) | [KCND1](http://www.ncbi.nlm.nih.gov/gene/?term=3750)(80.882)[KCNA3](http://www.ncbi.nlm.nih.gov/gene/?term=3738)(80.882)[PRKAB1](http://www.ncbi.nlm.nih.gov/gene/?term=5564)(80.882)[ADH1A](http://www.ncbi.nlm.nih.gov/gene/?term=124)(80.882)[KCNA10](http://www.ncbi.nlm.nih.gov/gene/?term=3744)(80.882) [GAMT](http://www.ncbi.nlm.nih.gov/gene/?term=2593)(80.882)[KCNC3](http://www.ncbi.nlm.nih.gov/gene/?term=3748)(80.882)[KCNA1](http://www.ncbi.nlm.nih.gov/gene/?term=3736)(80.882)[KCNA2](http://www.ncbi.nlm.nih.gov/gene/?term=3737)(80.882)[TPO](http://www.ncbi.nlm.nih.gov/gene/?term=7173)(80.882) [CAT](http://www.ncbi.nlm.nih.gov/gene/?term=847)(80.882)[KCNB1](http://www.ncbi.nlm.nih.gov/gene/?term=3745)(80.882)[ADH1B](http://www.ncbi.nlm.nih.gov/gene/?term=125)(80.882)[DLG4](http://www.ncbi.nlm.nih.gov/gene/?term=1742)(80.882)[KCNC2](http://www.ncbi.nlm.nih.gov/gene/?term=3747)(80.882) [KCNC1](http://www.ncbi.nlm.nih.gov/gene/?term=3746)(80.882)[ADH1C](http://www.ncbi.nlm.nih.gov/gene/?term=126)(80.882)[KCNB2](http://www.ncbi.nlm.nih.gov/gene/?term=9312)(80.882)[KCNA5](http://www.ncbi.nlm.nih.gov/gene/?term=3741)(80.882)[RNASE1](http://www.ncbi.nlm.nih.gov/gene/?term=6035)(80.882) [KCND2](http://www.ncbi.nlm.nih.gov/gene/?term=3751)(80.882)[ALDH2](http://www.ncbi.nlm.nih.gov/gene/?term=217)(80.882)[KCNA4](http://www.ncbi.nlm.nih.gov/gene/?term=3739)(80.882)[KCNA7](http://www.ncbi.nlm.nih.gov/gene/?term=3743)(80.882)[KCNA6](http://www.ncbi.nlm.nih.gov/gene/?term=3742)(80.882) [KCND3](http://www.ncbi.nlm.nih.gov/gene/?term=3752)(80.882)[GUCY1B3](http://www.ncbi.nlm.nih.gov/gene/?term=2983)(55.444)[GATM](http://www.ncbi.nlm.nih.gov/gene/?term=2628)(55.444)[KCNK4](http://www.ncbi.nlm.nih.gov/gene/?term=50801)(55.444)[IYD](http://www.ncbi.nlm.nih.gov/gene/?term=389434)(55.444) [KCNQ1](http://www.ncbi.nlm.nih.gov/gene/?term=3784)(55.444)[CYP17A1](http://www.ncbi.nlm.nih.gov/gene/?term=1586)(48.000)[ESR1](http://www.ncbi.nlm.nih.gov/gene/?term=2099)(48.000)[PGR](http://www.ncbi.nlm.nih.gov/gene/?term=5241)(48.000)[OPRK1](http://www.ncbi.nlm.nih.gov/gene/?term=4986)(48.000) [NR3C2](http://www.ncbi.nlm.nih.gov/gene/?term=4306)(48.000)[FBP1](http://www.ncbi.nlm.nih.gov/gene/?term=2203)(22.373)[DGKI](http://www.ncbi.nlm.nih.gov/gene/?term=9162)(22.373)[ADH7](http://www.ncbi.nlm.nih.gov/gene/?term=131)(22.373)[NFIB](http://www.ncbi.nlm.nih.gov/gene/?term=4781)(22.373) [CYGB](http://www.ncbi.nlm.nih.gov/gene/?term=114757)(22.373)[NRXN3](http://www.ncbi.nlm.nih.gov/gene/?term=9369)(22.373)[LRRC4B](http://www.ncbi.nlm.nih.gov/gene/?term=94030)(22.373)[AQP8](http://www.ncbi.nlm.nih.gov/gene/?term=343)(22.373)[ARX](http://www.ncbi.nlm.nih.gov/gene/?term=170302)(22.373) [IGF1](http://www.ncbi.nlm.nih.gov/gene/?term=3479)(22.373)[SHANK3](http://www.ncbi.nlm.nih.gov/gene/?term=85358)(22.373)[ACY3](http://www.ncbi.nlm.nih.gov/gene/?term=91703)(22.373)[ASPA](http://www.ncbi.nlm.nih.gov/gene/?term=443)(22.373)[NRXN1](http://www.ncbi.nlm.nih.gov/gene/?term=9378)(22.373) [EDN1](http://www.ncbi.nlm.nih.gov/gene/?term=1906)(22.373)[OXT](http://www.ncbi.nlm.nih.gov/gene/?term=5020)(22.373)[MAGI2](http://www.ncbi.nlm.nih.gov/gene/?term=9863)(22.373)[APOE](http://www.ncbi.nlm.nih.gov/gene/?term=348)(22.373)[SOX15](http://www.ncbi.nlm.nih.gov/gene/?term=6665)(22.373) [CNTNAP4](http://www.ncbi.nlm.nih.gov/gene/?term=85445)(22.373)[RNASE4](http://www.ncbi.nlm.nih.gov/gene/?term=6038)(22.373)[RAB3A](http://www.ncbi.nlm.nih.gov/gene/?term=5864)(22.373)[MC4R](http://www.ncbi.nlm.nih.gov/gene/?term=4160)(22.373)[IL1B](http://www.ncbi.nlm.nih.gov/gene/?term=3553)(22.373) [GRIN2A](http://www.ncbi.nlm.nih.gov/gene/?term=2903)(22.373)[SLC17A7](http://www.ncbi.nlm.nih.gov/gene/?term=57030)(22.373)[CYP11A1](http://www.ncbi.nlm.nih.gov/gene/?term=1583)(22.373)[SCN5A](http://www.ncbi.nlm.nih.gov/gene/?term=6331)(22.373)[SCN10A](http://www.ncbi.nlm.nih.gov/gene/?term=6336)(22.373) [SPARC](http://www.ncbi.nlm.nih.gov/gene/?term=6678)(22.373)[SORCS3](http://www.ncbi.nlm.nih.gov/gene/?term=22986)(22.373)[RNASE8](http://www.ncbi.nlm.nih.gov/gene/?term=122665)(22.373)[KCNIP2](http://www.ncbi.nlm.nih.gov/gene/?term=30819)(22.373)[GPX7](http://www.ncbi.nlm.nih.gov/gene/?term=2882)(22.373) [CRLF1](http://www.ncbi.nlm.nih.gov/gene/?term=9244)(22.373)[FADD](http://www.ncbi.nlm.nih.gov/gene/?term=8772)(22.373)[SNTG2](http://www.ncbi.nlm.nih.gov/gene/?term=54221)(22.373)[SCGB1A1](http://www.ncbi.nlm.nih.gov/gene/?term=7356)(22.373)[OXTR](http://www.ncbi.nlm.nih.gov/gene/?term=5021)(22.373) [ALDH1B1](http://www.ncbi.nlm.nih.gov/gene/?term=219)(22.373)[LEP](http://www.ncbi.nlm.nih.gov/gene/?term=3952)(22.373)[NLGN1](http://www.ncbi.nlm.nih.gov/gene/?term=22871)(22.373)[CACNA1D](http://www.ncbi.nlm.nih.gov/gene/?term=776)(22.373)[RAPGEF2](http://www.ncbi.nlm.nih.gov/gene/?term=9693)(22.373) [DARS](http://www.ncbi.nlm.nih.gov/gene/?term=1615)(22.373)[ANK3](http://www.ncbi.nlm.nih.gov/gene/?term=288)(22.373)[KCNE5](http://www.ncbi.nlm.nih.gov/gene/?term=23630)(22.373)[PPARD](http://www.ncbi.nlm.nih.gov/gene/?term=5467)(22.373)[NKX2-1](http://www.ncbi.nlm.nih.gov/gene/?term=7080)(22.373) [MIP](http://www.ncbi.nlm.nih.gov/gene/?term=4284)(22.373)[RYR3](http://www.ncbi.nlm.nih.gov/gene/?term=6263)(22.373)[ZPR1](http://www.ncbi.nlm.nih.gov/gene/?term=8882)(22.373)[ALDH3B1](http://www.ncbi.nlm.nih.gov/gene/?term=221)(22.373)[NPPA](http://www.ncbi.nlm.nih.gov/gene/?term=4878)(22.373) [STX1A](http://www.ncbi.nlm.nih.gov/gene/?term=6804)(22.373)[PAXBP1](http://www.ncbi.nlm.nih.gov/gene/?term=94104)(22.373)[PAX7](http://www.ncbi.nlm.nih.gov/gene/?term=5081)(22.373)[ALDH3B2](http://www.ncbi.nlm.nih.gov/gene/?term=222)(22.373)[RNASE2](http://www.ncbi.nlm.nih.gov/gene/?term=6036)(22.373) [NRXN2](http://www.ncbi.nlm.nih.gov/gene/?term=9379)(22.373)[ADORA1](http://www.ncbi.nlm.nih.gov/gene/?term=134)(22.373)[ADH4](http://www.ncbi.nlm.nih.gov/gene/?term=127)(22.373)[GJA5](http://www.ncbi.nlm.nih.gov/gene/?term=2702)(22.373)[FAS](http://www.ncbi.nlm.nih.gov/gene/?term=355)(22.373) [DAB2IP](http://www.ncbi.nlm.nih.gov/gene/?term=153090)(22.373)[CYP2E1](http://www.ncbi.nlm.nih.gov/gene/?term=1571)(22.373)[ACY1](http://www.ncbi.nlm.nih.gov/gene/?term=95)(22.373) |
| [Norjuzunal](http://www.megabionet.org/tcmid/ingredient/15756/) | [ESR1](http://www.ncbi.nlm.nih.gov/gene/?term=2099)(23.000) |
| [Acetyleugenol](http://www.megabionet.org/tcmid/ingredient/391/) | [ADRA1A](http://www.ncbi.nlm.nih.gov/gene/?term=148)(48.000)[ADRB2](http://www.ncbi.nlm.nih.gov/gene/?term=154)(48.000)[ADRA2A](http://www.ncbi.nlm.nih.gov/gene/?term=150)(48.000)[ACHE](http://www.ncbi.nlm.nih.gov/gene/?term=43)(48.000)[BCHE](http://www.ncbi.nlm.nih.gov/gene/?term=590)(48.000) |
| [Aloe-Emodin](http://www.megabionet.org/tcmid/ingredient/23304/) | [CHRM3](http://www.ncbi.nlm.nih.gov/gene/?term=1131)(23.000)[CHRM1](http://www.ncbi.nlm.nih.gov/gene/?term=1128)(23.000)[CHRM2](http://www.ncbi.nlm.nih.gov/gene/?term=1129)(23.000)[CHRM4](http://www.ncbi.nlm.nih.gov/gene/?term=1132)(23.000) |
| [Norisocorydine](http://www.megabionet.org/tcmid/ingredient/15754/) | [HTR2A](http://www.ncbi.nlm.nih.gov/gene/?term=3356)(122.778)[HTR2B](http://www.ncbi.nlm.nih.gov/gene/?term=3357)(122.778)[ADRA2A](http://www.ncbi.nlm.nih.gov/gene/?term=150)(122.778)[HTR1B](http://www.ncbi.nlm.nih.gov/gene/?term=3351)(122.778)[HTR2C](http://www.ncbi.nlm.nih.gov/gene/?term=3358)(122.778) [HTR1D](http://www.ncbi.nlm.nih.gov/gene/?term=3352)(122.778)[DRD2](http://www.ncbi.nlm.nih.gov/gene/?term=1813)(122.778)[DRD4](http://www.ncbi.nlm.nih.gov/gene/?term=1815)(122.778)[HTR1A](http://www.ncbi.nlm.nih.gov/gene/?term=3350)(122.778)[ADRA2C](http://www.ncbi.nlm.nih.gov/gene/?term=152)(122.778) [CALY](http://www.ncbi.nlm.nih.gov/gene/?term=50632)(122.778)[DRD3](http://www.ncbi.nlm.nih.gov/gene/?term=1814)(122.778)[DRD5](http://www.ncbi.nlm.nih.gov/gene/?term=1816)(122.778)[DRD1](http://www.ncbi.nlm.nih.gov/gene/?term=1812)(122.778)[ADRA2B](http://www.ncbi.nlm.nih.gov/gene/?term=151)(122.778) |
| [Cumaldehyde](http://www.megabionet.org/tcmid/ingredient/4353/) | This compound doesn't have any potential target with score larger than 20. |
| [Corylidin](http://www.megabionet.org/tcmid/ingredient/4107/) | This compound doesn't have any potential target with score larger than 20. |
| [Rheumin](http://www.megabionet.org/tcmid/ingredient/18752/) | This compound doesn't have any potential target with score larger than 20. |
| [Reticuline](http://www.megabionet.org/tcmid/ingredient/18640/) | [HTR2A](http://www.ncbi.nlm.nih.gov/gene/?term=3356)(48.000)[CHRM3](http://www.ncbi.nlm.nih.gov/gene/?term=1131)(48.000)[HTR2B](http://www.ncbi.nlm.nih.gov/gene/?term=3357)(48.000)[ADRA2A](http://www.ncbi.nlm.nih.gov/gene/?term=150)(48.000)[HTR1B](http://www.ncbi.nlm.nih.gov/gene/?term=3351)(48.000) [HTR2C](http://www.ncbi.nlm.nih.gov/gene/?term=3358)(48.000)[HTR1D](http://www.ncbi.nlm.nih.gov/gene/?term=3352)(48.000)[DRD2](http://www.ncbi.nlm.nih.gov/gene/?term=1813)(48.000)[ACHE](http://www.ncbi.nlm.nih.gov/gene/?term=43)(48.000)[SLC18A2](http://www.ncbi.nlm.nih.gov/gene/?term=6571)(48.000) [DRD4](http://www.ncbi.nlm.nih.gov/gene/?term=1815)(48.000)[CHRNA2](http://www.ncbi.nlm.nih.gov/gene/?term=1135)(48.000)[HTR1A](http://www.ncbi.nlm.nih.gov/gene/?term=3350)(48.000)[ADRA2C](http://www.ncbi.nlm.nih.gov/gene/?term=152)(48.000)[CALY](http://www.ncbi.nlm.nih.gov/gene/?term=50632)(48.000) [DRD3](http://www.ncbi.nlm.nih.gov/gene/?term=1814)(48.000)[DRD5](http://www.ncbi.nlm.nih.gov/gene/?term=1816)(48.000)[HTR3A](http://www.ncbi.nlm.nih.gov/gene/?term=3359)(48.000)[DRD1](http://www.ncbi.nlm.nih.gov/gene/?term=1812)(48.000)[BCHE](http://www.ncbi.nlm.nih.gov/gene/?term=590)(48.000) [CHRM2](http://www.ncbi.nlm.nih.gov/gene/?term=1129)(48.000)[ADRA2B](http://www.ncbi.nlm.nih.gov/gene/?term=151)(48.000)[ESR2](http://www.ncbi.nlm.nih.gov/gene/?term=2100)(23.000)[OPRD1](http://www.ncbi.nlm.nih.gov/gene/?term=4985)(23.000)[ESR1](http://www.ncbi.nlm.nih.gov/gene/?term=2099)(23.000) [OPRK1](http://www.ncbi.nlm.nih.gov/gene/?term=4986)(23.000)[OPRM1](http://www.ncbi.nlm.nih.gov/gene/?term=4988)(23.000) |
| [Rubixanthin](http://www.megabionet.org/tcmid/ingredient/19024/) | [VDR](http://www.ncbi.nlm.nih.gov/gene/?term=7421)(80.882)[CYP27B1](http://www.ncbi.nlm.nih.gov/gene/?term=1594)(80.882)[GC](http://www.ncbi.nlm.nih.gov/gene/?term=2638)(55.444)[SNW1](http://www.ncbi.nlm.nih.gov/gene/?term=22938)(55.444)[RDH11](http://www.ncbi.nlm.nih.gov/gene/?term=51109)(48.000) [RBP3](http://www.ncbi.nlm.nih.gov/gene/?term=5949)(48.000)[RETSAT](http://www.ncbi.nlm.nih.gov/gene/?term=54884)(48.000)[RDH13](http://www.ncbi.nlm.nih.gov/gene/?term=112724)(48.000)[DHRS3](http://www.ncbi.nlm.nih.gov/gene/?term=9249)(48.000)[RDH5](http://www.ncbi.nlm.nih.gov/gene/?term=5959)(48.000) [ESR1](http://www.ncbi.nlm.nih.gov/gene/?term=2099)(48.000)[PGR](http://www.ncbi.nlm.nih.gov/gene/?term=5241)(48.000)[ALDH1A3](http://www.ncbi.nlm.nih.gov/gene/?term=220)(48.000)[RDH12](http://www.ncbi.nlm.nih.gov/gene/?term=145226)(48.000)[DHRS4](http://www.ncbi.nlm.nih.gov/gene/?term=10901)(48.000) [RBP1](http://www.ncbi.nlm.nih.gov/gene/?term=5947)(48.000)[ALDH1A1](http://www.ncbi.nlm.nih.gov/gene/?term=216)(48.000)[RLBP1](http://www.ncbi.nlm.nih.gov/gene/?term=6017)(48.000)[RDH14](http://www.ncbi.nlm.nih.gov/gene/?term=57665)(48.000)[RDH8](http://www.ncbi.nlm.nih.gov/gene/?term=50700)(48.000) [LRAT](http://www.ncbi.nlm.nih.gov/gene/?term=9227)(48.000)[ALDH1A2](http://www.ncbi.nlm.nih.gov/gene/?term=8854)(48.000)[NFKB1](http://www.ncbi.nlm.nih.gov/gene/?term=4790)(22.373)[AKR1C3](http://www.ncbi.nlm.nih.gov/gene/?term=8644)(22.373)[CYP24A1](http://www.ncbi.nlm.nih.gov/gene/?term=1591)(22.373) [GPBAR1](http://www.ncbi.nlm.nih.gov/gene/?term=151306)(22.373)[SNAI2](http://www.ncbi.nlm.nih.gov/gene/?term=6591)(22.373)[MED1](http://www.ncbi.nlm.nih.gov/gene/?term=5469)(22.373)[SNAI1](http://www.ncbi.nlm.nih.gov/gene/?term=6615)(22.373)[CYP3A4](http://www.ncbi.nlm.nih.gov/gene/?term=1576)(22.373) [CALB1](http://www.ncbi.nlm.nih.gov/gene/?term=793)(22.373)[FGF23](http://www.ncbi.nlm.nih.gov/gene/?term=8074)(22.373)[GFI1](http://www.ncbi.nlm.nih.gov/gene/?term=2672)(22.373)[LANCL2](http://www.ncbi.nlm.nih.gov/gene/?term=55915)(22.373)[WNT4](http://www.ncbi.nlm.nih.gov/gene/?term=54361)(22.373) [TCF3](http://www.ncbi.nlm.nih.gov/gene/?term=6929)(22.373)[BAX](http://www.ncbi.nlm.nih.gov/gene/?term=581)(22.373)[KL](http://www.ncbi.nlm.nih.gov/gene/?term=9365)(22.373)[CYP2R1](http://www.ncbi.nlm.nih.gov/gene/?term=120227)(22.373)[PML](http://www.ncbi.nlm.nih.gov/gene/?term=5371)(22.373) [B4GALT1](http://www.ncbi.nlm.nih.gov/gene/?term=2683)(22.373)[S100G](http://www.ncbi.nlm.nih.gov/gene/?term=795)(22.373)[CYP27A1](http://www.ncbi.nlm.nih.gov/gene/?term=1593)(22.373)[KANK2](http://www.ncbi.nlm.nih.gov/gene/?term=25959)(22.373)[IRX5](http://www.ncbi.nlm.nih.gov/gene/?term=10265)(22.373) [RXRA](http://www.ncbi.nlm.nih.gov/gene/?term=6256)(22.373)[NR1H4](http://www.ncbi.nlm.nih.gov/gene/?term=9971)(22.373)[TRIM24](http://www.ncbi.nlm.nih.gov/gene/?term=8805)(22.373) |
| [Eugenol Methyl Ether](http://www.megabionet.org/tcmid/ingredient/7522/) | This compound doesn't have any potential target with score larger than 20. |
| [Isocorynoline](http://www.megabionet.org/tcmid/ingredient/11343/) | [DRD2](http://www.ncbi.nlm.nih.gov/gene/?term=1813)(48.000)[SLC18A2](http://www.ncbi.nlm.nih.gov/gene/?term=6571)(48.000)[CHRNA2](http://www.ncbi.nlm.nih.gov/gene/?term=1135)(48.000)[CHRM2](http://www.ncbi.nlm.nih.gov/gene/?term=1129)(48.000) |
| [Isocrotonylpterosin B](http://www.megabionet.org/tcmid/ingredient/31340/) | [AR](http://www.ncbi.nlm.nih.gov/gene/?term=367)(80.882)[F2](http://www.ncbi.nlm.nih.gov/gene/?term=2147)(80.882)[GPR27](http://www.ncbi.nlm.nih.gov/gene/?term=2850)(55.444)[ESR1](http://www.ncbi.nlm.nih.gov/gene/?term=2099)(23.000)[PGR](http://www.ncbi.nlm.nih.gov/gene/?term=5241)(23.000) [NR3C1](http://www.ncbi.nlm.nih.gov/gene/?term=2908)(23.000)[COL5A1](http://www.ncbi.nlm.nih.gov/gene/?term=1289)(22.373)[NOX1](http://www.ncbi.nlm.nih.gov/gene/?term=27035)(22.373)[CD36](http://www.ncbi.nlm.nih.gov/gene/?term=948)(22.373)[SDC4](http://www.ncbi.nlm.nih.gov/gene/?term=6385)(22.373) [NKX3-1](http://www.ncbi.nlm.nih.gov/gene/?term=4824)(22.373)[SHBG](http://www.ncbi.nlm.nih.gov/gene/?term=6462)(22.373)[TGFB2](http://www.ncbi.nlm.nih.gov/gene/?term=7042)(22.373)[CD47](http://www.ncbi.nlm.nih.gov/gene/?term=961)(22.373)[BICD1](http://www.ncbi.nlm.nih.gov/gene/?term=636)(22.373) [NR1I3](http://www.ncbi.nlm.nih.gov/gene/?term=9970)(22.373)[ALDH1A1](http://www.ncbi.nlm.nih.gov/gene/?term=216)(22.373)[TSPO](http://www.ncbi.nlm.nih.gov/gene/?term=706)(22.373)[S100A9](http://www.ncbi.nlm.nih.gov/gene/?term=6280)(22.373) |
| [Alpha-Humulene](http://www.megabionet.org/tcmid/ingredient/23256/) | [KCND1](http://www.ncbi.nlm.nih.gov/gene/?term=3750)(80.882)[KCNA3](http://www.ncbi.nlm.nih.gov/gene/?term=3738)(80.882)[PRKAB1](http://www.ncbi.nlm.nih.gov/gene/?term=5564)(80.882)[ADH1A](http://www.ncbi.nlm.nih.gov/gene/?term=124)(80.882)[KCNA10](http://www.ncbi.nlm.nih.gov/gene/?term=3744)(80.882) [GAMT](http://www.ncbi.nlm.nih.gov/gene/?term=2593)(80.882)[KCNC3](http://www.ncbi.nlm.nih.gov/gene/?term=3748)(80.882)[KCNA1](http://www.ncbi.nlm.nih.gov/gene/?term=3736)(80.882)[KCNA2](http://www.ncbi.nlm.nih.gov/gene/?term=3737)(80.882)[TPO](http://www.ncbi.nlm.nih.gov/gene/?term=7173)(80.882) [CAT](http://www.ncbi.nlm.nih.gov/gene/?term=847)(80.882)[KCNB1](http://www.ncbi.nlm.nih.gov/gene/?term=3745)(80.882)[ADH1B](http://www.ncbi.nlm.nih.gov/gene/?term=125)(80.882)[DLG4](http://www.ncbi.nlm.nih.gov/gene/?term=1742)(80.882)[KCNC2](http://www.ncbi.nlm.nih.gov/gene/?term=3747)(80.882) [KCNC1](http://www.ncbi.nlm.nih.gov/gene/?term=3746)(80.882)[ADH1C](http://www.ncbi.nlm.nih.gov/gene/?term=126)(80.882)[KCNB2](http://www.ncbi.nlm.nih.gov/gene/?term=9312)(80.882)[KCNA5](http://www.ncbi.nlm.nih.gov/gene/?term=3741)(80.882)[RNASE1](http://www.ncbi.nlm.nih.gov/gene/?term=6035)(80.882) [KCND2](http://www.ncbi.nlm.nih.gov/gene/?term=3751)(80.882)[ALDH2](http://www.ncbi.nlm.nih.gov/gene/?term=217)(80.882)[KCNA4](http://www.ncbi.nlm.nih.gov/gene/?term=3739)(80.882)[KCNA7](http://www.ncbi.nlm.nih.gov/gene/?term=3743)(80.882)[KCNA6](http://www.ncbi.nlm.nih.gov/gene/?term=3742)(80.882) [KCND3](http://www.ncbi.nlm.nih.gov/gene/?term=3752)(80.882)[GUCY1B3](http://www.ncbi.nlm.nih.gov/gene/?term=2983)(55.444)[GATM](http://www.ncbi.nlm.nih.gov/gene/?term=2628)(55.444)[KCNK4](http://www.ncbi.nlm.nih.gov/gene/?term=50801)(55.444)[IYD](http://www.ncbi.nlm.nih.gov/gene/?term=389434)(55.444) [KCNQ1](http://www.ncbi.nlm.nih.gov/gene/?term=3784)(55.444)[FBP1](http://www.ncbi.nlm.nih.gov/gene/?term=2203)(22.373)[DGKI](http://www.ncbi.nlm.nih.gov/gene/?term=9162)(22.373)[ADH7](http://www.ncbi.nlm.nih.gov/gene/?term=131)(22.373)[NFIB](http://www.ncbi.nlm.nih.gov/gene/?term=4781)(22.373) [CYGB](http://www.ncbi.nlm.nih.gov/gene/?term=114757)(22.373)[NRXN3](http://www.ncbi.nlm.nih.gov/gene/?term=9369)(22.373)[LRRC4B](http://www.ncbi.nlm.nih.gov/gene/?term=94030)(22.373)[AQP8](http://www.ncbi.nlm.nih.gov/gene/?term=343)(22.373)[ARX](http://www.ncbi.nlm.nih.gov/gene/?term=170302)(22.373) [IGF1](http://www.ncbi.nlm.nih.gov/gene/?term=3479)(22.373)[SHANK3](http://www.ncbi.nlm.nih.gov/gene/?term=85358)(22.373)[ACY3](http://www.ncbi.nlm.nih.gov/gene/?term=91703)(22.373)[ASPA](http://www.ncbi.nlm.nih.gov/gene/?term=443)(22.373)[NRXN1](http://www.ncbi.nlm.nih.gov/gene/?term=9378)(22.373) [EDN1](http://www.ncbi.nlm.nih.gov/gene/?term=1906)(22.373)[OXT](http://www.ncbi.nlm.nih.gov/gene/?term=5020)(22.373)[MAGI2](http://www.ncbi.nlm.nih.gov/gene/?term=9863)(22.373)[APOE](http://www.ncbi.nlm.nih.gov/gene/?term=348)(22.373)[SOX15](http://www.ncbi.nlm.nih.gov/gene/?term=6665)(22.373) [CNTNAP4](http://www.ncbi.nlm.nih.gov/gene/?term=85445)(22.373)[RNASE4](http://www.ncbi.nlm.nih.gov/gene/?term=6038)(22.373)[RAB3A](http://www.ncbi.nlm.nih.gov/gene/?term=5864)(22.373)[MC4R](http://www.ncbi.nlm.nih.gov/gene/?term=4160)(22.373)[IL1B](http://www.ncbi.nlm.nih.gov/gene/?term=3553)(22.373) [GRIN2A](http://www.ncbi.nlm.nih.gov/gene/?term=2903)(22.373)[SLC17A7](http://www.ncbi.nlm.nih.gov/gene/?term=57030)(22.373)[CYP11A1](http://www.ncbi.nlm.nih.gov/gene/?term=1583)(22.373)[SCN5A](http://www.ncbi.nlm.nih.gov/gene/?term=6331)(22.373)[SCN10A](http://www.ncbi.nlm.nih.gov/gene/?term=6336)(22.373) [SPARC](http://www.ncbi.nlm.nih.gov/gene/?term=6678)(22.373)[SORCS3](http://www.ncbi.nlm.nih.gov/gene/?term=22986)(22.373)[RNASE8](http://www.ncbi.nlm.nih.gov/gene/?term=122665)(22.373)[KCNIP2](http://www.ncbi.nlm.nih.gov/gene/?term=30819)(22.373)[GPX7](http://www.ncbi.nlm.nih.gov/gene/?term=2882)(22.373) [CRLF1](http://www.ncbi.nlm.nih.gov/gene/?term=9244)(22.373)[FADD](http://www.ncbi.nlm.nih.gov/gene/?term=8772)(22.373)[SNTG2](http://www.ncbi.nlm.nih.gov/gene/?term=54221)(22.373)[SCGB1A1](http://www.ncbi.nlm.nih.gov/gene/?term=7356)(22.373)[OXTR](http://www.ncbi.nlm.nih.gov/gene/?term=5021)(22.373) [ALDH1B1](http://www.ncbi.nlm.nih.gov/gene/?term=219)(22.373)[LEP](http://www.ncbi.nlm.nih.gov/gene/?term=3952)(22.373)[NLGN1](http://www.ncbi.nlm.nih.gov/gene/?term=22871)(22.373)[CACNA1D](http://www.ncbi.nlm.nih.gov/gene/?term=776)(22.373)[RAPGEF2](http://www.ncbi.nlm.nih.gov/gene/?term=9693)(22.373) [DARS](http://www.ncbi.nlm.nih.gov/gene/?term=1615)(22.373)[ANK3](http://www.ncbi.nlm.nih.gov/gene/?term=288)(22.373)[KCNE5](http://www.ncbi.nlm.nih.gov/gene/?term=23630)(22.373)[PPARD](http://www.ncbi.nlm.nih.gov/gene/?term=5467)(22.373)[NKX2-1](http://www.ncbi.nlm.nih.gov/gene/?term=7080)(22.373) [MIP](http://www.ncbi.nlm.nih.gov/gene/?term=4284)(22.373)[RYR3](http://www.ncbi.nlm.nih.gov/gene/?term=6263)(22.373)[ZPR1](http://www.ncbi.nlm.nih.gov/gene/?term=8882)(22.373)[ALDH3B1](http://www.ncbi.nlm.nih.gov/gene/?term=221)(22.373)[NPPA](http://www.ncbi.nlm.nih.gov/gene/?term=4878)(22.373) [STX1A](http://www.ncbi.nlm.nih.gov/gene/?term=6804)(22.373)[PAXBP1](http://www.ncbi.nlm.nih.gov/gene/?term=94104)(22.373)[PAX7](http://www.ncbi.nlm.nih.gov/gene/?term=5081)(22.373)[ALDH3B2](http://www.ncbi.nlm.nih.gov/gene/?term=222)(22.373)[RNASE2](http://www.ncbi.nlm.nih.gov/gene/?term=6036)(22.373) [NRXN2](http://www.ncbi.nlm.nih.gov/gene/?term=9379)(22.373)[ADORA1](http://www.ncbi.nlm.nih.gov/gene/?term=134)(22.373)[ADH4](http://www.ncbi.nlm.nih.gov/gene/?term=127)(22.373)[GJA5](http://www.ncbi.nlm.nih.gov/gene/?term=2702)(22.373)[FAS](http://www.ncbi.nlm.nih.gov/gene/?term=355)(22.373) [DAB2IP](http://www.ncbi.nlm.nih.gov/gene/?term=153090)(22.373)[CYP2E1](http://www.ncbi.nlm.nih.gov/gene/?term=1571)(22.373)[ACY1](http://www.ncbi.nlm.nih.gov/gene/?term=95)(22.373) |
| [Rugosin E](http://www.megabionet.org/tcmid/ingredient/19043/) | This compound doesn't have any potential target with score larger than 20. |
| [Chrysophanol-8-O-Beta-D-(6'-O-Galloyl)-Glucopyranoside](http://www.megabionet.org/tcmid/ingredient/30775/) | This compound doesn't have any potential target with score larger than 20. |
| [3'-O-Methyl Sappanol](http://www.megabionet.org/tcmid/ingredient/14712/) | [SEC14L3](http://www.ncbi.nlm.nih.gov/gene/?term=266629)(48.000)[SOAT1](http://www.ncbi.nlm.nih.gov/gene/?term=6646)(48.000)[MTTP](http://www.ncbi.nlm.nih.gov/gene/?term=4547)(48.000)[CNR2](http://www.ncbi.nlm.nih.gov/gene/?term=1269)(48.000)[PPP2CA](http://www.ncbi.nlm.nih.gov/gene/?term=5515)(48.000) [PRKCA](http://www.ncbi.nlm.nih.gov/gene/?term=5578)(48.000)[NR1I2](http://www.ncbi.nlm.nih.gov/gene/?term=8856)(48.000)[CNR1](http://www.ncbi.nlm.nih.gov/gene/?term=1268)(48.000)[ALOX5](http://www.ncbi.nlm.nih.gov/gene/?term=240)(48.000)[PPP2CB](http://www.ncbi.nlm.nih.gov/gene/?term=5516)(48.000) [SEC14L2](http://www.ncbi.nlm.nih.gov/gene/?term=23541)(48.000)[DGKA](http://www.ncbi.nlm.nih.gov/gene/?term=1606)(48.000)[PRKCB](http://www.ncbi.nlm.nih.gov/gene/?term=5579)(48.000)[SOAT2](http://www.ncbi.nlm.nih.gov/gene/?term=8435)(48.000)[SEC14L4](http://www.ncbi.nlm.nih.gov/gene/?term=284904)(48.000) |
| [(+)-Erythro-Guaiacylglycerol](http://www.megabionet.org/tcmid/ingredient/25768/) | [ADRA1A](http://www.ncbi.nlm.nih.gov/gene/?term=148)(48.000)[SLC6A2](http://www.ncbi.nlm.nih.gov/gene/?term=6530)(48.000) |
| [Corycavine](http://www.megabionet.org/tcmid/ingredient/4099/) | [PDGFRB](http://www.ncbi.nlm.nih.gov/gene/?term=5159)(23.000)[NTRK1](http://www.ncbi.nlm.nih.gov/gene/?term=4914)(23.000)[CSF1R](http://www.ncbi.nlm.nih.gov/gene/?term=1436)(23.000)[PDGFRA](http://www.ncbi.nlm.nih.gov/gene/?term=5156)(23.000)[KIT](http://www.ncbi.nlm.nih.gov/gene/?term=3815)(23.000) [ABL1](http://www.ncbi.nlm.nih.gov/gene/?term=25)(23.000)[DDR1](http://www.ncbi.nlm.nih.gov/gene/?term=780)(23.000)[HTR3A](http://www.ncbi.nlm.nih.gov/gene/?term=3359)(23.000)[CSF1](http://www.ncbi.nlm.nih.gov/gene/?term=1435)(22.373)[EPOR](http://www.ncbi.nlm.nih.gov/gene/?term=2057)(22.373) [DDR2](http://www.ncbi.nlm.nih.gov/gene/?term=4921)(22.373)[PDGFRL](http://www.ncbi.nlm.nih.gov/gene/?term=5157)(22.373)[BICD1](http://www.ncbi.nlm.nih.gov/gene/?term=636)(22.373)[ZEB2](http://www.ncbi.nlm.nih.gov/gene/?term=9839)(22.373)[MST1R](http://www.ncbi.nlm.nih.gov/gene/?term=4486)(22.373) [NRP1](http://www.ncbi.nlm.nih.gov/gene/?term=8829)(22.373)[SORT1](http://www.ncbi.nlm.nih.gov/gene/?term=6272)(22.373) |
| [Casuarictin](http://www.megabionet.org/tcmid/ingredient/3301/) | This compound doesn't have any potential target with score larger than 20. |
| [Rhein Diglucoside](http://www.megabionet.org/tcmid/ingredient/18745/) | This compound doesn't have any potential target with score larger than 20. |
| [1-O-Galloyl-Glycerol](http://www.megabionet.org/tcmid/ingredient/8109/) | This compound doesn't have any potential target with score larger than 20. |
| [Arabic Acid](http://www.megabionet.org/tcmid/ingredient/24176/) | [TOP1](http://www.ncbi.nlm.nih.gov/gene/?term=7150)(122.778)[GABRB1](http://www.ncbi.nlm.nih.gov/gene/?term=2560)(48.000)[SLC52A2](http://www.ncbi.nlm.nih.gov/gene/?term=79581)(48.000)[TOP2A](http://www.ncbi.nlm.nih.gov/gene/?term=7153)(22.373)[CDC45](http://www.ncbi.nlm.nih.gov/gene/?term=8318)(22.373) [TOP3A](http://www.ncbi.nlm.nih.gov/gene/?term=7156)(22.373)[TOP3B](http://www.ncbi.nlm.nih.gov/gene/?term=8940)(22.373)[MCM10](http://www.ncbi.nlm.nih.gov/gene/?term=55388)(22.373)[GINS4](http://www.ncbi.nlm.nih.gov/gene/?term=84296)(22.373)[TOP1MT](http://www.ncbi.nlm.nih.gov/gene/?term=116447)(22.373) [TOP2B](http://www.ncbi.nlm.nih.gov/gene/?term=7155)(22.373)[GINS2](http://www.ncbi.nlm.nih.gov/gene/?term=51659)(22.373) |
| [(E)-4-Phenyl-3-Buten-2-One](http://www.megabionet.org/tcmid/ingredient/17087/) | [HTR4](http://www.ncbi.nlm.nih.gov/gene/?term=3360)(80.882)[GABRA2](http://www.ncbi.nlm.nih.gov/gene/?term=2555)(80.882)[PROS1](http://www.ncbi.nlm.nih.gov/gene/?term=5627)(80.882)[PROZ](http://www.ncbi.nlm.nih.gov/gene/?term=8858)(80.882)[AVPR1A](http://www.ncbi.nlm.nih.gov/gene/?term=552)(80.882) [NQO2](http://www.ncbi.nlm.nih.gov/gene/?term=4835)(80.882)[HTR1B](http://www.ncbi.nlm.nih.gov/gene/?term=3351)(80.882)[GABRA3](http://www.ncbi.nlm.nih.gov/gene/?term=2556)(80.882)[F7](http://www.ncbi.nlm.nih.gov/gene/?term=2155)(80.882)[F9](http://www.ncbi.nlm.nih.gov/gene/?term=2158)(80.882) [GGCX](http://www.ncbi.nlm.nih.gov/gene/?term=2677)(80.882)[F10](http://www.ncbi.nlm.nih.gov/gene/?term=2159)(80.882)[VKORC1](http://www.ncbi.nlm.nih.gov/gene/?term=79001)(80.882)[JUN](http://www.ncbi.nlm.nih.gov/gene/?term=3725)(80.882)[PROC](http://www.ncbi.nlm.nih.gov/gene/?term=5624)(80.882) [OPRM1](http://www.ncbi.nlm.nih.gov/gene/?term=4988)(80.882)[AGTR2](http://www.ncbi.nlm.nih.gov/gene/?term=186)(80.882)[BGLAP](http://www.ncbi.nlm.nih.gov/gene/?term=632)(80.882)[PTGS1](http://www.ncbi.nlm.nih.gov/gene/?term=5742)(80.882)[AVPR2](http://www.ncbi.nlm.nih.gov/gene/?term=554)(80.882) [HTR1A](http://www.ncbi.nlm.nih.gov/gene/?term=3350)(80.882)[PTGS2](http://www.ncbi.nlm.nih.gov/gene/?term=5743)(80.882)[HTR3A](http://www.ncbi.nlm.nih.gov/gene/?term=3359)(80.882)[GABRA1](http://www.ncbi.nlm.nih.gov/gene/?term=2554)(80.882)[NQO1](http://www.ncbi.nlm.nih.gov/gene/?term=1728)(80.882) [AGTR1](http://www.ncbi.nlm.nih.gov/gene/?term=185)(80.882)[VKORC1L1](http://www.ncbi.nlm.nih.gov/gene/?term=154807)(80.882)[F2](http://www.ncbi.nlm.nih.gov/gene/?term=2147)(80.882)[AGT](http://www.ncbi.nlm.nih.gov/gene/?term=183)(55.444)[CBR4](http://www.ncbi.nlm.nih.gov/gene/?term=84869)(55.444) [AVP](http://www.ncbi.nlm.nih.gov/gene/?term=551)(55.444)[PAX2](http://www.ncbi.nlm.nih.gov/gene/?term=5076)(55.444)[DAB2](http://www.ncbi.nlm.nih.gov/gene/?term=1601)(55.444)[MGP](http://www.ncbi.nlm.nih.gov/gene/?term=4256)(55.444)[F3](http://www.ncbi.nlm.nih.gov/gene/?term=2152)(55.444) [WLS](http://www.ncbi.nlm.nih.gov/gene/?term=79971)(55.444)[GPR27](http://www.ncbi.nlm.nih.gov/gene/?term=2850)(55.444)[SERPINB7](http://www.ncbi.nlm.nih.gov/gene/?term=8710)(55.444)[ACTN2](http://www.ncbi.nlm.nih.gov/gene/?term=88)(55.444)[NDRG2](http://www.ncbi.nlm.nih.gov/gene/?term=57447)(22.373) [PCK1](http://www.ncbi.nlm.nih.gov/gene/?term=5105)(22.373)[ADCY6](http://www.ncbi.nlm.nih.gov/gene/?term=112)(22.373)[NAMPT](http://www.ncbi.nlm.nih.gov/gene/?term=10135)(22.373)[RGCC](http://www.ncbi.nlm.nih.gov/gene/?term=28984)(22.373)[HACD1](http://www.ncbi.nlm.nih.gov/gene/?term=9200)(22.373) [CSF1](http://www.ncbi.nlm.nih.gov/gene/?term=1435)(22.373)[IL34](http://www.ncbi.nlm.nih.gov/gene/?term=146433)(22.373)[C1QTNF1](http://www.ncbi.nlm.nih.gov/gene/?term=114897)(22.373)[MC2R](http://www.ncbi.nlm.nih.gov/gene/?term=4158)(22.373)[UTS2](http://www.ncbi.nlm.nih.gov/gene/?term=10911)(22.373) [FNDC5](http://www.ncbi.nlm.nih.gov/gene/?term=252995)(22.373)[ACIN1](http://www.ncbi.nlm.nih.gov/gene/?term=22985)(22.373)[ANG](http://www.ncbi.nlm.nih.gov/gene/?term=283)(22.373)[CD36](http://www.ncbi.nlm.nih.gov/gene/?term=948)(22.373)[SDC4](http://www.ncbi.nlm.nih.gov/gene/?term=6385)(22.373) [CA7](http://www.ncbi.nlm.nih.gov/gene/?term=766)(22.373)[ADRB2](http://www.ncbi.nlm.nih.gov/gene/?term=154)(22.373)[WT1](http://www.ncbi.nlm.nih.gov/gene/?term=7490)(22.373)[HNF1B](http://www.ncbi.nlm.nih.gov/gene/?term=6928)(22.373)[OXT](http://www.ncbi.nlm.nih.gov/gene/?term=5020)(22.373) [SLC38A3](http://www.ncbi.nlm.nih.gov/gene/?term=10991)(22.373)[CCL5](http://www.ncbi.nlm.nih.gov/gene/?term=6352)(22.373)[PLA2R1](http://www.ncbi.nlm.nih.gov/gene/?term=22925)(22.373)[CLDN5](http://www.ncbi.nlm.nih.gov/gene/?term=7122)(22.373)[DCSTAMP](http://www.ncbi.nlm.nih.gov/gene/?term=81501)(22.373) [RXFP4](http://www.ncbi.nlm.nih.gov/gene/?term=339403)(22.373)[METRNL](http://www.ncbi.nlm.nih.gov/gene/?term=284207)(22.373)[PPP1R9B](http://www.ncbi.nlm.nih.gov/gene/?term=84687)(22.373)[HTR2B](http://www.ncbi.nlm.nih.gov/gene/?term=3357)(22.373)[COMT](http://www.ncbi.nlm.nih.gov/gene/?term=1312)(22.373) [MAPK9](http://www.ncbi.nlm.nih.gov/gene/?term=5601)(22.373)[LILRB1](http://www.ncbi.nlm.nih.gov/gene/?term=10859)(22.373)[PKD2](http://www.ncbi.nlm.nih.gov/gene/?term=5311)(22.373)[AQP2](http://www.ncbi.nlm.nih.gov/gene/?term=359)(22.373)[MC1R](http://www.ncbi.nlm.nih.gov/gene/?term=4157)(22.373) [MC4R](http://www.ncbi.nlm.nih.gov/gene/?term=4160)(22.373)[CYP4F2](http://www.ncbi.nlm.nih.gov/gene/?term=8529)(22.373)[CRHBP](http://www.ncbi.nlm.nih.gov/gene/?term=1393)(22.373)[CYP11A1](http://www.ncbi.nlm.nih.gov/gene/?term=1583)(22.373)[PTGIS](http://www.ncbi.nlm.nih.gov/gene/?term=5740)(22.373) [MTNR1B](http://www.ncbi.nlm.nih.gov/gene/?term=4544)(22.373)[PPP1R1B](http://www.ncbi.nlm.nih.gov/gene/?term=84152)(22.373)[HTN1](http://www.ncbi.nlm.nih.gov/gene/?term=3346)(22.373)[ACE2](http://www.ncbi.nlm.nih.gov/gene/?term=59272)(22.373)[CYP4F11](http://www.ncbi.nlm.nih.gov/gene/?term=57834)(22.373) [MAS1](http://www.ncbi.nlm.nih.gov/gene/?term=4142)(22.373)[BMP6](http://www.ncbi.nlm.nih.gov/gene/?term=654)(22.373)[MAOB](http://www.ncbi.nlm.nih.gov/gene/?term=4129)(22.373)[SIPA1](http://www.ncbi.nlm.nih.gov/gene/?term=6494)(22.373)[AMELX](http://www.ncbi.nlm.nih.gov/gene/?term=265)(22.373) [S1PR2](http://www.ncbi.nlm.nih.gov/gene/?term=9294)(22.373)[EGR1](http://www.ncbi.nlm.nih.gov/gene/?term=1958)(22.373)[SERPINF2](http://www.ncbi.nlm.nih.gov/gene/?term=5345)(22.373)[SPRY1](http://www.ncbi.nlm.nih.gov/gene/?term=10252)(22.373)[HRH3](http://www.ncbi.nlm.nih.gov/gene/?term=11255)(22.373) [HEG1](http://www.ncbi.nlm.nih.gov/gene/?term=57493)(22.373)[AGTRAP](http://www.ncbi.nlm.nih.gov/gene/?term=57085)(22.373)[BCL2](http://www.ncbi.nlm.nih.gov/gene/?term=596)(22.373)[GAS6](http://www.ncbi.nlm.nih.gov/gene/?term=2621)(22.373)[CTNNBIP1](http://www.ncbi.nlm.nih.gov/gene/?term=56998)(22.373) [CDH5](http://www.ncbi.nlm.nih.gov/gene/?term=1003)(22.373)[TFPI](http://www.ncbi.nlm.nih.gov/gene/?term=7035)(22.373)[OXTR](http://www.ncbi.nlm.nih.gov/gene/?term=5021)(22.373)[PPP1R15A](http://www.ncbi.nlm.nih.gov/gene/?term=23645)(22.373)[PLA2G1B](http://www.ncbi.nlm.nih.gov/gene/?term=5319)(22.373) [KCNK4](http://www.ncbi.nlm.nih.gov/gene/?term=50801)(22.373)[SOD1](http://www.ncbi.nlm.nih.gov/gene/?term=6647)(22.373)[CCS](http://www.ncbi.nlm.nih.gov/gene/?term=9973)(22.373)[UTS2R](http://www.ncbi.nlm.nih.gov/gene/?term=2837)(22.373)[ACVR2A](http://www.ncbi.nlm.nih.gov/gene/?term=92)(22.373) [SLC6A4](http://www.ncbi.nlm.nih.gov/gene/?term=6532)(22.373)[ARRB1](http://www.ncbi.nlm.nih.gov/gene/?term=408)(22.373)[AIF1](http://www.ncbi.nlm.nih.gov/gene/?term=199)(22.373)[BDKRB2](http://www.ncbi.nlm.nih.gov/gene/?term=624)(22.373)[HCRT](http://www.ncbi.nlm.nih.gov/gene/?term=3060)(22.373) [HTR3D](http://www.ncbi.nlm.nih.gov/gene/?term=200909)(22.373)[NEUROD2](http://www.ncbi.nlm.nih.gov/gene/?term=4761)(22.373)[NMUR2](http://www.ncbi.nlm.nih.gov/gene/?term=56923)(22.373)[INS](http://www.ncbi.nlm.nih.gov/gene/?term=3630)(22.373)[ACE](http://www.ncbi.nlm.nih.gov/gene/?term=1636)(22.373) [ANAPC2](http://www.ncbi.nlm.nih.gov/gene/?term=29882)(22.373)[WNT11](http://www.ncbi.nlm.nih.gov/gene/?term=7481)(22.373)[RPS6KA2](http://www.ncbi.nlm.nih.gov/gene/?term=6196)(22.373)[CD47](http://www.ncbi.nlm.nih.gov/gene/?term=961)(22.373)[ALOX15B](http://www.ncbi.nlm.nih.gov/gene/?term=247)(22.373) [BICD1](http://www.ncbi.nlm.nih.gov/gene/?term=636)(22.373)[ACSL4](http://www.ncbi.nlm.nih.gov/gene/?term=2182)(22.373)[CDC20](http://www.ncbi.nlm.nih.gov/gene/?term=991)(22.373)[SPX](http://www.ncbi.nlm.nih.gov/gene/?term=80763)(22.373)[UGT3A2](http://www.ncbi.nlm.nih.gov/gene/?term=167127)(22.373) [RET](http://www.ncbi.nlm.nih.gov/gene/?term=5979)(22.373)[LRRK2](http://www.ncbi.nlm.nih.gov/gene/?term=120892)(22.373)[SOD3](http://www.ncbi.nlm.nih.gov/gene/?term=6649)(22.373)[THRB](http://www.ncbi.nlm.nih.gov/gene/?term=7068)(22.373)[REN](http://www.ncbi.nlm.nih.gov/gene/?term=5972)(22.373) [SOD2](http://www.ncbi.nlm.nih.gov/gene/?term=6648)(22.373)[MC5R](http://www.ncbi.nlm.nih.gov/gene/?term=4161)(22.373)[MC3R](http://www.ncbi.nlm.nih.gov/gene/?term=4159)(22.373)[HTR3B](http://www.ncbi.nlm.nih.gov/gene/?term=9177)(22.373)[SPRY2](http://www.ncbi.nlm.nih.gov/gene/?term=10253)(22.373) [STATH](http://www.ncbi.nlm.nih.gov/gene/?term=6779)(22.373)[HTR3E](http://www.ncbi.nlm.nih.gov/gene/?term=285242)(22.373)[DRD5](http://www.ncbi.nlm.nih.gov/gene/?term=1816)(22.373)[RFK](http://www.ncbi.nlm.nih.gov/gene/?term=55312)(22.373)[NCK2](http://www.ncbi.nlm.nih.gov/gene/?term=8440)(22.373) [TMBIM6](http://www.ncbi.nlm.nih.gov/gene/?term=7009)(22.373)[NAPRT](http://www.ncbi.nlm.nih.gov/gene/?term=93100)(22.373)[MECOM](http://www.ncbi.nlm.nih.gov/gene/?term=2122)(22.373)[AGER](http://www.ncbi.nlm.nih.gov/gene/?term=177)(22.373)[FGA](http://www.ncbi.nlm.nih.gov/gene/?term=2243)(22.373) [SNCA](http://www.ncbi.nlm.nih.gov/gene/?term=6622)(22.373)[NCK1](http://www.ncbi.nlm.nih.gov/gene/?term=4690)(22.373)[ADIPOQ](http://www.ncbi.nlm.nih.gov/gene/?term=9370)(22.373)[CPLX2](http://www.ncbi.nlm.nih.gov/gene/?term=10814)(22.373)[CA2](http://www.ncbi.nlm.nih.gov/gene/?term=760)(22.373) [ADORA1](http://www.ncbi.nlm.nih.gov/gene/?term=134)(22.373)[ALOX15](http://www.ncbi.nlm.nih.gov/gene/?term=246)(22.373)[ANXA1](http://www.ncbi.nlm.nih.gov/gene/?term=301)(22.373)[UBIAD1](http://www.ncbi.nlm.nih.gov/gene/?term=29914)(22.373)[AVPR1B](http://www.ncbi.nlm.nih.gov/gene/?term=553)(22.373) [EGFR](http://www.ncbi.nlm.nih.gov/gene/?term=1956)(22.373)[CBR1](http://www.ncbi.nlm.nih.gov/gene/?term=873)(22.373)[HTR3C](http://www.ncbi.nlm.nih.gov/gene/?term=170572)(22.373)[GLRA1](http://www.ncbi.nlm.nih.gov/gene/?term=2741)(22.373)[PRDM16](http://www.ncbi.nlm.nih.gov/gene/?term=63976)(22.373) [CCM2L](http://www.ncbi.nlm.nih.gov/gene/?term=140706)(22.373) |
| [Eugenin](http://www.megabionet.org/tcmid/ingredient/7518/) | [S100P](http://www.ncbi.nlm.nih.gov/gene/?term=6286)(48.000)[KCNMA1](http://www.ncbi.nlm.nih.gov/gene/?term=3778)(48.000) |
| [Corytuberine](http://www.megabionet.org/tcmid/ingredient/4123/) | [HTR2A](http://www.ncbi.nlm.nih.gov/gene/?term=3356)(122.778)[HTR2B](http://www.ncbi.nlm.nih.gov/gene/?term=3357)(122.778)[ADRA2A](http://www.ncbi.nlm.nih.gov/gene/?term=150)(122.778)[HTR1B](http://www.ncbi.nlm.nih.gov/gene/?term=3351)(122.778)[HTR2C](http://www.ncbi.nlm.nih.gov/gene/?term=3358)(122.778) [HTR1D](http://www.ncbi.nlm.nih.gov/gene/?term=3352)(122.778)[DRD2](http://www.ncbi.nlm.nih.gov/gene/?term=1813)(122.778)[DRD4](http://www.ncbi.nlm.nih.gov/gene/?term=1815)(122.778)[HTR1A](http://www.ncbi.nlm.nih.gov/gene/?term=3350)(122.778)[ADRA2C](http://www.ncbi.nlm.nih.gov/gene/?term=152)(122.778) [CALY](http://www.ncbi.nlm.nih.gov/gene/?term=50632)(122.778)[DRD3](http://www.ncbi.nlm.nih.gov/gene/?term=1814)(122.778)[DRD5](http://www.ncbi.nlm.nih.gov/gene/?term=1816)(122.778)[DRD1](http://www.ncbi.nlm.nih.gov/gene/?term=1812)(122.778)[ADRA2B](http://www.ncbi.nlm.nih.gov/gene/?term=151)(122.778) [ESR2](http://www.ncbi.nlm.nih.gov/gene/?term=2100)(23.000)[OPRD1](http://www.ncbi.nlm.nih.gov/gene/?term=4985)(23.000)[ESR1](http://www.ncbi.nlm.nih.gov/gene/?term=2099)(23.000)[OPRK1](http://www.ncbi.nlm.nih.gov/gene/?term=4986)(23.000)[OPRM1](http://www.ncbi.nlm.nih.gov/gene/?term=4988)(23.000) |
| [8-Geranyloxy Psoralen](http://www.megabionet.org/tcmid/ingredient/8325/) | This compound doesn't have any potential target with score larger than 20. |
| [Corchoroside A](http://www.megabionet.org/tcmid/ingredient/4036/) | [ATP1A1](http://www.ncbi.nlm.nih.gov/gene/?term=476)(122.778)[NR3C1](http://www.ncbi.nlm.nih.gov/gene/?term=2908)(48.000) |
| [Bicuculline](http://www.megabionet.org/tcmid/ingredient/2350/) | This compound doesn't have any potential target with score larger than 20. |
| [Corydine](http://www.megabionet.org/tcmid/ingredient/4106/) | [HTR2A](http://www.ncbi.nlm.nih.gov/gene/?term=3356)(122.778)[HTR2B](http://www.ncbi.nlm.nih.gov/gene/?term=3357)(122.778)[ADRA2A](http://www.ncbi.nlm.nih.gov/gene/?term=150)(122.778)[HTR1B](http://www.ncbi.nlm.nih.gov/gene/?term=3351)(122.778)[HTR2C](http://www.ncbi.nlm.nih.gov/gene/?term=3358)(122.778) [HTR1D](http://www.ncbi.nlm.nih.gov/gene/?term=3352)(122.778)[DRD2](http://www.ncbi.nlm.nih.gov/gene/?term=1813)(122.778)[DRD4](http://www.ncbi.nlm.nih.gov/gene/?term=1815)(122.778)[HTR1A](http://www.ncbi.nlm.nih.gov/gene/?term=3350)(122.778)[ADRA2C](http://www.ncbi.nlm.nih.gov/gene/?term=152)(122.778) [CALY](http://www.ncbi.nlm.nih.gov/gene/?term=50632)(122.778)[DRD3](http://www.ncbi.nlm.nih.gov/gene/?term=1814)(122.778)[DRD5](http://www.ncbi.nlm.nih.gov/gene/?term=1816)(122.778)[DRD1](http://www.ncbi.nlm.nih.gov/gene/?term=1812)(122.778)[ADRA2B](http://www.ncbi.nlm.nih.gov/gene/?term=151)(122.778) [ESR2](http://www.ncbi.nlm.nih.gov/gene/?term=2100)(23.000)[OPRD1](http://www.ncbi.nlm.nih.gov/gene/?term=4985)(23.000)[ESR1](http://www.ncbi.nlm.nih.gov/gene/?term=2099)(23.000)[OPRK1](http://www.ncbi.nlm.nih.gov/gene/?term=4986)(23.000)[OPRM1](http://www.ncbi.nlm.nih.gov/gene/?term=4988)(23.000) |
| [3,4,5-Trihydroxybenzoic Acid](http://www.megabionet.org/tcmid/ingredient/23210/) | [AKR1C1](http://www.ncbi.nlm.nih.gov/gene/?term=1645)(48.000)[PTGS1](http://www.ncbi.nlm.nih.gov/gene/?term=5742)(48.000)[PTGS2](http://www.ncbi.nlm.nih.gov/gene/?term=5743)(48.000)[PLA2G2E](http://www.ncbi.nlm.nih.gov/gene/?term=30814)(23.000)[AKR1C2](http://www.ncbi.nlm.nih.gov/gene/?term=1646)(23.000) [PPARG](http://www.ncbi.nlm.nih.gov/gene/?term=5468)(23.000)[MPO](http://www.ncbi.nlm.nih.gov/gene/?term=4353)(23.000)[DDC](http://www.ncbi.nlm.nih.gov/gene/?term=1644)(23.000)[ALOX5](http://www.ncbi.nlm.nih.gov/gene/?term=240)(23.000)[IKBKB](http://www.ncbi.nlm.nih.gov/gene/?term=3551)(23.000) [CHUK](http://www.ncbi.nlm.nih.gov/gene/?term=1147)(23.000) |
| [Sennoside](http://www.megabionet.org/tcmid/ingredient/24877/) | This compound doesn't have any potential target with score larger than 20. |
| [Nerol](http://www.megabionet.org/tcmid/ingredient/15489/) | [RDH11](http://www.ncbi.nlm.nih.gov/gene/?term=51109)(80.882)[RBP3](http://www.ncbi.nlm.nih.gov/gene/?term=5949)(80.882)[RETSAT](http://www.ncbi.nlm.nih.gov/gene/?term=54884)(80.882)[RDH13](http://www.ncbi.nlm.nih.gov/gene/?term=112724)(80.882)[DHRS3](http://www.ncbi.nlm.nih.gov/gene/?term=9249)(80.882) [RDH5](http://www.ncbi.nlm.nih.gov/gene/?term=5959)(80.882)[ALDH1A3](http://www.ncbi.nlm.nih.gov/gene/?term=220)(80.882)[RDH12](http://www.ncbi.nlm.nih.gov/gene/?term=145226)(80.882)[DHRS4](http://www.ncbi.nlm.nih.gov/gene/?term=10901)(80.882)[RBP1](http://www.ncbi.nlm.nih.gov/gene/?term=5947)(80.882) [ALDH1A1](http://www.ncbi.nlm.nih.gov/gene/?term=216)(80.882)[RLBP1](http://www.ncbi.nlm.nih.gov/gene/?term=6017)(80.882)[RDH14](http://www.ncbi.nlm.nih.gov/gene/?term=57665)(80.882)[RDH8](http://www.ncbi.nlm.nih.gov/gene/?term=50700)(80.882)[LRAT](http://www.ncbi.nlm.nih.gov/gene/?term=9227)(80.882) [ALDH1A2](http://www.ncbi.nlm.nih.gov/gene/?term=8854)(80.882)[ALDH3A1](http://www.ncbi.nlm.nih.gov/gene/?term=218)(55.444)[RHO](http://www.ncbi.nlm.nih.gov/gene/?term=6010)(55.444)[RS1](http://www.ncbi.nlm.nih.gov/gene/?term=6247)(55.444)[CTSH](http://www.ncbi.nlm.nih.gov/gene/?term=1512)(22.373) [OPN4](http://www.ncbi.nlm.nih.gov/gene/?term=94233)(22.373)[RDH10](http://www.ncbi.nlm.nih.gov/gene/?term=157506)(22.373)[ALDH8A1](http://www.ncbi.nlm.nih.gov/gene/?term=64577)(22.373)[SHBG](http://www.ncbi.nlm.nih.gov/gene/?term=6462)(22.373)[DHRS2](http://www.ncbi.nlm.nih.gov/gene/?term=10202)(22.373) [HSD17B7](http://www.ncbi.nlm.nih.gov/gene/?term=51478)(22.373)[AR](http://www.ncbi.nlm.nih.gov/gene/?term=367)(22.373)[THRA](http://www.ncbi.nlm.nih.gov/gene/?term=7067)(22.373)[PAX2](http://www.ncbi.nlm.nih.gov/gene/?term=5076)(22.373)[CBR3](http://www.ncbi.nlm.nih.gov/gene/?term=874)(22.373) [GATA3](http://www.ncbi.nlm.nih.gov/gene/?term=2625)(22.373)[ASCL1](http://www.ncbi.nlm.nih.gov/gene/?term=429)(22.373)[DLX5](http://www.ncbi.nlm.nih.gov/gene/?term=1749)(22.373)[RET](http://www.ncbi.nlm.nih.gov/gene/?term=5979)(22.373)[THRB](http://www.ncbi.nlm.nih.gov/gene/?term=7068)(22.373) [CRYM](http://www.ncbi.nlm.nih.gov/gene/?term=1428)(22.373)[TSPO](http://www.ncbi.nlm.nih.gov/gene/?term=706)(22.373)[OPN5](http://www.ncbi.nlm.nih.gov/gene/?term=221391)(22.373)[DHRS9](http://www.ncbi.nlm.nih.gov/gene/?term=10170)(22.373)[CBR1](http://www.ncbi.nlm.nih.gov/gene/?term=873)(22.373) [CYP1A1](http://www.ncbi.nlm.nih.gov/gene/?term=1543)(22.373) |
| [Rheidin B](http://www.megabionet.org/tcmid/ingredient/18742/) | [PPARG](http://www.ncbi.nlm.nih.gov/gene/?term=5468)(23.000)[FPGS](http://www.ncbi.nlm.nih.gov/gene/?term=2356)(23.000)[PTGS1](http://www.ncbi.nlm.nih.gov/gene/?term=5742)(23.000)[PTGS2](http://www.ncbi.nlm.nih.gov/gene/?term=5743)(23.000)[ALOX5](http://www.ncbi.nlm.nih.gov/gene/?term=240)(23.000) [MPL](http://www.ncbi.nlm.nih.gov/gene/?term=4352)(23.000)[TYMS](http://www.ncbi.nlm.nih.gov/gene/?term=7298)(23.000)[APOA2](http://www.ncbi.nlm.nih.gov/gene/?term=336)(22.373)[SHMT1](http://www.ncbi.nlm.nih.gov/gene/?term=6470)(22.373)[SERPINE1](http://www.ncbi.nlm.nih.gov/gene/?term=5054)(22.373) [IL1B](http://www.ncbi.nlm.nih.gov/gene/?term=3553)(22.373)[IL13](http://www.ncbi.nlm.nih.gov/gene/?term=3596)(22.373)[GGH](http://www.ncbi.nlm.nih.gov/gene/?term=8836)(22.373)[SERPINB7](http://www.ncbi.nlm.nih.gov/gene/?term=8710)(22.373) |
| [Isoeugenol](http://www.megabionet.org/tcmid/ingredient/11418/) | [CNR2](http://www.ncbi.nlm.nih.gov/gene/?term=1269)(23.000)[TYR](http://www.ncbi.nlm.nih.gov/gene/?term=7299)(23.000)[CNR1](http://www.ncbi.nlm.nih.gov/gene/?term=1268)(23.000) |
| [Oleanolic Acid](http://www.megabionet.org/tcmid/ingredient/23101/) | [AR](http://www.ncbi.nlm.nih.gov/gene/?term=367)(122.778)[SRD5A1](http://www.ncbi.nlm.nih.gov/gene/?term=6715)(48.000)[CYP17A1](http://www.ncbi.nlm.nih.gov/gene/?term=1586)(48.000)[SLC8A1](http://www.ncbi.nlm.nih.gov/gene/?term=6546)(48.000)[F12](http://www.ncbi.nlm.nih.gov/gene/?term=2161)(48.000) [PRLR](http://www.ncbi.nlm.nih.gov/gene/?term=5618)(48.000)[ESR1](http://www.ncbi.nlm.nih.gov/gene/?term=2099)(48.000)[TRPV1](http://www.ncbi.nlm.nih.gov/gene/?term=7442)(48.000)[PPARG](http://www.ncbi.nlm.nih.gov/gene/?term=5468)(48.000)[PTGER1](http://www.ncbi.nlm.nih.gov/gene/?term=5731)(48.000) [FFAR1](http://www.ncbi.nlm.nih.gov/gene/?term=2864)(48.000)[PTGER4](http://www.ncbi.nlm.nih.gov/gene/?term=5734)(48.000)[PGR](http://www.ncbi.nlm.nih.gov/gene/?term=5241)(48.000)[NR3C1](http://www.ncbi.nlm.nih.gov/gene/?term=2908)(48.000)[OPRK1](http://www.ncbi.nlm.nih.gov/gene/?term=4986)(48.000) [FADS2](http://www.ncbi.nlm.nih.gov/gene/?term=9415)(48.000)[FADS1](http://www.ncbi.nlm.nih.gov/gene/?term=3992)(48.000)[PPARD](http://www.ncbi.nlm.nih.gov/gene/?term=5467)(48.000)[PTGER2](http://www.ncbi.nlm.nih.gov/gene/?term=5732)(48.000)[PTGS1](http://www.ncbi.nlm.nih.gov/gene/?term=5742)(48.000) [ACSL4](http://www.ncbi.nlm.nih.gov/gene/?term=2182)(48.000)[PTGER3](http://www.ncbi.nlm.nih.gov/gene/?term=5733)(48.000)[PTGS2](http://www.ncbi.nlm.nih.gov/gene/?term=5743)(48.000)[ELOVL4](http://www.ncbi.nlm.nih.gov/gene/?term=6785)(48.000)[ANXA1](http://www.ncbi.nlm.nih.gov/gene/?term=301)(48.000) [ACSL3](http://www.ncbi.nlm.nih.gov/gene/?term=2181)(48.000)[NR3C2](http://www.ncbi.nlm.nih.gov/gene/?term=4306)(48.000)[ESRRG](http://www.ncbi.nlm.nih.gov/gene/?term=2104)(23.000)[COX6C](http://www.ncbi.nlm.nih.gov/gene/?term=1345)(23.000)[COX5B](http://www.ncbi.nlm.nih.gov/gene/?term=1329)(23.000) [COX7C](http://www.ncbi.nlm.nih.gov/gene/?term=1350)(23.000)[COX1](http://www.ncbi.nlm.nih.gov/gene/?term=4512)(23.000)[AKR1C2](http://www.ncbi.nlm.nih.gov/gene/?term=1646)(23.000)[ADH1B](http://www.ncbi.nlm.nih.gov/gene/?term=125)(23.000)[COX5A](http://www.ncbi.nlm.nih.gov/gene/?term=9377)(23.000) [COX3](http://www.ncbi.nlm.nih.gov/gene/?term=4514)(23.000)[COX7A1](http://www.ncbi.nlm.nih.gov/gene/?term=1346)(23.000)[FECH](http://www.ncbi.nlm.nih.gov/gene/?term=2235)(23.000)[COX4I1](http://www.ncbi.nlm.nih.gov/gene/?term=1327)(23.000)[PLA2G1B](http://www.ncbi.nlm.nih.gov/gene/?term=5319)(23.000) [AKR1C1](http://www.ncbi.nlm.nih.gov/gene/?term=1645)(23.000)[COX6A2](http://www.ncbi.nlm.nih.gov/gene/?term=1339)(23.000)[HMGCR](http://www.ncbi.nlm.nih.gov/gene/?term=3156)(23.000)[ADH1C](http://www.ncbi.nlm.nih.gov/gene/?term=126)(23.000)[COX6B1](http://www.ncbi.nlm.nih.gov/gene/?term=1340)(23.000) [FABP6](http://www.ncbi.nlm.nih.gov/gene/?term=2172)(23.000)[CES1](http://www.ncbi.nlm.nih.gov/gene/?term=1066)(23.000)[COX7B](http://www.ncbi.nlm.nih.gov/gene/?term=1349)(23.000)[COX2](http://www.ncbi.nlm.nih.gov/gene/?term=4513)(23.000)[COX8A](http://www.ncbi.nlm.nih.gov/gene/?term=1351)(23.000) [NR1H4](http://www.ncbi.nlm.nih.gov/gene/?term=9971)(23.000) |
| [Tirucallol](http://www.megabionet.org/tcmid/ingredient/21393/) | [ESR1](http://www.ncbi.nlm.nih.gov/gene/?term=2099)(122.778)[PGR](http://www.ncbi.nlm.nih.gov/gene/?term=5241)(122.778)[VDR](http://www.ncbi.nlm.nih.gov/gene/?term=7421)(80.882)[CYP27B1](http://www.ncbi.nlm.nih.gov/gene/?term=1594)(80.882)[GC](http://www.ncbi.nlm.nih.gov/gene/?term=2638)(55.444) [SNW1](http://www.ncbi.nlm.nih.gov/gene/?term=22938)(55.444)[SRD5A1](http://www.ncbi.nlm.nih.gov/gene/?term=6715)(48.000)[AR](http://www.ncbi.nlm.nih.gov/gene/?term=367)(48.000)[NR3C1](http://www.ncbi.nlm.nih.gov/gene/?term=2908)(48.000)[ANXA1](http://www.ncbi.nlm.nih.gov/gene/?term=301)(48.000) [NFKB1](http://www.ncbi.nlm.nih.gov/gene/?term=4790)(22.373)[AKR1C3](http://www.ncbi.nlm.nih.gov/gene/?term=8644)(22.373)[CYP24A1](http://www.ncbi.nlm.nih.gov/gene/?term=1591)(22.373)[GPBAR1](http://www.ncbi.nlm.nih.gov/gene/?term=151306)(22.373)[SNAI2](http://www.ncbi.nlm.nih.gov/gene/?term=6591)(22.373) [MED1](http://www.ncbi.nlm.nih.gov/gene/?term=5469)(22.373)[SNAI1](http://www.ncbi.nlm.nih.gov/gene/?term=6615)(22.373)[CYP3A4](http://www.ncbi.nlm.nih.gov/gene/?term=1576)(22.373)[CALB1](http://www.ncbi.nlm.nih.gov/gene/?term=793)(22.373)[FGF23](http://www.ncbi.nlm.nih.gov/gene/?term=8074)(22.373) [GFI1](http://www.ncbi.nlm.nih.gov/gene/?term=2672)(22.373)[LANCL2](http://www.ncbi.nlm.nih.gov/gene/?term=55915)(22.373)[WNT4](http://www.ncbi.nlm.nih.gov/gene/?term=54361)(22.373)[TCF3](http://www.ncbi.nlm.nih.gov/gene/?term=6929)(22.373)[BAX](http://www.ncbi.nlm.nih.gov/gene/?term=581)(22.373) [KL](http://www.ncbi.nlm.nih.gov/gene/?term=9365)(22.373)[CYP2R1](http://www.ncbi.nlm.nih.gov/gene/?term=120227)(22.373)[PML](http://www.ncbi.nlm.nih.gov/gene/?term=5371)(22.373)[B4GALT1](http://www.ncbi.nlm.nih.gov/gene/?term=2683)(22.373)[S100G](http://www.ncbi.nlm.nih.gov/gene/?term=795)(22.373) [CYP27A1](http://www.ncbi.nlm.nih.gov/gene/?term=1593)(22.373)[KANK2](http://www.ncbi.nlm.nih.gov/gene/?term=25959)(22.373)[IRX5](http://www.ncbi.nlm.nih.gov/gene/?term=10265)(22.373)[RXRA](http://www.ncbi.nlm.nih.gov/gene/?term=6256)(22.373)[NR1H4](http://www.ncbi.nlm.nih.gov/gene/?term=9971)(22.373) [TRIM24](http://www.ncbi.nlm.nih.gov/gene/?term=8805)(22.373) |
| [Hydroginkgolinic Acid](http://www.megabionet.org/tcmid/ingredient/31132/) | [PLA2G2E](http://www.ncbi.nlm.nih.gov/gene/?term=30814)(80.882)[AKR1C2](http://www.ncbi.nlm.nih.gov/gene/?term=1646)(80.882)[PPARG](http://www.ncbi.nlm.nih.gov/gene/?term=5468)(80.882)[MPO](http://www.ncbi.nlm.nih.gov/gene/?term=4353)(80.882)[AKR1C1](http://www.ncbi.nlm.nih.gov/gene/?term=1645)(80.882) [PTGS1](http://www.ncbi.nlm.nih.gov/gene/?term=5742)(80.882)[PTGS2](http://www.ncbi.nlm.nih.gov/gene/?term=5743)(80.882)[ALOX5](http://www.ncbi.nlm.nih.gov/gene/?term=240)(80.882)[IKBKB](http://www.ncbi.nlm.nih.gov/gene/?term=3551)(80.882)[CHUK](http://www.ncbi.nlm.nih.gov/gene/?term=1147)(80.882) [APOA2](http://www.ncbi.nlm.nih.gov/gene/?term=336)(55.444)[HSD17B2](http://www.ncbi.nlm.nih.gov/gene/?term=3294)(55.444)[SERPINE1](http://www.ncbi.nlm.nih.gov/gene/?term=5054)(55.444)[IL1B](http://www.ncbi.nlm.nih.gov/gene/?term=3553)(55.444)[IL13](http://www.ncbi.nlm.nih.gov/gene/?term=3596)(55.444) [SERPINB7](http://www.ncbi.nlm.nih.gov/gene/?term=8710)(55.444)[AKR1C3](http://www.ncbi.nlm.nih.gov/gene/?term=8644)(23.000)[IFNG](http://www.ncbi.nlm.nih.gov/gene/?term=3458)(23.000)[AKR1C4](http://www.ncbi.nlm.nih.gov/gene/?term=1109)(23.000)[TPMT](http://www.ncbi.nlm.nih.gov/gene/?term=7172)(23.000) [NDRG2](http://www.ncbi.nlm.nih.gov/gene/?term=57447)(22.373)[NAMPT](http://www.ncbi.nlm.nih.gov/gene/?term=10135)(22.373)[RGCC](http://www.ncbi.nlm.nih.gov/gene/?term=28984)(22.373)[FNDC5](http://www.ncbi.nlm.nih.gov/gene/?term=252995)(22.373)[NR1H3](http://www.ncbi.nlm.nih.gov/gene/?term=10062)(22.373) [OSBPL8](http://www.ncbi.nlm.nih.gov/gene/?term=114882)(22.373)[AVPR1A](http://www.ncbi.nlm.nih.gov/gene/?term=552)(22.373)[IKBKE](http://www.ncbi.nlm.nih.gov/gene/?term=9641)(22.373)[ITGB3](http://www.ncbi.nlm.nih.gov/gene/?term=3690)(22.373)[METRNL](http://www.ncbi.nlm.nih.gov/gene/?term=284207)(22.373) [MAPK9](http://www.ncbi.nlm.nih.gov/gene/?term=5601)(22.373)[NCKAP1L](http://www.ncbi.nlm.nih.gov/gene/?term=3071)(22.373)[STX3](http://www.ncbi.nlm.nih.gov/gene/?term=6809)(22.373)[OXER1](http://www.ncbi.nlm.nih.gov/gene/?term=165140)(22.373)[PNPLA2](http://www.ncbi.nlm.nih.gov/gene/?term=57104)(22.373) [TNF](http://www.ncbi.nlm.nih.gov/gene/?term=7124)(22.373)[PTGIS](http://www.ncbi.nlm.nih.gov/gene/?term=5740)(22.373)[HEG1](http://www.ncbi.nlm.nih.gov/gene/?term=57493)(22.373)[ITGAV](http://www.ncbi.nlm.nih.gov/gene/?term=3685)(22.373)[PTPN2](http://www.ncbi.nlm.nih.gov/gene/?term=5771)(22.373) [S100A8](http://www.ncbi.nlm.nih.gov/gene/?term=6279)(22.373)[ALOX5AP](http://www.ncbi.nlm.nih.gov/gene/?term=241)(22.373)[AVP](http://www.ncbi.nlm.nih.gov/gene/?term=551)(22.373)[SLC6A4](http://www.ncbi.nlm.nih.gov/gene/?term=6532)(22.373)[ROCK2](http://www.ncbi.nlm.nih.gov/gene/?term=9475)(22.373) [BDKRB2](http://www.ncbi.nlm.nih.gov/gene/?term=624)(22.373)[NR1H2](http://www.ncbi.nlm.nih.gov/gene/?term=7376)(22.373)[NEUROD2](http://www.ncbi.nlm.nih.gov/gene/?term=4761)(22.373)[INS](http://www.ncbi.nlm.nih.gov/gene/?term=3630)(22.373)[ANAPC2](http://www.ncbi.nlm.nih.gov/gene/?term=29882)(22.373) [WNT11](http://www.ncbi.nlm.nih.gov/gene/?term=7481)(22.373)[ALOX15B](http://www.ncbi.nlm.nih.gov/gene/?term=247)(22.373)[DHDH](http://www.ncbi.nlm.nih.gov/gene/?term=27294)(22.373)[CDC20](http://www.ncbi.nlm.nih.gov/gene/?term=991)(22.373)[DNAJA3](http://www.ncbi.nlm.nih.gov/gene/?term=9093)(22.373) [ROCK1](http://www.ncbi.nlm.nih.gov/gene/?term=6093)(22.373)[COL1A1](http://www.ncbi.nlm.nih.gov/gene/?term=1277)(22.373)[NR1D1](http://www.ncbi.nlm.nih.gov/gene/?term=9572)(22.373)[ABCA1](http://www.ncbi.nlm.nih.gov/gene/?term=19)(22.373)[NAPRT](http://www.ncbi.nlm.nih.gov/gene/?term=93100)(22.373) [MECOM](http://www.ncbi.nlm.nih.gov/gene/?term=2122)(22.373)[ABHD5](http://www.ncbi.nlm.nih.gov/gene/?term=51099)(22.373)[SNCA](http://www.ncbi.nlm.nih.gov/gene/?term=6622)(22.373)[ADIPOQ](http://www.ncbi.nlm.nih.gov/gene/?term=9370)(22.373)[CPLX2](http://www.ncbi.nlm.nih.gov/gene/?term=10814)(22.373) [PPARA](http://www.ncbi.nlm.nih.gov/gene/?term=5465)(22.373)[ALOX15](http://www.ncbi.nlm.nih.gov/gene/?term=246)(22.373)[ANXA1](http://www.ncbi.nlm.nih.gov/gene/?term=301)(22.373)[PAWR](http://www.ncbi.nlm.nih.gov/gene/?term=5074)(22.373)[AKR1B10](http://www.ncbi.nlm.nih.gov/gene/?term=57016)(22.373) [S100A9](http://www.ncbi.nlm.nih.gov/gene/?term=6280)(22.373)[FABP3](http://www.ncbi.nlm.nih.gov/gene/?term=2170)(22.373)[ABCG1](http://www.ncbi.nlm.nih.gov/gene/?term=9619)(22.373)[PRDM16](http://www.ncbi.nlm.nih.gov/gene/?term=63976)(22.373)[CCM2L](http://www.ncbi.nlm.nih.gov/gene/?term=140706)(22.373) |
| [Canaline](http://www.megabionet.org/tcmid/ingredient/3059/) | [OAT](http://www.ncbi.nlm.nih.gov/gene/?term=4942)(known target in DrugBank)[GOT2](http://www.ncbi.nlm.nih.gov/gene/?term=2806)(48.000)[SLC1A1](http://www.ncbi.nlm.nih.gov/gene/?term=6505)(48.000)[ACY3](http://www.ncbi.nlm.nih.gov/gene/?term=91703)(48.000)[ASPA](http://www.ncbi.nlm.nih.gov/gene/?term=443)(48.000) [BCAT1](http://www.ncbi.nlm.nih.gov/gene/?term=586)(48.000)[VARS](http://www.ncbi.nlm.nih.gov/gene/?term=7407)(48.000)[ADSSL1](http://www.ncbi.nlm.nih.gov/gene/?term=122622)(48.000)[ASNS](http://www.ncbi.nlm.nih.gov/gene/?term=440)(48.000)[DARS2](http://www.ncbi.nlm.nih.gov/gene/?term=55157)(48.000) [GOT1](http://www.ncbi.nlm.nih.gov/gene/?term=2805)(48.000)[ASPH](http://www.ncbi.nlm.nih.gov/gene/?term=444)(48.000)[SLC25A12](http://www.ncbi.nlm.nih.gov/gene/?term=8604)(48.000)[DARS](http://www.ncbi.nlm.nih.gov/gene/?term=1615)(48.000)[ASRGL1](http://www.ncbi.nlm.nih.gov/gene/?term=80150)(48.000) [RNASE1](http://www.ncbi.nlm.nih.gov/gene/?term=6035)(48.000)[ASS1](http://www.ncbi.nlm.nih.gov/gene/?term=445)(48.000)[ADSS](http://www.ncbi.nlm.nih.gov/gene/?term=159)(48.000)[CAD](http://www.ncbi.nlm.nih.gov/gene/?term=790)(48.000)[PCCB](http://www.ncbi.nlm.nih.gov/gene/?term=5096)(48.000) [ACY1](http://www.ncbi.nlm.nih.gov/gene/?term=95)(48.000)[SLC25A13](http://www.ncbi.nlm.nih.gov/gene/?term=10165)(48.000)[LYZ](http://www.ncbi.nlm.nih.gov/gene/?term=4069)(48.000)[PAICS](http://www.ncbi.nlm.nih.gov/gene/?term=10606)(48.000)[SLC1A5](http://www.ncbi.nlm.nih.gov/gene/?term=6510)(23.000) [GPT2](http://www.ncbi.nlm.nih.gov/gene/?term=84706)(23.000)[BAAT](http://www.ncbi.nlm.nih.gov/gene/?term=570)(23.000)[OAZ3](http://www.ncbi.nlm.nih.gov/gene/?term=51686)(23.000)[SHMT1](http://www.ncbi.nlm.nih.gov/gene/?term=6470)(23.000)[ABAT](http://www.ncbi.nlm.nih.gov/gene/?term=18)(23.000) [SLC7A2](http://www.ncbi.nlm.nih.gov/gene/?term=6542)(23.000)[GCSH](http://www.ncbi.nlm.nih.gov/gene/?term=2653)(23.000)[SLC7A8](http://www.ncbi.nlm.nih.gov/gene/?term=23428)(23.000)[PPAT](http://www.ncbi.nlm.nih.gov/gene/?term=5471)(23.000)[IARS2](http://www.ncbi.nlm.nih.gov/gene/?term=55699)(23.000) [GLRA2](http://www.ncbi.nlm.nih.gov/gene/?term=2742)(23.000)[ALAS1](http://www.ncbi.nlm.nih.gov/gene/?term=211)(23.000)[GCAT](http://www.ncbi.nlm.nih.gov/gene/?term=23464)(23.000)[SLC38A3](http://www.ncbi.nlm.nih.gov/gene/?term=10991)(23.000)[NFS1](http://www.ncbi.nlm.nih.gov/gene/?term=9054)(23.000) [SLC25A15](http://www.ncbi.nlm.nih.gov/gene/?term=10166)(23.000)[SLC1A4](http://www.ncbi.nlm.nih.gov/gene/?term=6509)(23.000)[VDAC3](http://www.ncbi.nlm.nih.gov/gene/?term=7419)(23.000)[GSS](http://www.ncbi.nlm.nih.gov/gene/?term=2937)(23.000)[GLRA3](http://www.ncbi.nlm.nih.gov/gene/?term=8001)(23.000) [GRIN3B](http://www.ncbi.nlm.nih.gov/gene/?term=116444)(23.000)[LCMT1](http://www.ncbi.nlm.nih.gov/gene/?term=51451)(23.000)[GRIN2A](http://www.ncbi.nlm.nih.gov/gene/?term=2903)(23.000)[GNMT](http://www.ncbi.nlm.nih.gov/gene/?term=27232)(23.000)[GLRB](http://www.ncbi.nlm.nih.gov/gene/?term=2743)(23.000) [GLYAT](http://www.ncbi.nlm.nih.gov/gene/?term=10249)(23.000)[AARS2](http://www.ncbi.nlm.nih.gov/gene/?term=57505)(23.000)[GPR18](http://www.ncbi.nlm.nih.gov/gene/?term=2841)(23.000)[CTPS1](http://www.ncbi.nlm.nih.gov/gene/?term=1503)(23.000)[PHYKPL](http://www.ncbi.nlm.nih.gov/gene/?term=85007)(23.000) [GLYATL1](http://www.ncbi.nlm.nih.gov/gene/?term=92292)(23.000)[GATM](http://www.ncbi.nlm.nih.gov/gene/?term=2628)(23.000)[LARS](http://www.ncbi.nlm.nih.gov/gene/?term=51520)(23.000)[ARG1](http://www.ncbi.nlm.nih.gov/gene/?term=383)(23.000)[NARS2](http://www.ncbi.nlm.nih.gov/gene/?term=79731)(23.000) [TNNC1](http://www.ncbi.nlm.nih.gov/gene/?term=7134)(23.000)[GRIN2C](http://www.ncbi.nlm.nih.gov/gene/?term=2905)(23.000)[GLDC](http://www.ncbi.nlm.nih.gov/gene/?term=2731)(23.000)[VDAC2](http://www.ncbi.nlm.nih.gov/gene/?term=7417)(23.000)[OAZ2](http://www.ncbi.nlm.nih.gov/gene/?term=4947)(23.000) [PIPOX](http://www.ncbi.nlm.nih.gov/gene/?term=51268)(23.000)[SLC25A2](http://www.ncbi.nlm.nih.gov/gene/?term=83884)(23.000)[GARS](http://www.ncbi.nlm.nih.gov/gene/?term=2617)(23.000)[GPT](http://www.ncbi.nlm.nih.gov/gene/?term=2875)(23.000)[AARS](http://www.ncbi.nlm.nih.gov/gene/?term=16)(23.000) [SHMT2](http://www.ncbi.nlm.nih.gov/gene/?term=6472)(23.000)[ALAS2](http://www.ncbi.nlm.nih.gov/gene/?term=212)(23.000)[VDAC1](http://www.ncbi.nlm.nih.gov/gene/?term=7416)(23.000)[SLC6A9](http://www.ncbi.nlm.nih.gov/gene/?term=6536)(23.000)[SLC7A3](http://www.ncbi.nlm.nih.gov/gene/?term=84889)(23.000) [GLYATL2](http://www.ncbi.nlm.nih.gov/gene/?term=219970)(23.000)[SLC32A1](http://www.ncbi.nlm.nih.gov/gene/?term=140679)(23.000)[OAZ1](http://www.ncbi.nlm.nih.gov/gene/?term=4946)(23.000)[KARS](http://www.ncbi.nlm.nih.gov/gene/?term=3735)(23.000)[AGXT](http://www.ncbi.nlm.nih.gov/gene/?term=189)(23.000) [LARS2](http://www.ncbi.nlm.nih.gov/gene/?term=23395)(23.000)[SLC7A1](http://www.ncbi.nlm.nih.gov/gene/?term=6541)(23.000)[SLC36A1](http://www.ncbi.nlm.nih.gov/gene/?term=206358)(23.000)[ACADSB](http://www.ncbi.nlm.nih.gov/gene/?term=36)(23.000)[SLC6A5](http://www.ncbi.nlm.nih.gov/gene/?term=9152)(23.000) [SLC7A4](http://www.ncbi.nlm.nih.gov/gene/?term=6545)(23.000)[OTC](http://www.ncbi.nlm.nih.gov/gene/?term=5009)(23.000)[GLUL](http://www.ncbi.nlm.nih.gov/gene/?term=2752)(23.000)[AGXT2](http://www.ncbi.nlm.nih.gov/gene/?term=64902)(23.000)[ARG2](http://www.ncbi.nlm.nih.gov/gene/?term=384)(23.000) [IARS](http://www.ncbi.nlm.nih.gov/gene/?term=3376)(23.000)[BCAT2](http://www.ncbi.nlm.nih.gov/gene/?term=587)(23.000)[KYNU](http://www.ncbi.nlm.nih.gov/gene/?term=8942)(23.000)[GLRA1](http://www.ncbi.nlm.nih.gov/gene/?term=2741)(23.000)[LCMT2](http://www.ncbi.nlm.nih.gov/gene/?term=9836)(23.000) [NARS](http://www.ncbi.nlm.nih.gov/gene/?term=4677)(23.000) |
| [Dipentene](http://www.megabionet.org/tcmid/ingredient/6481/) | [KCND1](http://www.ncbi.nlm.nih.gov/gene/?term=3750)(80.882)[KCNA3](http://www.ncbi.nlm.nih.gov/gene/?term=3738)(80.882)[PRKAB1](http://www.ncbi.nlm.nih.gov/gene/?term=5564)(80.882)[ADH1A](http://www.ncbi.nlm.nih.gov/gene/?term=124)(80.882)[KCNA10](http://www.ncbi.nlm.nih.gov/gene/?term=3744)(80.882) [GAMT](http://www.ncbi.nlm.nih.gov/gene/?term=2593)(80.882)[KCNC3](http://www.ncbi.nlm.nih.gov/gene/?term=3748)(80.882)[KCNA1](http://www.ncbi.nlm.nih.gov/gene/?term=3736)(80.882)[KCNA2](http://www.ncbi.nlm.nih.gov/gene/?term=3737)(80.882)[TPO](http://www.ncbi.nlm.nih.gov/gene/?term=7173)(80.882) [CAT](http://www.ncbi.nlm.nih.gov/gene/?term=847)(80.882)[KCNB1](http://www.ncbi.nlm.nih.gov/gene/?term=3745)(80.882)[ADH1B](http://www.ncbi.nlm.nih.gov/gene/?term=125)(80.882)[DLG4](http://www.ncbi.nlm.nih.gov/gene/?term=1742)(80.882)[KCNC2](http://www.ncbi.nlm.nih.gov/gene/?term=3747)(80.882) [KCNC1](http://www.ncbi.nlm.nih.gov/gene/?term=3746)(80.882)[ADH1C](http://www.ncbi.nlm.nih.gov/gene/?term=126)(80.882)[KCNB2](http://www.ncbi.nlm.nih.gov/gene/?term=9312)(80.882)[KCNA5](http://www.ncbi.nlm.nih.gov/gene/?term=3741)(80.882)[RNASE1](http://www.ncbi.nlm.nih.gov/gene/?term=6035)(80.882) [KCND2](http://www.ncbi.nlm.nih.gov/gene/?term=3751)(80.882)[ALDH2](http://www.ncbi.nlm.nih.gov/gene/?term=217)(80.882)[KCNA4](http://www.ncbi.nlm.nih.gov/gene/?term=3739)(80.882)[KCNA7](http://www.ncbi.nlm.nih.gov/gene/?term=3743)(80.882)[KCNA6](http://www.ncbi.nlm.nih.gov/gene/?term=3742)(80.882) [KCND3](http://www.ncbi.nlm.nih.gov/gene/?term=3752)(80.882)[GUCY1B3](http://www.ncbi.nlm.nih.gov/gene/?term=2983)(55.444)[GATM](http://www.ncbi.nlm.nih.gov/gene/?term=2628)(55.444)[KCNK4](http://www.ncbi.nlm.nih.gov/gene/?term=50801)(55.444)[IYD](http://www.ncbi.nlm.nih.gov/gene/?term=389434)(55.444) [KCNQ1](http://www.ncbi.nlm.nih.gov/gene/?term=3784)(55.444)[FBP1](http://www.ncbi.nlm.nih.gov/gene/?term=2203)(22.373)[DGKI](http://www.ncbi.nlm.nih.gov/gene/?term=9162)(22.373)[ADH7](http://www.ncbi.nlm.nih.gov/gene/?term=131)(22.373)[NFIB](http://www.ncbi.nlm.nih.gov/gene/?term=4781)(22.373) [CYGB](http://www.ncbi.nlm.nih.gov/gene/?term=114757)(22.373)[NRXN3](http://www.ncbi.nlm.nih.gov/gene/?term=9369)(22.373)[LRRC4B](http://www.ncbi.nlm.nih.gov/gene/?term=94030)(22.373)[AQP8](http://www.ncbi.nlm.nih.gov/gene/?term=343)(22.373)[ARX](http://www.ncbi.nlm.nih.gov/gene/?term=170302)(22.373) [IGF1](http://www.ncbi.nlm.nih.gov/gene/?term=3479)(22.373)[SHANK3](http://www.ncbi.nlm.nih.gov/gene/?term=85358)(22.373)[ACY3](http://www.ncbi.nlm.nih.gov/gene/?term=91703)(22.373)[ASPA](http://www.ncbi.nlm.nih.gov/gene/?term=443)(22.373)[NRXN1](http://www.ncbi.nlm.nih.gov/gene/?term=9378)(22.373) [EDN1](http://www.ncbi.nlm.nih.gov/gene/?term=1906)(22.373)[OXT](http://www.ncbi.nlm.nih.gov/gene/?term=5020)(22.373)[MAGI2](http://www.ncbi.nlm.nih.gov/gene/?term=9863)(22.373)[APOE](http://www.ncbi.nlm.nih.gov/gene/?term=348)(22.373)[SOX15](http://www.ncbi.nlm.nih.gov/gene/?term=6665)(22.373) [CNTNAP4](http://www.ncbi.nlm.nih.gov/gene/?term=85445)(22.373)[RNASE4](http://www.ncbi.nlm.nih.gov/gene/?term=6038)(22.373)[RAB3A](http://www.ncbi.nlm.nih.gov/gene/?term=5864)(22.373)[MC4R](http://www.ncbi.nlm.nih.gov/gene/?term=4160)(22.373)[IL1B](http://www.ncbi.nlm.nih.gov/gene/?term=3553)(22.373) [GRIN2A](http://www.ncbi.nlm.nih.gov/gene/?term=2903)(22.373)[SLC17A7](http://www.ncbi.nlm.nih.gov/gene/?term=57030)(22.373)[CYP11A1](http://www.ncbi.nlm.nih.gov/gene/?term=1583)(22.373)[SCN5A](http://www.ncbi.nlm.nih.gov/gene/?term=6331)(22.373)[SCN10A](http://www.ncbi.nlm.nih.gov/gene/?term=6336)(22.373) [SPARC](http://www.ncbi.nlm.nih.gov/gene/?term=6678)(22.373)[SORCS3](http://www.ncbi.nlm.nih.gov/gene/?term=22986)(22.373)[RNASE8](http://www.ncbi.nlm.nih.gov/gene/?term=122665)(22.373)[KCNIP2](http://www.ncbi.nlm.nih.gov/gene/?term=30819)(22.373)[GPX7](http://www.ncbi.nlm.nih.gov/gene/?term=2882)(22.373) [CRLF1](http://www.ncbi.nlm.nih.gov/gene/?term=9244)(22.373)[FADD](http://www.ncbi.nlm.nih.gov/gene/?term=8772)(22.373)[SNTG2](http://www.ncbi.nlm.nih.gov/gene/?term=54221)(22.373)[SCGB1A1](http://www.ncbi.nlm.nih.gov/gene/?term=7356)(22.373)[OXTR](http://www.ncbi.nlm.nih.gov/gene/?term=5021)(22.373) [ALDH1B1](http://www.ncbi.nlm.nih.gov/gene/?term=219)(22.373)[LEP](http://www.ncbi.nlm.nih.gov/gene/?term=3952)(22.373)[NLGN1](http://www.ncbi.nlm.nih.gov/gene/?term=22871)(22.373)[CACNA1D](http://www.ncbi.nlm.nih.gov/gene/?term=776)(22.373)[RAPGEF2](http://www.ncbi.nlm.nih.gov/gene/?term=9693)(22.373) [DARS](http://www.ncbi.nlm.nih.gov/gene/?term=1615)(22.373)[ANK3](http://www.ncbi.nlm.nih.gov/gene/?term=288)(22.373)[KCNE5](http://www.ncbi.nlm.nih.gov/gene/?term=23630)(22.373)[PPARD](http://www.ncbi.nlm.nih.gov/gene/?term=5467)(22.373)[NKX2-1](http://www.ncbi.nlm.nih.gov/gene/?term=7080)(22.373) [MIP](http://www.ncbi.nlm.nih.gov/gene/?term=4284)(22.373)[RYR3](http://www.ncbi.nlm.nih.gov/gene/?term=6263)(22.373)[ZPR1](http://www.ncbi.nlm.nih.gov/gene/?term=8882)(22.373)[ALDH3B1](http://www.ncbi.nlm.nih.gov/gene/?term=221)(22.373)[NPPA](http://www.ncbi.nlm.nih.gov/gene/?term=4878)(22.373) [STX1A](http://www.ncbi.nlm.nih.gov/gene/?term=6804)(22.373)[PAXBP1](http://www.ncbi.nlm.nih.gov/gene/?term=94104)(22.373)[PAX7](http://www.ncbi.nlm.nih.gov/gene/?term=5081)(22.373)[ALDH3B2](http://www.ncbi.nlm.nih.gov/gene/?term=222)(22.373)[RNASE2](http://www.ncbi.nlm.nih.gov/gene/?term=6036)(22.373) [NRXN2](http://www.ncbi.nlm.nih.gov/gene/?term=9379)(22.373)[ADORA1](http://www.ncbi.nlm.nih.gov/gene/?term=134)(22.373)[ADH4](http://www.ncbi.nlm.nih.gov/gene/?term=127)(22.373)[GJA5](http://www.ncbi.nlm.nih.gov/gene/?term=2702)(22.373)[FAS](http://www.ncbi.nlm.nih.gov/gene/?term=355)(22.373) [DAB2IP](http://www.ncbi.nlm.nih.gov/gene/?term=153090)(22.373)[CYP2E1](http://www.ncbi.nlm.nih.gov/gene/?term=1571)(22.373)[ACY1](http://www.ncbi.nlm.nih.gov/gene/?term=95)(22.373) |
| [Emodin Anthrone](http://www.megabionet.org/tcmid/ingredient/6776/) | [ESR1](http://www.ncbi.nlm.nih.gov/gene/?term=2099)(80.882)[HSD17B1](http://www.ncbi.nlm.nih.gov/gene/?term=3292)(23.000)[TFAP2C](http://www.ncbi.nlm.nih.gov/gene/?term=7022)(22.373)[TOX3](http://www.ncbi.nlm.nih.gov/gene/?term=27324)(22.373)[TGFB1](http://www.ncbi.nlm.nih.gov/gene/?term=7040)(22.373) [MED1](http://www.ncbi.nlm.nih.gov/gene/?term=5469)(22.373)[NKX3-1](http://www.ncbi.nlm.nih.gov/gene/?term=4824)(22.373)[ESR2](http://www.ncbi.nlm.nih.gov/gene/?term=2100)(22.373)[SHH](http://www.ncbi.nlm.nih.gov/gene/?term=6469)(22.373)[LEF1](http://www.ncbi.nlm.nih.gov/gene/?term=51176)(22.373) [FGFR2](http://www.ncbi.nlm.nih.gov/gene/?term=2263)(22.373)[PGR](http://www.ncbi.nlm.nih.gov/gene/?term=5241)(22.373)[WNT4](http://www.ncbi.nlm.nih.gov/gene/?term=54361)(22.373)[AREG](http://www.ncbi.nlm.nih.gov/gene/?term=374)(22.373)[GPER1](http://www.ncbi.nlm.nih.gov/gene/?term=2852)(22.373) [WNT5A](http://www.ncbi.nlm.nih.gov/gene/?term=7474)(22.373)[SOX9](http://www.ncbi.nlm.nih.gov/gene/?term=6662)(22.373)[VDR](http://www.ncbi.nlm.nih.gov/gene/?term=7421)(22.373)[TRIM24](http://www.ncbi.nlm.nih.gov/gene/?term=8805)(22.373) |
| [Benzyl Acetate](http://www.megabionet.org/tcmid/ingredient/2273/) | [ACHE](http://www.ncbi.nlm.nih.gov/gene/?term=43)(80.882)[CHRNA2](http://www.ncbi.nlm.nih.gov/gene/?term=1135)(80.882)[CHRM2](http://www.ncbi.nlm.nih.gov/gene/?term=1129)(80.882)[COLQ](http://www.ncbi.nlm.nih.gov/gene/?term=8292)(55.444)[BCHE](http://www.ncbi.nlm.nih.gov/gene/?term=590)(55.444) [SLC5A7](http://www.ncbi.nlm.nih.gov/gene/?term=60482)(22.373)[NRG1](http://www.ncbi.nlm.nih.gov/gene/?term=3084)(22.373)[ARFGEF2](http://www.ncbi.nlm.nih.gov/gene/?term=10564)(22.373)[CHRM3](http://www.ncbi.nlm.nih.gov/gene/?term=1131)(22.373)[CHRM1](http://www.ncbi.nlm.nih.gov/gene/?term=1128)(22.373) [NTSR1](http://www.ncbi.nlm.nih.gov/gene/?term=4923)(22.373)[PRSS12](http://www.ncbi.nlm.nih.gov/gene/?term=8492)(22.373)[SLC44A4](http://www.ncbi.nlm.nih.gov/gene/?term=80736)(22.373)[DNM3](http://www.ncbi.nlm.nih.gov/gene/?term=26052)(22.373)[CHRM5](http://www.ncbi.nlm.nih.gov/gene/?term=1133)(22.373) [CDH8](http://www.ncbi.nlm.nih.gov/gene/?term=1006)(22.373)[FNTA](http://www.ncbi.nlm.nih.gov/gene/?term=2339)(22.373)[GNA15](http://www.ncbi.nlm.nih.gov/gene/?term=2769)(22.373)[AGRN](http://www.ncbi.nlm.nih.gov/gene/?term=375790)(22.373)[CHKA](http://www.ncbi.nlm.nih.gov/gene/?term=1119)(22.373) [CHRM4](http://www.ncbi.nlm.nih.gov/gene/?term=1132)(22.373)[GRIN1](http://www.ncbi.nlm.nih.gov/gene/?term=2902)(22.373) |
| [Procyanidin B-5,3'-O-Gallate](http://www.megabionet.org/tcmid/ingredient/31843/) | [SOAT1](http://www.ncbi.nlm.nih.gov/gene/?term=6646)(48.000)[MTTP](http://www.ncbi.nlm.nih.gov/gene/?term=4547)(48.000)[SOAT2](http://www.ncbi.nlm.nih.gov/gene/?term=8435)(48.000) |
| [Isoamylamine](http://www.megabionet.org/tcmid/ingredient/11216/) | [GRIN2A](http://www.ncbi.nlm.nih.gov/gene/?term=2903)(80.882)[DRD2](http://www.ncbi.nlm.nih.gov/gene/?term=1813)(80.882)[GRIN2B](http://www.ncbi.nlm.nih.gov/gene/?term=2904)(80.882)[GRIN3A](http://www.ncbi.nlm.nih.gov/gene/?term=116443)(80.882)[HTR3A](http://www.ncbi.nlm.nih.gov/gene/?term=3359)(80.882) [GRIN1](http://www.ncbi.nlm.nih.gov/gene/?term=2902)(80.882)[CNR1](http://www.ncbi.nlm.nih.gov/gene/?term=1268)(55.444)[EPHX2](http://www.ncbi.nlm.nih.gov/gene/?term=2053)(55.444)[CD34](http://www.ncbi.nlm.nih.gov/gene/?term=947)(55.444)[FBP1](http://www.ncbi.nlm.nih.gov/gene/?term=2203)(22.373) [CDK5R2](http://www.ncbi.nlm.nih.gov/gene/?term=8941)(22.373)[GNB5](http://www.ncbi.nlm.nih.gov/gene/?term=10681)(22.373)[FEZF2](http://www.ncbi.nlm.nih.gov/gene/?term=55079)(22.373)[ARPIN](http://www.ncbi.nlm.nih.gov/gene/?term=348110)(22.373)[ARX](http://www.ncbi.nlm.nih.gov/gene/?term=170302)(22.373) [FLNA](http://www.ncbi.nlm.nih.gov/gene/?term=2316)(22.373)[CNTN2](http://www.ncbi.nlm.nih.gov/gene/?term=6900)(22.373)[DTNBP1](http://www.ncbi.nlm.nih.gov/gene/?term=84062)(22.373)[CRHR1](http://www.ncbi.nlm.nih.gov/gene/?term=1394)(22.373)[TH](http://www.ncbi.nlm.nih.gov/gene/?term=7054)(22.373) [OPRL1](http://www.ncbi.nlm.nih.gov/gene/?term=4987)(22.373)[ADCY5](http://www.ncbi.nlm.nih.gov/gene/?term=111)(22.373)[KCNA1](http://www.ncbi.nlm.nih.gov/gene/?term=3736)(22.373)[CYP11A1](http://www.ncbi.nlm.nih.gov/gene/?term=1583)(22.373)[CAV2](http://www.ncbi.nlm.nih.gov/gene/?term=858)(22.373) [PRSS12](http://www.ncbi.nlm.nih.gov/gene/?term=8492)(22.373)[CABP1](http://www.ncbi.nlm.nih.gov/gene/?term=9478)(22.373)[LHX6](http://www.ncbi.nlm.nih.gov/gene/?term=26468)(22.373)[FLOT1](http://www.ncbi.nlm.nih.gov/gene/?term=10211)(22.373)[FGFR2](http://www.ncbi.nlm.nih.gov/gene/?term=2263)(22.373) [GHRL](http://www.ncbi.nlm.nih.gov/gene/?term=51738)(22.373)[TBR1](http://www.ncbi.nlm.nih.gov/gene/?term=10716)(22.373)[DNM3](http://www.ncbi.nlm.nih.gov/gene/?term=26052)(22.373)[CDK5R1](http://www.ncbi.nlm.nih.gov/gene/?term=8851)(22.373)[CHRNB2](http://www.ncbi.nlm.nih.gov/gene/?term=1141)(22.373) [HTR3D](http://www.ncbi.nlm.nih.gov/gene/?term=200909)(22.373)[RAB3B](http://www.ncbi.nlm.nih.gov/gene/?term=5865)(22.373)[ACHE](http://www.ncbi.nlm.nih.gov/gene/?term=43)(22.373)[ANK3](http://www.ncbi.nlm.nih.gov/gene/?term=288)(22.373)[COLQ](http://www.ncbi.nlm.nih.gov/gene/?term=8292)(22.373) [GRIN2C](http://www.ncbi.nlm.nih.gov/gene/?term=2905)(22.373)[UROD](http://www.ncbi.nlm.nih.gov/gene/?term=7389)(22.373)[DRD4](http://www.ncbi.nlm.nih.gov/gene/?term=1815)(22.373)[CRH](http://www.ncbi.nlm.nih.gov/gene/?term=1392)(22.373)[STAR](http://www.ncbi.nlm.nih.gov/gene/?term=6770)(22.373) [ATP13A2](http://www.ncbi.nlm.nih.gov/gene/?term=23400)(22.373)[ADCY3](http://www.ncbi.nlm.nih.gov/gene/?term=109)(22.373)[CDH8](http://www.ncbi.nlm.nih.gov/gene/?term=1006)(22.373)[RYR3](http://www.ncbi.nlm.nih.gov/gene/?term=6263)(22.373)[LRRK2](http://www.ncbi.nlm.nih.gov/gene/?term=120892)(22.373) [DRD3](http://www.ncbi.nlm.nih.gov/gene/?term=1814)(22.373)[FGFR1](http://www.ncbi.nlm.nih.gov/gene/?term=2260)(22.373)[HTR3B](http://www.ncbi.nlm.nih.gov/gene/?term=9177)(22.373)[HTR3E](http://www.ncbi.nlm.nih.gov/gene/?term=285242)(22.373)[DRD5](http://www.ncbi.nlm.nih.gov/gene/?term=1816)(22.373) [ARRDC3](http://www.ncbi.nlm.nih.gov/gene/?term=57561)(22.373)[DRD1](http://www.ncbi.nlm.nih.gov/gene/?term=1812)(22.373)[KDM6B](http://www.ncbi.nlm.nih.gov/gene/?term=23135)(22.373)[ADA](http://www.ncbi.nlm.nih.gov/gene/?term=100)(22.373)[PARK2](http://www.ncbi.nlm.nih.gov/gene/?term=5071)(22.373) [ADORA1](http://www.ncbi.nlm.nih.gov/gene/?term=134)(22.373)[PINK1](http://www.ncbi.nlm.nih.gov/gene/?term=65018)(22.373)[PALM](http://www.ncbi.nlm.nih.gov/gene/?term=5064)(22.373)[GRM7](http://www.ncbi.nlm.nih.gov/gene/?term=2917)(22.373)[HTR3C](http://www.ncbi.nlm.nih.gov/gene/?term=170572)(22.373) [FOS](http://www.ncbi.nlm.nih.gov/gene/?term=2353)(22.373) |
| [Columbamine](http://www.megabionet.org/tcmid/ingredient/3934/) | [TYR](http://www.ncbi.nlm.nih.gov/gene/?term=7299)(80.882)[DCT](http://www.ncbi.nlm.nih.gov/gene/?term=1638)(55.444)[TYRP1](http://www.ncbi.nlm.nih.gov/gene/?term=7306)(55.444)[SEC14L3](http://www.ncbi.nlm.nih.gov/gene/?term=266629)(23.000)[PPP2CA](http://www.ncbi.nlm.nih.gov/gene/?term=5515)(23.000) [PRKCA](http://www.ncbi.nlm.nih.gov/gene/?term=5578)(23.000)[NR1I2](http://www.ncbi.nlm.nih.gov/gene/?term=8856)(23.000)[ALOX5](http://www.ncbi.nlm.nih.gov/gene/?term=240)(23.000)[PPP2CB](http://www.ncbi.nlm.nih.gov/gene/?term=5516)(23.000)[SEC14L2](http://www.ncbi.nlm.nih.gov/gene/?term=23541)(23.000) [DGKA](http://www.ncbi.nlm.nih.gov/gene/?term=1606)(23.000)[PRKCB](http://www.ncbi.nlm.nih.gov/gene/?term=5579)(23.000)[SEC14L4](http://www.ncbi.nlm.nih.gov/gene/?term=284904)(23.000)[AP3D1](http://www.ncbi.nlm.nih.gov/gene/?term=8943)(22.373)[OCA2](http://www.ncbi.nlm.nih.gov/gene/?term=4948)(22.373) [GPR143](http://www.ncbi.nlm.nih.gov/gene/?term=4935)(22.373) |
| [Emodin-6-Glucoside](http://www.megabionet.org/tcmid/ingredient/30947/) | This compound doesn't have any potential target with score larger than 20. |
| [5-Acetyl-7-Hydroxy-2-Methylbenzopyran-Gamma-One](http://www.megabionet.org/tcmid/ingredient/26093/) | [VKORC1](http://www.ncbi.nlm.nih.gov/gene/?term=79001)(80.882)[VKORC1L1](http://www.ncbi.nlm.nih.gov/gene/?term=154807)(55.444)[CYP4F2](http://www.ncbi.nlm.nih.gov/gene/?term=8529)(22.373)[CYP4F11](http://www.ncbi.nlm.nih.gov/gene/?term=57834)(22.373)[UBIAD1](http://www.ncbi.nlm.nih.gov/gene/?term=29914)(22.373) [CBR1](http://www.ncbi.nlm.nih.gov/gene/?term=873)(22.373) |
| [Eugenitin](http://www.megabionet.org/tcmid/ingredient/7519/) | [S100P](http://www.ncbi.nlm.nih.gov/gene/?term=6286)(48.000)[KCNMA1](http://www.ncbi.nlm.nih.gov/gene/?term=3778)(48.000) |
| [Dehydrocorydalmine](http://www.megabionet.org/tcmid/ingredient/4889/) | [PDE3A](http://www.ncbi.nlm.nih.gov/gene/?term=5139)(23.000) |
| [Rugosin A](http://www.megabionet.org/tcmid/ingredient/19039/) | This compound doesn't have any potential target with score larger than 20. |
| [Rel-1s,2s-Epoxy-4r-Furanogermacr-10(15)-En-6-One](http://www.megabionet.org/tcmid/ingredient/7099/) | [ADRA1A](http://www.ncbi.nlm.nih.gov/gene/?term=148)(23.000)[KCNH6](http://www.ncbi.nlm.nih.gov/gene/?term=81033)(23.000)[ADRA1D](http://www.ncbi.nlm.nih.gov/gene/?term=146)(23.000)[KCNH2](http://www.ncbi.nlm.nih.gov/gene/?term=3757)(23.000)[ADRA1B](http://www.ncbi.nlm.nih.gov/gene/?term=147)(23.000) [KCNH7](http://www.ncbi.nlm.nih.gov/gene/?term=90134)(23.000)[PHKG2](http://www.ncbi.nlm.nih.gov/gene/?term=5261)(22.373)[YWHAE](http://www.ncbi.nlm.nih.gov/gene/?term=7531)(22.373)[CHRNA7](http://www.ncbi.nlm.nih.gov/gene/?term=1139)(22.373)[INS](http://www.ncbi.nlm.nih.gov/gene/?term=3630)(22.373) [NPPA](http://www.ncbi.nlm.nih.gov/gene/?term=4878)(22.373)[CALCA](http://www.ncbi.nlm.nih.gov/gene/?term=796)(22.373) |
| [P-Menth-4-En-3-One](http://www.megabionet.org/tcmid/ingredient/13765/) | [CYP17A1](http://www.ncbi.nlm.nih.gov/gene/?term=1586)(122.778)[ESR1](http://www.ncbi.nlm.nih.gov/gene/?term=2099)(122.778)[PGR](http://www.ncbi.nlm.nih.gov/gene/?term=5241)(122.778)[OPRK1](http://www.ncbi.nlm.nih.gov/gene/?term=4986)(122.778)[NR3C2](http://www.ncbi.nlm.nih.gov/gene/?term=4306)(122.778) [PDE7B](http://www.ncbi.nlm.nih.gov/gene/?term=27115)(80.882)[PDE5A](http://www.ncbi.nlm.nih.gov/gene/?term=8654)(80.882)[PDE9A](http://www.ncbi.nlm.nih.gov/gene/?term=5152)(80.882)[PDE4A](http://www.ncbi.nlm.nih.gov/gene/?term=5141)(80.882)[RYR1](http://www.ncbi.nlm.nih.gov/gene/?term=6261)(80.882) [PDE3A](http://www.ncbi.nlm.nih.gov/gene/?term=5139)(80.882)[ADORA2A](http://www.ncbi.nlm.nih.gov/gene/?term=135)(80.882)[ITPR1](http://www.ncbi.nlm.nih.gov/gene/?term=3708)(80.882)[PDE6A](http://www.ncbi.nlm.nih.gov/gene/?term=5145)(80.882)[PRKDC](http://www.ncbi.nlm.nih.gov/gene/?term=5591)(80.882) [PIK3CD](http://www.ncbi.nlm.nih.gov/gene/?term=5293)(80.882)[PDE3B](http://www.ncbi.nlm.nih.gov/gene/?term=5140)(80.882)[PGD](http://www.ncbi.nlm.nih.gov/gene/?term=5226)(80.882)[PIK3CA](http://www.ncbi.nlm.nih.gov/gene/?term=5290)(80.882)[ADORA2B](http://www.ncbi.nlm.nih.gov/gene/?term=136)(80.882) [PDE1A](http://www.ncbi.nlm.nih.gov/gene/?term=5136)(80.882)[PDE4D](http://www.ncbi.nlm.nih.gov/gene/?term=5144)(80.882)[PIK3CB](http://www.ncbi.nlm.nih.gov/gene/?term=5291)(80.882)[ITPR2](http://www.ncbi.nlm.nih.gov/gene/?term=3709)(80.882)[PDE1B](http://www.ncbi.nlm.nih.gov/gene/?term=5153)(80.882) [PDE7A](http://www.ncbi.nlm.nih.gov/gene/?term=5150)(80.882)[ITPR3](http://www.ncbi.nlm.nih.gov/gene/?term=3710)(80.882)[POLA2](http://www.ncbi.nlm.nih.gov/gene/?term=23649)(80.882)[PDE4C](http://www.ncbi.nlm.nih.gov/gene/?term=5143)(80.882)[PDE10A](http://www.ncbi.nlm.nih.gov/gene/?term=10846)(80.882) [PDE1C](http://www.ncbi.nlm.nih.gov/gene/?term=5137)(80.882)[ATM](http://www.ncbi.nlm.nih.gov/gene/?term=472)(80.882)[PDE6B](http://www.ncbi.nlm.nih.gov/gene/?term=5158)(80.882)[PDE4B](http://www.ncbi.nlm.nih.gov/gene/?term=5142)(80.882)[PDE2A](http://www.ncbi.nlm.nih.gov/gene/?term=5138)(80.882) [ADORA1](http://www.ncbi.nlm.nih.gov/gene/?term=134)(80.882)[CYP19A1](http://www.ncbi.nlm.nih.gov/gene/?term=1588)(80.882)[PDE8B](http://www.ncbi.nlm.nih.gov/gene/?term=8622)(80.882)[NT5E](http://www.ncbi.nlm.nih.gov/gene/?term=4907)(80.882)[HDAC2](http://www.ncbi.nlm.nih.gov/gene/?term=3066)(80.882) [PDE6C](http://www.ncbi.nlm.nih.gov/gene/?term=5146)(80.882)[PDE11A](http://www.ncbi.nlm.nih.gov/gene/?term=50940)(80.882)[PDE8A](http://www.ncbi.nlm.nih.gov/gene/?term=5151)(80.882)[RINT1](http://www.ncbi.nlm.nih.gov/gene/?term=60561)(55.444)[RIPK1](http://www.ncbi.nlm.nih.gov/gene/?term=8737)(55.444) [PIK3R1](http://www.ncbi.nlm.nih.gov/gene/?term=5295)(55.444)[WNT4](http://www.ncbi.nlm.nih.gov/gene/?term=54361)(55.444)[TACR2](http://www.ncbi.nlm.nih.gov/gene/?term=6865)(55.444)[AMPD3](http://www.ncbi.nlm.nih.gov/gene/?term=272)(55.444)[CX3CR1](http://www.ncbi.nlm.nih.gov/gene/?term=1524)(55.444) [ADA](http://www.ncbi.nlm.nih.gov/gene/?term=100)(55.444)[HAP1](http://www.ncbi.nlm.nih.gov/gene/?term=9001)(55.444)[IDNK](http://www.ncbi.nlm.nih.gov/gene/?term=414328)(55.444)[SRD5A1](http://www.ncbi.nlm.nih.gov/gene/?term=6715)(48.000)[AR](http://www.ncbi.nlm.nih.gov/gene/?term=367)(48.000) [NR3C1](http://www.ncbi.nlm.nih.gov/gene/?term=2908)(48.000)[ANXA1](http://www.ncbi.nlm.nih.gov/gene/?term=301)(48.000)[HDAC1](http://www.ncbi.nlm.nih.gov/gene/?term=3065)(23.000)[TFAP2C](http://www.ncbi.nlm.nih.gov/gene/?term=7022)(22.373)[HMGA2](http://www.ncbi.nlm.nih.gov/gene/?term=8091)(22.373) [TNFSF11](http://www.ncbi.nlm.nih.gov/gene/?term=8600)(22.373)[NT5C1A](http://www.ncbi.nlm.nih.gov/gene/?term=84618)(22.373)[ACTN3](http://www.ncbi.nlm.nih.gov/gene/?term=89)(22.373)[NOS1AP](http://www.ncbi.nlm.nih.gov/gene/?term=9722)(22.373)[TAC1](http://www.ncbi.nlm.nih.gov/gene/?term=6863)(22.373) [PHB](http://www.ncbi.nlm.nih.gov/gene/?term=5245)(22.373)[SIRT1](http://www.ncbi.nlm.nih.gov/gene/?term=23411)(22.373)[DGKI](http://www.ncbi.nlm.nih.gov/gene/?term=9162)(22.373)[FKBP1A](http://www.ncbi.nlm.nih.gov/gene/?term=2280)(22.373)[FURIN](http://www.ncbi.nlm.nih.gov/gene/?term=5045)(22.373) [TOX3](http://www.ncbi.nlm.nih.gov/gene/?term=27324)(22.373)[UTS2](http://www.ncbi.nlm.nih.gov/gene/?term=10911)(22.373)[STUB1](http://www.ncbi.nlm.nih.gov/gene/?term=10273)(22.373)[CTR9](http://www.ncbi.nlm.nih.gov/gene/?term=9646)(22.373)[G6PD](http://www.ncbi.nlm.nih.gov/gene/?term=2539)(22.373) [GNAT1](http://www.ncbi.nlm.nih.gov/gene/?term=2779)(22.373)[SIX1](http://www.ncbi.nlm.nih.gov/gene/?term=6495)(22.373)[QDPR](http://www.ncbi.nlm.nih.gov/gene/?term=5860)(22.373)[TGFB1](http://www.ncbi.nlm.nih.gov/gene/?term=7040)(22.373)[ATP11C](http://www.ncbi.nlm.nih.gov/gene/?term=286410)(22.373) [KHDRBS1](http://www.ncbi.nlm.nih.gov/gene/?term=10657)(22.373)[PPP3CB](http://www.ncbi.nlm.nih.gov/gene/?term=5532)(22.373)[SYT2](http://www.ncbi.nlm.nih.gov/gene/?term=127833)(22.373)[SIX4](http://www.ncbi.nlm.nih.gov/gene/?term=51804)(22.373)[SIRT2](http://www.ncbi.nlm.nih.gov/gene/?term=22933)(22.373) [SPI1](http://www.ncbi.nlm.nih.gov/gene/?term=6688)(22.373)[HOMER1](http://www.ncbi.nlm.nih.gov/gene/?term=9456)(22.373)[SHANK3](http://www.ncbi.nlm.nih.gov/gene/?term=85358)(22.373)[TAS1R1](http://www.ncbi.nlm.nih.gov/gene/?term=80835)(22.373)[TRIM28](http://www.ncbi.nlm.nih.gov/gene/?term=10155)(22.373) [XRCC4](http://www.ncbi.nlm.nih.gov/gene/?term=7518)(22.373)[MED1](http://www.ncbi.nlm.nih.gov/gene/?term=5469)(22.373)[NKX3-1](http://www.ncbi.nlm.nih.gov/gene/?term=4824)(22.373)[UBE2B](http://www.ncbi.nlm.nih.gov/gene/?term=7320)(22.373)[ESR2](http://www.ncbi.nlm.nih.gov/gene/?term=2100)(22.373) [HCN4](http://www.ncbi.nlm.nih.gov/gene/?term=10021)(22.373)[EDN1](http://www.ncbi.nlm.nih.gov/gene/?term=1906)(22.373)[CACNA1A](http://www.ncbi.nlm.nih.gov/gene/?term=773)(22.373)[STAT5A](http://www.ncbi.nlm.nih.gov/gene/?term=6776)(22.373)[TP53](http://www.ncbi.nlm.nih.gov/gene/?term=7157)(22.373) [GNAT3](http://www.ncbi.nlm.nih.gov/gene/?term=346562)(22.373)[HELB](http://www.ncbi.nlm.nih.gov/gene/?term=92797)(22.373)[COMT](http://www.ncbi.nlm.nih.gov/gene/?term=1312)(22.373)[SLC26A6](http://www.ncbi.nlm.nih.gov/gene/?term=65010)(22.373)[ADRA2A](http://www.ncbi.nlm.nih.gov/gene/?term=150)(22.373) [YWHAE](http://www.ncbi.nlm.nih.gov/gene/?term=7531)(22.373)[CETN2](http://www.ncbi.nlm.nih.gov/gene/?term=1069)(22.373)[POLA1](http://www.ncbi.nlm.nih.gov/gene/?term=5422)(22.373)[CRP](http://www.ncbi.nlm.nih.gov/gene/?term=1401)(22.373)[CETN1](http://www.ncbi.nlm.nih.gov/gene/?term=1068)(22.373) [NUDT9](http://www.ncbi.nlm.nih.gov/gene/?term=53343)(22.373)[PIK3CG](http://www.ncbi.nlm.nih.gov/gene/?term=5294)(22.373)[BMP2](http://www.ncbi.nlm.nih.gov/gene/?term=650)(22.373)[TNF](http://www.ncbi.nlm.nih.gov/gene/?term=7124)(22.373)[NEDD4](http://www.ncbi.nlm.nih.gov/gene/?term=4734)(22.373) [AGT](http://www.ncbi.nlm.nih.gov/gene/?term=183)(22.373)[FER](http://www.ncbi.nlm.nih.gov/gene/?term=2241)(22.373)[SHH](http://www.ncbi.nlm.nih.gov/gene/?term=6469)(22.373)[SERPINB3](http://www.ncbi.nlm.nih.gov/gene/?term=6317)(22.373)[FOXP3](http://www.ncbi.nlm.nih.gov/gene/?term=50943)(22.373) [CXCL13](http://www.ncbi.nlm.nih.gov/gene/?term=10563)(22.373)[ARID1A](http://www.ncbi.nlm.nih.gov/gene/?term=8289)(22.373)[FGF10](http://www.ncbi.nlm.nih.gov/gene/?term=2255)(22.373)[KCNB1](http://www.ncbi.nlm.nih.gov/gene/?term=3745)(22.373)[LEF1](http://www.ncbi.nlm.nih.gov/gene/?term=51176)(22.373) [PTK2B](http://www.ncbi.nlm.nih.gov/gene/?term=2185)(22.373)[LIG4](http://www.ncbi.nlm.nih.gov/gene/?term=3981)(22.373)[ADRBK1](http://www.ncbi.nlm.nih.gov/gene/?term=156)(22.373)[MAOB](http://www.ncbi.nlm.nih.gov/gene/?term=4129)(22.373)[SORCS3](http://www.ncbi.nlm.nih.gov/gene/?term=22986)(22.373) [ADRB1](http://www.ncbi.nlm.nih.gov/gene/?term=153)(22.373)[ADAM8](http://www.ncbi.nlm.nih.gov/gene/?term=101)(22.373)[VTI1A](http://www.ncbi.nlm.nih.gov/gene/?term=143187)(22.373)[SLC44A4](http://www.ncbi.nlm.nih.gov/gene/?term=80736)(22.373)[ADAP2](http://www.ncbi.nlm.nih.gov/gene/?term=55803)(22.373) [LRRC8A](http://www.ncbi.nlm.nih.gov/gene/?term=56262)(22.373)[FGFR2](http://www.ncbi.nlm.nih.gov/gene/?term=2263)(22.373)[AURKA](http://www.ncbi.nlm.nih.gov/gene/?term=6790)(22.373)[MCM3](http://www.ncbi.nlm.nih.gov/gene/?term=4172)(22.373)[VCP](http://www.ncbi.nlm.nih.gov/gene/?term=7415)(22.373) [GHRL](http://www.ncbi.nlm.nih.gov/gene/?term=51738)(22.373)[BCL11B](http://www.ncbi.nlm.nih.gov/gene/?term=64919)(22.373)[BCL2](http://www.ncbi.nlm.nih.gov/gene/?term=596)(22.373)[8-Mar](http://www.ncbi.nlm.nih.gov/gene/?term=220972)(22.373)[GAS6](http://www.ncbi.nlm.nih.gov/gene/?term=2621)(22.373) [C5](http://www.ncbi.nlm.nih.gov/gene/?term=727)(22.373)[SREBF1](http://www.ncbi.nlm.nih.gov/gene/?term=6720)(22.373)[DRD2](http://www.ncbi.nlm.nih.gov/gene/?term=1813)(22.373)[PLN](http://www.ncbi.nlm.nih.gov/gene/?term=5350)(22.373)[DNM3](http://www.ncbi.nlm.nih.gov/gene/?term=26052)(22.373) [CHRNA3](http://www.ncbi.nlm.nih.gov/gene/?term=1136)(22.373)[TCF3](http://www.ncbi.nlm.nih.gov/gene/?term=6929)(22.373)[SRC](http://www.ncbi.nlm.nih.gov/gene/?term=6714)(22.373)[LONP1](http://www.ncbi.nlm.nih.gov/gene/?term=9361)(22.373)[XCL1](http://www.ncbi.nlm.nih.gov/gene/?term=6375)(22.373) [HSP90AB1](http://www.ncbi.nlm.nih.gov/gene/?term=3326)(22.373)[HIBADH](http://www.ncbi.nlm.nih.gov/gene/?term=11112)(22.373)[STAP1](http://www.ncbi.nlm.nih.gov/gene/?term=26228)(22.373)[HRC](http://www.ncbi.nlm.nih.gov/gene/?term=3270)(22.373)[UTS2R](http://www.ncbi.nlm.nih.gov/gene/?term=2837)(22.373) [SLC6A4](http://www.ncbi.nlm.nih.gov/gene/?term=6532)(22.373)[HMGCR](http://www.ncbi.nlm.nih.gov/gene/?term=3156)(22.373)[HIPK2](http://www.ncbi.nlm.nih.gov/gene/?term=28996)(22.373)[PPP3CA](http://www.ncbi.nlm.nih.gov/gene/?term=5530)(22.373)[RAPGEF2](http://www.ncbi.nlm.nih.gov/gene/?term=9693)(22.373) [TET1](http://www.ncbi.nlm.nih.gov/gene/?term=80312)(22.373)[C2CD5](http://www.ncbi.nlm.nih.gov/gene/?term=9847)(22.373)[CSF2](http://www.ncbi.nlm.nih.gov/gene/?term=1437)(22.373)[TALDO1](http://www.ncbi.nlm.nih.gov/gene/?term=6888)(22.373)[BDKRB2](http://www.ncbi.nlm.nih.gov/gene/?term=624)(22.373) [KCNH2](http://www.ncbi.nlm.nih.gov/gene/?term=3757)(22.373)[MMP28](http://www.ncbi.nlm.nih.gov/gene/?term=79148)(22.373)[HIF1A](http://www.ncbi.nlm.nih.gov/gene/?term=3091)(22.373)[KIT](http://www.ncbi.nlm.nih.gov/gene/?term=3815)(22.373)[INS](http://www.ncbi.nlm.nih.gov/gene/?term=3630)(22.373) [MTOR](http://www.ncbi.nlm.nih.gov/gene/?term=2475)(22.373)[WNT10B](http://www.ncbi.nlm.nih.gov/gene/?term=7480)(22.373)[SLC18A3](http://www.ncbi.nlm.nih.gov/gene/?term=6572)(22.373)[ABCC4](http://www.ncbi.nlm.nih.gov/gene/?term=10257)(22.373)[BNIP3](http://www.ncbi.nlm.nih.gov/gene/?term=664)(22.373) [ZFPM1](http://www.ncbi.nlm.nih.gov/gene/?term=161882)(22.373)[GATA3](http://www.ncbi.nlm.nih.gov/gene/?term=2625)(22.373)[UBR5](http://www.ncbi.nlm.nih.gov/gene/?term=51366)(22.373)[AREG](http://www.ncbi.nlm.nih.gov/gene/?term=374)(22.373)[CRH](http://www.ncbi.nlm.nih.gov/gene/?term=1392)(22.373) [ZFP42](http://www.ncbi.nlm.nih.gov/gene/?term=132625)(22.373)[PIK3R6](http://www.ncbi.nlm.nih.gov/gene/?term=146850)(22.373)[PF4](http://www.ncbi.nlm.nih.gov/gene/?term=5196)(22.373)[ATP1A2](http://www.ncbi.nlm.nih.gov/gene/?term=477)(22.373)[AVPR2](http://www.ncbi.nlm.nih.gov/gene/?term=554)(22.373) [CAMK2D](http://www.ncbi.nlm.nih.gov/gene/?term=817)(22.373)[ADRA1B](http://www.ncbi.nlm.nih.gov/gene/?term=147)(22.373)[IL10](http://www.ncbi.nlm.nih.gov/gene/?term=3586)(22.373)[NEFL](http://www.ncbi.nlm.nih.gov/gene/?term=4747)(22.373)[UCN2](http://www.ncbi.nlm.nih.gov/gene/?term=90226)(22.373) [ATP1A1](http://www.ncbi.nlm.nih.gov/gene/?term=476)(22.373)[RYR3](http://www.ncbi.nlm.nih.gov/gene/?term=6263)(22.373)[PPP3R1](http://www.ncbi.nlm.nih.gov/gene/?term=5534)(22.373)[SPX](http://www.ncbi.nlm.nih.gov/gene/?term=80763)(22.373)[ATP2A1](http://www.ncbi.nlm.nih.gov/gene/?term=487)(22.373) [SMO](http://www.ncbi.nlm.nih.gov/gene/?term=6608)(22.373)[RAG1](http://www.ncbi.nlm.nih.gov/gene/?term=5896)(22.373)[RAB8B](http://www.ncbi.nlm.nih.gov/gene/?term=51762)(22.373)[DKK3](http://www.ncbi.nlm.nih.gov/gene/?term=27122)(22.373)[AHCYL1](http://www.ncbi.nlm.nih.gov/gene/?term=10768)(22.373) [AQP1](http://www.ncbi.nlm.nih.gov/gene/?term=358)(22.373)[TNFAIP3](http://www.ncbi.nlm.nih.gov/gene/?term=7128)(22.373)[TAC4](http://www.ncbi.nlm.nih.gov/gene/?term=255061)(22.373)[DPPA3](http://www.ncbi.nlm.nih.gov/gene/?term=359787)(22.373)[CASQ1](http://www.ncbi.nlm.nih.gov/gene/?term=844)(22.373) [FKBP1B](http://www.ncbi.nlm.nih.gov/gene/?term=2281)(22.373)[CDC42](http://www.ncbi.nlm.nih.gov/gene/?term=998)(22.373)[IL4](http://www.ncbi.nlm.nih.gov/gene/?term=3565)(22.373)[POLB](http://www.ncbi.nlm.nih.gov/gene/?term=5423)(22.373)[IDO1](http://www.ncbi.nlm.nih.gov/gene/?term=3620)(22.373) [ATP2B4](http://www.ncbi.nlm.nih.gov/gene/?term=493)(22.373)[CTNNB1](http://www.ncbi.nlm.nih.gov/gene/?term=1499)(22.373)[PGLS](http://www.ncbi.nlm.nih.gov/gene/?term=25796)(22.373)[SLC9A3R1](http://www.ncbi.nlm.nih.gov/gene/?term=9368)(22.373)[IGF2](http://www.ncbi.nlm.nih.gov/gene/?term=3481)(22.373) [HPS4](http://www.ncbi.nlm.nih.gov/gene/?term=89781)(22.373)[TKT](http://www.ncbi.nlm.nih.gov/gene/?term=7086)(22.373)[DRD1](http://www.ncbi.nlm.nih.gov/gene/?term=1812)(22.373)[ADAP1](http://www.ncbi.nlm.nih.gov/gene/?term=11033)(22.373)[EDA](http://www.ncbi.nlm.nih.gov/gene/?term=1896)(22.373) [RYR2](http://www.ncbi.nlm.nih.gov/gene/?term=6262)(22.373)[CD34](http://www.ncbi.nlm.nih.gov/gene/?term=947)(22.373)[HSP90AA1](http://www.ncbi.nlm.nih.gov/gene/?term=3320)(22.373)[LTA](http://www.ncbi.nlm.nih.gov/gene/?term=4049)(22.373)[SPR](http://www.ncbi.nlm.nih.gov/gene/?term=6697)(22.373) [GPER1](http://www.ncbi.nlm.nih.gov/gene/?term=2852)(22.373)[CBFA2T3](http://www.ncbi.nlm.nih.gov/gene/?term=863)(22.373)[CALHM1](http://www.ncbi.nlm.nih.gov/gene/?term=255022)(22.373)[WNT5A](http://www.ncbi.nlm.nih.gov/gene/?term=7474)(22.373)[SELP](http://www.ncbi.nlm.nih.gov/gene/?term=6403)(22.373) [XRCC6BP1](http://www.ncbi.nlm.nih.gov/gene/?term=91419)(22.373)[PARP10](http://www.ncbi.nlm.nih.gov/gene/?term=84875)(22.373)[HPRT1](http://www.ncbi.nlm.nih.gov/gene/?term=3251)(22.373)[AMICA1](http://www.ncbi.nlm.nih.gov/gene/?term=120425)(22.373)[TAS1R3](http://www.ncbi.nlm.nih.gov/gene/?term=83756)(22.373) [NOS1](http://www.ncbi.nlm.nih.gov/gene/?term=4842)(22.373)[TREM1](http://www.ncbi.nlm.nih.gov/gene/?term=54210)(22.373)[TAL1](http://www.ncbi.nlm.nih.gov/gene/?term=6886)(22.373)[ALAD](http://www.ncbi.nlm.nih.gov/gene/?term=210)(22.373)[PAWR](http://www.ncbi.nlm.nih.gov/gene/?term=5074)(22.373) [SOX9](http://www.ncbi.nlm.nih.gov/gene/?term=6662)(22.373)[BMP5](http://www.ncbi.nlm.nih.gov/gene/?term=653)(22.373)[PDGFB](http://www.ncbi.nlm.nih.gov/gene/?term=5155)(22.373)[RTN2](http://www.ncbi.nlm.nih.gov/gene/?term=6253)(22.373)[CD63](http://www.ncbi.nlm.nih.gov/gene/?term=967)(22.373) [CAMK2G](http://www.ncbi.nlm.nih.gov/gene/?term=818)(22.373)[LAMP2](http://www.ncbi.nlm.nih.gov/gene/?term=3920)(22.373)[NEFH](http://www.ncbi.nlm.nih.gov/gene/?term=4744)(22.373)[GLYR1](http://www.ncbi.nlm.nih.gov/gene/?term=84656)(22.373)[VDR](http://www.ncbi.nlm.nih.gov/gene/?term=7421)(22.373) [GNAS](http://www.ncbi.nlm.nih.gov/gene/?term=2778)(22.373)[NCBP2](http://www.ncbi.nlm.nih.gov/gene/?term=22916)(22.373)[TPM1](http://www.ncbi.nlm.nih.gov/gene/?term=7168)(22.373)[PIK3R5](http://www.ncbi.nlm.nih.gov/gene/?term=23533)(22.373)[PCSK6](http://www.ncbi.nlm.nih.gov/gene/?term=5046)(22.373) [TRIM24](http://www.ncbi.nlm.nih.gov/gene/?term=8805)(22.373)[REST](http://www.ncbi.nlm.nih.gov/gene/?term=5978)(22.373)[CRHR2](http://www.ncbi.nlm.nih.gov/gene/?term=1395)(22.373)[HCN2](http://www.ncbi.nlm.nih.gov/gene/?term=610)(22.373)[RAG2](http://www.ncbi.nlm.nih.gov/gene/?term=5897)(22.373) [NFX1](http://www.ncbi.nlm.nih.gov/gene/?term=4799)(22.373)[TAS1R2](http://www.ncbi.nlm.nih.gov/gene/?term=80834)(22.373) |
| [Chrysanthemin](http://www.megabionet.org/tcmid/ingredient/3594/) | This compound doesn't have any potential target with score larger than 20. |
| [Corypalmine](http://www.megabionet.org/tcmid/ingredient/4122/) | This compound doesn't have any potential target with score larger than 20. |
| [Rhapontigenin](http://www.megabionet.org/tcmid/ingredient/18733/) | [CNR2](http://www.ncbi.nlm.nih.gov/gene/?term=1269)(23.000)[TYR](http://www.ncbi.nlm.nih.gov/gene/?term=7299)(23.000)[CNR1](http://www.ncbi.nlm.nih.gov/gene/?term=1268)(23.000) |
| [Rottlerin](http://www.megabionet.org/tcmid/ingredient/18925/) | [SOAT1](http://www.ncbi.nlm.nih.gov/gene/?term=6646)(23.000)[MTTP](http://www.ncbi.nlm.nih.gov/gene/?term=4547)(23.000)[CNR2](http://www.ncbi.nlm.nih.gov/gene/?term=1269)(23.000)[CNR1](http://www.ncbi.nlm.nih.gov/gene/?term=1268)(23.000)[SOAT2](http://www.ncbi.nlm.nih.gov/gene/?term=8435)(23.000) [DRD2](http://www.ncbi.nlm.nih.gov/gene/?term=1813)(22.373)[PAWR](http://www.ncbi.nlm.nih.gov/gene/?term=5074)(22.373) |
| [3,5-Di-O-Galloyl-4-O-Digalloylquinic Acid](http://www.megabionet.org/tcmid/ingredient/30914/) | This compound doesn't have any potential target with score larger than 20. |
| [Corydalmine](http://www.megabionet.org/tcmid/ingredient/4104/) | [ACHE](http://www.ncbi.nlm.nih.gov/gene/?term=43)(122.778)[CHRNA2](http://www.ncbi.nlm.nih.gov/gene/?term=1135)(122.778)[HTR3A](http://www.ncbi.nlm.nih.gov/gene/?term=3359)(122.778)[CHRM2](http://www.ncbi.nlm.nih.gov/gene/?term=1129)(122.778)[CHRM3](http://www.ncbi.nlm.nih.gov/gene/?term=1131)(48.000) [DRD2](http://www.ncbi.nlm.nih.gov/gene/?term=1813)(48.000)[SLC18A2](http://www.ncbi.nlm.nih.gov/gene/?term=6571)(48.000)[BCHE](http://www.ncbi.nlm.nih.gov/gene/?term=590)(48.000)[ESR2](http://www.ncbi.nlm.nih.gov/gene/?term=2100)(23.000)[OPRD1](http://www.ncbi.nlm.nih.gov/gene/?term=4985)(23.000) [ESR1](http://www.ncbi.nlm.nih.gov/gene/?term=2099)(23.000)[OPRK1](http://www.ncbi.nlm.nih.gov/gene/?term=4986)(23.000)[OPRM1](http://www.ncbi.nlm.nih.gov/gene/?term=4988)(23.000) |
| [Myrtenol](http://www.megabionet.org/tcmid/ingredient/15213/) | [ESR1](http://www.ncbi.nlm.nih.gov/gene/?term=2099)(122.778)[PGR](http://www.ncbi.nlm.nih.gov/gene/?term=5241)(122.778)[RDH11](http://www.ncbi.nlm.nih.gov/gene/?term=51109)(80.882)[RBP3](http://www.ncbi.nlm.nih.gov/gene/?term=5949)(80.882)[RETSAT](http://www.ncbi.nlm.nih.gov/gene/?term=54884)(80.882) [RDH13](http://www.ncbi.nlm.nih.gov/gene/?term=112724)(80.882)[DHRS3](http://www.ncbi.nlm.nih.gov/gene/?term=9249)(80.882)[RDH5](http://www.ncbi.nlm.nih.gov/gene/?term=5959)(80.882)[ALDH1A3](http://www.ncbi.nlm.nih.gov/gene/?term=220)(80.882)[RDH12](http://www.ncbi.nlm.nih.gov/gene/?term=145226)(80.882) [DHRS4](http://www.ncbi.nlm.nih.gov/gene/?term=10901)(80.882)[RBP1](http://www.ncbi.nlm.nih.gov/gene/?term=5947)(80.882)[ALDH1A1](http://www.ncbi.nlm.nih.gov/gene/?term=216)(80.882)[RLBP1](http://www.ncbi.nlm.nih.gov/gene/?term=6017)(80.882)[RDH14](http://www.ncbi.nlm.nih.gov/gene/?term=57665)(80.882) [RDH8](http://www.ncbi.nlm.nih.gov/gene/?term=50700)(80.882)[LRAT](http://www.ncbi.nlm.nih.gov/gene/?term=9227)(80.882)[ALDH1A2](http://www.ncbi.nlm.nih.gov/gene/?term=8854)(80.882)[ALDH3A1](http://www.ncbi.nlm.nih.gov/gene/?term=218)(55.444)[RHO](http://www.ncbi.nlm.nih.gov/gene/?term=6010)(55.444) [RS1](http://www.ncbi.nlm.nih.gov/gene/?term=6247)(55.444)[AR](http://www.ncbi.nlm.nih.gov/gene/?term=367)(48.000)[CTSH](http://www.ncbi.nlm.nih.gov/gene/?term=1512)(22.373)[OPN4](http://www.ncbi.nlm.nih.gov/gene/?term=94233)(22.373)[RDH10](http://www.ncbi.nlm.nih.gov/gene/?term=157506)(22.373) [ALDH8A1](http://www.ncbi.nlm.nih.gov/gene/?term=64577)(22.373)[SHBG](http://www.ncbi.nlm.nih.gov/gene/?term=6462)(22.373)[DHRS2](http://www.ncbi.nlm.nih.gov/gene/?term=10202)(22.373)[HSD17B7](http://www.ncbi.nlm.nih.gov/gene/?term=51478)(22.373)[THRA](http://www.ncbi.nlm.nih.gov/gene/?term=7067)(22.373) [PAX2](http://www.ncbi.nlm.nih.gov/gene/?term=5076)(22.373)[CBR3](http://www.ncbi.nlm.nih.gov/gene/?term=874)(22.373)[GATA3](http://www.ncbi.nlm.nih.gov/gene/?term=2625)(22.373)[ASCL1](http://www.ncbi.nlm.nih.gov/gene/?term=429)(22.373)[DLX5](http://www.ncbi.nlm.nih.gov/gene/?term=1749)(22.373) [RET](http://www.ncbi.nlm.nih.gov/gene/?term=5979)(22.373)[THRB](http://www.ncbi.nlm.nih.gov/gene/?term=7068)(22.373)[CRYM](http://www.ncbi.nlm.nih.gov/gene/?term=1428)(22.373)[TSPO](http://www.ncbi.nlm.nih.gov/gene/?term=706)(22.373)[OPN5](http://www.ncbi.nlm.nih.gov/gene/?term=221391)(22.373) [DHRS9](http://www.ncbi.nlm.nih.gov/gene/?term=10170)(22.373)[CBR1](http://www.ncbi.nlm.nih.gov/gene/?term=873)(22.373)[CYP1A1](http://www.ncbi.nlm.nih.gov/gene/?term=1543)(22.373) |
| [Chebulagic Acid](http://www.megabionet.org/tcmid/ingredient/25032/) | This compound doesn't have any potential target with score larger than 20. |
| [Thaliporphine](http://www.megabionet.org/tcmid/ingredient/21243/) | [HTR2A](http://www.ncbi.nlm.nih.gov/gene/?term=3356)(122.778)[HTR2B](http://www.ncbi.nlm.nih.gov/gene/?term=3357)(122.778)[ADRA2A](http://www.ncbi.nlm.nih.gov/gene/?term=150)(122.778)[HTR1B](http://www.ncbi.nlm.nih.gov/gene/?term=3351)(122.778)[HTR2C](http://www.ncbi.nlm.nih.gov/gene/?term=3358)(122.778) [HTR1D](http://www.ncbi.nlm.nih.gov/gene/?term=3352)(122.778)[DRD2](http://www.ncbi.nlm.nih.gov/gene/?term=1813)(122.778)[DRD4](http://www.ncbi.nlm.nih.gov/gene/?term=1815)(122.778)[HTR1A](http://www.ncbi.nlm.nih.gov/gene/?term=3350)(122.778)[ADRA2C](http://www.ncbi.nlm.nih.gov/gene/?term=152)(122.778) [CALY](http://www.ncbi.nlm.nih.gov/gene/?term=50632)(122.778)[DRD3](http://www.ncbi.nlm.nih.gov/gene/?term=1814)(122.778)[DRD5](http://www.ncbi.nlm.nih.gov/gene/?term=1816)(122.778)[DRD1](http://www.ncbi.nlm.nih.gov/gene/?term=1812)(122.778)[ADRA2B](http://www.ncbi.nlm.nih.gov/gene/?term=151)(122.778) [ACHE](http://www.ncbi.nlm.nih.gov/gene/?term=43)(48.000)[SLC18A2](http://www.ncbi.nlm.nih.gov/gene/?term=6571)(48.000)[CHRNA2](http://www.ncbi.nlm.nih.gov/gene/?term=1135)(48.000)[HTR3A](http://www.ncbi.nlm.nih.gov/gene/?term=3359)(48.000)[CHRM2](http://www.ncbi.nlm.nih.gov/gene/?term=1129)(48.000) [ESR2](http://www.ncbi.nlm.nih.gov/gene/?term=2100)(23.000)[OPRD1](http://www.ncbi.nlm.nih.gov/gene/?term=4985)(23.000)[ESR1](http://www.ncbi.nlm.nih.gov/gene/?term=2099)(23.000)[OPRK1](http://www.ncbi.nlm.nih.gov/gene/?term=4986)(23.000)[OPRM1](http://www.ncbi.nlm.nih.gov/gene/?term=4988)(23.000) |
| [(+)-Corydaline](http://www.megabionet.org/tcmid/ingredient/4103/) | [ACHE](http://www.ncbi.nlm.nih.gov/gene/?term=43)(122.778)[CHRNA2](http://www.ncbi.nlm.nih.gov/gene/?term=1135)(122.778)[HTR3A](http://www.ncbi.nlm.nih.gov/gene/?term=3359)(122.778)[CHRM2](http://www.ncbi.nlm.nih.gov/gene/?term=1129)(122.778)[CYBA](http://www.ncbi.nlm.nih.gov/gene/?term=1535)(80.882) [CHRNA4](http://www.ncbi.nlm.nih.gov/gene/?term=1137)(80.882)[RAC2](http://www.ncbi.nlm.nih.gov/gene/?term=5880)(80.882)[RAC1](http://www.ncbi.nlm.nih.gov/gene/?term=5879)(80.882)[OPRD1](http://www.ncbi.nlm.nih.gov/gene/?term=4985)(80.882)[NCF4](http://www.ncbi.nlm.nih.gov/gene/?term=4689)(80.882) [CHRNA7](http://www.ncbi.nlm.nih.gov/gene/?term=1139)(80.882)[CYBB](http://www.ncbi.nlm.nih.gov/gene/?term=1536)(80.882)[NCF1](http://www.ncbi.nlm.nih.gov/gene/?term=653361)(80.882)[SIGMAR1](http://www.ncbi.nlm.nih.gov/gene/?term=10280)(80.882)[NCF2](http://www.ncbi.nlm.nih.gov/gene/?term=4688)(80.882) [OPRK1](http://www.ncbi.nlm.nih.gov/gene/?term=4986)(80.882)[CHRNA3](http://www.ncbi.nlm.nih.gov/gene/?term=1136)(80.882)[RAC3](http://www.ncbi.nlm.nih.gov/gene/?term=5881)(80.882)[CHRNB2](http://www.ncbi.nlm.nih.gov/gene/?term=1141)(80.882)[SLC6A4](http://www.ncbi.nlm.nih.gov/gene/?term=6532)(80.882) [OPRM1](http://www.ncbi.nlm.nih.gov/gene/?term=4988)(80.882)[PGRMC1](http://www.ncbi.nlm.nih.gov/gene/?term=10857)(80.882)[GRIN3A](http://www.ncbi.nlm.nih.gov/gene/?term=116443)(80.882)[CHRFAM7A](http://www.ncbi.nlm.nih.gov/gene/?term=89832)(80.882)[CHRNB4](http://www.ncbi.nlm.nih.gov/gene/?term=1143)(80.882) [SLC6A2](http://www.ncbi.nlm.nih.gov/gene/?term=6530)(80.882)[TLR4](http://www.ncbi.nlm.nih.gov/gene/?term=7099)(55.444)[ELANE](http://www.ncbi.nlm.nih.gov/gene/?term=1991)(55.444)[CTSG](http://www.ncbi.nlm.nih.gov/gene/?term=1511)(55.444)[SLC18A1](http://www.ncbi.nlm.nih.gov/gene/?term=6570)(55.444) [CEND1](http://www.ncbi.nlm.nih.gov/gene/?term=51286)(55.444)[SLC22A1](http://www.ncbi.nlm.nih.gov/gene/?term=6580)(55.444)[ADRA1B](http://www.ncbi.nlm.nih.gov/gene/?term=147)(55.444)[WLS](http://www.ncbi.nlm.nih.gov/gene/?term=79971)(55.444)[CHRNA9](http://www.ncbi.nlm.nih.gov/gene/?term=55584)(55.444) [CHRM3](http://www.ncbi.nlm.nih.gov/gene/?term=1131)(48.000)[DRD2](http://www.ncbi.nlm.nih.gov/gene/?term=1813)(48.000)[SLC18A2](http://www.ncbi.nlm.nih.gov/gene/?term=6571)(48.000)[BCHE](http://www.ncbi.nlm.nih.gov/gene/?term=590)(48.000)[ADRA1A](http://www.ncbi.nlm.nih.gov/gene/?term=148)(23.000) [HTR2A](http://www.ncbi.nlm.nih.gov/gene/?term=3356)(23.000)[CHRM1](http://www.ncbi.nlm.nih.gov/gene/?term=1128)(23.000)[HTR2B](http://www.ncbi.nlm.nih.gov/gene/?term=3357)(23.000)[ADRA2A](http://www.ncbi.nlm.nih.gov/gene/?term=150)(23.000)[HTR6](http://www.ncbi.nlm.nih.gov/gene/?term=3362)(23.000) [HTR2C](http://www.ncbi.nlm.nih.gov/gene/?term=3358)(23.000)[ADRA1D](http://www.ncbi.nlm.nih.gov/gene/?term=146)(23.000)[CHRM5](http://www.ncbi.nlm.nih.gov/gene/?term=1133)(23.000)[HRH4](http://www.ncbi.nlm.nih.gov/gene/?term=59340)(23.000)[HRH1](http://www.ncbi.nlm.nih.gov/gene/?term=3269)(23.000) [KCNH2](http://www.ncbi.nlm.nih.gov/gene/?term=3757)(23.000)[HRH2](http://www.ncbi.nlm.nih.gov/gene/?term=3274)(23.000)[HTR1A](http://www.ncbi.nlm.nih.gov/gene/?term=3350)(23.000)[ADRA2C](http://www.ncbi.nlm.nih.gov/gene/?term=152)(23.000)[CHRM4](http://www.ncbi.nlm.nih.gov/gene/?term=1132)(23.000) [ADRA2B](http://www.ncbi.nlm.nih.gov/gene/?term=151)(23.000)[ACTN3](http://www.ncbi.nlm.nih.gov/gene/?term=89)(22.373)[GPM6B](http://www.ncbi.nlm.nih.gov/gene/?term=2824)(22.373)[HMOX1](http://www.ncbi.nlm.nih.gov/gene/?term=3162)(22.373)[TAC1](http://www.ncbi.nlm.nih.gov/gene/?term=6863)(22.373) [MC2R](http://www.ncbi.nlm.nih.gov/gene/?term=4158)(22.373)[SLITRK6](http://www.ncbi.nlm.nih.gov/gene/?term=84189)(22.373)[UTS2](http://www.ncbi.nlm.nih.gov/gene/?term=10911)(22.373)[NAV2](http://www.ncbi.nlm.nih.gov/gene/?term=89797)(22.373)[PDE3A](http://www.ncbi.nlm.nih.gov/gene/?term=5139)(22.373) [SCT](http://www.ncbi.nlm.nih.gov/gene/?term=6343)(22.373)[TUSC2](http://www.ncbi.nlm.nih.gov/gene/?term=11334)(22.373)[RHOA](http://www.ncbi.nlm.nih.gov/gene/?term=387)(22.373)[AVPR1A](http://www.ncbi.nlm.nih.gov/gene/?term=552)(22.373)[ADORA2A](http://www.ncbi.nlm.nih.gov/gene/?term=135)(22.373) [HCN4](http://www.ncbi.nlm.nih.gov/gene/?term=10021)(22.373)[PHKG2](http://www.ncbi.nlm.nih.gov/gene/?term=5261)(22.373)[OXT](http://www.ncbi.nlm.nih.gov/gene/?term=5020)(22.373)[CACNA1A](http://www.ncbi.nlm.nih.gov/gene/?term=773)(22.373)[CCL5](http://www.ncbi.nlm.nih.gov/gene/?term=6352)(22.373) [APLP1](http://www.ncbi.nlm.nih.gov/gene/?term=333)(22.373)[LAMP1](http://www.ncbi.nlm.nih.gov/gene/?term=3916)(22.373)[PPP1R9B](http://www.ncbi.nlm.nih.gov/gene/?term=84687)(22.373)[YWHAE](http://www.ncbi.nlm.nih.gov/gene/?term=7531)(22.373)[SUMO1](http://www.ncbi.nlm.nih.gov/gene/?term=7341)(22.373) [HTR1B](http://www.ncbi.nlm.nih.gov/gene/?term=3351)(22.373)[CNR2](http://www.ncbi.nlm.nih.gov/gene/?term=1269)(22.373)[NOXA1](http://www.ncbi.nlm.nih.gov/gene/?term=10811)(22.373)[MC1R](http://www.ncbi.nlm.nih.gov/gene/?term=4157)(22.373)[MC4R](http://www.ncbi.nlm.nih.gov/gene/?term=4160)(22.373) [IL1B](http://www.ncbi.nlm.nih.gov/gene/?term=3553)(22.373)[AGT](http://www.ncbi.nlm.nih.gov/gene/?term=183)(22.373)[CRHBP](http://www.ncbi.nlm.nih.gov/gene/?term=1393)(22.373)[F2R](http://www.ncbi.nlm.nih.gov/gene/?term=2149)(22.373)[MTNR1B](http://www.ncbi.nlm.nih.gov/gene/?term=4544)(22.373) [FGF10](http://www.ncbi.nlm.nih.gov/gene/?term=2255)(22.373)[EGR1](http://www.ncbi.nlm.nih.gov/gene/?term=1958)(22.373)[NOXO1](http://www.ncbi.nlm.nih.gov/gene/?term=124056)(22.373)[ADAM8](http://www.ncbi.nlm.nih.gov/gene/?term=101)(22.373)[SLC44A4](http://www.ncbi.nlm.nih.gov/gene/?term=80736)(22.373) [FLOT1](http://www.ncbi.nlm.nih.gov/gene/?term=10211)(22.373)[NPY5R](http://www.ncbi.nlm.nih.gov/gene/?term=4889)(22.373)[VCP](http://www.ncbi.nlm.nih.gov/gene/?term=7415)(22.373)[GHRL](http://www.ncbi.nlm.nih.gov/gene/?term=51738)(22.373)[TBR1](http://www.ncbi.nlm.nih.gov/gene/?term=10716)(22.373) [PTPRO](http://www.ncbi.nlm.nih.gov/gene/?term=5800)(22.373)[OXTR](http://www.ncbi.nlm.nih.gov/gene/?term=5021)(22.373)[PAX2](http://www.ncbi.nlm.nih.gov/gene/?term=5076)(22.373)[CACNA1C](http://www.ncbi.nlm.nih.gov/gene/?term=775)(22.373)[F2RL1](http://www.ncbi.nlm.nih.gov/gene/?term=2150)(22.373) [UTS2R](http://www.ncbi.nlm.nih.gov/gene/?term=2837)(22.373)[ACVR2A](http://www.ncbi.nlm.nih.gov/gene/?term=92)(22.373)[RAPGEF2](http://www.ncbi.nlm.nih.gov/gene/?term=9693)(22.373)[TIRAP](http://www.ncbi.nlm.nih.gov/gene/?term=114609)(22.373)[AIF1](http://www.ncbi.nlm.nih.gov/gene/?term=199)(22.373) [GHRH](http://www.ncbi.nlm.nih.gov/gene/?term=2691)(22.373)[HCRT](http://www.ncbi.nlm.nih.gov/gene/?term=3060)(22.373)[RAB3B](http://www.ncbi.nlm.nih.gov/gene/?term=5865)(22.373)[CNR1](http://www.ncbi.nlm.nih.gov/gene/?term=1268)(22.373)[HIF1A](http://www.ncbi.nlm.nih.gov/gene/?term=3091)(22.373) [INS](http://www.ncbi.nlm.nih.gov/gene/?term=3630)(22.373)[ACE](http://www.ncbi.nlm.nih.gov/gene/?term=1636)(22.373)[SLC18A3](http://www.ncbi.nlm.nih.gov/gene/?term=6572)(22.373)[BNIP3](http://www.ncbi.nlm.nih.gov/gene/?term=664)(22.373)[DRD4](http://www.ncbi.nlm.nih.gov/gene/?term=1815)(22.373) [CRH](http://www.ncbi.nlm.nih.gov/gene/?term=1392)(22.373)[GLI3](http://www.ncbi.nlm.nih.gov/gene/?term=2737)(22.373)[PLXNB3](http://www.ncbi.nlm.nih.gov/gene/?term=5365)(22.373)[CAV3](http://www.ncbi.nlm.nih.gov/gene/?term=859)(22.373)[NEFL](http://www.ncbi.nlm.nih.gov/gene/?term=4747)(22.373) [UCN2](http://www.ncbi.nlm.nih.gov/gene/?term=90226)(22.373)[IL6](http://www.ncbi.nlm.nih.gov/gene/?term=3569)(22.373)[RNF207](http://www.ncbi.nlm.nih.gov/gene/?term=388591)(22.373)[TACR2](http://www.ncbi.nlm.nih.gov/gene/?term=6865)(22.373)[PTGS2](http://www.ncbi.nlm.nih.gov/gene/?term=5743)(22.373) [NRP1](http://www.ncbi.nlm.nih.gov/gene/?term=8829)(22.373)[NPPA](http://www.ncbi.nlm.nih.gov/gene/?term=4878)(22.373)[MC5R](http://www.ncbi.nlm.nih.gov/gene/?term=4161)(22.373)[AQP1](http://www.ncbi.nlm.nih.gov/gene/?term=358)(22.373)[MC3R](http://www.ncbi.nlm.nih.gov/gene/?term=4159)(22.373) [TAC4](http://www.ncbi.nlm.nih.gov/gene/?term=255061)(22.373)[TNR](http://www.ncbi.nlm.nih.gov/gene/?term=7143)(22.373)[NOS3](http://www.ncbi.nlm.nih.gov/gene/?term=4846)(22.373)[EPHB1](http://www.ncbi.nlm.nih.gov/gene/?term=2047)(22.373)[SNCA](http://www.ncbi.nlm.nih.gov/gene/?term=6622)(22.373) [DRD1](http://www.ncbi.nlm.nih.gov/gene/?term=1812)(22.373)[PDE2A](http://www.ncbi.nlm.nih.gov/gene/?term=5138)(22.373)[CD34](http://www.ncbi.nlm.nih.gov/gene/?term=947)(22.373)[KCNMA1](http://www.ncbi.nlm.nih.gov/gene/?term=3778)(22.373)[POU4F3](http://www.ncbi.nlm.nih.gov/gene/?term=5459)(22.373) [CBFA2T3](http://www.ncbi.nlm.nih.gov/gene/?term=863)(22.373)[CALCA](http://www.ncbi.nlm.nih.gov/gene/?term=796)(22.373)[PINK1](http://www.ncbi.nlm.nih.gov/gene/?term=65018)(22.373)[NOS1](http://www.ncbi.nlm.nih.gov/gene/?term=4842)(22.373)[TREM1](http://www.ncbi.nlm.nih.gov/gene/?term=54210)(22.373) [GJA5](http://www.ncbi.nlm.nih.gov/gene/?term=2702)(22.373)[PDGFB](http://www.ncbi.nlm.nih.gov/gene/?term=5155)(22.373)[CARTPT](http://www.ncbi.nlm.nih.gov/gene/?term=9607)(22.373)[NEFH](http://www.ncbi.nlm.nih.gov/gene/?term=4744)(22.373)[ATP8B1](http://www.ncbi.nlm.nih.gov/gene/?term=5205)(22.373) [KISS1](http://www.ncbi.nlm.nih.gov/gene/?term=3814)(22.373)[GRM7](http://www.ncbi.nlm.nih.gov/gene/?term=2917)(22.373)[FOS](http://www.ncbi.nlm.nih.gov/gene/?term=2353)(22.373)[CRHR2](http://www.ncbi.nlm.nih.gov/gene/?term=1395)(22.373)[GRIN1](http://www.ncbi.nlm.nih.gov/gene/?term=2902)(22.373) [HCN2](http://www.ncbi.nlm.nih.gov/gene/?term=610)(22.373) |
| [Phellandral](http://www.megabionet.org/tcmid/ingredient/17040/) | [CYP17A1](http://www.ncbi.nlm.nih.gov/gene/?term=1586)(122.778)[ESR1](http://www.ncbi.nlm.nih.gov/gene/?term=2099)(122.778)[PGR](http://www.ncbi.nlm.nih.gov/gene/?term=5241)(122.778)[AR](http://www.ncbi.nlm.nih.gov/gene/?term=367)(122.778)[OPRK1](http://www.ncbi.nlm.nih.gov/gene/?term=4986)(122.778) [NR3C2](http://www.ncbi.nlm.nih.gov/gene/?term=4306)(122.778)[ACHE](http://www.ncbi.nlm.nih.gov/gene/?term=43)(80.882)[BCHE](http://www.ncbi.nlm.nih.gov/gene/?term=590)(80.882)[COLQ](http://www.ncbi.nlm.nih.gov/gene/?term=8292)(55.444)[SRD5A1](http://www.ncbi.nlm.nih.gov/gene/?term=6715)(48.000) [NR3C1](http://www.ncbi.nlm.nih.gov/gene/?term=2908)(48.000)[ANXA1](http://www.ncbi.nlm.nih.gov/gene/?term=301)(48.000)[CYP19A1](http://www.ncbi.nlm.nih.gov/gene/?term=1588)(48.000)[SLC5A7](http://www.ncbi.nlm.nih.gov/gene/?term=60482)(22.373)[NRG1](http://www.ncbi.nlm.nih.gov/gene/?term=3084)(22.373) [DMGDH](http://www.ncbi.nlm.nih.gov/gene/?term=29958)(22.373)[SIX3](http://www.ncbi.nlm.nih.gov/gene/?term=6496)(22.373)[CRP](http://www.ncbi.nlm.nih.gov/gene/?term=1401)(22.373)[PRSS12](http://www.ncbi.nlm.nih.gov/gene/?term=8492)(22.373)[SLC44A4](http://www.ncbi.nlm.nih.gov/gene/?term=80736)(22.373) [ALDH7A1](http://www.ncbi.nlm.nih.gov/gene/?term=501)(22.373)[DNM3](http://www.ncbi.nlm.nih.gov/gene/?term=26052)(22.373)[ASCL1](http://www.ncbi.nlm.nih.gov/gene/?term=429)(22.373)[ENPP6](http://www.ncbi.nlm.nih.gov/gene/?term=133121)(22.373)[CDH8](http://www.ncbi.nlm.nih.gov/gene/?term=1006)(22.373) [FNTA](http://www.ncbi.nlm.nih.gov/gene/?term=2339)(22.373)[AGRN](http://www.ncbi.nlm.nih.gov/gene/?term=375790)(22.373)[CHKA](http://www.ncbi.nlm.nih.gov/gene/?term=1119)(22.373)[CHDH](http://www.ncbi.nlm.nih.gov/gene/?term=55349)(22.373)[GRIN1](http://www.ncbi.nlm.nih.gov/gene/?term=2902)(22.373) |
| [Sennidin C](http://www.megabionet.org/tcmid/ingredient/19733/) | This compound doesn't have any potential target with score larger than 20. |
| [Dehydrocorybulbine](http://www.megabionet.org/tcmid/ingredient/4887/) | This compound doesn't have any potential target with score larger than 20. |
| [Yuehchukene](http://www.megabionet.org/tcmid/ingredient/22921/) | [SCNN1A](http://www.ncbi.nlm.nih.gov/gene/?term=6337)(80.882)[KCNJ8](http://www.ncbi.nlm.nih.gov/gene/?term=3764)(80.882)[ADRA1A](http://www.ncbi.nlm.nih.gov/gene/?term=148)(80.882)[SCN1A](http://www.ncbi.nlm.nih.gov/gene/?term=6323)(80.882)[SCNN1G](http://www.ncbi.nlm.nih.gov/gene/?term=6340)(80.882) [HTR2A](http://www.ncbi.nlm.nih.gov/gene/?term=3356)(80.882)[ADRB2](http://www.ncbi.nlm.nih.gov/gene/?term=154)(80.882)[ACACB](http://www.ncbi.nlm.nih.gov/gene/?term=32)(80.882)[MTAP](http://www.ncbi.nlm.nih.gov/gene/?term=4507)(80.882)[ADRA2A](http://www.ncbi.nlm.nih.gov/gene/?term=150)(80.882) [TLR8](http://www.ncbi.nlm.nih.gov/gene/?term=51311)(80.882)[ADRB1](http://www.ncbi.nlm.nih.gov/gene/?term=153)(80.882)[APRT](http://www.ncbi.nlm.nih.gov/gene/?term=353)(80.882)[ADRA1D](http://www.ncbi.nlm.nih.gov/gene/?term=146)(80.882)[PECR](http://www.ncbi.nlm.nih.gov/gene/?term=55825)(80.882) [HRH1](http://www.ncbi.nlm.nih.gov/gene/?term=3269)(80.882)[PRKAA1](http://www.ncbi.nlm.nih.gov/gene/?term=5562)(80.882)[HTR7](http://www.ncbi.nlm.nih.gov/gene/?term=3363)(80.882)[HRH2](http://www.ncbi.nlm.nih.gov/gene/?term=3274)(80.882)[ADRA1B](http://www.ncbi.nlm.nih.gov/gene/?term=147)(80.882) [SCNN1D](http://www.ncbi.nlm.nih.gov/gene/?term=6339)(80.882)[ADRA2C](http://www.ncbi.nlm.nih.gov/gene/?term=152)(80.882)[AOC3](http://www.ncbi.nlm.nih.gov/gene/?term=8639)(80.882)[P4HA1](http://www.ncbi.nlm.nih.gov/gene/?term=5033)(80.882)[SRPK2](http://www.ncbi.nlm.nih.gov/gene/?term=6733)(80.882) [TLR7](http://www.ncbi.nlm.nih.gov/gene/?term=51284)(80.882)[KCNJ1](http://www.ncbi.nlm.nih.gov/gene/?term=3758)(80.882)[AGTR1](http://www.ncbi.nlm.nih.gov/gene/?term=185)(80.882)[ADRB3](http://www.ncbi.nlm.nih.gov/gene/?term=155)(80.882)[ACP1](http://www.ncbi.nlm.nih.gov/gene/?term=52)(80.882) [SCNN1B](http://www.ncbi.nlm.nih.gov/gene/?term=6338)(80.882)[CYP2B6](http://www.ncbi.nlm.nih.gov/gene/?term=1555)(80.882)[ADRA2B](http://www.ncbi.nlm.nih.gov/gene/?term=151)(80.882)[SLC6A2](http://www.ncbi.nlm.nih.gov/gene/?term=6530)(80.882)[SAXO1](http://www.ncbi.nlm.nih.gov/gene/?term=158297)(55.444) [SCT](http://www.ncbi.nlm.nih.gov/gene/?term=6343)(55.444)[UGCG](http://www.ncbi.nlm.nih.gov/gene/?term=7357)(55.444)[PHKG2](http://www.ncbi.nlm.nih.gov/gene/?term=5261)(55.444)[APLP1](http://www.ncbi.nlm.nih.gov/gene/?term=333)(55.444)[ADK](http://www.ncbi.nlm.nih.gov/gene/?term=132)(55.444) [ACACA](http://www.ncbi.nlm.nih.gov/gene/?term=31)(55.444)[CHRNA7](http://www.ncbi.nlm.nih.gov/gene/?term=1139)(55.444)[PNP](http://www.ncbi.nlm.nih.gov/gene/?term=4860)(55.444)[ARRB2](http://www.ncbi.nlm.nih.gov/gene/?term=409)(55.444)[RAB7A](http://www.ncbi.nlm.nih.gov/gene/?term=7879)(55.444) [PRKAA2](http://www.ncbi.nlm.nih.gov/gene/?term=5563)(55.444)[HTR2C](http://www.ncbi.nlm.nih.gov/gene/?term=3358)(55.444)[HSP90AB1](http://www.ncbi.nlm.nih.gov/gene/?term=3326)(55.444)[ALDH3A2](http://www.ncbi.nlm.nih.gov/gene/?term=224)(55.444)[INS](http://www.ncbi.nlm.nih.gov/gene/?term=3630)(55.444) [CALCOCO2](http://www.ncbi.nlm.nih.gov/gene/?term=10241)(55.444)[SLC22A1](http://www.ncbi.nlm.nih.gov/gene/?term=6580)(55.444)[AOC2](http://www.ncbi.nlm.nih.gov/gene/?term=314)(55.444)[ARRDC3](http://www.ncbi.nlm.nih.gov/gene/?term=57561)(55.444)[CALCA](http://www.ncbi.nlm.nih.gov/gene/?term=796)(55.444) [HPRT1](http://www.ncbi.nlm.nih.gov/gene/?term=3251)(55.444)[ACTN2](http://www.ncbi.nlm.nih.gov/gene/?term=88)(55.444)[SCN3A](http://www.ncbi.nlm.nih.gov/gene/?term=6328)(23.000)[SCN2A](http://www.ncbi.nlm.nih.gov/gene/?term=6326)(23.000)[ADCY6](http://www.ncbi.nlm.nih.gov/gene/?term=112)(22.373) [ACTN3](http://www.ncbi.nlm.nih.gov/gene/?term=89)(22.373)[RSAD2](http://www.ncbi.nlm.nih.gov/gene/?term=91543)(22.373)[C1QTNF1](http://www.ncbi.nlm.nih.gov/gene/?term=114897)(22.373)[APIP](http://www.ncbi.nlm.nih.gov/gene/?term=51074)(22.373)[ORMDL1](http://www.ncbi.nlm.nih.gov/gene/?term=94101)(22.373) [UTS2](http://www.ncbi.nlm.nih.gov/gene/?term=10911)(22.373)[NAV2](http://www.ncbi.nlm.nih.gov/gene/?term=89797)(22.373)[GBA2](http://www.ncbi.nlm.nih.gov/gene/?term=57704)(22.373)[PCCA](http://www.ncbi.nlm.nih.gov/gene/?term=5095)(22.373)[ANG](http://www.ncbi.nlm.nih.gov/gene/?term=283)(22.373) [P2RX2](http://www.ncbi.nlm.nih.gov/gene/?term=22953)(22.373)[GAA](http://www.ncbi.nlm.nih.gov/gene/?term=2548)(22.373)[GBA](http://www.ncbi.nlm.nih.gov/gene/?term=2629)(22.373)[GSK3A](http://www.ncbi.nlm.nih.gov/gene/?term=2931)(22.373)[FGF1](http://www.ncbi.nlm.nih.gov/gene/?term=2246)(22.373) [OXT](http://www.ncbi.nlm.nih.gov/gene/?term=5020)(22.373)[PC](http://www.ncbi.nlm.nih.gov/gene/?term=5091)(22.373)[DLL1](http://www.ncbi.nlm.nih.gov/gene/?term=28514)(22.373)[KLHL3](http://www.ncbi.nlm.nih.gov/gene/?term=26249)(22.373)[RXFP4](http://www.ncbi.nlm.nih.gov/gene/?term=339403)(22.373) [HTR2B](http://www.ncbi.nlm.nih.gov/gene/?term=3357)(22.373)[WNK4](http://www.ncbi.nlm.nih.gov/gene/?term=65266)(22.373)[LHCGR](http://www.ncbi.nlm.nih.gov/gene/?term=3973)(22.373)[CETN2](http://www.ncbi.nlm.nih.gov/gene/?term=1069)(22.373)[CETN1](http://www.ncbi.nlm.nih.gov/gene/?term=1068)(22.373) [TLR3](http://www.ncbi.nlm.nih.gov/gene/?term=7098)(22.373)[SPIDR](http://www.ncbi.nlm.nih.gov/gene/?term=23514)(22.373)[SNX3](http://www.ncbi.nlm.nih.gov/gene/?term=8724)(22.373)[VCAM1](http://www.ncbi.nlm.nih.gov/gene/?term=7412)(22.373)[TRPV1](http://www.ncbi.nlm.nih.gov/gene/?term=7442)(22.373) [ADI1](http://www.ncbi.nlm.nih.gov/gene/?term=55256)(22.373)[BLM](http://www.ncbi.nlm.nih.gov/gene/?term=641)(22.373)[AGT](http://www.ncbi.nlm.nih.gov/gene/?term=183)(22.373)[CECR1](http://www.ncbi.nlm.nih.gov/gene/?term=51816)(22.373)[GOT1](http://www.ncbi.nlm.nih.gov/gene/?term=2805)(22.373) [HTR6](http://www.ncbi.nlm.nih.gov/gene/?term=3362)(22.373)[ENOPH1](http://www.ncbi.nlm.nih.gov/gene/?term=58478)(22.373)[CLN3](http://www.ncbi.nlm.nih.gov/gene/?term=1201)(22.373)[POR](http://www.ncbi.nlm.nih.gov/gene/?term=5447)(22.373)[PTK2B](http://www.ncbi.nlm.nih.gov/gene/?term=2185)(22.373) [MAS1](http://www.ncbi.nlm.nih.gov/gene/?term=4142)(22.373)[BMP6](http://www.ncbi.nlm.nih.gov/gene/?term=654)(22.373)[ADRBK1](http://www.ncbi.nlm.nih.gov/gene/?term=156)(22.373)[MAOB](http://www.ncbi.nlm.nih.gov/gene/?term=4129)(22.373)[MCCC1](http://www.ncbi.nlm.nih.gov/gene/?term=56922)(22.373) [P2RX3](http://www.ncbi.nlm.nih.gov/gene/?term=5024)(22.373)[KCNJ11](http://www.ncbi.nlm.nih.gov/gene/?term=3767)(22.373)[SERPINF2](http://www.ncbi.nlm.nih.gov/gene/?term=5345)(22.373)[UCHL1](http://www.ncbi.nlm.nih.gov/gene/?term=7345)(22.373)[MCCC2](http://www.ncbi.nlm.nih.gov/gene/?term=64087)(22.373) [PDE4D](http://www.ncbi.nlm.nih.gov/gene/?term=5144)(22.373)[MZB1](http://www.ncbi.nlm.nih.gov/gene/?term=51237)(22.373)[HRH3](http://www.ncbi.nlm.nih.gov/gene/?term=11255)(22.373)[P2RY1](http://www.ncbi.nlm.nih.gov/gene/?term=5028)(22.373)[AGTRAP](http://www.ncbi.nlm.nih.gov/gene/?term=57085)(22.373) [ORMDL2](http://www.ncbi.nlm.nih.gov/gene/?term=29095)(22.373)[SGK1](http://www.ncbi.nlm.nih.gov/gene/?term=6446)(22.373)[SREBF1](http://www.ncbi.nlm.nih.gov/gene/?term=6720)(22.373)[GAL3ST1](http://www.ncbi.nlm.nih.gov/gene/?term=9514)(22.373)[PLA2G1B](http://www.ncbi.nlm.nih.gov/gene/?term=5319)(22.373) [DPYS](http://www.ncbi.nlm.nih.gov/gene/?term=1807)(22.373)[MRI1](http://www.ncbi.nlm.nih.gov/gene/?term=84245)(22.373)[MSTN](http://www.ncbi.nlm.nih.gov/gene/?term=2660)(22.373)[CACNA1C](http://www.ncbi.nlm.nih.gov/gene/?term=775)(22.373)[AOC1](http://www.ncbi.nlm.nih.gov/gene/?term=26)(22.373) [MAOA](http://www.ncbi.nlm.nih.gov/gene/?term=4128)(22.373)[TYMP](http://www.ncbi.nlm.nih.gov/gene/?term=1890)(22.373)[UTS2R](http://www.ncbi.nlm.nih.gov/gene/?term=2837)(22.373)[ARRB1](http://www.ncbi.nlm.nih.gov/gene/?term=408)(22.373)[TNNC1](http://www.ncbi.nlm.nih.gov/gene/?term=7134)(22.373) [NCOA3](http://www.ncbi.nlm.nih.gov/gene/?term=8202)(22.373)[HRH4](http://www.ncbi.nlm.nih.gov/gene/?term=59340)(22.373)[NMUR2](http://www.ncbi.nlm.nih.gov/gene/?term=56923)(22.373)[TLR9](http://www.ncbi.nlm.nih.gov/gene/?term=54106)(22.373)[ACE](http://www.ncbi.nlm.nih.gov/gene/?term=1636)(22.373) [UGT8](http://www.ncbi.nlm.nih.gov/gene/?term=7368)(22.373)[AGTR2](http://www.ncbi.nlm.nih.gov/gene/?term=186)(22.373)[PYGM](http://www.ncbi.nlm.nih.gov/gene/?term=5837)(22.373)[ABCC9](http://www.ncbi.nlm.nih.gov/gene/?term=10060)(22.373)[CAV1](http://www.ncbi.nlm.nih.gov/gene/?term=857)(22.373) [PYGL](http://www.ncbi.nlm.nih.gov/gene/?term=5836)(22.373)[AVPR2](http://www.ncbi.nlm.nih.gov/gene/?term=554)(22.373)[KCNJ10](http://www.ncbi.nlm.nih.gov/gene/?term=3766)(22.373)[IL10](http://www.ncbi.nlm.nih.gov/gene/?term=3586)(22.373)[GPR101](http://www.ncbi.nlm.nih.gov/gene/?term=83550)(22.373) [PID1](http://www.ncbi.nlm.nih.gov/gene/?term=55022)(22.373)[DECR2](http://www.ncbi.nlm.nih.gov/gene/?term=26063)(22.373)[REN](http://www.ncbi.nlm.nih.gov/gene/?term=5972)(22.373)[PDGFA](http://www.ncbi.nlm.nih.gov/gene/?term=5154)(22.373)[MLYCD](http://www.ncbi.nlm.nih.gov/gene/?term=23417)(22.373) [NMI](http://www.ncbi.nlm.nih.gov/gene/?term=9111)(22.373)[PIK3AP1](http://www.ncbi.nlm.nih.gov/gene/?term=118788)(22.373)[SEC14L2](http://www.ncbi.nlm.nih.gov/gene/?term=23541)(22.373)[DRD5](http://www.ncbi.nlm.nih.gov/gene/?term=1816)(22.373)[ZNF219](http://www.ncbi.nlm.nih.gov/gene/?term=51222)(22.373) [RFK](http://www.ncbi.nlm.nih.gov/gene/?term=55312)(22.373)[ZP3](http://www.ncbi.nlm.nih.gov/gene/?term=7784)(22.373)[PCCB](http://www.ncbi.nlm.nih.gov/gene/?term=5096)(22.373)[ATP2B4](http://www.ncbi.nlm.nih.gov/gene/?term=493)(22.373)[P4HA3](http://www.ncbi.nlm.nih.gov/gene/?term=283208)(22.373) [SLC9A3R1](http://www.ncbi.nlm.nih.gov/gene/?term=9368)(22.373)[NACA](http://www.ncbi.nlm.nih.gov/gene/?term=4666)(22.373)[ADA](http://www.ncbi.nlm.nih.gov/gene/?term=100)(22.373)[POU1F1](http://www.ncbi.nlm.nih.gov/gene/?term=5449)(22.373)[KCNMA1](http://www.ncbi.nlm.nih.gov/gene/?term=3778)(22.373) [GPER1](http://www.ncbi.nlm.nih.gov/gene/?term=2852)(22.373)[KCNE2](http://www.ncbi.nlm.nih.gov/gene/?term=9992)(22.373)[ADORA1](http://www.ncbi.nlm.nih.gov/gene/?term=134)(22.373)[ADAM17](http://www.ncbi.nlm.nih.gov/gene/?term=6868)(22.373)[P4HB](http://www.ncbi.nlm.nih.gov/gene/?term=5034)(22.373) [NOS1](http://www.ncbi.nlm.nih.gov/gene/?term=4842)(22.373)[RGS2](http://www.ncbi.nlm.nih.gov/gene/?term=5997)(22.373)[TRPM8](http://www.ncbi.nlm.nih.gov/gene/?term=79054)(22.373)[UNC93B1](http://www.ncbi.nlm.nih.gov/gene/?term=81622)(22.373)[PDGFB](http://www.ncbi.nlm.nih.gov/gene/?term=5155)(22.373) [P4HA2](http://www.ncbi.nlm.nih.gov/gene/?term=8974)(22.373)[GDPGP1](http://www.ncbi.nlm.nih.gov/gene/?term=390637)(22.373)[EGFR](http://www.ncbi.nlm.nih.gov/gene/?term=1956)(22.373)[MECR](http://www.ncbi.nlm.nih.gov/gene/?term=51102)(22.373)[TPM1](http://www.ncbi.nlm.nih.gov/gene/?term=7168)(22.373) [RPS6KB1](http://www.ncbi.nlm.nih.gov/gene/?term=6198)(22.373)[EDN2](http://www.ncbi.nlm.nih.gov/gene/?term=1907)(22.373)[SYK](http://www.ncbi.nlm.nih.gov/gene/?term=6850)(22.373)[PYGB](http://www.ncbi.nlm.nih.gov/gene/?term=5834)(22.373)[PRKCD](http://www.ncbi.nlm.nih.gov/gene/?term=5580)(22.373) [ABCG1](http://www.ncbi.nlm.nih.gov/gene/?term=9619)(22.373)[RAD51](http://www.ncbi.nlm.nih.gov/gene/?term=5888)(22.373)[PAICS](http://www.ncbi.nlm.nih.gov/gene/?term=10606)(22.373)[ATP8A2](http://www.ncbi.nlm.nih.gov/gene/?term=51761)(22.373)[TRPA1](http://www.ncbi.nlm.nih.gov/gene/?term=8989)(22.373) |
| [Geraniol](http://www.megabionet.org/tcmid/ingredient/8311/) | [RDH11](http://www.ncbi.nlm.nih.gov/gene/?term=51109)(80.882)[RBP3](http://www.ncbi.nlm.nih.gov/gene/?term=5949)(80.882)[RETSAT](http://www.ncbi.nlm.nih.gov/gene/?term=54884)(80.882)[RDH13](http://www.ncbi.nlm.nih.gov/gene/?term=112724)(80.882)[DHRS3](http://www.ncbi.nlm.nih.gov/gene/?term=9249)(80.882) [RDH5](http://www.ncbi.nlm.nih.gov/gene/?term=5959)(80.882)[ALDH1A3](http://www.ncbi.nlm.nih.gov/gene/?term=220)(80.882)[RDH12](http://www.ncbi.nlm.nih.gov/gene/?term=145226)(80.882)[DHRS4](http://www.ncbi.nlm.nih.gov/gene/?term=10901)(80.882)[RBP1](http://www.ncbi.nlm.nih.gov/gene/?term=5947)(80.882) [ALDH1A1](http://www.ncbi.nlm.nih.gov/gene/?term=216)(80.882)[RLBP1](http://www.ncbi.nlm.nih.gov/gene/?term=6017)(80.882)[RDH14](http://www.ncbi.nlm.nih.gov/gene/?term=57665)(80.882)[RDH8](http://www.ncbi.nlm.nih.gov/gene/?term=50700)(80.882)[LRAT](http://www.ncbi.nlm.nih.gov/gene/?term=9227)(80.882) [ALDH1A2](http://www.ncbi.nlm.nih.gov/gene/?term=8854)(80.882)[ALDH3A1](http://www.ncbi.nlm.nih.gov/gene/?term=218)(55.444)[RHO](http://www.ncbi.nlm.nih.gov/gene/?term=6010)(55.444)[RS1](http://www.ncbi.nlm.nih.gov/gene/?term=6247)(55.444)[CTSH](http://www.ncbi.nlm.nih.gov/gene/?term=1512)(22.373) [OPN4](http://www.ncbi.nlm.nih.gov/gene/?term=94233)(22.373)[RDH10](http://www.ncbi.nlm.nih.gov/gene/?term=157506)(22.373)[ALDH8A1](http://www.ncbi.nlm.nih.gov/gene/?term=64577)(22.373)[SHBG](http://www.ncbi.nlm.nih.gov/gene/?term=6462)(22.373)[DHRS2](http://www.ncbi.nlm.nih.gov/gene/?term=10202)(22.373) [HSD17B7](http://www.ncbi.nlm.nih.gov/gene/?term=51478)(22.373)[AR](http://www.ncbi.nlm.nih.gov/gene/?term=367)(22.373)[THRA](http://www.ncbi.nlm.nih.gov/gene/?term=7067)(22.373)[PAX2](http://www.ncbi.nlm.nih.gov/gene/?term=5076)(22.373)[CBR3](http://www.ncbi.nlm.nih.gov/gene/?term=874)(22.373) [GATA3](http://www.ncbi.nlm.nih.gov/gene/?term=2625)(22.373)[ASCL1](http://www.ncbi.nlm.nih.gov/gene/?term=429)(22.373)[DLX5](http://www.ncbi.nlm.nih.gov/gene/?term=1749)(22.373)[RET](http://www.ncbi.nlm.nih.gov/gene/?term=5979)(22.373)[THRB](http://www.ncbi.nlm.nih.gov/gene/?term=7068)(22.373) [CRYM](http://www.ncbi.nlm.nih.gov/gene/?term=1428)(22.373)[TSPO](http://www.ncbi.nlm.nih.gov/gene/?term=706)(22.373)[OPN5](http://www.ncbi.nlm.nih.gov/gene/?term=221391)(22.373)[DHRS9](http://www.ncbi.nlm.nih.gov/gene/?term=10170)(22.373)[CBR1](http://www.ncbi.nlm.nih.gov/gene/?term=873)(22.373) [CYP1A1](http://www.ncbi.nlm.nih.gov/gene/?term=1543)(22.373) |
| [Rheidin A](http://www.megabionet.org/tcmid/ingredient/18741/) | [PPARG](http://www.ncbi.nlm.nih.gov/gene/?term=5468)(23.000)[FPGS](http://www.ncbi.nlm.nih.gov/gene/?term=2356)(23.000)[PTGS1](http://www.ncbi.nlm.nih.gov/gene/?term=5742)(23.000)[PTGS2](http://www.ncbi.nlm.nih.gov/gene/?term=5743)(23.000)[ALOX5](http://www.ncbi.nlm.nih.gov/gene/?term=240)(23.000) [MPL](http://www.ncbi.nlm.nih.gov/gene/?term=4352)(23.000)[TYMS](http://www.ncbi.nlm.nih.gov/gene/?term=7298)(23.000)[APOA2](http://www.ncbi.nlm.nih.gov/gene/?term=336)(22.373)[SHMT1](http://www.ncbi.nlm.nih.gov/gene/?term=6470)(22.373)[SERPINE1](http://www.ncbi.nlm.nih.gov/gene/?term=5054)(22.373) [IL1B](http://www.ncbi.nlm.nih.gov/gene/?term=3553)(22.373)[IL13](http://www.ncbi.nlm.nih.gov/gene/?term=3596)(22.373)[GGH](http://www.ncbi.nlm.nih.gov/gene/?term=8836)(22.373)[SERPINB7](http://www.ncbi.nlm.nih.gov/gene/?term=8710)(22.373) |
| [Palmatine](http://www.megabionet.org/tcmid/ingredient/16544/) | [PDE10A](http://www.ncbi.nlm.nih.gov/gene/?term=10846)(80.882)[PDE4B](http://www.ncbi.nlm.nih.gov/gene/?term=5142)(80.882)[PDE4D](http://www.ncbi.nlm.nih.gov/gene/?term=5144)(55.444)[PDE5A](http://www.ncbi.nlm.nih.gov/gene/?term=8654)(22.373)[PDE9A](http://www.ncbi.nlm.nih.gov/gene/?term=5152)(22.373) [PDE1B](http://www.ncbi.nlm.nih.gov/gene/?term=5153)(22.373)[PLN](http://www.ncbi.nlm.nih.gov/gene/?term=5350)(22.373)[HRC](http://www.ncbi.nlm.nih.gov/gene/?term=3270)(22.373)[CAMK2D](http://www.ncbi.nlm.nih.gov/gene/?term=817)(22.373)[PDE2A](http://www.ncbi.nlm.nih.gov/gene/?term=5138)(22.373) [CAMK2G](http://www.ncbi.nlm.nih.gov/gene/?term=818)(22.373)[PDE11A](http://www.ncbi.nlm.nih.gov/gene/?term=50940)(22.373) |
| [Rugosal A](http://www.megabionet.org/tcmid/ingredient/19032/) | This compound doesn't have any potential target with score larger than 20. |
| [D-Borneol](http://www.megabionet.org/tcmid/ingredient/2553/) | [TRPV3](http://www.ncbi.nlm.nih.gov/gene/?term=162514)(122.778)[OPRK1](http://www.ncbi.nlm.nih.gov/gene/?term=4986)(122.778)[TRPM8](http://www.ncbi.nlm.nih.gov/gene/?term=79054)(122.778)[TRPA1](http://www.ncbi.nlm.nih.gov/gene/?term=8989)(122.778)[KCNK4](http://www.ncbi.nlm.nih.gov/gene/?term=50801)(55.444) [ESRRG](http://www.ncbi.nlm.nih.gov/gene/?term=2104)(48.000)[COX6C](http://www.ncbi.nlm.nih.gov/gene/?term=1345)(48.000)[COX5B](http://www.ncbi.nlm.nih.gov/gene/?term=1329)(48.000)[COX7C](http://www.ncbi.nlm.nih.gov/gene/?term=1350)(48.000)[COX1](http://www.ncbi.nlm.nih.gov/gene/?term=4512)(48.000) [AKR1C2](http://www.ncbi.nlm.nih.gov/gene/?term=1646)(48.000)[COX5A](http://www.ncbi.nlm.nih.gov/gene/?term=9377)(48.000)[COX3](http://www.ncbi.nlm.nih.gov/gene/?term=4514)(48.000)[AR](http://www.ncbi.nlm.nih.gov/gene/?term=367)(48.000)[COX7A1](http://www.ncbi.nlm.nih.gov/gene/?term=1346)(48.000) [FECH](http://www.ncbi.nlm.nih.gov/gene/?term=2235)(48.000)[COX4I1](http://www.ncbi.nlm.nih.gov/gene/?term=1327)(48.000)[PLA2G1B](http://www.ncbi.nlm.nih.gov/gene/?term=5319)(48.000)[COX6A2](http://www.ncbi.nlm.nih.gov/gene/?term=1339)(48.000)[ADH1C](http://www.ncbi.nlm.nih.gov/gene/?term=126)(48.000) [COX6B1](http://www.ncbi.nlm.nih.gov/gene/?term=1340)(48.000)[FABP6](http://www.ncbi.nlm.nih.gov/gene/?term=2172)(48.000)[CES1](http://www.ncbi.nlm.nih.gov/gene/?term=1066)(48.000)[COX7B](http://www.ncbi.nlm.nih.gov/gene/?term=1349)(48.000)[COX2](http://www.ncbi.nlm.nih.gov/gene/?term=4513)(48.000) [COX8A](http://www.ncbi.nlm.nih.gov/gene/?term=1351)(48.000)[NR1H4](http://www.ncbi.nlm.nih.gov/gene/?term=9971)(48.000)[ACTN3](http://www.ncbi.nlm.nih.gov/gene/?term=89)(22.373)[TAC1](http://www.ncbi.nlm.nih.gov/gene/?term=6863)(22.373)[NGFR](http://www.ncbi.nlm.nih.gov/gene/?term=4804)(22.373) [ADRA2A](http://www.ncbi.nlm.nih.gov/gene/?term=150)(22.373)[TRPV1](http://www.ncbi.nlm.nih.gov/gene/?term=7442)(22.373)[FGF10](http://www.ncbi.nlm.nih.gov/gene/?term=2255)(22.373)[VCP](http://www.ncbi.nlm.nih.gov/gene/?term=7415)(22.373)[HIF1A](http://www.ncbi.nlm.nih.gov/gene/?term=3091)(22.373) [BNIP3](http://www.ncbi.nlm.nih.gov/gene/?term=664)(22.373)[CRH](http://www.ncbi.nlm.nih.gov/gene/?term=1392)(22.373)[NEFL](http://www.ncbi.nlm.nih.gov/gene/?term=4747)(22.373)[UCN2](http://www.ncbi.nlm.nih.gov/gene/?term=90226)(22.373)[SMO](http://www.ncbi.nlm.nih.gov/gene/?term=6608)(22.373) [TACR2](http://www.ncbi.nlm.nih.gov/gene/?term=6865)(22.373)[AQP1](http://www.ncbi.nlm.nih.gov/gene/?term=358)(22.373)[TAC4](http://www.ncbi.nlm.nih.gov/gene/?term=255061)(22.373)[CDH3](http://www.ncbi.nlm.nih.gov/gene/?term=1001)(22.373)[CBFA2T3](http://www.ncbi.nlm.nih.gov/gene/?term=863)(22.373) [NEFH](http://www.ncbi.nlm.nih.gov/gene/?term=4744)(22.373)[CRHR2](http://www.ncbi.nlm.nih.gov/gene/?term=1395)(22.373) |
| [Gallicacid](http://www.megabionet.org/tcmid/ingredient/8094/) | [SEC14L3](http://www.ncbi.nlm.nih.gov/gene/?term=266629)(114.143)[PPP2CA](http://www.ncbi.nlm.nih.gov/gene/?term=5515)(114.143)[PRKCA](http://www.ncbi.nlm.nih.gov/gene/?term=5578)(114.143)[NR1I2](http://www.ncbi.nlm.nih.gov/gene/?term=8856)(114.143)[ALOX5](http://www.ncbi.nlm.nih.gov/gene/?term=240)(114.143) [PPP2CB](http://www.ncbi.nlm.nih.gov/gene/?term=5516)(114.143)[SEC14L2](http://www.ncbi.nlm.nih.gov/gene/?term=23541)(114.143)[DGKA](http://www.ncbi.nlm.nih.gov/gene/?term=1606)(114.143)[PRKCB](http://www.ncbi.nlm.nih.gov/gene/?term=5579)(114.143)[SEC14L4](http://www.ncbi.nlm.nih.gov/gene/?term=284904)(114.143) [AKR1C1](http://www.ncbi.nlm.nih.gov/gene/?term=1645)(48.000)[PTGS1](http://www.ncbi.nlm.nih.gov/gene/?term=5742)(48.000)[PTGS2](http://www.ncbi.nlm.nih.gov/gene/?term=5743)(48.000)[SEC14L6](http://www.ncbi.nlm.nih.gov/gene/?term=730005)(25.857)[PLA2G2E](http://www.ncbi.nlm.nih.gov/gene/?term=30814)(23.000) [AKR1C2](http://www.ncbi.nlm.nih.gov/gene/?term=1646)(23.000)[PPARG](http://www.ncbi.nlm.nih.gov/gene/?term=5468)(23.000)[MPO](http://www.ncbi.nlm.nih.gov/gene/?term=4353)(23.000)[DDC](http://www.ncbi.nlm.nih.gov/gene/?term=1644)(23.000)[IKBKB](http://www.ncbi.nlm.nih.gov/gene/?term=3551)(23.000) [CHUK](http://www.ncbi.nlm.nih.gov/gene/?term=1147)(23.000) |
| [Eugenone](http://www.megabionet.org/tcmid/ingredient/7523/) | [GGCX](http://www.ncbi.nlm.nih.gov/gene/?term=2677)(80.882)[PTGS1](http://www.ncbi.nlm.nih.gov/gene/?term=5742)(80.882)[PTGS2](http://www.ncbi.nlm.nih.gov/gene/?term=5743)(80.882)[SERPINB7](http://www.ncbi.nlm.nih.gov/gene/?term=8710)(55.444)[PDE3A](http://www.ncbi.nlm.nih.gov/gene/?term=5139)(23.000) [NDRG2](http://www.ncbi.nlm.nih.gov/gene/?term=57447)(22.373)[NAMPT](http://www.ncbi.nlm.nih.gov/gene/?term=10135)(22.373)[RGCC](http://www.ncbi.nlm.nih.gov/gene/?term=28984)(22.373)[FNDC5](http://www.ncbi.nlm.nih.gov/gene/?term=252995)(22.373)[AVPR1A](http://www.ncbi.nlm.nih.gov/gene/?term=552)(22.373) [METRNL](http://www.ncbi.nlm.nih.gov/gene/?term=284207)(22.373)[MAPK9](http://www.ncbi.nlm.nih.gov/gene/?term=5601)(22.373)[PTGIS](http://www.ncbi.nlm.nih.gov/gene/?term=5740)(22.373)[HEG1](http://www.ncbi.nlm.nih.gov/gene/?term=57493)(22.373)[AVP](http://www.ncbi.nlm.nih.gov/gene/?term=551)(22.373) [SLC6A4](http://www.ncbi.nlm.nih.gov/gene/?term=6532)(22.373)[BDKRB2](http://www.ncbi.nlm.nih.gov/gene/?term=624)(22.373)[NEUROD2](http://www.ncbi.nlm.nih.gov/gene/?term=4761)(22.373)[INS](http://www.ncbi.nlm.nih.gov/gene/?term=3630)(22.373)[ANAPC2](http://www.ncbi.nlm.nih.gov/gene/?term=29882)(22.373) [WNT11](http://www.ncbi.nlm.nih.gov/gene/?term=7481)(22.373)[ALOX15B](http://www.ncbi.nlm.nih.gov/gene/?term=247)(22.373)[CDC20](http://www.ncbi.nlm.nih.gov/gene/?term=991)(22.373)[NAPRT](http://www.ncbi.nlm.nih.gov/gene/?term=93100)(22.373)[MECOM](http://www.ncbi.nlm.nih.gov/gene/?term=2122)(22.373) [SNCA](http://www.ncbi.nlm.nih.gov/gene/?term=6622)(22.373)[CPLX2](http://www.ncbi.nlm.nih.gov/gene/?term=10814)(22.373)[ALOX15](http://www.ncbi.nlm.nih.gov/gene/?term=246)(22.373)[ANXA1](http://www.ncbi.nlm.nih.gov/gene/?term=301)(22.373)[PRDM16](http://www.ncbi.nlm.nih.gov/gene/?term=63976)(22.373) [CCM2L](http://www.ncbi.nlm.nih.gov/gene/?term=140706)(22.373) |
| [Juglone](http://www.megabionet.org/tcmid/ingredient/11900/) | [HSD17B1](http://www.ncbi.nlm.nih.gov/gene/?term=3292)(80.882)[PROS1](http://www.ncbi.nlm.nih.gov/gene/?term=5627)(48.000)[PROZ](http://www.ncbi.nlm.nih.gov/gene/?term=8858)(48.000)[NQO2](http://www.ncbi.nlm.nih.gov/gene/?term=4835)(48.000)[F7](http://www.ncbi.nlm.nih.gov/gene/?term=2155)(48.000) [F9](http://www.ncbi.nlm.nih.gov/gene/?term=2158)(48.000)[GGCX](http://www.ncbi.nlm.nih.gov/gene/?term=2677)(48.000)[F10](http://www.ncbi.nlm.nih.gov/gene/?term=2159)(48.000)[VKORC1](http://www.ncbi.nlm.nih.gov/gene/?term=79001)(48.000)[PROC](http://www.ncbi.nlm.nih.gov/gene/?term=5624)(48.000) [BGLAP](http://www.ncbi.nlm.nih.gov/gene/?term=632)(48.000)[NQO1](http://www.ncbi.nlm.nih.gov/gene/?term=1728)(48.000)[VKORC1L1](http://www.ncbi.nlm.nih.gov/gene/?term=154807)(48.000)[F2](http://www.ncbi.nlm.nih.gov/gene/?term=2147)(48.000)[DHODH](http://www.ncbi.nlm.nih.gov/gene/?term=1723)(25.857) [ESR1](http://www.ncbi.nlm.nih.gov/gene/?term=2099)(23.000)[CFTR](http://www.ncbi.nlm.nih.gov/gene/?term=1080)(23.000)[AKR1C3](http://www.ncbi.nlm.nih.gov/gene/?term=8644)(22.373)[HSD17B6](http://www.ncbi.nlm.nih.gov/gene/?term=8630)(22.373)[HSD17B2](http://www.ncbi.nlm.nih.gov/gene/?term=3294)(22.373) [CYP11A1](http://www.ncbi.nlm.nih.gov/gene/?term=1583)(22.373)[WNT4](http://www.ncbi.nlm.nih.gov/gene/?term=54361)(22.373)[STAR](http://www.ncbi.nlm.nih.gov/gene/?term=6770)(22.373)[HSD17B8](http://www.ncbi.nlm.nih.gov/gene/?term=7923)(22.373)[DHRS9](http://www.ncbi.nlm.nih.gov/gene/?term=10170)(22.373) [HSD17B3](http://www.ncbi.nlm.nih.gov/gene/?term=3293)(22.373) |
| [Tellimagrandin Ii](http://www.megabionet.org/tcmid/ingredient/32004/) | This compound doesn't have any potential target with score larger than 20. |
| [Corydaline](http://www.megabionet.org/tcmid/ingredient/24291/) | [ACHE](http://www.ncbi.nlm.nih.gov/gene/?term=43)(122.778)[CHRNA2](http://www.ncbi.nlm.nih.gov/gene/?term=1135)(122.778)[HTR3A](http://www.ncbi.nlm.nih.gov/gene/?term=3359)(122.778)[CHRM2](http://www.ncbi.nlm.nih.gov/gene/?term=1129)(122.778)[CYBA](http://www.ncbi.nlm.nih.gov/gene/?term=1535)(80.882) [CHRNA4](http://www.ncbi.nlm.nih.gov/gene/?term=1137)(80.882)[RAC2](http://www.ncbi.nlm.nih.gov/gene/?term=5880)(80.882)[RAC1](http://www.ncbi.nlm.nih.gov/gene/?term=5879)(80.882)[OPRD1](http://www.ncbi.nlm.nih.gov/gene/?term=4985)(80.882)[NCF4](http://www.ncbi.nlm.nih.gov/gene/?term=4689)(80.882) [CHRNA7](http://www.ncbi.nlm.nih.gov/gene/?term=1139)(80.882)[CYBB](http://www.ncbi.nlm.nih.gov/gene/?term=1536)(80.882)[NCF1](http://www.ncbi.nlm.nih.gov/gene/?term=653361)(80.882)[SIGMAR1](http://www.ncbi.nlm.nih.gov/gene/?term=10280)(80.882)[NCF2](http://www.ncbi.nlm.nih.gov/gene/?term=4688)(80.882) [OPRK1](http://www.ncbi.nlm.nih.gov/gene/?term=4986)(80.882)[CHRNA3](http://www.ncbi.nlm.nih.gov/gene/?term=1136)(80.882)[RAC3](http://www.ncbi.nlm.nih.gov/gene/?term=5881)(80.882)[CHRNB2](http://www.ncbi.nlm.nih.gov/gene/?term=1141)(80.882)[SLC6A4](http://www.ncbi.nlm.nih.gov/gene/?term=6532)(80.882) [OPRM1](http://www.ncbi.nlm.nih.gov/gene/?term=4988)(80.882)[PGRMC1](http://www.ncbi.nlm.nih.gov/gene/?term=10857)(80.882)[GRIN3A](http://www.ncbi.nlm.nih.gov/gene/?term=116443)(80.882)[CHRFAM7A](http://www.ncbi.nlm.nih.gov/gene/?term=89832)(80.882)[CHRNB4](http://www.ncbi.nlm.nih.gov/gene/?term=1143)(80.882) [SLC6A2](http://www.ncbi.nlm.nih.gov/gene/?term=6530)(80.882)[TLR4](http://www.ncbi.nlm.nih.gov/gene/?term=7099)(55.444)[ELANE](http://www.ncbi.nlm.nih.gov/gene/?term=1991)(55.444)[CTSG](http://www.ncbi.nlm.nih.gov/gene/?term=1511)(55.444)[SLC18A1](http://www.ncbi.nlm.nih.gov/gene/?term=6570)(55.444) [CEND1](http://www.ncbi.nlm.nih.gov/gene/?term=51286)(55.444)[SLC22A1](http://www.ncbi.nlm.nih.gov/gene/?term=6580)(55.444)[ADRA1B](http://www.ncbi.nlm.nih.gov/gene/?term=147)(55.444)[WLS](http://www.ncbi.nlm.nih.gov/gene/?term=79971)(55.444)[CHRNA9](http://www.ncbi.nlm.nih.gov/gene/?term=55584)(55.444) [CHRM3](http://www.ncbi.nlm.nih.gov/gene/?term=1131)(48.000)[DRD2](http://www.ncbi.nlm.nih.gov/gene/?term=1813)(48.000)[SLC18A2](http://www.ncbi.nlm.nih.gov/gene/?term=6571)(48.000)[BCHE](http://www.ncbi.nlm.nih.gov/gene/?term=590)(48.000)[ADRA1A](http://www.ncbi.nlm.nih.gov/gene/?term=148)(23.000) [HTR2A](http://www.ncbi.nlm.nih.gov/gene/?term=3356)(23.000)[CHRM1](http://www.ncbi.nlm.nih.gov/gene/?term=1128)(23.000)[HTR2B](http://www.ncbi.nlm.nih.gov/gene/?term=3357)(23.000)[ADRA2A](http://www.ncbi.nlm.nih.gov/gene/?term=150)(23.000)[HTR6](http://www.ncbi.nlm.nih.gov/gene/?term=3362)(23.000) [HTR2C](http://www.ncbi.nlm.nih.gov/gene/?term=3358)(23.000)[ADRA1D](http://www.ncbi.nlm.nih.gov/gene/?term=146)(23.000)[CHRM5](http://www.ncbi.nlm.nih.gov/gene/?term=1133)(23.000)[HRH4](http://www.ncbi.nlm.nih.gov/gene/?term=59340)(23.000)[HRH1](http://www.ncbi.nlm.nih.gov/gene/?term=3269)(23.000) [KCNH2](http://www.ncbi.nlm.nih.gov/gene/?term=3757)(23.000)[HRH2](http://www.ncbi.nlm.nih.gov/gene/?term=3274)(23.000)[HTR1A](http://www.ncbi.nlm.nih.gov/gene/?term=3350)(23.000)[ADRA2C](http://www.ncbi.nlm.nih.gov/gene/?term=152)(23.000)[CHRM4](http://www.ncbi.nlm.nih.gov/gene/?term=1132)(23.000) [ADRA2B](http://www.ncbi.nlm.nih.gov/gene/?term=151)(23.000)[ACTN3](http://www.ncbi.nlm.nih.gov/gene/?term=89)(22.373)[GPM6B](http://www.ncbi.nlm.nih.gov/gene/?term=2824)(22.373)[HMOX1](http://www.ncbi.nlm.nih.gov/gene/?term=3162)(22.373)[TAC1](http://www.ncbi.nlm.nih.gov/gene/?term=6863)(22.373) [MC2R](http://www.ncbi.nlm.nih.gov/gene/?term=4158)(22.373)[SLITRK6](http://www.ncbi.nlm.nih.gov/gene/?term=84189)(22.373)[UTS2](http://www.ncbi.nlm.nih.gov/gene/?term=10911)(22.373)[NAV2](http://www.ncbi.nlm.nih.gov/gene/?term=89797)(22.373)[PDE3A](http://www.ncbi.nlm.nih.gov/gene/?term=5139)(22.373) [SCT](http://www.ncbi.nlm.nih.gov/gene/?term=6343)(22.373)[TUSC2](http://www.ncbi.nlm.nih.gov/gene/?term=11334)(22.373)[RHOA](http://www.ncbi.nlm.nih.gov/gene/?term=387)(22.373)[AVPR1A](http://www.ncbi.nlm.nih.gov/gene/?term=552)(22.373)[ADORA2A](http://www.ncbi.nlm.nih.gov/gene/?term=135)(22.373) [HCN4](http://www.ncbi.nlm.nih.gov/gene/?term=10021)(22.373)[PHKG2](http://www.ncbi.nlm.nih.gov/gene/?term=5261)(22.373)[OXT](http://www.ncbi.nlm.nih.gov/gene/?term=5020)(22.373)[CACNA1A](http://www.ncbi.nlm.nih.gov/gene/?term=773)(22.373)[CCL5](http://www.ncbi.nlm.nih.gov/gene/?term=6352)(22.373) [APLP1](http://www.ncbi.nlm.nih.gov/gene/?term=333)(22.373)[LAMP1](http://www.ncbi.nlm.nih.gov/gene/?term=3916)(22.373)[PPP1R9B](http://www.ncbi.nlm.nih.gov/gene/?term=84687)(22.373)[YWHAE](http://www.ncbi.nlm.nih.gov/gene/?term=7531)(22.373)[SUMO1](http://www.ncbi.nlm.nih.gov/gene/?term=7341)(22.373) [HTR1B](http://www.ncbi.nlm.nih.gov/gene/?term=3351)(22.373)[CNR2](http://www.ncbi.nlm.nih.gov/gene/?term=1269)(22.373)[NOXA1](http://www.ncbi.nlm.nih.gov/gene/?term=10811)(22.373)[MC1R](http://www.ncbi.nlm.nih.gov/gene/?term=4157)(22.373)[MC4R](http://www.ncbi.nlm.nih.gov/gene/?term=4160)(22.373) [IL1B](http://www.ncbi.nlm.nih.gov/gene/?term=3553)(22.373)[AGT](http://www.ncbi.nlm.nih.gov/gene/?term=183)(22.373)[CRHBP](http://www.ncbi.nlm.nih.gov/gene/?term=1393)(22.373)[F2R](http://www.ncbi.nlm.nih.gov/gene/?term=2149)(22.373)[MTNR1B](http://www.ncbi.nlm.nih.gov/gene/?term=4544)(22.373) [FGF10](http://www.ncbi.nlm.nih.gov/gene/?term=2255)(22.373)[EGR1](http://www.ncbi.nlm.nih.gov/gene/?term=1958)(22.373)[NOXO1](http://www.ncbi.nlm.nih.gov/gene/?term=124056)(22.373)[ADAM8](http://www.ncbi.nlm.nih.gov/gene/?term=101)(22.373)[SLC44A4](http://www.ncbi.nlm.nih.gov/gene/?term=80736)(22.373) [FLOT1](http://www.ncbi.nlm.nih.gov/gene/?term=10211)(22.373)[NPY5R](http://www.ncbi.nlm.nih.gov/gene/?term=4889)(22.373)[VCP](http://www.ncbi.nlm.nih.gov/gene/?term=7415)(22.373)[GHRL](http://www.ncbi.nlm.nih.gov/gene/?term=51738)(22.373)[TBR1](http://www.ncbi.nlm.nih.gov/gene/?term=10716)(22.373) [PTPRO](http://www.ncbi.nlm.nih.gov/gene/?term=5800)(22.373)[OXTR](http://www.ncbi.nlm.nih.gov/gene/?term=5021)(22.373)[PAX2](http://www.ncbi.nlm.nih.gov/gene/?term=5076)(22.373)[CACNA1C](http://www.ncbi.nlm.nih.gov/gene/?term=775)(22.373)[F2RL1](http://www.ncbi.nlm.nih.gov/gene/?term=2150)(22.373) [UTS2R](http://www.ncbi.nlm.nih.gov/gene/?term=2837)(22.373)[ACVR2A](http://www.ncbi.nlm.nih.gov/gene/?term=92)(22.373)[RAPGEF2](http://www.ncbi.nlm.nih.gov/gene/?term=9693)(22.373)[TIRAP](http://www.ncbi.nlm.nih.gov/gene/?term=114609)(22.373)[AIF1](http://www.ncbi.nlm.nih.gov/gene/?term=199)(22.373) [GHRH](http://www.ncbi.nlm.nih.gov/gene/?term=2691)(22.373)[HCRT](http://www.ncbi.nlm.nih.gov/gene/?term=3060)(22.373)[RAB3B](http://www.ncbi.nlm.nih.gov/gene/?term=5865)(22.373)[CNR1](http://www.ncbi.nlm.nih.gov/gene/?term=1268)(22.373)[HIF1A](http://www.ncbi.nlm.nih.gov/gene/?term=3091)(22.373) [INS](http://www.ncbi.nlm.nih.gov/gene/?term=3630)(22.373)[ACE](http://www.ncbi.nlm.nih.gov/gene/?term=1636)(22.373)[SLC18A3](http://www.ncbi.nlm.nih.gov/gene/?term=6572)(22.373)[BNIP3](http://www.ncbi.nlm.nih.gov/gene/?term=664)(22.373)[DRD4](http://www.ncbi.nlm.nih.gov/gene/?term=1815)(22.373) [CRH](http://www.ncbi.nlm.nih.gov/gene/?term=1392)(22.373)[GLI3](http://www.ncbi.nlm.nih.gov/gene/?term=2737)(22.373)[PLXNB3](http://www.ncbi.nlm.nih.gov/gene/?term=5365)(22.373)[CAV3](http://www.ncbi.nlm.nih.gov/gene/?term=859)(22.373)[NEFL](http://www.ncbi.nlm.nih.gov/gene/?term=4747)(22.373) [UCN2](http://www.ncbi.nlm.nih.gov/gene/?term=90226)(22.373)[IL6](http://www.ncbi.nlm.nih.gov/gene/?term=3569)(22.373)[RNF207](http://www.ncbi.nlm.nih.gov/gene/?term=388591)(22.373)[TACR2](http://www.ncbi.nlm.nih.gov/gene/?term=6865)(22.373)[PTGS2](http://www.ncbi.nlm.nih.gov/gene/?term=5743)(22.373) [NRP1](http://www.ncbi.nlm.nih.gov/gene/?term=8829)(22.373)[NPPA](http://www.ncbi.nlm.nih.gov/gene/?term=4878)(22.373)[MC5R](http://www.ncbi.nlm.nih.gov/gene/?term=4161)(22.373)[AQP1](http://www.ncbi.nlm.nih.gov/gene/?term=358)(22.373)[MC3R](http://www.ncbi.nlm.nih.gov/gene/?term=4159)(22.373) [TAC4](http://www.ncbi.nlm.nih.gov/gene/?term=255061)(22.373)[TNR](http://www.ncbi.nlm.nih.gov/gene/?term=7143)(22.373)[NOS3](http://www.ncbi.nlm.nih.gov/gene/?term=4846)(22.373)[EPHB1](http://www.ncbi.nlm.nih.gov/gene/?term=2047)(22.373)[SNCA](http://www.ncbi.nlm.nih.gov/gene/?term=6622)(22.373) [DRD1](http://www.ncbi.nlm.nih.gov/gene/?term=1812)(22.373)[PDE2A](http://www.ncbi.nlm.nih.gov/gene/?term=5138)(22.373)[CD34](http://www.ncbi.nlm.nih.gov/gene/?term=947)(22.373)[KCNMA1](http://www.ncbi.nlm.nih.gov/gene/?term=3778)(22.373)[POU4F3](http://www.ncbi.nlm.nih.gov/gene/?term=5459)(22.373) [CBFA2T3](http://www.ncbi.nlm.nih.gov/gene/?term=863)(22.373)[CALCA](http://www.ncbi.nlm.nih.gov/gene/?term=796)(22.373)[PINK1](http://www.ncbi.nlm.nih.gov/gene/?term=65018)(22.373)[NOS1](http://www.ncbi.nlm.nih.gov/gene/?term=4842)(22.373)[TREM1](http://www.ncbi.nlm.nih.gov/gene/?term=54210)(22.373) [GJA5](http://www.ncbi.nlm.nih.gov/gene/?term=2702)(22.373)[PDGFB](http://www.ncbi.nlm.nih.gov/gene/?term=5155)(22.373)[CARTPT](http://www.ncbi.nlm.nih.gov/gene/?term=9607)(22.373)[NEFH](http://www.ncbi.nlm.nih.gov/gene/?term=4744)(22.373)[ATP8B1](http://www.ncbi.nlm.nih.gov/gene/?term=5205)(22.373) [KISS1](http://www.ncbi.nlm.nih.gov/gene/?term=3814)(22.373)[GRM7](http://www.ncbi.nlm.nih.gov/gene/?term=2917)(22.373)[FOS](http://www.ncbi.nlm.nih.gov/gene/?term=2353)(22.373)[CRHR2](http://www.ncbi.nlm.nih.gov/gene/?term=1395)(22.373)[GRIN1](http://www.ncbi.nlm.nih.gov/gene/?term=2902)(22.373) [HCN2](http://www.ncbi.nlm.nih.gov/gene/?term=610)(22.373) |
| [Epicatechin](http://www.megabionet.org/tcmid/ingredient/6853/) | [SEC14L3](http://www.ncbi.nlm.nih.gov/gene/?term=266629)(48.000)[SOAT1](http://www.ncbi.nlm.nih.gov/gene/?term=6646)(48.000)[MTTP](http://www.ncbi.nlm.nih.gov/gene/?term=4547)(48.000)[CNR2](http://www.ncbi.nlm.nih.gov/gene/?term=1269)(48.000)[PPP2CA](http://www.ncbi.nlm.nih.gov/gene/?term=5515)(48.000) [PRKCA](http://www.ncbi.nlm.nih.gov/gene/?term=5578)(48.000)[NR1I2](http://www.ncbi.nlm.nih.gov/gene/?term=8856)(48.000)[CNR1](http://www.ncbi.nlm.nih.gov/gene/?term=1268)(48.000)[ALOX5](http://www.ncbi.nlm.nih.gov/gene/?term=240)(48.000)[PPP2CB](http://www.ncbi.nlm.nih.gov/gene/?term=5516)(48.000) [SEC14L2](http://www.ncbi.nlm.nih.gov/gene/?term=23541)(48.000)[DGKA](http://www.ncbi.nlm.nih.gov/gene/?term=1606)(48.000)[PRKCB](http://www.ncbi.nlm.nih.gov/gene/?term=5579)(48.000)[SOAT2](http://www.ncbi.nlm.nih.gov/gene/?term=8435)(48.000)[SEC14L4](http://www.ncbi.nlm.nih.gov/gene/?term=284904)(48.000) |
| [Toralactone](http://www.megabionet.org/tcmid/ingredient/21439/) | [SEC14L3](http://www.ncbi.nlm.nih.gov/gene/?term=266629)(23.000)[PPP2CA](http://www.ncbi.nlm.nih.gov/gene/?term=5515)(23.000)[PRKCA](http://www.ncbi.nlm.nih.gov/gene/?term=5578)(23.000)[NR1I2](http://www.ncbi.nlm.nih.gov/gene/?term=8856)(23.000)[ALOX5](http://www.ncbi.nlm.nih.gov/gene/?term=240)(23.000) [PPP2CB](http://www.ncbi.nlm.nih.gov/gene/?term=5516)(23.000)[SEC14L2](http://www.ncbi.nlm.nih.gov/gene/?term=23541)(23.000)[DGKA](http://www.ncbi.nlm.nih.gov/gene/?term=1606)(23.000)[PRKCB](http://www.ncbi.nlm.nih.gov/gene/?term=5579)(23.000)[SEC14L4](http://www.ncbi.nlm.nih.gov/gene/?term=284904)(23.000) |
| [Isoamyl Alcohol](http://www.megabionet.org/tcmid/ingredient/11215/) | [REN](http://www.ncbi.nlm.nih.gov/gene/?term=5972)(known target in DrugBank)[CACNB1](http://www.ncbi.nlm.nih.gov/gene/?term=782)(373.000)[GRIA4](http://www.ncbi.nlm.nih.gov/gene/?term=2893)(373.000)[GABRA2](http://www.ncbi.nlm.nih.gov/gene/?term=2555)(373.000)[CACNG2](http://www.ncbi.nlm.nih.gov/gene/?term=10369)(373.000) [GABRD](http://www.ncbi.nlm.nih.gov/gene/?term=2563)(373.000)[CHRNA4](http://www.ncbi.nlm.nih.gov/gene/?term=1137)(373.000)[CHRNB3](http://www.ncbi.nlm.nih.gov/gene/?term=1142)(373.000)[GABRB1](http://www.ncbi.nlm.nih.gov/gene/?term=2560)(373.000)[GRIA1](http://www.ncbi.nlm.nih.gov/gene/?term=2890)(373.000) [GLRA2](http://www.ncbi.nlm.nih.gov/gene/?term=2742)(373.000)[KCNJ3](http://www.ncbi.nlm.nih.gov/gene/?term=3760)(373.000)[GABRG3](http://www.ncbi.nlm.nih.gov/gene/?term=2567)(373.000)[VCAM1](http://www.ncbi.nlm.nih.gov/gene/?term=7412)(373.000)[CHRNA7](http://www.ncbi.nlm.nih.gov/gene/?term=1139)(373.000) [KCNJ6](http://www.ncbi.nlm.nih.gov/gene/?term=3763)(373.000)[GABRE](http://www.ncbi.nlm.nih.gov/gene/?term=2564)(373.000)[GABRA3](http://www.ncbi.nlm.nih.gov/gene/?term=2556)(373.000)[SLC29A1](http://www.ncbi.nlm.nih.gov/gene/?term=2030)(373.000)[CACNA1S](http://www.ncbi.nlm.nih.gov/gene/?term=779)(373.000) [GABRG1](http://www.ncbi.nlm.nih.gov/gene/?term=2565)(373.000)[GABRP](http://www.ncbi.nlm.nih.gov/gene/?term=2568)(373.000)[CHRNA10](http://www.ncbi.nlm.nih.gov/gene/?term=57053)(373.000)[GRIA2](http://www.ncbi.nlm.nih.gov/gene/?term=2891)(373.000)[GABRA4](http://www.ncbi.nlm.nih.gov/gene/?term=2557)(373.000) [CHRNA3](http://www.ncbi.nlm.nih.gov/gene/?term=1136)(373.000)[GABRB2](http://www.ncbi.nlm.nih.gov/gene/?term=2561)(373.000)[CACNA1C](http://www.ncbi.nlm.nih.gov/gene/?term=775)(373.000)[CHRNB2](http://www.ncbi.nlm.nih.gov/gene/?term=1141)(373.000)[CACNA1D](http://www.ncbi.nlm.nih.gov/gene/?term=776)(373.000) [HTR3D](http://www.ncbi.nlm.nih.gov/gene/?term=200909)(373.000)[GABRQ](http://www.ncbi.nlm.nih.gov/gene/?term=55879)(373.000)[CHRNA2](http://www.ncbi.nlm.nih.gov/gene/?term=1135)(373.000)[CHRNA6](http://www.ncbi.nlm.nih.gov/gene/?term=8973)(373.000)[GABRB3](http://www.ncbi.nlm.nih.gov/gene/?term=2562)(373.000) [KCNJ5](http://www.ncbi.nlm.nih.gov/gene/?term=3762)(373.000)[GRIN3A](http://www.ncbi.nlm.nih.gov/gene/?term=116443)(373.000)[HTR3B](http://www.ncbi.nlm.nih.gov/gene/?term=9177)(373.000)[GRIA3](http://www.ncbi.nlm.nih.gov/gene/?term=2892)(373.000)[GABRA5](http://www.ncbi.nlm.nih.gov/gene/?term=2558)(373.000) [SLC29A2](http://www.ncbi.nlm.nih.gov/gene/?term=3177)(373.000)[HTR3E](http://www.ncbi.nlm.nih.gov/gene/?term=285242)(373.000)[GABRA6](http://www.ncbi.nlm.nih.gov/gene/?term=2559)(373.000)[HTR3A](http://www.ncbi.nlm.nih.gov/gene/?term=3359)(373.000)[GABRA1](http://www.ncbi.nlm.nih.gov/gene/?term=2554)(373.000) [CHRNA9](http://www.ncbi.nlm.nih.gov/gene/?term=55584)(373.000)[CHRFAM7A](http://www.ncbi.nlm.nih.gov/gene/?term=89832)(373.000)[CHRNA5](http://www.ncbi.nlm.nih.gov/gene/?term=1138)(373.000)[CHRNB4](http://www.ncbi.nlm.nih.gov/gene/?term=1143)(373.000)[KCNJ9](http://www.ncbi.nlm.nih.gov/gene/?term=3765)(373.000) [HTR3C](http://www.ncbi.nlm.nih.gov/gene/?term=170572)(373.000)[GLRA1](http://www.ncbi.nlm.nih.gov/gene/?term=2741)(373.000)[CACNG1](http://www.ncbi.nlm.nih.gov/gene/?term=786)(373.000)[GLRA3](http://www.ncbi.nlm.nih.gov/gene/?term=8001)(114.143)[ADAM8](http://www.ncbi.nlm.nih.gov/gene/?term=101)(55.444) [SLC28A3](http://www.ncbi.nlm.nih.gov/gene/?term=64078)(55.444)[ADRA1B](http://www.ncbi.nlm.nih.gov/gene/?term=147)(55.444)[FOXL2](http://www.ncbi.nlm.nih.gov/gene/?term=668)(55.444)[DMTN](http://www.ncbi.nlm.nih.gov/gene/?term=2039)(55.444)[KCNMA1](http://www.ncbi.nlm.nih.gov/gene/?term=3778)(55.444) [CACNG3](http://www.ncbi.nlm.nih.gov/gene/?term=10368)(25.857)[GLRA4](http://www.ncbi.nlm.nih.gov/gene/?term=441509)(25.857)[GABRG2](http://www.ncbi.nlm.nih.gov/gene/?term=2566)(25.857)[HMGA2](http://www.ncbi.nlm.nih.gov/gene/?term=8091)(22.373)[CACNA1H](http://www.ncbi.nlm.nih.gov/gene/?term=8912)(22.373) [SLITRK6](http://www.ncbi.nlm.nih.gov/gene/?term=84189)(22.373)[UTS2](http://www.ncbi.nlm.nih.gov/gene/?term=10911)(22.373)[NAV2](http://www.ncbi.nlm.nih.gov/gene/?term=89797)(22.373)[P2RX2](http://www.ncbi.nlm.nih.gov/gene/?term=22953)(22.373)[HTR2A](http://www.ncbi.nlm.nih.gov/gene/?term=3356)(22.373) [AVPR1A](http://www.ncbi.nlm.nih.gov/gene/?term=552)(22.373)[ADORA2A](http://www.ncbi.nlm.nih.gov/gene/?term=135)(22.373)[CACNA1A](http://www.ncbi.nlm.nih.gov/gene/?term=773)(22.373)[ANK2](http://www.ncbi.nlm.nih.gov/gene/?term=287)(22.373)[ZP4](http://www.ncbi.nlm.nih.gov/gene/?term=57829)(22.373) [SUMO1](http://www.ncbi.nlm.nih.gov/gene/?term=7341)(22.373)[CNR2](http://www.ncbi.nlm.nih.gov/gene/?term=1269)(22.373)[MTNR1B](http://www.ncbi.nlm.nih.gov/gene/?term=4544)(22.373)[SCN5A](http://www.ncbi.nlm.nih.gov/gene/?term=6331)(22.373)[MAOB](http://www.ncbi.nlm.nih.gov/gene/?term=4129)(22.373) [GLRB](http://www.ncbi.nlm.nih.gov/gene/?term=2743)(22.373)[P2RX3](http://www.ncbi.nlm.nih.gov/gene/?term=5024)(22.373)[SLC44A4](http://www.ncbi.nlm.nih.gov/gene/?term=80736)(22.373)[FLOT1](http://www.ncbi.nlm.nih.gov/gene/?term=10211)(22.373)[NPY5R](http://www.ncbi.nlm.nih.gov/gene/?term=4889)(22.373) [MSN](http://www.ncbi.nlm.nih.gov/gene/?term=4478)(22.373)[GHRL](http://www.ncbi.nlm.nih.gov/gene/?term=51738)(22.373)[TBR1](http://www.ncbi.nlm.nih.gov/gene/?term=10716)(22.373)[ADRA1D](http://www.ncbi.nlm.nih.gov/gene/?term=146)(22.373)[DRD2](http://www.ncbi.nlm.nih.gov/gene/?term=1813)(22.373) [AVP](http://www.ncbi.nlm.nih.gov/gene/?term=551)(22.373)[PAX2](http://www.ncbi.nlm.nih.gov/gene/?term=5076)(22.373)[AOC1](http://www.ncbi.nlm.nih.gov/gene/?term=26)(22.373)[MAOA](http://www.ncbi.nlm.nih.gov/gene/?term=4128)(22.373)[UTS2R](http://www.ncbi.nlm.nih.gov/gene/?term=2837)(22.373) [ADIRF](http://www.ncbi.nlm.nih.gov/gene/?term=10974)(22.373)[GHRH](http://www.ncbi.nlm.nih.gov/gene/?term=2691)(22.373)[HCRT](http://www.ncbi.nlm.nih.gov/gene/?term=3060)(22.373)[RAB3B](http://www.ncbi.nlm.nih.gov/gene/?term=5865)(22.373)[KCNE5](http://www.ncbi.nlm.nih.gov/gene/?term=23630)(22.373) [CNR1](http://www.ncbi.nlm.nih.gov/gene/?term=1268)(22.373)[SLC18A3](http://www.ncbi.nlm.nih.gov/gene/?term=6572)(22.373)[EZR](http://www.ncbi.nlm.nih.gov/gene/?term=7430)(22.373)[DRD4](http://www.ncbi.nlm.nih.gov/gene/?term=1815)(22.373)[CRH](http://www.ncbi.nlm.nih.gov/gene/?term=1392)(22.373) [GLI3](http://www.ncbi.nlm.nih.gov/gene/?term=2737)(22.373)[CAV3](http://www.ncbi.nlm.nih.gov/gene/?term=859)(22.373)[SLC9A6](http://www.ncbi.nlm.nih.gov/gene/?term=10479)(22.373)[AOC3](http://www.ncbi.nlm.nih.gov/gene/?term=8639)(22.373)[NCOA1](http://www.ncbi.nlm.nih.gov/gene/?term=8648)(22.373) [AOC2](http://www.ncbi.nlm.nih.gov/gene/?term=314)(22.373)[IL6](http://www.ncbi.nlm.nih.gov/gene/?term=3569)(22.373)[RNF207](http://www.ncbi.nlm.nih.gov/gene/?term=388591)(22.373)[ROCK1](http://www.ncbi.nlm.nih.gov/gene/?term=6093)(22.373)[KCNA5](http://www.ncbi.nlm.nih.gov/gene/?term=3741)(22.373) [TACR2](http://www.ncbi.nlm.nih.gov/gene/?term=6865)(22.373)[NRP1](http://www.ncbi.nlm.nih.gov/gene/?term=8829)(22.373)[NPPA](http://www.ncbi.nlm.nih.gov/gene/?term=4878)(22.373)[KMT2A](http://www.ncbi.nlm.nih.gov/gene/?term=4297)(22.373)[TNR](http://www.ncbi.nlm.nih.gov/gene/?term=7143)(22.373) [ZP3](http://www.ncbi.nlm.nih.gov/gene/?term=7784)(22.373)[PLCB1](http://www.ncbi.nlm.nih.gov/gene/?term=23236)(22.373)[EPHB1](http://www.ncbi.nlm.nih.gov/gene/?term=2047)(22.373)[DRD1](http://www.ncbi.nlm.nih.gov/gene/?term=1812)(22.373)[EEA1](http://www.ncbi.nlm.nih.gov/gene/?term=8411)(22.373) [POU4F3](http://www.ncbi.nlm.nih.gov/gene/?term=5459)(22.373)[CALCA](http://www.ncbi.nlm.nih.gov/gene/?term=796)(22.373)[PINK1](http://www.ncbi.nlm.nih.gov/gene/?term=65018)(22.373)[KCNQ1](http://www.ncbi.nlm.nih.gov/gene/?term=3784)(22.373)[ICAM1](http://www.ncbi.nlm.nih.gov/gene/?term=3383)(22.373) [CARTPT](http://www.ncbi.nlm.nih.gov/gene/?term=9607)(22.373)[ATP8B1](http://www.ncbi.nlm.nih.gov/gene/?term=5205)(22.373)[KISS1](http://www.ncbi.nlm.nih.gov/gene/?term=3814)(22.373)[GRM7](http://www.ncbi.nlm.nih.gov/gene/?term=2917)(22.373)[FOS](http://www.ncbi.nlm.nih.gov/gene/?term=2353)(22.373) [GRIN1](http://www.ncbi.nlm.nih.gov/gene/?term=2902)(22.373) |
| [Camphor](http://www.megabionet.org/tcmid/ingredient/3048/) | [ESRRG](http://www.ncbi.nlm.nih.gov/gene/?term=2104)(48.000)[COX6C](http://www.ncbi.nlm.nih.gov/gene/?term=1345)(48.000)[COX5B](http://www.ncbi.nlm.nih.gov/gene/?term=1329)(48.000)[COX7C](http://www.ncbi.nlm.nih.gov/gene/?term=1350)(48.000)[COX1](http://www.ncbi.nlm.nih.gov/gene/?term=4512)(48.000) [AKR1C2](http://www.ncbi.nlm.nih.gov/gene/?term=1646)(48.000)[COX5A](http://www.ncbi.nlm.nih.gov/gene/?term=9377)(48.000)[COX3](http://www.ncbi.nlm.nih.gov/gene/?term=4514)(48.000)[AR](http://www.ncbi.nlm.nih.gov/gene/?term=367)(48.000)[COX7A1](http://www.ncbi.nlm.nih.gov/gene/?term=1346)(48.000) [FECH](http://www.ncbi.nlm.nih.gov/gene/?term=2235)(48.000)[COX4I1](http://www.ncbi.nlm.nih.gov/gene/?term=1327)(48.000)[PLA2G1B](http://www.ncbi.nlm.nih.gov/gene/?term=5319)(48.000)[COX6A2](http://www.ncbi.nlm.nih.gov/gene/?term=1339)(48.000)[ADH1C](http://www.ncbi.nlm.nih.gov/gene/?term=126)(48.000) [COX6B1](http://www.ncbi.nlm.nih.gov/gene/?term=1340)(48.000)[FABP6](http://www.ncbi.nlm.nih.gov/gene/?term=2172)(48.000)[CES1](http://www.ncbi.nlm.nih.gov/gene/?term=1066)(48.000)[COX7B](http://www.ncbi.nlm.nih.gov/gene/?term=1349)(48.000)[COX2](http://www.ncbi.nlm.nih.gov/gene/?term=4513)(48.000) [COX8A](http://www.ncbi.nlm.nih.gov/gene/?term=1351)(48.000)[NR1H4](http://www.ncbi.nlm.nih.gov/gene/?term=9971)(48.000) |
| [Commiferin](http://www.megabionet.org/tcmid/ingredient/3943/) | [HMGCR](http://www.ncbi.nlm.nih.gov/gene/?term=3156)(80.882)[ITGB2](http://www.ncbi.nlm.nih.gov/gene/?term=3689)(80.882)[ITGAL](http://www.ncbi.nlm.nih.gov/gene/?term=3683)(80.882)[HDAC2](http://www.ncbi.nlm.nih.gov/gene/?term=3066)(80.882)[ATP1A1](http://www.ncbi.nlm.nih.gov/gene/?term=476)(48.000) [HDAC1](http://www.ncbi.nlm.nih.gov/gene/?term=3065)(23.000)[LTK](http://www.ncbi.nlm.nih.gov/gene/?term=4058)(22.373)[SIRT1](http://www.ncbi.nlm.nih.gov/gene/?term=23411)(22.373)[SIX1](http://www.ncbi.nlm.nih.gov/gene/?term=6495)(22.373)[SIX4](http://www.ncbi.nlm.nih.gov/gene/?term=51804)(22.373) [SPI1](http://www.ncbi.nlm.nih.gov/gene/?term=6688)(22.373)[UBE2B](http://www.ncbi.nlm.nih.gov/gene/?term=7320)(22.373)[TP53](http://www.ncbi.nlm.nih.gov/gene/?term=7157)(22.373)[CAMK2A](http://www.ncbi.nlm.nih.gov/gene/?term=815)(22.373)[CRP](http://www.ncbi.nlm.nih.gov/gene/?term=1401)(22.373) [ARID1A](http://www.ncbi.nlm.nih.gov/gene/?term=8289)(22.373)[8-Mar](http://www.ncbi.nlm.nih.gov/gene/?term=220972)(22.373)[INS](http://www.ncbi.nlm.nih.gov/gene/?term=3630)(22.373)[WNT10B](http://www.ncbi.nlm.nih.gov/gene/?term=7480)(22.373)[TIGAR](http://www.ncbi.nlm.nih.gov/gene/?term=57103)(22.373) [PF4](http://www.ncbi.nlm.nih.gov/gene/?term=5196)(22.373)[CAMK2D](http://www.ncbi.nlm.nih.gov/gene/?term=817)(22.373)[IL10](http://www.ncbi.nlm.nih.gov/gene/?term=3586)(22.373)[CDC42](http://www.ncbi.nlm.nih.gov/gene/?term=998)(22.373)[CTNNB1](http://www.ncbi.nlm.nih.gov/gene/?term=1499)(22.373) [EDA](http://www.ncbi.nlm.nih.gov/gene/?term=1896)(22.373)[ADORA1](http://www.ncbi.nlm.nih.gov/gene/?term=134)(22.373)[CLEC4M](http://www.ncbi.nlm.nih.gov/gene/?term=10332)(22.373)[GNAS](http://www.ncbi.nlm.nih.gov/gene/?term=2778)(22.373)[NFX1](http://www.ncbi.nlm.nih.gov/gene/?term=4799)(22.373) |
| [Torachrysone-8-O-Beta-D-(6'-Oxayl)-Glucoside](http://www.megabionet.org/tcmid/ingredient/32078/) | This compound doesn't have any potential target with score larger than 20. |
| [Rheidin C](http://www.megabionet.org/tcmid/ingredient/18743/) | This compound doesn't have any potential target with score larger than 20. |
| [Rugosin B](http://www.megabionet.org/tcmid/ingredient/19040/) | This compound doesn't have any potential target with score larger than 20. |
| [Physcion 8-Glucoside](http://www.megabionet.org/tcmid/ingredient/31802/) | This compound doesn't have any potential target with score larger than 20. |
| [Sennidin A](http://www.megabionet.org/tcmid/ingredient/19731/) | [PPARG](http://www.ncbi.nlm.nih.gov/gene/?term=5468)(23.000)[FPGS](http://www.ncbi.nlm.nih.gov/gene/?term=2356)(23.000)[PTGS1](http://www.ncbi.nlm.nih.gov/gene/?term=5742)(23.000)[PTGS2](http://www.ncbi.nlm.nih.gov/gene/?term=5743)(23.000)[ALOX5](http://www.ncbi.nlm.nih.gov/gene/?term=240)(23.000) [MPL](http://www.ncbi.nlm.nih.gov/gene/?term=4352)(23.000)[TYMS](http://www.ncbi.nlm.nih.gov/gene/?term=7298)(23.000)[APOA2](http://www.ncbi.nlm.nih.gov/gene/?term=336)(22.373)[SHMT1](http://www.ncbi.nlm.nih.gov/gene/?term=6470)(22.373)[SERPINE1](http://www.ncbi.nlm.nih.gov/gene/?term=5054)(22.373) [IL1B](http://www.ncbi.nlm.nih.gov/gene/?term=3553)(22.373)[IL13](http://www.ncbi.nlm.nih.gov/gene/?term=3596)(22.373)[GGH](http://www.ncbi.nlm.nih.gov/gene/?term=8836)(22.373)[SERPINB7](http://www.ncbi.nlm.nih.gov/gene/?term=8710)(22.373) |
| [Isoquercitrin](http://www.megabionet.org/tcmid/ingredient/11639/) | This compound doesn't have any potential target with score larger than 20. |
| [Coptisine](http://www.megabionet.org/tcmid/ingredient/4032/) | This compound doesn't have any potential target with score larger than 20. |
| [Boswellic Acid](http://www.megabionet.org/tcmid/ingredient/24461/) | [SRD5A1](http://www.ncbi.nlm.nih.gov/gene/?term=6715)(48.000)[CYP17A1](http://www.ncbi.nlm.nih.gov/gene/?term=1586)(48.000)[SLC8A1](http://www.ncbi.nlm.nih.gov/gene/?term=6546)(48.000)[F12](http://www.ncbi.nlm.nih.gov/gene/?term=2161)(48.000)[ESR1](http://www.ncbi.nlm.nih.gov/gene/?term=2099)(48.000) [TRPV1](http://www.ncbi.nlm.nih.gov/gene/?term=7442)(48.000)[PTGER1](http://www.ncbi.nlm.nih.gov/gene/?term=5731)(48.000)[PTGER4](http://www.ncbi.nlm.nih.gov/gene/?term=5734)(48.000)[PGR](http://www.ncbi.nlm.nih.gov/gene/?term=5241)(48.000)[AR](http://www.ncbi.nlm.nih.gov/gene/?term=367)(48.000) [NR3C1](http://www.ncbi.nlm.nih.gov/gene/?term=2908)(48.000)[OPRK1](http://www.ncbi.nlm.nih.gov/gene/?term=4986)(48.000)[FADS2](http://www.ncbi.nlm.nih.gov/gene/?term=9415)(48.000)[FADS1](http://www.ncbi.nlm.nih.gov/gene/?term=3992)(48.000)[PTGER2](http://www.ncbi.nlm.nih.gov/gene/?term=5732)(48.000) [PTGS1](http://www.ncbi.nlm.nih.gov/gene/?term=5742)(48.000)[PTGER3](http://www.ncbi.nlm.nih.gov/gene/?term=5733)(48.000)[PTGS2](http://www.ncbi.nlm.nih.gov/gene/?term=5743)(48.000)[ELOVL4](http://www.ncbi.nlm.nih.gov/gene/?term=6785)(48.000)[ANXA1](http://www.ncbi.nlm.nih.gov/gene/?term=301)(48.000) [NR3C2](http://www.ncbi.nlm.nih.gov/gene/?term=4306)(48.000)[ESRRG](http://www.ncbi.nlm.nih.gov/gene/?term=2104)(23.000)[COX6C](http://www.ncbi.nlm.nih.gov/gene/?term=1345)(23.000)[COX5B](http://www.ncbi.nlm.nih.gov/gene/?term=1329)(23.000)[COX7C](http://www.ncbi.nlm.nih.gov/gene/?term=1350)(23.000) [COX1](http://www.ncbi.nlm.nih.gov/gene/?term=4512)(23.000)[AKR1C2](http://www.ncbi.nlm.nih.gov/gene/?term=1646)(23.000)[ADH1B](http://www.ncbi.nlm.nih.gov/gene/?term=125)(23.000)[COX5A](http://www.ncbi.nlm.nih.gov/gene/?term=9377)(23.000)[COX3](http://www.ncbi.nlm.nih.gov/gene/?term=4514)(23.000) [COX7A1](http://www.ncbi.nlm.nih.gov/gene/?term=1346)(23.000)[FECH](http://www.ncbi.nlm.nih.gov/gene/?term=2235)(23.000)[COX4I1](http://www.ncbi.nlm.nih.gov/gene/?term=1327)(23.000)[PLA2G1B](http://www.ncbi.nlm.nih.gov/gene/?term=5319)(23.000)[AKR1C1](http://www.ncbi.nlm.nih.gov/gene/?term=1645)(23.000) [COX6A2](http://www.ncbi.nlm.nih.gov/gene/?term=1339)(23.000)[HMGCR](http://www.ncbi.nlm.nih.gov/gene/?term=3156)(23.000)[ADH1C](http://www.ncbi.nlm.nih.gov/gene/?term=126)(23.000)[COX6B1](http://www.ncbi.nlm.nih.gov/gene/?term=1340)(23.000)[FABP6](http://www.ncbi.nlm.nih.gov/gene/?term=2172)(23.000) [CES1](http://www.ncbi.nlm.nih.gov/gene/?term=1066)(23.000)[COX7B](http://www.ncbi.nlm.nih.gov/gene/?term=1349)(23.000)[COX2](http://www.ncbi.nlm.nih.gov/gene/?term=4513)(23.000)[COX8A](http://www.ncbi.nlm.nih.gov/gene/?term=1351)(23.000)[NR1H4](http://www.ncbi.nlm.nih.gov/gene/?term=9971)(23.000) |
| [1,6-Digalloyl-2-Cinnamoyl-Glucose](http://www.megabionet.org/tcmid/ingredient/5513/) | This compound doesn't have any potential target with score larger than 20. |
| [Vitamin E](http://www.megabionet.org/tcmid/ingredient/23125/) | [FOLR2](http://www.ncbi.nlm.nih.gov/gene/?term=2350)(124.000)[SLC5A6](http://www.ncbi.nlm.nih.gov/gene/?term=8884)(124.000)[PCCA](http://www.ncbi.nlm.nih.gov/gene/?term=5095)(124.000)[MUT](http://www.ncbi.nlm.nih.gov/gene/?term=4594)(124.000)[ACACB](http://www.ncbi.nlm.nih.gov/gene/?term=32)(124.000) [PC](http://www.ncbi.nlm.nih.gov/gene/?term=5091)(124.000)[MTRR](http://www.ncbi.nlm.nih.gov/gene/?term=4552)(124.000)[ACACA](http://www.ncbi.nlm.nih.gov/gene/?term=31)(124.000)[PDXK](http://www.ncbi.nlm.nih.gov/gene/?term=8566)(124.000)[MCCC1](http://www.ncbi.nlm.nih.gov/gene/?term=56922)(124.000) [BLVRB](http://www.ncbi.nlm.nih.gov/gene/?term=645)(124.000)[MCCC2](http://www.ncbi.nlm.nih.gov/gene/?term=64087)(124.000)[MTR](http://www.ncbi.nlm.nih.gov/gene/?term=4548)(124.000)[SLC19A2](http://www.ncbi.nlm.nih.gov/gene/?term=10560)(124.000)[HLCS](http://www.ncbi.nlm.nih.gov/gene/?term=3141)(124.000) [FOLR3](http://www.ncbi.nlm.nih.gov/gene/?term=2352)(124.000)[MMACHC](http://www.ncbi.nlm.nih.gov/gene/?term=25974)(124.000)[TPK1](http://www.ncbi.nlm.nih.gov/gene/?term=27010)(124.000)[RFK](http://www.ncbi.nlm.nih.gov/gene/?term=55312)(124.000)[PCCB](http://www.ncbi.nlm.nih.gov/gene/?term=5096)(124.000) [MMAA](http://www.ncbi.nlm.nih.gov/gene/?term=166785)(124.000)[VDR](http://www.ncbi.nlm.nih.gov/gene/?term=7421)(124.000)[MTHFR](http://www.ncbi.nlm.nih.gov/gene/?term=4524)(124.000)[CREBBP](http://www.ncbi.nlm.nih.gov/gene/?term=1387)(26.373)[MED12](http://www.ncbi.nlm.nih.gov/gene/?term=9968)(26.373) [RXRB](http://www.ncbi.nlm.nih.gov/gene/?term=6257)(26.373)[GOT2](http://www.ncbi.nlm.nih.gov/gene/?term=2806)(26.373)[PRKAB1](http://www.ncbi.nlm.nih.gov/gene/?term=5564)(26.373)[MDH2](http://www.ncbi.nlm.nih.gov/gene/?term=4191)(26.373)[ILK](http://www.ncbi.nlm.nih.gov/gene/?term=3611)(26.373) [HMOX1](http://www.ncbi.nlm.nih.gov/gene/?term=3162)(26.373)[HTR4](http://www.ncbi.nlm.nih.gov/gene/?term=3360)(26.373)[CFH](http://www.ncbi.nlm.nih.gov/gene/?term=3075)(26.373)[SIRT1](http://www.ncbi.nlm.nih.gov/gene/?term=23411)(26.373)[ACVR1](http://www.ncbi.nlm.nih.gov/gene/?term=90)(26.373) [NCOR2](http://www.ncbi.nlm.nih.gov/gene/?term=9612)(26.373)[CCND3](http://www.ncbi.nlm.nih.gov/gene/?term=896)(26.373)[SMARCB1](http://www.ncbi.nlm.nih.gov/gene/?term=6598)(26.373)[NR0B2](http://www.ncbi.nlm.nih.gov/gene/?term=8431)(26.373)[HR](http://www.ncbi.nlm.nih.gov/gene/?term=55806)(26.373) [BRCA1](http://www.ncbi.nlm.nih.gov/gene/?term=672)(26.373)[HTR2A](http://www.ncbi.nlm.nih.gov/gene/?term=3356)(26.373)[STAT1](http://www.ncbi.nlm.nih.gov/gene/?term=6772)(26.373)[ADRB2](http://www.ncbi.nlm.nih.gov/gene/?term=154)(26.373)[NCOR1](http://www.ncbi.nlm.nih.gov/gene/?term=9611)(26.373) [MED1](http://www.ncbi.nlm.nih.gov/gene/?term=5469)(26.373)[MCC](http://www.ncbi.nlm.nih.gov/gene/?term=4163)(26.373)[RUNX1T1](http://www.ncbi.nlm.nih.gov/gene/?term=862)(26.373)[IKBKE](http://www.ncbi.nlm.nih.gov/gene/?term=9641)(26.373)[TP53](http://www.ncbi.nlm.nih.gov/gene/?term=7157)(26.373) [MMAB](http://www.ncbi.nlm.nih.gov/gene/?term=326625)(26.373)[HMGN3](http://www.ncbi.nlm.nih.gov/gene/?term=9324)(26.373)[BAZ1B](http://www.ncbi.nlm.nih.gov/gene/?term=9031)(26.373)[KDM5A](http://www.ncbi.nlm.nih.gov/gene/?term=5927)(26.373)[SMAD3](http://www.ncbi.nlm.nih.gov/gene/?term=4088)(26.373) [YBX1](http://www.ncbi.nlm.nih.gov/gene/?term=4904)(26.373)[CIAO1](http://www.ncbi.nlm.nih.gov/gene/?term=9391)(26.373)[NAT10](http://www.ncbi.nlm.nih.gov/gene/?term=55226)(26.373)[PPP1CA](http://www.ncbi.nlm.nih.gov/gene/?term=5499)(26.373)[GABARAPL2](http://www.ncbi.nlm.nih.gov/gene/?term=11345)(26.373) [ATP4A](http://www.ncbi.nlm.nih.gov/gene/?term=495)(26.373)[HIST2H2AB](http://www.ncbi.nlm.nih.gov/gene/?term=317772)(26.373)[TSC22D1](http://www.ncbi.nlm.nih.gov/gene/?term=8848)(26.373)[MYC](http://www.ncbi.nlm.nih.gov/gene/?term=4609)(26.373)[CSNK2B](http://www.ncbi.nlm.nih.gov/gene/?term=1460)(26.373) [TDG](http://www.ncbi.nlm.nih.gov/gene/?term=6996)(26.373)[LAMA3](http://www.ncbi.nlm.nih.gov/gene/?term=3909)(26.373)[DYNLL1](http://www.ncbi.nlm.nih.gov/gene/?term=8655)(26.373)[GOT1](http://www.ncbi.nlm.nih.gov/gene/?term=2805)(26.373)[RUNX1](http://www.ncbi.nlm.nih.gov/gene/?term=861)(26.373) [RAB1A](http://www.ncbi.nlm.nih.gov/gene/?term=5861)(26.373)[PPP2CA](http://www.ncbi.nlm.nih.gov/gene/?term=5515)(26.373)[SMARCC2](http://www.ncbi.nlm.nih.gov/gene/?term=6601)(26.373)[HSPA5](http://www.ncbi.nlm.nih.gov/gene/?term=3309)(26.373)[ARID1A](http://www.ncbi.nlm.nih.gov/gene/?term=8289)(26.373) [SNX27](http://www.ncbi.nlm.nih.gov/gene/?term=81609)(26.373)[GALNT6](http://www.ncbi.nlm.nih.gov/gene/?term=11226)(26.373)[CHAF1A](http://www.ncbi.nlm.nih.gov/gene/?term=10036)(26.373)[ZBTB16](http://www.ncbi.nlm.nih.gov/gene/?term=7704)(26.373)[ADRB1](http://www.ncbi.nlm.nih.gov/gene/?term=153)(26.373) [DNAJB9](http://www.ncbi.nlm.nih.gov/gene/?term=4189)(26.373)[CSNK2A2](http://www.ncbi.nlm.nih.gov/gene/?term=1459)(26.373)[DIAPH3](http://www.ncbi.nlm.nih.gov/gene/?term=81624)(26.373)[CXXC5](http://www.ncbi.nlm.nih.gov/gene/?term=51523)(26.373)[YWHAZ](http://www.ncbi.nlm.nih.gov/gene/?term=7534)(26.373) [PRKAA2](http://www.ncbi.nlm.nih.gov/gene/?term=5563)(26.373)[NR3C1](http://www.ncbi.nlm.nih.gov/gene/?term=2908)(26.373)[PSMC5](http://www.ncbi.nlm.nih.gov/gene/?term=5705)(26.373)[ACTL6A](http://www.ncbi.nlm.nih.gov/gene/?term=86)(26.373)[SMARCC1](http://www.ncbi.nlm.nih.gov/gene/?term=6599)(26.373) [MED21](http://www.ncbi.nlm.nih.gov/gene/?term=9412)(26.373)[HSPA1L](http://www.ncbi.nlm.nih.gov/gene/?term=3305)(26.373)[JUN](http://www.ncbi.nlm.nih.gov/gene/?term=3725)(26.373)[RXRG](http://www.ncbi.nlm.nih.gov/gene/?term=6258)(26.373)[SYNCRIP](http://www.ncbi.nlm.nih.gov/gene/?term=10492)(26.373) [CDCA7L](http://www.ncbi.nlm.nih.gov/gene/?term=55536)(26.373)[NCOA6](http://www.ncbi.nlm.nih.gov/gene/?term=23054)(26.373)[MED16](http://www.ncbi.nlm.nih.gov/gene/?term=10025)(26.373)[NRIP1](http://www.ncbi.nlm.nih.gov/gene/?term=8204)(26.373)[MMRN2](http://www.ncbi.nlm.nih.gov/gene/?term=79812)(26.373) [CACNA1C](http://www.ncbi.nlm.nih.gov/gene/?term=775)(26.373)[CHCHD2](http://www.ncbi.nlm.nih.gov/gene/?term=51142)(26.373)[KPNA3](http://www.ncbi.nlm.nih.gov/gene/?term=3839)(26.373)[NCOA3](http://www.ncbi.nlm.nih.gov/gene/?term=8202)(26.373)[ARHGAP29](http://www.ncbi.nlm.nih.gov/gene/?term=9411)(26.373) [SMARCD1](http://www.ncbi.nlm.nih.gov/gene/?term=6602)(26.373)[NR1H2](http://www.ncbi.nlm.nih.gov/gene/?term=7376)(26.373)[KCNH2](http://www.ncbi.nlm.nih.gov/gene/?term=3757)(26.373)[PRKAA1](http://www.ncbi.nlm.nih.gov/gene/?term=5562)(26.373)[POU2F1](http://www.ncbi.nlm.nih.gov/gene/?term=5451)(26.373) [SMARCA2](http://www.ncbi.nlm.nih.gov/gene/?term=6595)(26.373)[RB1](http://www.ncbi.nlm.nih.gov/gene/?term=5925)(26.373)[MED6](http://www.ncbi.nlm.nih.gov/gene/?term=10001)(26.373)[RPL31](http://www.ncbi.nlm.nih.gov/gene/?term=6160)(26.373)[MCF2L2](http://www.ncbi.nlm.nih.gov/gene/?term=23101)(26.373) [NCOA1](http://www.ncbi.nlm.nih.gov/gene/?term=8648)(26.373)[DYNC1H1](http://www.ncbi.nlm.nih.gov/gene/?term=1778)(26.373)[ORM1](http://www.ncbi.nlm.nih.gov/gene/?term=5004)(26.373)[KPNA2](http://www.ncbi.nlm.nih.gov/gene/?term=3838)(26.373)[GTF2B](http://www.ncbi.nlm.nih.gov/gene/?term=2959)(26.373) [TRRAP](http://www.ncbi.nlm.nih.gov/gene/?term=8295)(26.373)[CERS2](http://www.ncbi.nlm.nih.gov/gene/?term=29956)(26.373)[THRAP3](http://www.ncbi.nlm.nih.gov/gene/?term=9967)(26.373)[HSPD1](http://www.ncbi.nlm.nih.gov/gene/?term=3329)(26.373)[USP9Y](http://www.ncbi.nlm.nih.gov/gene/?term=8287)(26.373) [NCOA2](http://www.ncbi.nlm.nih.gov/gene/?term=10499)(26.373)[BAG1](http://www.ncbi.nlm.nih.gov/gene/?term=573)(26.373)[PRKCSH](http://www.ncbi.nlm.nih.gov/gene/?term=5589)(26.373)[SUPT16H](http://www.ncbi.nlm.nih.gov/gene/?term=11198)(26.373)[ATXN1](http://www.ncbi.nlm.nih.gov/gene/?term=6310)(26.373) [LSM8](http://www.ncbi.nlm.nih.gov/gene/?term=51691)(26.373)[HTR3A](http://www.ncbi.nlm.nih.gov/gene/?term=3359)(26.373)[CMYA5](http://www.ncbi.nlm.nih.gov/gene/?term=202333)(26.373)[CDK7](http://www.ncbi.nlm.nih.gov/gene/?term=1022)(26.373)[POU1F1](http://www.ncbi.nlm.nih.gov/gene/?term=5449)(26.373) [MED13](http://www.ncbi.nlm.nih.gov/gene/?term=9969)(26.373)[PRCP](http://www.ncbi.nlm.nih.gov/gene/?term=5547)(26.373)[MED14](http://www.ncbi.nlm.nih.gov/gene/?term=9282)(26.373)[MED24](http://www.ncbi.nlm.nih.gov/gene/?term=9862)(26.373)[ANXA1](http://www.ncbi.nlm.nih.gov/gene/?term=301)(26.373) [SMARCE1](http://www.ncbi.nlm.nih.gov/gene/?term=6605)(26.373)[PCBD1](http://www.ncbi.nlm.nih.gov/gene/?term=5092)(26.373)[SMARCA4](http://www.ncbi.nlm.nih.gov/gene/?term=6597)(26.373)[AKR1B10](http://www.ncbi.nlm.nih.gov/gene/?term=57016)(26.373)[ELL3](http://www.ncbi.nlm.nih.gov/gene/?term=80237)(26.373) [RXRA](http://www.ncbi.nlm.nih.gov/gene/?term=6256)(26.373)[CSNK2A1](http://www.ncbi.nlm.nih.gov/gene/?term=1457)(26.373)[HDAC2](http://www.ncbi.nlm.nih.gov/gene/?term=3066)(26.373)[DDX52](http://www.ncbi.nlm.nih.gov/gene/?term=11056)(26.373)[WRN](http://www.ncbi.nlm.nih.gov/gene/?term=7486)(26.373) [GABARAPL1](http://www.ncbi.nlm.nih.gov/gene/?term=23710)(26.373)[SNW1](http://www.ncbi.nlm.nih.gov/gene/?term=22938)(26.373)[NKD2](http://www.ncbi.nlm.nih.gov/gene/?term=85409)(26.373)[CHGB](http://www.ncbi.nlm.nih.gov/gene/?term=1114)(26.373)[FOS](http://www.ncbi.nlm.nih.gov/gene/?term=2353)(26.373) [TRIM24](http://www.ncbi.nlm.nih.gov/gene/?term=8805)(26.373)[TOP2B](http://www.ncbi.nlm.nih.gov/gene/?term=7155)(26.373)[FGR](http://www.ncbi.nlm.nih.gov/gene/?term=2268)(26.373) |
| [D-Limonene](http://www.megabionet.org/tcmid/ingredient/12837/) | [KCND1](http://www.ncbi.nlm.nih.gov/gene/?term=3750)(80.882)[KCNA3](http://www.ncbi.nlm.nih.gov/gene/?term=3738)(80.882)[PRKAB1](http://www.ncbi.nlm.nih.gov/gene/?term=5564)(80.882)[ADH1A](http://www.ncbi.nlm.nih.gov/gene/?term=124)(80.882)[KCNA10](http://www.ncbi.nlm.nih.gov/gene/?term=3744)(80.882) [GAMT](http://www.ncbi.nlm.nih.gov/gene/?term=2593)(80.882)[KCNC3](http://www.ncbi.nlm.nih.gov/gene/?term=3748)(80.882)[KCNA1](http://www.ncbi.nlm.nih.gov/gene/?term=3736)(80.882)[KCNA2](http://www.ncbi.nlm.nih.gov/gene/?term=3737)(80.882)[TPO](http://www.ncbi.nlm.nih.gov/gene/?term=7173)(80.882) [CAT](http://www.ncbi.nlm.nih.gov/gene/?term=847)(80.882)[KCNB1](http://www.ncbi.nlm.nih.gov/gene/?term=3745)(80.882)[ADH1B](http://www.ncbi.nlm.nih.gov/gene/?term=125)(80.882)[DLG4](http://www.ncbi.nlm.nih.gov/gene/?term=1742)(80.882)[KCNC2](http://www.ncbi.nlm.nih.gov/gene/?term=3747)(80.882) [KCNC1](http://www.ncbi.nlm.nih.gov/gene/?term=3746)(80.882)[ADH1C](http://www.ncbi.nlm.nih.gov/gene/?term=126)(80.882)[KCNB2](http://www.ncbi.nlm.nih.gov/gene/?term=9312)(80.882)[KCNA5](http://www.ncbi.nlm.nih.gov/gene/?term=3741)(80.882)[RNASE1](http://www.ncbi.nlm.nih.gov/gene/?term=6035)(80.882) [KCND2](http://www.ncbi.nlm.nih.gov/gene/?term=3751)(80.882)[ALDH2](http://www.ncbi.nlm.nih.gov/gene/?term=217)(80.882)[KCNA4](http://www.ncbi.nlm.nih.gov/gene/?term=3739)(80.882)[KCNA7](http://www.ncbi.nlm.nih.gov/gene/?term=3743)(80.882)[KCNA6](http://www.ncbi.nlm.nih.gov/gene/?term=3742)(80.882) [KCND3](http://www.ncbi.nlm.nih.gov/gene/?term=3752)(80.882)[GUCY1B3](http://www.ncbi.nlm.nih.gov/gene/?term=2983)(55.444)[GATM](http://www.ncbi.nlm.nih.gov/gene/?term=2628)(55.444)[KCNK4](http://www.ncbi.nlm.nih.gov/gene/?term=50801)(55.444)[IYD](http://www.ncbi.nlm.nih.gov/gene/?term=389434)(55.444) [KCNQ1](http://www.ncbi.nlm.nih.gov/gene/?term=3784)(55.444)[CYP17A1](http://www.ncbi.nlm.nih.gov/gene/?term=1586)(48.000)[ESR1](http://www.ncbi.nlm.nih.gov/gene/?term=2099)(48.000)[PGR](http://www.ncbi.nlm.nih.gov/gene/?term=5241)(48.000)[OPRK1](http://www.ncbi.nlm.nih.gov/gene/?term=4986)(48.000) [NR3C2](http://www.ncbi.nlm.nih.gov/gene/?term=4306)(48.000)[FBP1](http://www.ncbi.nlm.nih.gov/gene/?term=2203)(22.373)[DGKI](http://www.ncbi.nlm.nih.gov/gene/?term=9162)(22.373)[ADH7](http://www.ncbi.nlm.nih.gov/gene/?term=131)(22.373)[NFIB](http://www.ncbi.nlm.nih.gov/gene/?term=4781)(22.373) [CYGB](http://www.ncbi.nlm.nih.gov/gene/?term=114757)(22.373)[NRXN3](http://www.ncbi.nlm.nih.gov/gene/?term=9369)(22.373)[LRRC4B](http://www.ncbi.nlm.nih.gov/gene/?term=94030)(22.373)[AQP8](http://www.ncbi.nlm.nih.gov/gene/?term=343)(22.373)[ARX](http://www.ncbi.nlm.nih.gov/gene/?term=170302)(22.373) [IGF1](http://www.ncbi.nlm.nih.gov/gene/?term=3479)(22.373)[SHANK3](http://www.ncbi.nlm.nih.gov/gene/?term=85358)(22.373)[ACY3](http://www.ncbi.nlm.nih.gov/gene/?term=91703)(22.373)[ASPA](http://www.ncbi.nlm.nih.gov/gene/?term=443)(22.373)[NRXN1](http://www.ncbi.nlm.nih.gov/gene/?term=9378)(22.373) [EDN1](http://www.ncbi.nlm.nih.gov/gene/?term=1906)(22.373)[OXT](http://www.ncbi.nlm.nih.gov/gene/?term=5020)(22.373)[MAGI2](http://www.ncbi.nlm.nih.gov/gene/?term=9863)(22.373)[APOE](http://www.ncbi.nlm.nih.gov/gene/?term=348)(22.373)[SOX15](http://www.ncbi.nlm.nih.gov/gene/?term=6665)(22.373) [CNTNAP4](http://www.ncbi.nlm.nih.gov/gene/?term=85445)(22.373)[RNASE4](http://www.ncbi.nlm.nih.gov/gene/?term=6038)(22.373)[RAB3A](http://www.ncbi.nlm.nih.gov/gene/?term=5864)(22.373)[MC4R](http://www.ncbi.nlm.nih.gov/gene/?term=4160)(22.373)[IL1B](http://www.ncbi.nlm.nih.gov/gene/?term=3553)(22.373) [GRIN2A](http://www.ncbi.nlm.nih.gov/gene/?term=2903)(22.373)[SLC17A7](http://www.ncbi.nlm.nih.gov/gene/?term=57030)(22.373)[CYP11A1](http://www.ncbi.nlm.nih.gov/gene/?term=1583)(22.373)[SCN5A](http://www.ncbi.nlm.nih.gov/gene/?term=6331)(22.373)[SCN10A](http://www.ncbi.nlm.nih.gov/gene/?term=6336)(22.373) [SPARC](http://www.ncbi.nlm.nih.gov/gene/?term=6678)(22.373)[SORCS3](http://www.ncbi.nlm.nih.gov/gene/?term=22986)(22.373)[RNASE8](http://www.ncbi.nlm.nih.gov/gene/?term=122665)(22.373)[KCNIP2](http://www.ncbi.nlm.nih.gov/gene/?term=30819)(22.373)[GPX7](http://www.ncbi.nlm.nih.gov/gene/?term=2882)(22.373) [CRLF1](http://www.ncbi.nlm.nih.gov/gene/?term=9244)(22.373)[FADD](http://www.ncbi.nlm.nih.gov/gene/?term=8772)(22.373)[SNTG2](http://www.ncbi.nlm.nih.gov/gene/?term=54221)(22.373)[SCGB1A1](http://www.ncbi.nlm.nih.gov/gene/?term=7356)(22.373)[OXTR](http://www.ncbi.nlm.nih.gov/gene/?term=5021)(22.373) [ALDH1B1](http://www.ncbi.nlm.nih.gov/gene/?term=219)(22.373)[LEP](http://www.ncbi.nlm.nih.gov/gene/?term=3952)(22.373)[NLGN1](http://www.ncbi.nlm.nih.gov/gene/?term=22871)(22.373)[CACNA1D](http://www.ncbi.nlm.nih.gov/gene/?term=776)(22.373)[RAPGEF2](http://www.ncbi.nlm.nih.gov/gene/?term=9693)(22.373) [DARS](http://www.ncbi.nlm.nih.gov/gene/?term=1615)(22.373)[ANK3](http://www.ncbi.nlm.nih.gov/gene/?term=288)(22.373)[KCNE5](http://www.ncbi.nlm.nih.gov/gene/?term=23630)(22.373)[PPARD](http://www.ncbi.nlm.nih.gov/gene/?term=5467)(22.373)[NKX2-1](http://www.ncbi.nlm.nih.gov/gene/?term=7080)(22.373) [MIP](http://www.ncbi.nlm.nih.gov/gene/?term=4284)(22.373)[RYR3](http://www.ncbi.nlm.nih.gov/gene/?term=6263)(22.373)[ZPR1](http://www.ncbi.nlm.nih.gov/gene/?term=8882)(22.373)[ALDH3B1](http://www.ncbi.nlm.nih.gov/gene/?term=221)(22.373)[NPPA](http://www.ncbi.nlm.nih.gov/gene/?term=4878)(22.373) [STX1A](http://www.ncbi.nlm.nih.gov/gene/?term=6804)(22.373)[PAXBP1](http://www.ncbi.nlm.nih.gov/gene/?term=94104)(22.373)[PAX7](http://www.ncbi.nlm.nih.gov/gene/?term=5081)(22.373)[ALDH3B2](http://www.ncbi.nlm.nih.gov/gene/?term=222)(22.373)[RNASE2](http://www.ncbi.nlm.nih.gov/gene/?term=6036)(22.373) [NRXN2](http://www.ncbi.nlm.nih.gov/gene/?term=9379)(22.373)[ADORA1](http://www.ncbi.nlm.nih.gov/gene/?term=134)(22.373)[ADH4](http://www.ncbi.nlm.nih.gov/gene/?term=127)(22.373)[GJA5](http://www.ncbi.nlm.nih.gov/gene/?term=2702)(22.373)[FAS](http://www.ncbi.nlm.nih.gov/gene/?term=355)(22.373) [DAB2IP](http://www.ncbi.nlm.nih.gov/gene/?term=153090)(22.373)[CYP2E1](http://www.ncbi.nlm.nih.gov/gene/?term=1571)(22.373)[ACY1](http://www.ncbi.nlm.nih.gov/gene/?term=95)(22.373) |
| [Dehydrocorydaline](http://www.megabionet.org/tcmid/ingredient/4888/) | [PDE10A](http://www.ncbi.nlm.nih.gov/gene/?term=10846)(80.882)[PDE4B](http://www.ncbi.nlm.nih.gov/gene/?term=5142)(80.882)[PDE4D](http://www.ncbi.nlm.nih.gov/gene/?term=5144)(55.444)[PDE5A](http://www.ncbi.nlm.nih.gov/gene/?term=8654)(22.373)[PDE9A](http://www.ncbi.nlm.nih.gov/gene/?term=5152)(22.373) [PDE1B](http://www.ncbi.nlm.nih.gov/gene/?term=5153)(22.373)[PLN](http://www.ncbi.nlm.nih.gov/gene/?term=5350)(22.373)[HRC](http://www.ncbi.nlm.nih.gov/gene/?term=3270)(22.373)[CAMK2D](http://www.ncbi.nlm.nih.gov/gene/?term=817)(22.373)[PDE2A](http://www.ncbi.nlm.nih.gov/gene/?term=5138)(22.373) [CAMK2G](http://www.ncbi.nlm.nih.gov/gene/?term=818)(22.373)[PDE11A](http://www.ncbi.nlm.nih.gov/gene/?term=50940)(22.373) |
| [P-Cresol](http://www.megabionet.org/tcmid/ingredient/4233/) | [GABRA2](http://www.ncbi.nlm.nih.gov/gene/?term=2555)(80.882)[GABRD](http://www.ncbi.nlm.nih.gov/gene/?term=2563)(80.882)[PDE3A](http://www.ncbi.nlm.nih.gov/gene/?term=5139)(80.882)[GABRB1](http://www.ncbi.nlm.nih.gov/gene/?term=2560)(80.882)[GABRG3](http://www.ncbi.nlm.nih.gov/gene/?term=2567)(80.882) [TNF](http://www.ncbi.nlm.nih.gov/gene/?term=7124)(80.882)[GABRE](http://www.ncbi.nlm.nih.gov/gene/?term=2564)(80.882)[SCN2A](http://www.ncbi.nlm.nih.gov/gene/?term=6326)(80.882)[GABRA3](http://www.ncbi.nlm.nih.gov/gene/?term=2556)(80.882)[GABRG1](http://www.ncbi.nlm.nih.gov/gene/?term=2565)(80.882) [GABRP](http://www.ncbi.nlm.nih.gov/gene/?term=2568)(80.882)[GABRA4](http://www.ncbi.nlm.nih.gov/gene/?term=2557)(80.882)[GABRB2](http://www.ncbi.nlm.nih.gov/gene/?term=2561)(80.882)[ACHE](http://www.ncbi.nlm.nih.gov/gene/?term=43)(80.882)[SCN4A](http://www.ncbi.nlm.nih.gov/gene/?term=6329)(80.882) [GABRQ](http://www.ncbi.nlm.nih.gov/gene/?term=55879)(80.882)[GABRB3](http://www.ncbi.nlm.nih.gov/gene/?term=2562)(80.882)[GABRA5](http://www.ncbi.nlm.nih.gov/gene/?term=2558)(80.882)[GABRA6](http://www.ncbi.nlm.nih.gov/gene/?term=2559)(80.882)[GABRA1](http://www.ncbi.nlm.nih.gov/gene/?term=2554)(80.882) [PDE4B](http://www.ncbi.nlm.nih.gov/gene/?term=5142)(80.882)[BCHE](http://www.ncbi.nlm.nih.gov/gene/?term=590)(80.882)[GABRG2](http://www.ncbi.nlm.nih.gov/gene/?term=2566)(80.882)[PDE3B](http://www.ncbi.nlm.nih.gov/gene/?term=5140)(55.444)[PDE4D](http://www.ncbi.nlm.nih.gov/gene/?term=5144)(55.444) [COLQ](http://www.ncbi.nlm.nih.gov/gene/?term=8292)(55.444)[LTA](http://www.ncbi.nlm.nih.gov/gene/?term=4049)(55.444)[RXRA](http://www.ncbi.nlm.nih.gov/gene/?term=6256)(55.444)[SCN1A](http://www.ncbi.nlm.nih.gov/gene/?term=6323)(23.000)[SCN3A](http://www.ncbi.nlm.nih.gov/gene/?term=6328)(23.000) [HMGA2](http://www.ncbi.nlm.nih.gov/gene/?term=8091)(22.373)[PDE5A](http://www.ncbi.nlm.nih.gov/gene/?term=8654)(22.373)[APOA2](http://www.ncbi.nlm.nih.gov/gene/?term=336)(22.373)[SLC5A7](http://www.ncbi.nlm.nih.gov/gene/?term=60482)(22.373)[SEMA4D](http://www.ncbi.nlm.nih.gov/gene/?term=10507)(22.373) [NRG1](http://www.ncbi.nlm.nih.gov/gene/?term=3084)(22.373)[CD74](http://www.ncbi.nlm.nih.gov/gene/?term=972)(22.373)[HPX](http://www.ncbi.nlm.nih.gov/gene/?term=3263)(22.373)[SIRT2](http://www.ncbi.nlm.nih.gov/gene/?term=22933)(22.373)[MIF](http://www.ncbi.nlm.nih.gov/gene/?term=4282)(22.373) [DMGDH](http://www.ncbi.nlm.nih.gov/gene/?term=29958)(22.373)[NKX3-1](http://www.ncbi.nlm.nih.gov/gene/?term=4824)(22.373)[SIX3](http://www.ncbi.nlm.nih.gov/gene/?term=6496)(22.373)[HCN4](http://www.ncbi.nlm.nih.gov/gene/?term=10021)(22.373)[FASLG](http://www.ncbi.nlm.nih.gov/gene/?term=356)(22.373) [LGALS3](http://www.ncbi.nlm.nih.gov/gene/?term=3958)(22.373)[KLF4](http://www.ncbi.nlm.nih.gov/gene/?term=9314)(22.373)[LILRB1](http://www.ncbi.nlm.nih.gov/gene/?term=10859)(22.373)[NCKAP1L](http://www.ncbi.nlm.nih.gov/gene/?term=3071)(22.373)[CRP](http://www.ncbi.nlm.nih.gov/gene/?term=1401)(22.373) [TLR3](http://www.ncbi.nlm.nih.gov/gene/?term=7098)(22.373)[EPO](http://www.ncbi.nlm.nih.gov/gene/?term=2056)(22.373)[MAP2K5](http://www.ncbi.nlm.nih.gov/gene/?term=5607)(22.373)[EIF2AK1](http://www.ncbi.nlm.nih.gov/gene/?term=27102)(22.373)[IL1B](http://www.ncbi.nlm.nih.gov/gene/?term=3553)(22.373) [SHH](http://www.ncbi.nlm.nih.gov/gene/?term=6469)(22.373)[RIPK1](http://www.ncbi.nlm.nih.gov/gene/?term=8737)(22.373)[FGF10](http://www.ncbi.nlm.nih.gov/gene/?term=2255)(22.373)[PRSS12](http://www.ncbi.nlm.nih.gov/gene/?term=8492)(22.373)[SLC44A4](http://www.ncbi.nlm.nih.gov/gene/?term=80736)(22.373) [ADORA2B](http://www.ncbi.nlm.nih.gov/gene/?term=136)(22.373)[ALDH7A1](http://www.ncbi.nlm.nih.gov/gene/?term=501)(22.373)[FADD](http://www.ncbi.nlm.nih.gov/gene/?term=8772)(22.373)[FGFR2](http://www.ncbi.nlm.nih.gov/gene/?term=2263)(22.373)[ANGPT1](http://www.ncbi.nlm.nih.gov/gene/?term=284)(22.373) [AURKA](http://www.ncbi.nlm.nih.gov/gene/?term=6790)(22.373)[WNT2](http://www.ncbi.nlm.nih.gov/gene/?term=7472)(22.373)[YBX3](http://www.ncbi.nlm.nih.gov/gene/?term=8531)(22.373)[IL1RN](http://www.ncbi.nlm.nih.gov/gene/?term=3557)(22.373)[PLN](http://www.ncbi.nlm.nih.gov/gene/?term=5350)(22.373) [DNM3](http://www.ncbi.nlm.nih.gov/gene/?term=26052)(22.373)[FCER2](http://www.ncbi.nlm.nih.gov/gene/?term=2208)(22.373)[WNT2B](http://www.ncbi.nlm.nih.gov/gene/?term=7482)(22.373)[IFNG](http://www.ncbi.nlm.nih.gov/gene/?term=3458)(22.373)[HRC](http://www.ncbi.nlm.nih.gov/gene/?term=3270)(22.373) [SLC6A4](http://www.ncbi.nlm.nih.gov/gene/?term=6532)(22.373)[TGFB2](http://www.ncbi.nlm.nih.gov/gene/?term=7042)(22.373)[RAPGEF2](http://www.ncbi.nlm.nih.gov/gene/?term=9693)(22.373)[TIRAP](http://www.ncbi.nlm.nih.gov/gene/?term=114609)(22.373)[ROCK2](http://www.ncbi.nlm.nih.gov/gene/?term=9475)(22.373) [ADIRF](http://www.ncbi.nlm.nih.gov/gene/?term=10974)(22.373)[BDKRB2](http://www.ncbi.nlm.nih.gov/gene/?term=624)(22.373)[ASCL1](http://www.ncbi.nlm.nih.gov/gene/?term=429)(22.373)[ENPP6](http://www.ncbi.nlm.nih.gov/gene/?term=133121)(22.373)[CAMK2D](http://www.ncbi.nlm.nih.gov/gene/?term=817)(22.373) [IL10](http://www.ncbi.nlm.nih.gov/gene/?term=3586)(22.373)[CDH8](http://www.ncbi.nlm.nih.gov/gene/?term=1006)(22.373)[NCOA1](http://www.ncbi.nlm.nih.gov/gene/?term=8648)(22.373)[IL6](http://www.ncbi.nlm.nih.gov/gene/?term=3569)(22.373)[ROCK1](http://www.ncbi.nlm.nih.gov/gene/?term=6093)(22.373) [FNTA](http://www.ncbi.nlm.nih.gov/gene/?term=2339)(22.373)[HPN](http://www.ncbi.nlm.nih.gov/gene/?term=3249)(22.373)[KMT2A](http://www.ncbi.nlm.nih.gov/gene/?term=4297)(22.373)[NOD2](http://www.ncbi.nlm.nih.gov/gene/?term=64127)(22.373)[BMP4](http://www.ncbi.nlm.nih.gov/gene/?term=652)(22.373) [AHCY](http://www.ncbi.nlm.nih.gov/gene/?term=191)(22.373)[APOA1](http://www.ncbi.nlm.nih.gov/gene/?term=335)(22.373)[ARHGEF2](http://www.ncbi.nlm.nih.gov/gene/?term=9181)(22.373)[IL4](http://www.ncbi.nlm.nih.gov/gene/?term=3565)(22.373)[AGRN](http://www.ncbi.nlm.nih.gov/gene/?term=375790)(22.373) [IKBKB](http://www.ncbi.nlm.nih.gov/gene/?term=3551)(22.373)[IDO1](http://www.ncbi.nlm.nih.gov/gene/?term=3620)(22.373)[CTNNB1](http://www.ncbi.nlm.nih.gov/gene/?term=1499)(22.373)[CHKA](http://www.ncbi.nlm.nih.gov/gene/?term=1119)(22.373)[PDE2A](http://www.ncbi.nlm.nih.gov/gene/?term=5138)(22.373) [CHDH](http://www.ncbi.nlm.nih.gov/gene/?term=55349)(22.373)[SOX9](http://www.ncbi.nlm.nih.gov/gene/?term=6662)(22.373)[CAMK2G](http://www.ncbi.nlm.nih.gov/gene/?term=818)(22.373)[FAS](http://www.ncbi.nlm.nih.gov/gene/?term=355)(22.373)[GRIN1](http://www.ncbi.nlm.nih.gov/gene/?term=2902)(22.373) [HCN2](http://www.ncbi.nlm.nih.gov/gene/?term=610)(22.373) |
| [Rheinoside A](http://www.megabionet.org/tcmid/ingredient/18747/) | [TOP2A](http://www.ncbi.nlm.nih.gov/gene/?term=7153)(48.000)[CHD1](http://www.ncbi.nlm.nih.gov/gene/?term=1105)(48.000)[TOP2B](http://www.ncbi.nlm.nih.gov/gene/?term=7155)(48.000) |
| [Oleanolic Acid-28-O-Beta-D-Glucopyranoside](http://www.megabionet.org/tcmid/ingredient/31751/) | [GLRA3](http://www.ncbi.nlm.nih.gov/gene/?term=8001)(48.000)[NR3C1](http://www.ncbi.nlm.nih.gov/gene/?term=2908)(48.000)[ATP1A1](http://www.ncbi.nlm.nih.gov/gene/?term=476)(48.000)[GABRB3](http://www.ncbi.nlm.nih.gov/gene/?term=2562)(48.000) |
| [Sennoside E](http://www.megabionet.org/tcmid/ingredient/30570/) | This compound doesn't have any potential target with score larger than 20. |
| [O-Methylacetophenone](http://www.megabionet.org/tcmid/ingredient/14105/) | [PTGIS](http://www.ncbi.nlm.nih.gov/gene/?term=5740)(80.882)[VKORC1](http://www.ncbi.nlm.nih.gov/gene/?term=79001)(80.882)[CYP11B1](http://www.ncbi.nlm.nih.gov/gene/?term=1584)(80.882)[PTGS1](http://www.ncbi.nlm.nih.gov/gene/?term=5742)(80.882)[PTGS2](http://www.ncbi.nlm.nih.gov/gene/?term=5743)(80.882) [CYP11B2](http://www.ncbi.nlm.nih.gov/gene/?term=1585)(80.882)[CACNA1G](http://www.ncbi.nlm.nih.gov/gene/?term=8913)(80.882)[CACNA1H](http://www.ncbi.nlm.nih.gov/gene/?term=8912)(55.444)[LCT](http://www.ncbi.nlm.nih.gov/gene/?term=3938)(55.444)[VKORC1L1](http://www.ncbi.nlm.nih.gov/gene/?term=154807)(55.444) [SERPINB7](http://www.ncbi.nlm.nih.gov/gene/?term=8710)(55.444)[NDRG2](http://www.ncbi.nlm.nih.gov/gene/?term=57447)(22.373)[NAMPT](http://www.ncbi.nlm.nih.gov/gene/?term=10135)(22.373)[RGCC](http://www.ncbi.nlm.nih.gov/gene/?term=28984)(22.373)[FNDC5](http://www.ncbi.nlm.nih.gov/gene/?term=252995)(22.373) [AVPR1A](http://www.ncbi.nlm.nih.gov/gene/?term=552)(22.373)[METRNL](http://www.ncbi.nlm.nih.gov/gene/?term=284207)(22.373)[MAPK9](http://www.ncbi.nlm.nih.gov/gene/?term=5601)(22.373)[CYP4F2](http://www.ncbi.nlm.nih.gov/gene/?term=8529)(22.373)[CYP4F11](http://www.ncbi.nlm.nih.gov/gene/?term=57834)(22.373) [HEG1](http://www.ncbi.nlm.nih.gov/gene/?term=57493)(22.373)[AVP](http://www.ncbi.nlm.nih.gov/gene/?term=551)(22.373)[SLC6A4](http://www.ncbi.nlm.nih.gov/gene/?term=6532)(22.373)[BDKRB2](http://www.ncbi.nlm.nih.gov/gene/?term=624)(22.373)[NEUROD2](http://www.ncbi.nlm.nih.gov/gene/?term=4761)(22.373) [INS](http://www.ncbi.nlm.nih.gov/gene/?term=3630)(22.373)[ANAPC2](http://www.ncbi.nlm.nih.gov/gene/?term=29882)(22.373)[WNT11](http://www.ncbi.nlm.nih.gov/gene/?term=7481)(22.373)[ALOX15B](http://www.ncbi.nlm.nih.gov/gene/?term=247)(22.373)[CDC20](http://www.ncbi.nlm.nih.gov/gene/?term=991)(22.373) [NAPRT](http://www.ncbi.nlm.nih.gov/gene/?term=93100)(22.373)[MECOM](http://www.ncbi.nlm.nih.gov/gene/?term=2122)(22.373)[SNCA](http://www.ncbi.nlm.nih.gov/gene/?term=6622)(22.373)[CPLX2](http://www.ncbi.nlm.nih.gov/gene/?term=10814)(22.373)[ALOX15](http://www.ncbi.nlm.nih.gov/gene/?term=246)(22.373) [ANXA1](http://www.ncbi.nlm.nih.gov/gene/?term=301)(22.373)[UBIAD1](http://www.ncbi.nlm.nih.gov/gene/?term=29914)(22.373)[CBR1](http://www.ncbi.nlm.nih.gov/gene/?term=873)(22.373)[PRDM16](http://www.ncbi.nlm.nih.gov/gene/?term=63976)(22.373)[CCM2L](http://www.ncbi.nlm.nih.gov/gene/?term=140706)(22.373) |
| [Elemicin](http://www.megabionet.org/tcmid/ingredient/6744/) | [PDE4A](http://www.ncbi.nlm.nih.gov/gene/?term=5141)(80.882)[PDE3A](http://www.ncbi.nlm.nih.gov/gene/?term=5139)(80.882)[CACNA1C](http://www.ncbi.nlm.nih.gov/gene/?term=775)(80.882)[TRDMT1](http://www.ncbi.nlm.nih.gov/gene/?term=1787)(80.882)[DHFRL1](http://www.ncbi.nlm.nih.gov/gene/?term=200895)(80.882) [DHFR](http://www.ncbi.nlm.nih.gov/gene/?term=1719)(80.882)[PDE10A](http://www.ncbi.nlm.nih.gov/gene/?term=10846)(80.882)[PDE2A](http://www.ncbi.nlm.nih.gov/gene/?term=5138)(80.882)[TYMS](http://www.ncbi.nlm.nih.gov/gene/?term=7298)(80.882)[SHMT1](http://www.ncbi.nlm.nih.gov/gene/?term=6470)(55.444) [PDE3B](http://www.ncbi.nlm.nih.gov/gene/?term=5140)(55.444)[DMTN](http://www.ncbi.nlm.nih.gov/gene/?term=2039)(55.444)[KCNMA1](http://www.ncbi.nlm.nih.gov/gene/?term=3778)(55.444)[ABCC2](http://www.ncbi.nlm.nih.gov/gene/?term=1244)(22.373)[FOLR2](http://www.ncbi.nlm.nih.gov/gene/?term=2350)(22.373) [PDE5A](http://www.ncbi.nlm.nih.gov/gene/?term=8654)(22.373)[PDE9A](http://www.ncbi.nlm.nih.gov/gene/?term=5152)(22.373)[TBPL1](http://www.ncbi.nlm.nih.gov/gene/?term=9519)(22.373)[DNMT3A](http://www.ncbi.nlm.nih.gov/gene/?term=1788)(22.373)[STUB1](http://www.ncbi.nlm.nih.gov/gene/?term=10273)(22.373) [INSR](http://www.ncbi.nlm.nih.gov/gene/?term=3643)(22.373)[P2RX2](http://www.ncbi.nlm.nih.gov/gene/?term=22953)(22.373)[HTR2A](http://www.ncbi.nlm.nih.gov/gene/?term=3356)(22.373)[SIRT2](http://www.ncbi.nlm.nih.gov/gene/?term=22933)(22.373)[HCN4](http://www.ncbi.nlm.nih.gov/gene/?term=10021)(22.373) [DPYD](http://www.ncbi.nlm.nih.gov/gene/?term=1806)(22.373)[HOXA5](http://www.ncbi.nlm.nih.gov/gene/?term=3202)(22.373)[FBLN1](http://www.ncbi.nlm.nih.gov/gene/?term=2192)(22.373)[SLC22A6](http://www.ncbi.nlm.nih.gov/gene/?term=9356)(22.373)[ASNS](http://www.ncbi.nlm.nih.gov/gene/?term=440)(22.373) [ADK](http://www.ncbi.nlm.nih.gov/gene/?term=132)(22.373)[IL1B](http://www.ncbi.nlm.nih.gov/gene/?term=3553)(22.373)[NT5M](http://www.ncbi.nlm.nih.gov/gene/?term=56953)(22.373)[P2RX3](http://www.ncbi.nlm.nih.gov/gene/?term=5024)(22.373)[AURKA](http://www.ncbi.nlm.nih.gov/gene/?term=6790)(22.373) [GAS6](http://www.ncbi.nlm.nih.gov/gene/?term=2621)(22.373)[CMPK2](http://www.ncbi.nlm.nih.gov/gene/?term=129607)(22.373)[PDE1B](http://www.ncbi.nlm.nih.gov/gene/?term=5153)(22.373)[DPYS](http://www.ncbi.nlm.nih.gov/gene/?term=1807)(22.373)[CHRNA3](http://www.ncbi.nlm.nih.gov/gene/?term=1136)(22.373) [HSP90AB1](http://www.ncbi.nlm.nih.gov/gene/?term=3326)(22.373)[CHRNB2](http://www.ncbi.nlm.nih.gov/gene/?term=1141)(22.373)[SLC6A4](http://www.ncbi.nlm.nih.gov/gene/?term=6532)(22.373)[DUT](http://www.ncbi.nlm.nih.gov/gene/?term=1854)(22.373)[RAPGEF2](http://www.ncbi.nlm.nih.gov/gene/?term=9693)(22.373) [ATIC](http://www.ncbi.nlm.nih.gov/gene/?term=471)(22.373)[AK9](http://www.ncbi.nlm.nih.gov/gene/?term=221264)(22.373)[CDKN1A](http://www.ncbi.nlm.nih.gov/gene/?term=1026)(22.373)[HIF1A](http://www.ncbi.nlm.nih.gov/gene/?term=3091)(22.373)[FOLR1](http://www.ncbi.nlm.nih.gov/gene/?term=2348)(22.373) [RAB8B](http://www.ncbi.nlm.nih.gov/gene/?term=51762)(22.373)[HYAL2](http://www.ncbi.nlm.nih.gov/gene/?term=8692)(22.373)[DNMT3B](http://www.ncbi.nlm.nih.gov/gene/?term=1789)(22.373)[HSP90AA1](http://www.ncbi.nlm.nih.gov/gene/?term=3320)(22.373)[DNMT1](http://www.ncbi.nlm.nih.gov/gene/?term=1786)(22.373) [PARP10](http://www.ncbi.nlm.nih.gov/gene/?term=84875)(22.373)[CHRNB4](http://www.ncbi.nlm.nih.gov/gene/?term=1143)(22.373)[MGMT](http://www.ncbi.nlm.nih.gov/gene/?term=4255)(22.373)[DTYMK](http://www.ncbi.nlm.nih.gov/gene/?term=1841)(22.373)[PDE11A](http://www.ncbi.nlm.nih.gov/gene/?term=50940)(22.373) [HCN2](http://www.ncbi.nlm.nih.gov/gene/?term=610)(22.373) |
| [M-Methoxybenzaldehyde](http://www.megabionet.org/tcmid/ingredient/13841/) | This compound doesn't have any potential target with score larger than 20. |
| [Rhaponticin](http://www.megabionet.org/tcmid/ingredient/18729/) | This compound doesn't have any potential target with score larger than 20. |
| [Sennoside B](http://www.megabionet.org/tcmid/ingredient/19735/) | This compound doesn't have any potential target with score larger than 20. |
| [Reticulin](http://www.megabionet.org/tcmid/ingredient/18639/) | [PADI4](http://www.ncbi.nlm.nih.gov/gene/?term=23569)(122.778)[DHFR](http://www.ncbi.nlm.nih.gov/gene/?term=1719)(48.000)[LRP2](http://www.ncbi.nlm.nih.gov/gene/?term=4036)(48.000)[PADI2](http://www.ncbi.nlm.nih.gov/gene/?term=11240)(22.373) |
| [3-Methoxygallic Acid](http://www.megabionet.org/tcmid/ingredient/31542/) | This compound doesn't have any potential target with score larger than 20. |
| [Tetrahydrocorysamine](http://www.megabionet.org/tcmid/ingredient/21046/) | [CHRM3](http://www.ncbi.nlm.nih.gov/gene/?term=1131)(48.000)[DRD2](http://www.ncbi.nlm.nih.gov/gene/?term=1813)(48.000)[ACHE](http://www.ncbi.nlm.nih.gov/gene/?term=43)(48.000)[SLC18A2](http://www.ncbi.nlm.nih.gov/gene/?term=6571)(48.000)[CHRNA2](http://www.ncbi.nlm.nih.gov/gene/?term=1135)(48.000) [HTR3A](http://www.ncbi.nlm.nih.gov/gene/?term=3359)(48.000)[BCHE](http://www.ncbi.nlm.nih.gov/gene/?term=590)(48.000)[CHRM2](http://www.ncbi.nlm.nih.gov/gene/?term=1129)(48.000)[HTR2A](http://www.ncbi.nlm.nih.gov/gene/?term=3356)(23.000)[CHRNA4](http://www.ncbi.nlm.nih.gov/gene/?term=1137)(23.000) [CHRNB3](http://www.ncbi.nlm.nih.gov/gene/?term=1142)(23.000)[SULT1E1](http://www.ncbi.nlm.nih.gov/gene/?term=6783)(23.000)[CHRM1](http://www.ncbi.nlm.nih.gov/gene/?term=1128)(23.000)[CHRNA7](http://www.ncbi.nlm.nih.gov/gene/?term=1139)(23.000)[HTR2C](http://www.ncbi.nlm.nih.gov/gene/?term=3358)(23.000) [CHRNA10](http://www.ncbi.nlm.nih.gov/gene/?term=57053)(23.000)[CHRNA3](http://www.ncbi.nlm.nih.gov/gene/?term=1136)(23.000)[CHRNB2](http://www.ncbi.nlm.nih.gov/gene/?term=1141)(23.000)[HRH1](http://www.ncbi.nlm.nih.gov/gene/?term=3269)(23.000)[CHRNA6](http://www.ncbi.nlm.nih.gov/gene/?term=8973)(23.000) [CHAT](http://www.ncbi.nlm.nih.gov/gene/?term=1103)(23.000)[CHRNA9](http://www.ncbi.nlm.nih.gov/gene/?term=55584)(23.000)[CHRFAM7A](http://www.ncbi.nlm.nih.gov/gene/?term=89832)(23.000)[CYP19A1](http://www.ncbi.nlm.nih.gov/gene/?term=1588)(23.000)[CHRNA5](http://www.ncbi.nlm.nih.gov/gene/?term=1138)(23.000) [CHRNB4](http://www.ncbi.nlm.nih.gov/gene/?term=1143)(23.000) |
| [Vanillin Acetate](http://www.megabionet.org/tcmid/ingredient/22321/) | This compound doesn't have any potential target with score larger than 20. |
| [Scoulerine](http://www.megabionet.org/tcmid/ingredient/19551/) | [HTR2A](http://www.ncbi.nlm.nih.gov/gene/?term=3356)(48.000)[CHRM3](http://www.ncbi.nlm.nih.gov/gene/?term=1131)(48.000)[HTR2B](http://www.ncbi.nlm.nih.gov/gene/?term=3357)(48.000)[ADRA2A](http://www.ncbi.nlm.nih.gov/gene/?term=150)(48.000)[HTR1B](http://www.ncbi.nlm.nih.gov/gene/?term=3351)(48.000) [HTR2C](http://www.ncbi.nlm.nih.gov/gene/?term=3358)(48.000)[HTR1D](http://www.ncbi.nlm.nih.gov/gene/?term=3352)(48.000)[DRD2](http://www.ncbi.nlm.nih.gov/gene/?term=1813)(48.000)[ACHE](http://www.ncbi.nlm.nih.gov/gene/?term=43)(48.000)[SLC18A2](http://www.ncbi.nlm.nih.gov/gene/?term=6571)(48.000) [DRD4](http://www.ncbi.nlm.nih.gov/gene/?term=1815)(48.000)[CHRNA2](http://www.ncbi.nlm.nih.gov/gene/?term=1135)(48.000)[HTR1A](http://www.ncbi.nlm.nih.gov/gene/?term=3350)(48.000)[ADRA2C](http://www.ncbi.nlm.nih.gov/gene/?term=152)(48.000)[CALY](http://www.ncbi.nlm.nih.gov/gene/?term=50632)(48.000) [DRD3](http://www.ncbi.nlm.nih.gov/gene/?term=1814)(48.000)[DRD5](http://www.ncbi.nlm.nih.gov/gene/?term=1816)(48.000)[HTR3A](http://www.ncbi.nlm.nih.gov/gene/?term=3359)(48.000)[DRD1](http://www.ncbi.nlm.nih.gov/gene/?term=1812)(48.000)[BCHE](http://www.ncbi.nlm.nih.gov/gene/?term=590)(48.000) [CHRM2](http://www.ncbi.nlm.nih.gov/gene/?term=1129)(48.000)[ADRA2B](http://www.ncbi.nlm.nih.gov/gene/?term=151)(48.000)[ESR2](http://www.ncbi.nlm.nih.gov/gene/?term=2100)(23.000)[OPRD1](http://www.ncbi.nlm.nih.gov/gene/?term=4985)(23.000)[ESR1](http://www.ncbi.nlm.nih.gov/gene/?term=2099)(23.000) [OPRK1](http://www.ncbi.nlm.nih.gov/gene/?term=4986)(23.000)[OPRM1](http://www.ncbi.nlm.nih.gov/gene/?term=4988)(23.000) |
| [Isocorydine](http://www.megabionet.org/tcmid/ingredient/11341/) | [CHRM3](http://www.ncbi.nlm.nih.gov/gene/?term=1131)(686.000)[OPRD1](http://www.ncbi.nlm.nih.gov/gene/?term=4985)(686.000)[HTR2C](http://www.ncbi.nlm.nih.gov/gene/?term=3358)(686.000)[OPRK1](http://www.ncbi.nlm.nih.gov/gene/?term=4986)(686.000)[SLC6A4](http://www.ncbi.nlm.nih.gov/gene/?term=6532)(686.000) [OPRM1](http://www.ncbi.nlm.nih.gov/gene/?term=4988)(686.000)[GRIN3A](http://www.ncbi.nlm.nih.gov/gene/?term=116443)(686.000)[SLC6A2](http://www.ncbi.nlm.nih.gov/gene/?term=6530)(686.000)[SLC6A3](http://www.ncbi.nlm.nih.gov/gene/?term=6531)(80.882)[HTR2A](http://www.ncbi.nlm.nih.gov/gene/?term=3356)(55.444) [SLC18A1](http://www.ncbi.nlm.nih.gov/gene/?term=6570)(55.444)[CEND1](http://www.ncbi.nlm.nih.gov/gene/?term=51286)(55.444)[SLC22A1](http://www.ncbi.nlm.nih.gov/gene/?term=6580)(55.444)[WLS](http://www.ncbi.nlm.nih.gov/gene/?term=79971)(55.444)[CYBA](http://www.ncbi.nlm.nih.gov/gene/?term=1535)(48.000) [CHRNA4](http://www.ncbi.nlm.nih.gov/gene/?term=1137)(48.000)[RAC2](http://www.ncbi.nlm.nih.gov/gene/?term=5880)(48.000)[RAC1](http://www.ncbi.nlm.nih.gov/gene/?term=5879)(48.000)[CHRM1](http://www.ncbi.nlm.nih.gov/gene/?term=1128)(48.000)[NCF4](http://www.ncbi.nlm.nih.gov/gene/?term=4689)(48.000) [CHRNA7](http://www.ncbi.nlm.nih.gov/gene/?term=1139)(48.000)[CYBB](http://www.ncbi.nlm.nih.gov/gene/?term=1536)(48.000)[NCF1](http://www.ncbi.nlm.nih.gov/gene/?term=653361)(48.000)[SIGMAR1](http://www.ncbi.nlm.nih.gov/gene/?term=10280)(48.000)[NCF2](http://www.ncbi.nlm.nih.gov/gene/?term=4688)(48.000) [CHRNA3](http://www.ncbi.nlm.nih.gov/gene/?term=1136)(48.000)[CHRNB2](http://www.ncbi.nlm.nih.gov/gene/?term=1141)(48.000)[CHRM5](http://www.ncbi.nlm.nih.gov/gene/?term=1133)(48.000)[CHRNA2](http://www.ncbi.nlm.nih.gov/gene/?term=1135)(48.000)[PGRMC1](http://www.ncbi.nlm.nih.gov/gene/?term=10857)(48.000) [HTR3A](http://www.ncbi.nlm.nih.gov/gene/?term=3359)(48.000)[CHRFAM7A](http://www.ncbi.nlm.nih.gov/gene/?term=89832)(48.000)[CHRM2](http://www.ncbi.nlm.nih.gov/gene/?term=1129)(48.000)[CHRNB4](http://www.ncbi.nlm.nih.gov/gene/?term=1143)(48.000)[CHRM4](http://www.ncbi.nlm.nih.gov/gene/?term=1132)(48.000) [TNFSF11](http://www.ncbi.nlm.nih.gov/gene/?term=8600)(22.373)[ACTN3](http://www.ncbi.nlm.nih.gov/gene/?term=89)(22.373)[GPM6B](http://www.ncbi.nlm.nih.gov/gene/?term=2824)(22.373)[TAC1](http://www.ncbi.nlm.nih.gov/gene/?term=6863)(22.373)[MC2R](http://www.ncbi.nlm.nih.gov/gene/?term=4158)(22.373) [PDE3A](http://www.ncbi.nlm.nih.gov/gene/?term=5139)(22.373)[AVPR1A](http://www.ncbi.nlm.nih.gov/gene/?term=552)(22.373)[HCN4](http://www.ncbi.nlm.nih.gov/gene/?term=10021)(22.373)[OXT](http://www.ncbi.nlm.nih.gov/gene/?term=5020)(22.373)[CCL5](http://www.ncbi.nlm.nih.gov/gene/?term=6352)(22.373) [SLC22A2](http://www.ncbi.nlm.nih.gov/gene/?term=6582)(22.373)[PPP1R9B](http://www.ncbi.nlm.nih.gov/gene/?term=84687)(22.373)[HTR2B](http://www.ncbi.nlm.nih.gov/gene/?term=3357)(22.373)[COMT](http://www.ncbi.nlm.nih.gov/gene/?term=1312)(22.373)[DOCK5](http://www.ncbi.nlm.nih.gov/gene/?term=80005)(22.373) [MC1R](http://www.ncbi.nlm.nih.gov/gene/?term=4157)(22.373)[MC4R](http://www.ncbi.nlm.nih.gov/gene/?term=4160)(22.373)[FGF10](http://www.ncbi.nlm.nih.gov/gene/?term=2255)(22.373)[MAOB](http://www.ncbi.nlm.nih.gov/gene/?term=4129)(22.373)[EGR1](http://www.ncbi.nlm.nih.gov/gene/?term=1958)(22.373) [APLN](http://www.ncbi.nlm.nih.gov/gene/?term=8862)(22.373)[VCP](http://www.ncbi.nlm.nih.gov/gene/?term=7415)(22.373)[OXTR](http://www.ncbi.nlm.nih.gov/gene/?term=5021)(22.373)[MAOA](http://www.ncbi.nlm.nih.gov/gene/?term=4128)(22.373)[ACVR2A](http://www.ncbi.nlm.nih.gov/gene/?term=92)(22.373) [RAPGEF2](http://www.ncbi.nlm.nih.gov/gene/?term=9693)(22.373)[AIF1](http://www.ncbi.nlm.nih.gov/gene/?term=199)(22.373)[HIF1A](http://www.ncbi.nlm.nih.gov/gene/?term=3091)(22.373)[BNIP3](http://www.ncbi.nlm.nih.gov/gene/?term=664)(22.373)[CRH](http://www.ncbi.nlm.nih.gov/gene/?term=1392)(22.373) [LRTOMT](http://www.ncbi.nlm.nih.gov/gene/?term=220074)(22.373)[NEFL](http://www.ncbi.nlm.nih.gov/gene/?term=4747)(22.373)[DBH](http://www.ncbi.nlm.nih.gov/gene/?term=1621)(22.373)[UCN2](http://www.ncbi.nlm.nih.gov/gene/?term=90226)(22.373)[TACR2](http://www.ncbi.nlm.nih.gov/gene/?term=6865)(22.373) [PDGFA](http://www.ncbi.nlm.nih.gov/gene/?term=5154)(22.373)[PTGS2](http://www.ncbi.nlm.nih.gov/gene/?term=5743)(22.373)[MC5R](http://www.ncbi.nlm.nih.gov/gene/?term=4161)(22.373)[AQP1](http://www.ncbi.nlm.nih.gov/gene/?term=358)(22.373)[MC3R](http://www.ncbi.nlm.nih.gov/gene/?term=4159)(22.373) [TAC4](http://www.ncbi.nlm.nih.gov/gene/?term=255061)(22.373)[GNA15](http://www.ncbi.nlm.nih.gov/gene/?term=2769)(22.373)[ZP3](http://www.ncbi.nlm.nih.gov/gene/?term=7784)(22.373)[SNCA](http://www.ncbi.nlm.nih.gov/gene/?term=6622)(22.373)[PDE2A](http://www.ncbi.nlm.nih.gov/gene/?term=5138)(22.373) [CBFA2T3](http://www.ncbi.nlm.nih.gov/gene/?term=863)(22.373)[P2RX1](http://www.ncbi.nlm.nih.gov/gene/?term=5023)(22.373)[DOCK4](http://www.ncbi.nlm.nih.gov/gene/?term=9732)(22.373)[NOS1](http://www.ncbi.nlm.nih.gov/gene/?term=4842)(22.373)[PDGFB](http://www.ncbi.nlm.nih.gov/gene/?term=5155)(22.373) [MAP2K1](http://www.ncbi.nlm.nih.gov/gene/?term=5604)(22.373)[SLC22A3](http://www.ncbi.nlm.nih.gov/gene/?term=6581)(22.373)[NEFH](http://www.ncbi.nlm.nih.gov/gene/?term=4744)(22.373)[CRHR2](http://www.ncbi.nlm.nih.gov/gene/?term=1395)(22.373)[HCN2](http://www.ncbi.nlm.nih.gov/gene/?term=610)(22.373) |
| [Bisaborosaol A](http://www.megabionet.org/tcmid/ingredient/2417/) | [ESR1](http://www.ncbi.nlm.nih.gov/gene/?term=2099)(48.000)[PGR](http://www.ncbi.nlm.nih.gov/gene/?term=5241)(48.000)[AR](http://www.ncbi.nlm.nih.gov/gene/?term=367)(48.000)[NR3C1](http://www.ncbi.nlm.nih.gov/gene/?term=2908)(48.000)[ANXA1](http://www.ncbi.nlm.nih.gov/gene/?term=301)(48.000) [NR3C2](http://www.ncbi.nlm.nih.gov/gene/?term=4306)(48.000)[CHRM3](http://www.ncbi.nlm.nih.gov/gene/?term=1131)(23.000)[CHRM1](http://www.ncbi.nlm.nih.gov/gene/?term=1128)(23.000)[CHRM2](http://www.ncbi.nlm.nih.gov/gene/?term=1129)(23.000) |
| [4-Methoxy-1,2-Benzodioxole](http://www.megabionet.org/tcmid/ingredient/13842/) | This compound doesn't have any potential target with score larger than 20. |
| [Citronellyl Acetate](http://www.megabionet.org/tcmid/ingredient/3769/) | [ESR1](http://www.ncbi.nlm.nih.gov/gene/?term=2099)(48.000)[PGR](http://www.ncbi.nlm.nih.gov/gene/?term=5241)(48.000)[AR](http://www.ncbi.nlm.nih.gov/gene/?term=367)(48.000)[NR3C2](http://www.ncbi.nlm.nih.gov/gene/?term=4306)(48.000) |
| [Alphitolic Acid](http://www.megabionet.org/tcmid/ingredient/23870/) | [ESRRG](http://www.ncbi.nlm.nih.gov/gene/?term=2104)(48.000)[COX6C](http://www.ncbi.nlm.nih.gov/gene/?term=1345)(48.000)[COX5B](http://www.ncbi.nlm.nih.gov/gene/?term=1329)(48.000)[SLC8A1](http://www.ncbi.nlm.nih.gov/gene/?term=6546)(48.000)[COX7C](http://www.ncbi.nlm.nih.gov/gene/?term=1350)(48.000) [COX1](http://www.ncbi.nlm.nih.gov/gene/?term=4512)(48.000)[F12](http://www.ncbi.nlm.nih.gov/gene/?term=2161)(48.000)[AKR1C2](http://www.ncbi.nlm.nih.gov/gene/?term=1646)(48.000)[TRPV1](http://www.ncbi.nlm.nih.gov/gene/?term=7442)(48.000)[AKR1D1](http://www.ncbi.nlm.nih.gov/gene/?term=6718)(48.000) [COX5A](http://www.ncbi.nlm.nih.gov/gene/?term=9377)(48.000)[COX3](http://www.ncbi.nlm.nih.gov/gene/?term=4514)(48.000)[AR](http://www.ncbi.nlm.nih.gov/gene/?term=367)(48.000)[COX7A1](http://www.ncbi.nlm.nih.gov/gene/?term=1346)(48.000)[FECH](http://www.ncbi.nlm.nih.gov/gene/?term=2235)(48.000) [COX4I1](http://www.ncbi.nlm.nih.gov/gene/?term=1327)(48.000)[PLA2G1B](http://www.ncbi.nlm.nih.gov/gene/?term=5319)(48.000)[COX6A2](http://www.ncbi.nlm.nih.gov/gene/?term=1339)(48.000)[FADS2](http://www.ncbi.nlm.nih.gov/gene/?term=9415)(48.000)[TYR](http://www.ncbi.nlm.nih.gov/gene/?term=7299)(48.000) [FADS1](http://www.ncbi.nlm.nih.gov/gene/?term=3992)(48.000)[ADH1C](http://www.ncbi.nlm.nih.gov/gene/?term=126)(48.000)[PTGS1](http://www.ncbi.nlm.nih.gov/gene/?term=5742)(48.000)[COX6B1](http://www.ncbi.nlm.nih.gov/gene/?term=1340)(48.000)[FABP6](http://www.ncbi.nlm.nih.gov/gene/?term=2172)(48.000) [CES1](http://www.ncbi.nlm.nih.gov/gene/?term=1066)(48.000)[PTGS2](http://www.ncbi.nlm.nih.gov/gene/?term=5743)(48.000)[ELOVL4](http://www.ncbi.nlm.nih.gov/gene/?term=6785)(48.000)[SRD5A2](http://www.ncbi.nlm.nih.gov/gene/?term=6716)(48.000)[COX7B](http://www.ncbi.nlm.nih.gov/gene/?term=1349)(48.000) [COX2](http://www.ncbi.nlm.nih.gov/gene/?term=4513)(48.000)[COX8A](http://www.ncbi.nlm.nih.gov/gene/?term=1351)(48.000)[NR1H4](http://www.ncbi.nlm.nih.gov/gene/?term=9971)(48.000) |
| [Eugeniin](http://www.megabionet.org/tcmid/ingredient/7517/) | This compound doesn't have any potential target with score larger than 20. |
| [Chrysophanol Glucoside](http://www.megabionet.org/tcmid/ingredient/30777/) | This compound doesn't have any potential target with score larger than 20. |
| [Glaucine](http://www.megabionet.org/tcmid/ingredient/8512/) | [HTR2A](http://www.ncbi.nlm.nih.gov/gene/?term=3356)(122.778)[HTR2B](http://www.ncbi.nlm.nih.gov/gene/?term=3357)(122.778)[ADRA2A](http://www.ncbi.nlm.nih.gov/gene/?term=150)(122.778)[HTR1B](http://www.ncbi.nlm.nih.gov/gene/?term=3351)(122.778)[HTR2C](http://www.ncbi.nlm.nih.gov/gene/?term=3358)(122.778) [HTR1D](http://www.ncbi.nlm.nih.gov/gene/?term=3352)(122.778)[DRD2](http://www.ncbi.nlm.nih.gov/gene/?term=1813)(122.778)[DRD4](http://www.ncbi.nlm.nih.gov/gene/?term=1815)(122.778)[HTR1A](http://www.ncbi.nlm.nih.gov/gene/?term=3350)(122.778)[ADRA2C](http://www.ncbi.nlm.nih.gov/gene/?term=152)(122.778) [CALY](http://www.ncbi.nlm.nih.gov/gene/?term=50632)(122.778)[DRD3](http://www.ncbi.nlm.nih.gov/gene/?term=1814)(122.778)[DRD5](http://www.ncbi.nlm.nih.gov/gene/?term=1816)(122.778)[DRD1](http://www.ncbi.nlm.nih.gov/gene/?term=1812)(122.778)[ADRA2B](http://www.ncbi.nlm.nih.gov/gene/?term=151)(122.778) [CYBA](http://www.ncbi.nlm.nih.gov/gene/?term=1535)(80.882)[CHRNA4](http://www.ncbi.nlm.nih.gov/gene/?term=1137)(80.882)[RAC2](http://www.ncbi.nlm.nih.gov/gene/?term=5880)(80.882)[RAC1](http://www.ncbi.nlm.nih.gov/gene/?term=5879)(80.882)[OPRD1](http://www.ncbi.nlm.nih.gov/gene/?term=4985)(80.882) [NCF4](http://www.ncbi.nlm.nih.gov/gene/?term=4689)(80.882)[CHRNA7](http://www.ncbi.nlm.nih.gov/gene/?term=1139)(80.882)[CYBB](http://www.ncbi.nlm.nih.gov/gene/?term=1536)(80.882)[NCF1](http://www.ncbi.nlm.nih.gov/gene/?term=653361)(80.882)[SIGMAR1](http://www.ncbi.nlm.nih.gov/gene/?term=10280)(80.882) [NCF2](http://www.ncbi.nlm.nih.gov/gene/?term=4688)(80.882)[OPRK1](http://www.ncbi.nlm.nih.gov/gene/?term=4986)(80.882)[CHRNA3](http://www.ncbi.nlm.nih.gov/gene/?term=1136)(80.882)[RAC3](http://www.ncbi.nlm.nih.gov/gene/?term=5881)(80.882)[CHRNB2](http://www.ncbi.nlm.nih.gov/gene/?term=1141)(80.882) [SLC6A4](http://www.ncbi.nlm.nih.gov/gene/?term=6532)(80.882)[OPRM1](http://www.ncbi.nlm.nih.gov/gene/?term=4988)(80.882)[CHRNA2](http://www.ncbi.nlm.nih.gov/gene/?term=1135)(80.882)[PGRMC1](http://www.ncbi.nlm.nih.gov/gene/?term=10857)(80.882)[GRIN3A](http://www.ncbi.nlm.nih.gov/gene/?term=116443)(80.882) [CHRFAM7A](http://www.ncbi.nlm.nih.gov/gene/?term=89832)(80.882)[CHRNB4](http://www.ncbi.nlm.nih.gov/gene/?term=1143)(80.882)[SLC6A2](http://www.ncbi.nlm.nih.gov/gene/?term=6530)(80.882)[TLR4](http://www.ncbi.nlm.nih.gov/gene/?term=7099)(55.444)[ELANE](http://www.ncbi.nlm.nih.gov/gene/?term=1991)(55.444) [CTSG](http://www.ncbi.nlm.nih.gov/gene/?term=1511)(55.444)[SLC18A1](http://www.ncbi.nlm.nih.gov/gene/?term=6570)(55.444)[CEND1](http://www.ncbi.nlm.nih.gov/gene/?term=51286)(55.444)[SLC22A1](http://www.ncbi.nlm.nih.gov/gene/?term=6580)(55.444)[ADRA1B](http://www.ncbi.nlm.nih.gov/gene/?term=147)(55.444) [WLS](http://www.ncbi.nlm.nih.gov/gene/?term=79971)(55.444)[CHRNA9](http://www.ncbi.nlm.nih.gov/gene/?term=55584)(55.444)[ACHE](http://www.ncbi.nlm.nih.gov/gene/?term=43)(48.000)[SLC18A2](http://www.ncbi.nlm.nih.gov/gene/?term=6571)(48.000)[HTR3A](http://www.ncbi.nlm.nih.gov/gene/?term=3359)(48.000) [CHRM2](http://www.ncbi.nlm.nih.gov/gene/?term=1129)(48.000)[ADRA1A](http://www.ncbi.nlm.nih.gov/gene/?term=148)(23.000)[CHRM3](http://www.ncbi.nlm.nih.gov/gene/?term=1131)(23.000)[CHRM1](http://www.ncbi.nlm.nih.gov/gene/?term=1128)(23.000)[HTR6](http://www.ncbi.nlm.nih.gov/gene/?term=3362)(23.000) [ADRA1D](http://www.ncbi.nlm.nih.gov/gene/?term=146)(23.000)[CHRM5](http://www.ncbi.nlm.nih.gov/gene/?term=1133)(23.000)[HRH4](http://www.ncbi.nlm.nih.gov/gene/?term=59340)(23.000)[HRH1](http://www.ncbi.nlm.nih.gov/gene/?term=3269)(23.000)[KCNH2](http://www.ncbi.nlm.nih.gov/gene/?term=3757)(23.000) [HRH2](http://www.ncbi.nlm.nih.gov/gene/?term=3274)(23.000)[CHRM4](http://www.ncbi.nlm.nih.gov/gene/?term=1132)(23.000)[ACTN3](http://www.ncbi.nlm.nih.gov/gene/?term=89)(22.373)[GPM6B](http://www.ncbi.nlm.nih.gov/gene/?term=2824)(22.373)[HMOX1](http://www.ncbi.nlm.nih.gov/gene/?term=3162)(22.373) [TAC1](http://www.ncbi.nlm.nih.gov/gene/?term=6863)(22.373)[MC2R](http://www.ncbi.nlm.nih.gov/gene/?term=4158)(22.373)[SLITRK6](http://www.ncbi.nlm.nih.gov/gene/?term=84189)(22.373)[UTS2](http://www.ncbi.nlm.nih.gov/gene/?term=10911)(22.373)[NAV2](http://www.ncbi.nlm.nih.gov/gene/?term=89797)(22.373) [PDE3A](http://www.ncbi.nlm.nih.gov/gene/?term=5139)(22.373)[SCT](http://www.ncbi.nlm.nih.gov/gene/?term=6343)(22.373)[TUSC2](http://www.ncbi.nlm.nih.gov/gene/?term=11334)(22.373)[RHOA](http://www.ncbi.nlm.nih.gov/gene/?term=387)(22.373)[AVPR1A](http://www.ncbi.nlm.nih.gov/gene/?term=552)(22.373) [ADORA2A](http://www.ncbi.nlm.nih.gov/gene/?term=135)(22.373)[HCN4](http://www.ncbi.nlm.nih.gov/gene/?term=10021)(22.373)[PHKG2](http://www.ncbi.nlm.nih.gov/gene/?term=5261)(22.373)[OXT](http://www.ncbi.nlm.nih.gov/gene/?term=5020)(22.373)[CACNA1A](http://www.ncbi.nlm.nih.gov/gene/?term=773)(22.373) [CCL5](http://www.ncbi.nlm.nih.gov/gene/?term=6352)(22.373)[APLP1](http://www.ncbi.nlm.nih.gov/gene/?term=333)(22.373)[LAMP1](http://www.ncbi.nlm.nih.gov/gene/?term=3916)(22.373)[PPP1R9B](http://www.ncbi.nlm.nih.gov/gene/?term=84687)(22.373)[YWHAE](http://www.ncbi.nlm.nih.gov/gene/?term=7531)(22.373) [SUMO1](http://www.ncbi.nlm.nih.gov/gene/?term=7341)(22.373)[CNR2](http://www.ncbi.nlm.nih.gov/gene/?term=1269)(22.373)[NOXA1](http://www.ncbi.nlm.nih.gov/gene/?term=10811)(22.373)[MC1R](http://www.ncbi.nlm.nih.gov/gene/?term=4157)(22.373)[MC4R](http://www.ncbi.nlm.nih.gov/gene/?term=4160)(22.373) [IL1B](http://www.ncbi.nlm.nih.gov/gene/?term=3553)(22.373)[AGT](http://www.ncbi.nlm.nih.gov/gene/?term=183)(22.373)[CRHBP](http://www.ncbi.nlm.nih.gov/gene/?term=1393)(22.373)[F2R](http://www.ncbi.nlm.nih.gov/gene/?term=2149)(22.373)[MTNR1B](http://www.ncbi.nlm.nih.gov/gene/?term=4544)(22.373) [FGF10](http://www.ncbi.nlm.nih.gov/gene/?term=2255)(22.373)[EGR1](http://www.ncbi.nlm.nih.gov/gene/?term=1958)(22.373)[NOXO1](http://www.ncbi.nlm.nih.gov/gene/?term=124056)(22.373)[ADAM8](http://www.ncbi.nlm.nih.gov/gene/?term=101)(22.373)[SLC44A4](http://www.ncbi.nlm.nih.gov/gene/?term=80736)(22.373) [FLOT1](http://www.ncbi.nlm.nih.gov/gene/?term=10211)(22.373)[NPY5R](http://www.ncbi.nlm.nih.gov/gene/?term=4889)(22.373)[VCP](http://www.ncbi.nlm.nih.gov/gene/?term=7415)(22.373)[GHRL](http://www.ncbi.nlm.nih.gov/gene/?term=51738)(22.373)[TBR1](http://www.ncbi.nlm.nih.gov/gene/?term=10716)(22.373) [PTPRO](http://www.ncbi.nlm.nih.gov/gene/?term=5800)(22.373)[OXTR](http://www.ncbi.nlm.nih.gov/gene/?term=5021)(22.373)[PAX2](http://www.ncbi.nlm.nih.gov/gene/?term=5076)(22.373)[CACNA1C](http://www.ncbi.nlm.nih.gov/gene/?term=775)(22.373)[F2RL1](http://www.ncbi.nlm.nih.gov/gene/?term=2150)(22.373) [UTS2R](http://www.ncbi.nlm.nih.gov/gene/?term=2837)(22.373)[ACVR2A](http://www.ncbi.nlm.nih.gov/gene/?term=92)(22.373)[RAPGEF2](http://www.ncbi.nlm.nih.gov/gene/?term=9693)(22.373)[TIRAP](http://www.ncbi.nlm.nih.gov/gene/?term=114609)(22.373)[AIF1](http://www.ncbi.nlm.nih.gov/gene/?term=199)(22.373) [GHRH](http://www.ncbi.nlm.nih.gov/gene/?term=2691)(22.373)[HCRT](http://www.ncbi.nlm.nih.gov/gene/?term=3060)(22.373)[RAB3B](http://www.ncbi.nlm.nih.gov/gene/?term=5865)(22.373)[CNR1](http://www.ncbi.nlm.nih.gov/gene/?term=1268)(22.373)[HIF1A](http://www.ncbi.nlm.nih.gov/gene/?term=3091)(22.373) [INS](http://www.ncbi.nlm.nih.gov/gene/?term=3630)(22.373)[ACE](http://www.ncbi.nlm.nih.gov/gene/?term=1636)(22.373)[SLC18A3](http://www.ncbi.nlm.nih.gov/gene/?term=6572)(22.373)[BNIP3](http://www.ncbi.nlm.nih.gov/gene/?term=664)(22.373)[CRH](http://www.ncbi.nlm.nih.gov/gene/?term=1392)(22.373) [GLI3](http://www.ncbi.nlm.nih.gov/gene/?term=2737)(22.373)[PLXNB3](http://www.ncbi.nlm.nih.gov/gene/?term=5365)(22.373)[CAV3](http://www.ncbi.nlm.nih.gov/gene/?term=859)(22.373)[NEFL](http://www.ncbi.nlm.nih.gov/gene/?term=4747)(22.373)[UCN2](http://www.ncbi.nlm.nih.gov/gene/?term=90226)(22.373) [IL6](http://www.ncbi.nlm.nih.gov/gene/?term=3569)(22.373)[RNF207](http://www.ncbi.nlm.nih.gov/gene/?term=388591)(22.373)[TACR2](http://www.ncbi.nlm.nih.gov/gene/?term=6865)(22.373)[PTGS2](http://www.ncbi.nlm.nih.gov/gene/?term=5743)(22.373)[NRP1](http://www.ncbi.nlm.nih.gov/gene/?term=8829)(22.373) [NPPA](http://www.ncbi.nlm.nih.gov/gene/?term=4878)(22.373)[MC5R](http://www.ncbi.nlm.nih.gov/gene/?term=4161)(22.373)[AQP1](http://www.ncbi.nlm.nih.gov/gene/?term=358)(22.373)[MC3R](http://www.ncbi.nlm.nih.gov/gene/?term=4159)(22.373)[TAC4](http://www.ncbi.nlm.nih.gov/gene/?term=255061)(22.373) [TNR](http://www.ncbi.nlm.nih.gov/gene/?term=7143)(22.373)[NOS3](http://www.ncbi.nlm.nih.gov/gene/?term=4846)(22.373)[EPHB1](http://www.ncbi.nlm.nih.gov/gene/?term=2047)(22.373)[SNCA](http://www.ncbi.nlm.nih.gov/gene/?term=6622)(22.373)[PDE2A](http://www.ncbi.nlm.nih.gov/gene/?term=5138)(22.373) [CD34](http://www.ncbi.nlm.nih.gov/gene/?term=947)(22.373)[KCNMA1](http://www.ncbi.nlm.nih.gov/gene/?term=3778)(22.373)[POU4F3](http://www.ncbi.nlm.nih.gov/gene/?term=5459)(22.373)[CBFA2T3](http://www.ncbi.nlm.nih.gov/gene/?term=863)(22.373)[CALCA](http://www.ncbi.nlm.nih.gov/gene/?term=796)(22.373) [PINK1](http://www.ncbi.nlm.nih.gov/gene/?term=65018)(22.373)[NOS1](http://www.ncbi.nlm.nih.gov/gene/?term=4842)(22.373)[TREM1](http://www.ncbi.nlm.nih.gov/gene/?term=54210)(22.373)[GJA5](http://www.ncbi.nlm.nih.gov/gene/?term=2702)(22.373)[PDGFB](http://www.ncbi.nlm.nih.gov/gene/?term=5155)(22.373) [CARTPT](http://www.ncbi.nlm.nih.gov/gene/?term=9607)(22.373)[NEFH](http://www.ncbi.nlm.nih.gov/gene/?term=4744)(22.373)[ATP8B1](http://www.ncbi.nlm.nih.gov/gene/?term=5205)(22.373)[KISS1](http://www.ncbi.nlm.nih.gov/gene/?term=3814)(22.373)[GRM7](http://www.ncbi.nlm.nih.gov/gene/?term=2917)(22.373) [FOS](http://www.ncbi.nlm.nih.gov/gene/?term=2353)(22.373)[CRHR2](http://www.ncbi.nlm.nih.gov/gene/?term=1395)(22.373)[GRIN1](http://www.ncbi.nlm.nih.gov/gene/?term=2902)(22.373)[HCN2](http://www.ncbi.nlm.nih.gov/gene/?term=610)(22.373) |
| [Beta-Boswellic Acid](http://www.megabionet.org/tcmid/ingredient/30677/) | [SRD5A1](http://www.ncbi.nlm.nih.gov/gene/?term=6715)(48.000)[CYP17A1](http://www.ncbi.nlm.nih.gov/gene/?term=1586)(48.000)[SLC8A1](http://www.ncbi.nlm.nih.gov/gene/?term=6546)(48.000)[F12](http://www.ncbi.nlm.nih.gov/gene/?term=2161)(48.000)[ESR1](http://www.ncbi.nlm.nih.gov/gene/?term=2099)(48.000) [TRPV1](http://www.ncbi.nlm.nih.gov/gene/?term=7442)(48.000)[PTGER1](http://www.ncbi.nlm.nih.gov/gene/?term=5731)(48.000)[PTGER4](http://www.ncbi.nlm.nih.gov/gene/?term=5734)(48.000)[PGR](http://www.ncbi.nlm.nih.gov/gene/?term=5241)(48.000)[AR](http://www.ncbi.nlm.nih.gov/gene/?term=367)(48.000) [NR3C1](http://www.ncbi.nlm.nih.gov/gene/?term=2908)(48.000)[OPRK1](http://www.ncbi.nlm.nih.gov/gene/?term=4986)(48.000)[FADS2](http://www.ncbi.nlm.nih.gov/gene/?term=9415)(48.000)[FADS1](http://www.ncbi.nlm.nih.gov/gene/?term=3992)(48.000)[PTGER2](http://www.ncbi.nlm.nih.gov/gene/?term=5732)(48.000) [PTGS1](http://www.ncbi.nlm.nih.gov/gene/?term=5742)(48.000)[PTGER3](http://www.ncbi.nlm.nih.gov/gene/?term=5733)(48.000)[PTGS2](http://www.ncbi.nlm.nih.gov/gene/?term=5743)(48.000)[ELOVL4](http://www.ncbi.nlm.nih.gov/gene/?term=6785)(48.000)[ANXA1](http://www.ncbi.nlm.nih.gov/gene/?term=301)(48.000) [NR3C2](http://www.ncbi.nlm.nih.gov/gene/?term=4306)(48.000)[ESRRG](http://www.ncbi.nlm.nih.gov/gene/?term=2104)(23.000)[COX6C](http://www.ncbi.nlm.nih.gov/gene/?term=1345)(23.000)[COX5B](http://www.ncbi.nlm.nih.gov/gene/?term=1329)(23.000)[COX7C](http://www.ncbi.nlm.nih.gov/gene/?term=1350)(23.000) [COX1](http://www.ncbi.nlm.nih.gov/gene/?term=4512)(23.000)[AKR1C2](http://www.ncbi.nlm.nih.gov/gene/?term=1646)(23.000)[ADH1B](http://www.ncbi.nlm.nih.gov/gene/?term=125)(23.000)[COX5A](http://www.ncbi.nlm.nih.gov/gene/?term=9377)(23.000)[COX3](http://www.ncbi.nlm.nih.gov/gene/?term=4514)(23.000) [COX7A1](http://www.ncbi.nlm.nih.gov/gene/?term=1346)(23.000)[FECH](http://www.ncbi.nlm.nih.gov/gene/?term=2235)(23.000)[COX4I1](http://www.ncbi.nlm.nih.gov/gene/?term=1327)(23.000)[PLA2G1B](http://www.ncbi.nlm.nih.gov/gene/?term=5319)(23.000)[AKR1C1](http://www.ncbi.nlm.nih.gov/gene/?term=1645)(23.000) [COX6A2](http://www.ncbi.nlm.nih.gov/gene/?term=1339)(23.000)[HMGCR](http://www.ncbi.nlm.nih.gov/gene/?term=3156)(23.000)[ADH1C](http://www.ncbi.nlm.nih.gov/gene/?term=126)(23.000)[COX6B1](http://www.ncbi.nlm.nih.gov/gene/?term=1340)(23.000)[FABP6](http://www.ncbi.nlm.nih.gov/gene/?term=2172)(23.000) [CES1](http://www.ncbi.nlm.nih.gov/gene/?term=1066)(23.000)[COX7B](http://www.ncbi.nlm.nih.gov/gene/?term=1349)(23.000)[COX2](http://www.ncbi.nlm.nih.gov/gene/?term=4513)(23.000)[COX8A](http://www.ncbi.nlm.nih.gov/gene/?term=1351)(23.000)[NR1H4](http://www.ncbi.nlm.nih.gov/gene/?term=9971)(23.000) |
| [Mutatochrome](http://www.megabionet.org/tcmid/ingredient/15127/) | This compound doesn't have any potential target with score larger than 20. |
| [Phenylbutanone-Glucoside](http://www.megabionet.org/tcmid/ingredient/17085/) | This compound doesn't have any potential target with score larger than 20. |
| [Tellimagrandin I](http://www.megabionet.org/tcmid/ingredient/20894/) | This compound doesn't have any potential target with score larger than 20. |
| [Aloeemodin](http://www.megabionet.org/tcmid/ingredient/967/) | [CHRM3](http://www.ncbi.nlm.nih.gov/gene/?term=1131)(23.000)[CHRM1](http://www.ncbi.nlm.nih.gov/gene/?term=1128)(23.000)[CHRM2](http://www.ncbi.nlm.nih.gov/gene/?term=1129)(23.000)[CHRM4](http://www.ncbi.nlm.nih.gov/gene/?term=1132)(23.000) |
| [Methyl Salicylate](http://www.megabionet.org/tcmid/ingredient/14710/) | [AKR1C1](http://www.ncbi.nlm.nih.gov/gene/?term=1645)(122.778)[PTGS1](http://www.ncbi.nlm.nih.gov/gene/?term=5742)(122.778)[PTGS2](http://www.ncbi.nlm.nih.gov/gene/?term=5743)(122.778)[PRKAB1](http://www.ncbi.nlm.nih.gov/gene/?term=5564)(48.000)[PRKAG2](http://www.ncbi.nlm.nih.gov/gene/?term=51422)(48.000) [NFKB1](http://www.ncbi.nlm.nih.gov/gene/?term=4790)(48.000)[EDNRA](http://www.ncbi.nlm.nih.gov/gene/?term=1909)(48.000)[PLA2G2E](http://www.ncbi.nlm.nih.gov/gene/?term=30814)(48.000)[NFKBIA](http://www.ncbi.nlm.nih.gov/gene/?term=4792)(48.000)[TP53](http://www.ncbi.nlm.nih.gov/gene/?term=7157)(48.000) [NFKB2](http://www.ncbi.nlm.nih.gov/gene/?term=4791)(48.000)[PPARG](http://www.ncbi.nlm.nih.gov/gene/?term=5468)(48.000)[HSPA5](http://www.ncbi.nlm.nih.gov/gene/?term=3309)(48.000)[MPO](http://www.ncbi.nlm.nih.gov/gene/?term=4353)(48.000)[PRKAA2](http://www.ncbi.nlm.nih.gov/gene/?term=5563)(48.000) [PRKAG1](http://www.ncbi.nlm.nih.gov/gene/?term=5571)(48.000)[PRKAA1](http://www.ncbi.nlm.nih.gov/gene/?term=5562)(48.000)[PRKAG3](http://www.ncbi.nlm.nih.gov/gene/?term=53632)(48.000)[ALOX5](http://www.ncbi.nlm.nih.gov/gene/?term=240)(48.000)[IKBKB](http://www.ncbi.nlm.nih.gov/gene/?term=3551)(48.000) [CHUK](http://www.ncbi.nlm.nih.gov/gene/?term=1147)(48.000)[PRKAB2](http://www.ncbi.nlm.nih.gov/gene/?term=5565)(48.000)[RPS6KA3](http://www.ncbi.nlm.nih.gov/gene/?term=6197)(48.000) |
| [Isocembrol](http://www.megabionet.org/tcmid/ingredient/11311/) | [ESR1](http://www.ncbi.nlm.nih.gov/gene/?term=2099)(80.882)[PGR](http://www.ncbi.nlm.nih.gov/gene/?term=5241)(80.882)[WNT4](http://www.ncbi.nlm.nih.gov/gene/?term=54361)(55.444)[TFAP2C](http://www.ncbi.nlm.nih.gov/gene/?term=7022)(22.373)[TNFSF11](http://www.ncbi.nlm.nih.gov/gene/?term=8600)(22.373) [PHB](http://www.ncbi.nlm.nih.gov/gene/?term=5245)(22.373)[TOX3](http://www.ncbi.nlm.nih.gov/gene/?term=27324)(22.373)[TGFB1](http://www.ncbi.nlm.nih.gov/gene/?term=7040)(22.373)[MED1](http://www.ncbi.nlm.nih.gov/gene/?term=5469)(22.373)[NKX3-1](http://www.ncbi.nlm.nih.gov/gene/?term=4824)(22.373) [ESR2](http://www.ncbi.nlm.nih.gov/gene/?term=2100)(22.373)[NEDD4](http://www.ncbi.nlm.nih.gov/gene/?term=4734)(22.373)[SHH](http://www.ncbi.nlm.nih.gov/gene/?term=6469)(22.373)[SERPINB3](http://www.ncbi.nlm.nih.gov/gene/?term=6317)(22.373)[LEF1](http://www.ncbi.nlm.nih.gov/gene/?term=51176)(22.373) [FGFR2](http://www.ncbi.nlm.nih.gov/gene/?term=2263)(22.373)[SRC](http://www.ncbi.nlm.nih.gov/gene/?term=6714)(22.373)[UBR5](http://www.ncbi.nlm.nih.gov/gene/?term=51366)(22.373)[AREG](http://www.ncbi.nlm.nih.gov/gene/?term=374)(22.373)[CD34](http://www.ncbi.nlm.nih.gov/gene/?term=947)(22.373) [GPER1](http://www.ncbi.nlm.nih.gov/gene/?term=2852)(22.373)[WNT5A](http://www.ncbi.nlm.nih.gov/gene/?term=7474)(22.373)[SOX9](http://www.ncbi.nlm.nih.gov/gene/?term=6662)(22.373)[PDGFB](http://www.ncbi.nlm.nih.gov/gene/?term=5155)(22.373)[VDR](http://www.ncbi.nlm.nih.gov/gene/?term=7421)(22.373) [TRIM24](http://www.ncbi.nlm.nih.gov/gene/?term=8805)(22.373) |
| [Commisterone](http://www.megabionet.org/tcmid/ingredient/3944/) | [AR](http://www.ncbi.nlm.nih.gov/gene/?term=367)(122.778)[SRD5A1](http://www.ncbi.nlm.nih.gov/gene/?term=6715)(48.000)[CYP17A1](http://www.ncbi.nlm.nih.gov/gene/?term=1586)(48.000)[PRLR](http://www.ncbi.nlm.nih.gov/gene/?term=5618)(48.000)[ESR1](http://www.ncbi.nlm.nih.gov/gene/?term=2099)(48.000) [PTGER1](http://www.ncbi.nlm.nih.gov/gene/?term=5731)(48.000)[PTGER4](http://www.ncbi.nlm.nih.gov/gene/?term=5734)(48.000)[PGR](http://www.ncbi.nlm.nih.gov/gene/?term=5241)(48.000)[NR3C1](http://www.ncbi.nlm.nih.gov/gene/?term=2908)(48.000)[OPRK1](http://www.ncbi.nlm.nih.gov/gene/?term=4986)(48.000) [PTGER2](http://www.ncbi.nlm.nih.gov/gene/?term=5732)(48.000)[PTGER3](http://www.ncbi.nlm.nih.gov/gene/?term=5733)(48.000)[ANXA1](http://www.ncbi.nlm.nih.gov/gene/?term=301)(48.000)[NR3C2](http://www.ncbi.nlm.nih.gov/gene/?term=4306)(48.000) |
| [Methyl Phenylacetate](http://www.megabionet.org/tcmid/ingredient/14661/) | [SCN10A](http://www.ncbi.nlm.nih.gov/gene/?term=6336)(80.882)[ACHE](http://www.ncbi.nlm.nih.gov/gene/?term=43)(80.882)[BCHE](http://www.ncbi.nlm.nih.gov/gene/?term=590)(80.882)[COLQ](http://www.ncbi.nlm.nih.gov/gene/?term=8292)(55.444)[PLAT](http://www.ncbi.nlm.nih.gov/gene/?term=5327)(48.000) [THBD](http://www.ncbi.nlm.nih.gov/gene/?term=7056)(48.000)[PPARG](http://www.ncbi.nlm.nih.gov/gene/?term=5468)(48.000)[BCL2](http://www.ncbi.nlm.nih.gov/gene/?term=596)(48.000)[FABP2](http://www.ncbi.nlm.nih.gov/gene/?term=2169)(48.000)[CFTR](http://www.ncbi.nlm.nih.gov/gene/?term=1080)(48.000) [PTGS1](http://www.ncbi.nlm.nih.gov/gene/?term=5742)(48.000)[PTGS2](http://www.ncbi.nlm.nih.gov/gene/?term=5743)(48.000)[CXCR1](http://www.ncbi.nlm.nih.gov/gene/?term=3577)(48.000)[SLC5A7](http://www.ncbi.nlm.nih.gov/gene/?term=60482)(22.373)[NRG1](http://www.ncbi.nlm.nih.gov/gene/?term=3084)(22.373) [DMGDH](http://www.ncbi.nlm.nih.gov/gene/?term=29958)(22.373)[SIX3](http://www.ncbi.nlm.nih.gov/gene/?term=6496)(22.373)[ANK2](http://www.ncbi.nlm.nih.gov/gene/?term=287)(22.373)[CRP](http://www.ncbi.nlm.nih.gov/gene/?term=1401)(22.373)[SCN5A](http://www.ncbi.nlm.nih.gov/gene/?term=6331)(22.373) [PRSS12](http://www.ncbi.nlm.nih.gov/gene/?term=8492)(22.373)[SLC44A4](http://www.ncbi.nlm.nih.gov/gene/?term=80736)(22.373)[ALDH7A1](http://www.ncbi.nlm.nih.gov/gene/?term=501)(22.373)[DNM3](http://www.ncbi.nlm.nih.gov/gene/?term=26052)(22.373)[SCN4B](http://www.ncbi.nlm.nih.gov/gene/?term=6330)(22.373) [ASCL1](http://www.ncbi.nlm.nih.gov/gene/?term=429)(22.373)[ENPP6](http://www.ncbi.nlm.nih.gov/gene/?term=133121)(22.373)[CDH8](http://www.ncbi.nlm.nih.gov/gene/?term=1006)(22.373)[KCNA5](http://www.ncbi.nlm.nih.gov/gene/?term=3741)(22.373)[FNTA](http://www.ncbi.nlm.nih.gov/gene/?term=2339)(22.373) [AGRN](http://www.ncbi.nlm.nih.gov/gene/?term=375790)(22.373)[CHKA](http://www.ncbi.nlm.nih.gov/gene/?term=1119)(22.373)[RYR2](http://www.ncbi.nlm.nih.gov/gene/?term=6262)(22.373)[CHDH](http://www.ncbi.nlm.nih.gov/gene/?term=55349)(22.373)[GJA5](http://www.ncbi.nlm.nih.gov/gene/?term=2702)(22.373) [GRIN1](http://www.ncbi.nlm.nih.gov/gene/?term=2902)(22.373) |
| [Beta-Humulene](http://www.megabionet.org/tcmid/ingredient/31122/) | [KCND1](http://www.ncbi.nlm.nih.gov/gene/?term=3750)(80.882)[KCNA3](http://www.ncbi.nlm.nih.gov/gene/?term=3738)(80.882)[PRKAB1](http://www.ncbi.nlm.nih.gov/gene/?term=5564)(80.882)[ADH1A](http://www.ncbi.nlm.nih.gov/gene/?term=124)(80.882)[KCNA10](http://www.ncbi.nlm.nih.gov/gene/?term=3744)(80.882) [GAMT](http://www.ncbi.nlm.nih.gov/gene/?term=2593)(80.882)[KCNC3](http://www.ncbi.nlm.nih.gov/gene/?term=3748)(80.882)[KCNA1](http://www.ncbi.nlm.nih.gov/gene/?term=3736)(80.882)[KCNA2](http://www.ncbi.nlm.nih.gov/gene/?term=3737)(80.882)[TPO](http://www.ncbi.nlm.nih.gov/gene/?term=7173)(80.882) [CAT](http://www.ncbi.nlm.nih.gov/gene/?term=847)(80.882)[KCNB1](http://www.ncbi.nlm.nih.gov/gene/?term=3745)(80.882)[ADH1B](http://www.ncbi.nlm.nih.gov/gene/?term=125)(80.882)[DLG4](http://www.ncbi.nlm.nih.gov/gene/?term=1742)(80.882)[KCNC2](http://www.ncbi.nlm.nih.gov/gene/?term=3747)(80.882) [KCNC1](http://www.ncbi.nlm.nih.gov/gene/?term=3746)(80.882)[ADH1C](http://www.ncbi.nlm.nih.gov/gene/?term=126)(80.882)[KCNB2](http://www.ncbi.nlm.nih.gov/gene/?term=9312)(80.882)[KCNA5](http://www.ncbi.nlm.nih.gov/gene/?term=3741)(80.882)[RNASE1](http://www.ncbi.nlm.nih.gov/gene/?term=6035)(80.882) [KCND2](http://www.ncbi.nlm.nih.gov/gene/?term=3751)(80.882)[ALDH2](http://www.ncbi.nlm.nih.gov/gene/?term=217)(80.882)[KCNA4](http://www.ncbi.nlm.nih.gov/gene/?term=3739)(80.882)[KCNA7](http://www.ncbi.nlm.nih.gov/gene/?term=3743)(80.882)[KCNA6](http://www.ncbi.nlm.nih.gov/gene/?term=3742)(80.882) [KCND3](http://www.ncbi.nlm.nih.gov/gene/?term=3752)(80.882)[GUCY1B3](http://www.ncbi.nlm.nih.gov/gene/?term=2983)(55.444)[GATM](http://www.ncbi.nlm.nih.gov/gene/?term=2628)(55.444)[KCNK4](http://www.ncbi.nlm.nih.gov/gene/?term=50801)(55.444)[IYD](http://www.ncbi.nlm.nih.gov/gene/?term=389434)(55.444) [KCNQ1](http://www.ncbi.nlm.nih.gov/gene/?term=3784)(55.444)[FBP1](http://www.ncbi.nlm.nih.gov/gene/?term=2203)(22.373)[DGKI](http://www.ncbi.nlm.nih.gov/gene/?term=9162)(22.373)[ADH7](http://www.ncbi.nlm.nih.gov/gene/?term=131)(22.373)[NFIB](http://www.ncbi.nlm.nih.gov/gene/?term=4781)(22.373) [CYGB](http://www.ncbi.nlm.nih.gov/gene/?term=114757)(22.373)[NRXN3](http://www.ncbi.nlm.nih.gov/gene/?term=9369)(22.373)[LRRC4B](http://www.ncbi.nlm.nih.gov/gene/?term=94030)(22.373)[AQP8](http://www.ncbi.nlm.nih.gov/gene/?term=343)(22.373)[ARX](http://www.ncbi.nlm.nih.gov/gene/?term=170302)(22.373) [IGF1](http://www.ncbi.nlm.nih.gov/gene/?term=3479)(22.373)[SHANK3](http://www.ncbi.nlm.nih.gov/gene/?term=85358)(22.373)[ACY3](http://www.ncbi.nlm.nih.gov/gene/?term=91703)(22.373)[ASPA](http://www.ncbi.nlm.nih.gov/gene/?term=443)(22.373)[NRXN1](http://www.ncbi.nlm.nih.gov/gene/?term=9378)(22.373) [EDN1](http://www.ncbi.nlm.nih.gov/gene/?term=1906)(22.373)[OXT](http://www.ncbi.nlm.nih.gov/gene/?term=5020)(22.373)[MAGI2](http://www.ncbi.nlm.nih.gov/gene/?term=9863)(22.373)[APOE](http://www.ncbi.nlm.nih.gov/gene/?term=348)(22.373)[SOX15](http://www.ncbi.nlm.nih.gov/gene/?term=6665)(22.373) [CNTNAP4](http://www.ncbi.nlm.nih.gov/gene/?term=85445)(22.373)[RNASE4](http://www.ncbi.nlm.nih.gov/gene/?term=6038)(22.373)[RAB3A](http://www.ncbi.nlm.nih.gov/gene/?term=5864)(22.373)[MC4R](http://www.ncbi.nlm.nih.gov/gene/?term=4160)(22.373)[IL1B](http://www.ncbi.nlm.nih.gov/gene/?term=3553)(22.373) [GRIN2A](http://www.ncbi.nlm.nih.gov/gene/?term=2903)(22.373)[SLC17A7](http://www.ncbi.nlm.nih.gov/gene/?term=57030)(22.373)[CYP11A1](http://www.ncbi.nlm.nih.gov/gene/?term=1583)(22.373)[SCN5A](http://www.ncbi.nlm.nih.gov/gene/?term=6331)(22.373)[SCN10A](http://www.ncbi.nlm.nih.gov/gene/?term=6336)(22.373) [SPARC](http://www.ncbi.nlm.nih.gov/gene/?term=6678)(22.373)[SORCS3](http://www.ncbi.nlm.nih.gov/gene/?term=22986)(22.373)[RNASE8](http://www.ncbi.nlm.nih.gov/gene/?term=122665)(22.373)[KCNIP2](http://www.ncbi.nlm.nih.gov/gene/?term=30819)(22.373)[GPX7](http://www.ncbi.nlm.nih.gov/gene/?term=2882)(22.373) [CRLF1](http://www.ncbi.nlm.nih.gov/gene/?term=9244)(22.373)[FADD](http://www.ncbi.nlm.nih.gov/gene/?term=8772)(22.373)[SNTG2](http://www.ncbi.nlm.nih.gov/gene/?term=54221)(22.373)[SCGB1A1](http://www.ncbi.nlm.nih.gov/gene/?term=7356)(22.373)[OXTR](http://www.ncbi.nlm.nih.gov/gene/?term=5021)(22.373) [ALDH1B1](http://www.ncbi.nlm.nih.gov/gene/?term=219)(22.373)[LEP](http://www.ncbi.nlm.nih.gov/gene/?term=3952)(22.373)[NLGN1](http://www.ncbi.nlm.nih.gov/gene/?term=22871)(22.373)[CACNA1D](http://www.ncbi.nlm.nih.gov/gene/?term=776)(22.373)[RAPGEF2](http://www.ncbi.nlm.nih.gov/gene/?term=9693)(22.373) [DARS](http://www.ncbi.nlm.nih.gov/gene/?term=1615)(22.373)[ANK3](http://www.ncbi.nlm.nih.gov/gene/?term=288)(22.373)[KCNE5](http://www.ncbi.nlm.nih.gov/gene/?term=23630)(22.373)[PPARD](http://www.ncbi.nlm.nih.gov/gene/?term=5467)(22.373)[NKX2-1](http://www.ncbi.nlm.nih.gov/gene/?term=7080)(22.373) [MIP](http://www.ncbi.nlm.nih.gov/gene/?term=4284)(22.373)[RYR3](http://www.ncbi.nlm.nih.gov/gene/?term=6263)(22.373)[ZPR1](http://www.ncbi.nlm.nih.gov/gene/?term=8882)(22.373)[ALDH3B1](http://www.ncbi.nlm.nih.gov/gene/?term=221)(22.373)[NPPA](http://www.ncbi.nlm.nih.gov/gene/?term=4878)(22.373) [STX1A](http://www.ncbi.nlm.nih.gov/gene/?term=6804)(22.373)[PAXBP1](http://www.ncbi.nlm.nih.gov/gene/?term=94104)(22.373)[PAX7](http://www.ncbi.nlm.nih.gov/gene/?term=5081)(22.373)[ALDH3B2](http://www.ncbi.nlm.nih.gov/gene/?term=222)(22.373)[RNASE2](http://www.ncbi.nlm.nih.gov/gene/?term=6036)(22.373) [NRXN2](http://www.ncbi.nlm.nih.gov/gene/?term=9379)(22.373)[ADORA1](http://www.ncbi.nlm.nih.gov/gene/?term=134)(22.373)[ADH4](http://www.ncbi.nlm.nih.gov/gene/?term=127)(22.373)[GJA5](http://www.ncbi.nlm.nih.gov/gene/?term=2702)(22.373)[FAS](http://www.ncbi.nlm.nih.gov/gene/?term=355)(22.373) [DAB2IP](http://www.ncbi.nlm.nih.gov/gene/?term=153090)(22.373)[CYP2E1](http://www.ncbi.nlm.nih.gov/gene/?term=1571)(22.373)[ACY1](http://www.ncbi.nlm.nih.gov/gene/?term=95)(22.373) |
| [5-Carboxy-7-Hydroxy-2-Methyl-Benzopyran-Gamma-One](http://www.megabionet.org/tcmid/ingredient/30740/) | [S100P](http://www.ncbi.nlm.nih.gov/gene/?term=6286)(48.000)[KCNMA1](http://www.ncbi.nlm.nih.gov/gene/?term=3778)(48.000) |
| [Protoporphyrin](http://www.megabionet.org/tcmid/ingredient/17970/) | [RXRB](http://www.ncbi.nlm.nih.gov/gene/?term=6257)(80.882)[PPARG](http://www.ncbi.nlm.nih.gov/gene/?term=5468)(80.882)[RXRG](http://www.ncbi.nlm.nih.gov/gene/?term=6258)(80.882)[AGTR1](http://www.ncbi.nlm.nih.gov/gene/?term=185)(80.882)[RXRA](http://www.ncbi.nlm.nih.gov/gene/?term=6256)(80.882) [APOA2](http://www.ncbi.nlm.nih.gov/gene/?term=336)(55.444)[IL1B](http://www.ncbi.nlm.nih.gov/gene/?term=3553)(55.444)[TNF](http://www.ncbi.nlm.nih.gov/gene/?term=7124)(55.444)[IL13](http://www.ncbi.nlm.nih.gov/gene/?term=3596)(55.444)[ACTN2](http://www.ncbi.nlm.nih.gov/gene/?term=88)(55.444) [FCGR1A](http://www.ncbi.nlm.nih.gov/gene/?term=2209)(48.000)[LDLR](http://www.ncbi.nlm.nih.gov/gene/?term=3949)(48.000)[C1QTNF1](http://www.ncbi.nlm.nih.gov/gene/?term=114897)(22.373)[ANG](http://www.ncbi.nlm.nih.gov/gene/?term=283)(22.373)[NR1H3](http://www.ncbi.nlm.nih.gov/gene/?term=10062)(22.373) [OSBPL8](http://www.ncbi.nlm.nih.gov/gene/?term=114882)(22.373)[ADRB2](http://www.ncbi.nlm.nih.gov/gene/?term=154)(22.373)[ITGB3](http://www.ncbi.nlm.nih.gov/gene/?term=3690)(22.373)[RXFP4](http://www.ncbi.nlm.nih.gov/gene/?term=339403)(22.373)[FOXA1](http://www.ncbi.nlm.nih.gov/gene/?term=3169)(22.373) [STX3](http://www.ncbi.nlm.nih.gov/gene/?term=6809)(22.373)[OXER1](http://www.ncbi.nlm.nih.gov/gene/?term=165140)(22.373)[PNPLA2](http://www.ncbi.nlm.nih.gov/gene/?term=57104)(22.373)[EIF2AK1](http://www.ncbi.nlm.nih.gov/gene/?term=27102)(22.373)[AGT](http://www.ncbi.nlm.nih.gov/gene/?term=183)(22.373) [MAS1](http://www.ncbi.nlm.nih.gov/gene/?term=4142)(22.373)[BMP6](http://www.ncbi.nlm.nih.gov/gene/?term=654)(22.373)[SERPINF2](http://www.ncbi.nlm.nih.gov/gene/?term=5345)(22.373)[AGTRAP](http://www.ncbi.nlm.nih.gov/gene/?term=57085)(22.373)[ITGAV](http://www.ncbi.nlm.nih.gov/gene/?term=3685)(22.373) [PLA2G1B](http://www.ncbi.nlm.nih.gov/gene/?term=5319)(22.373)[PTPN2](http://www.ncbi.nlm.nih.gov/gene/?term=5771)(22.373)[S100A8](http://www.ncbi.nlm.nih.gov/gene/?term=6279)(22.373)[ALOX5AP](http://www.ncbi.nlm.nih.gov/gene/?term=241)(22.373)[TCF3](http://www.ncbi.nlm.nih.gov/gene/?term=6929)(22.373) [NR1H2](http://www.ncbi.nlm.nih.gov/gene/?term=7376)(22.373)[NMUR2](http://www.ncbi.nlm.nih.gov/gene/?term=56923)(22.373)[ACE](http://www.ncbi.nlm.nih.gov/gene/?term=1636)(22.373)[AGTR2](http://www.ncbi.nlm.nih.gov/gene/?term=186)(22.373)[ZNF536](http://www.ncbi.nlm.nih.gov/gene/?term=9745)(22.373) [DNAJA3](http://www.ncbi.nlm.nih.gov/gene/?term=9093)(22.373)[REN](http://www.ncbi.nlm.nih.gov/gene/?term=5972)(22.373)[COL1A1](http://www.ncbi.nlm.nih.gov/gene/?term=1277)(22.373)[NR1D1](http://www.ncbi.nlm.nih.gov/gene/?term=9572)(22.373)[NOTCH1](http://www.ncbi.nlm.nih.gov/gene/?term=4851)(22.373) [RFK](http://www.ncbi.nlm.nih.gov/gene/?term=55312)(22.373)[ABCA1](http://www.ncbi.nlm.nih.gov/gene/?term=19)(22.373)[WDR77](http://www.ncbi.nlm.nih.gov/gene/?term=79084)(22.373)[ABHD5](http://www.ncbi.nlm.nih.gov/gene/?term=51099)(22.373)[RARA](http://www.ncbi.nlm.nih.gov/gene/?term=5914)(22.373) [ADIPOQ](http://www.ncbi.nlm.nih.gov/gene/?term=9370)(22.373)[PPARA](http://www.ncbi.nlm.nih.gov/gene/?term=5465)(22.373)[PAWR](http://www.ncbi.nlm.nih.gov/gene/?term=5074)(22.373)[S100A9](http://www.ncbi.nlm.nih.gov/gene/?term=6280)(22.373)[VDR](http://www.ncbi.nlm.nih.gov/gene/?term=7421)(22.373) [FABP3](http://www.ncbi.nlm.nih.gov/gene/?term=2170)(22.373)[EGFR](http://www.ncbi.nlm.nih.gov/gene/?term=1956)(22.373)[ABCG1](http://www.ncbi.nlm.nih.gov/gene/?term=9619)(22.373) |
| [Gallocatechin](http://www.megabionet.org/tcmid/ingredient/23190/) | [SEC14L3](http://www.ncbi.nlm.nih.gov/gene/?term=266629)(48.000)[SOAT1](http://www.ncbi.nlm.nih.gov/gene/?term=6646)(48.000)[MTTP](http://www.ncbi.nlm.nih.gov/gene/?term=4547)(48.000)[CNR2](http://www.ncbi.nlm.nih.gov/gene/?term=1269)(48.000)[PPP2CA](http://www.ncbi.nlm.nih.gov/gene/?term=5515)(48.000) [PRKCA](http://www.ncbi.nlm.nih.gov/gene/?term=5578)(48.000)[NR1I2](http://www.ncbi.nlm.nih.gov/gene/?term=8856)(48.000)[CNR1](http://www.ncbi.nlm.nih.gov/gene/?term=1268)(48.000)[ALOX5](http://www.ncbi.nlm.nih.gov/gene/?term=240)(48.000)[PPP2CB](http://www.ncbi.nlm.nih.gov/gene/?term=5516)(48.000) [SEC14L2](http://www.ncbi.nlm.nih.gov/gene/?term=23541)(48.000)[DGKA](http://www.ncbi.nlm.nih.gov/gene/?term=1606)(48.000)[PRKCB](http://www.ncbi.nlm.nih.gov/gene/?term=5579)(48.000)[SOAT2](http://www.ncbi.nlm.nih.gov/gene/?term=8435)(48.000)[SEC14L4](http://www.ncbi.nlm.nih.gov/gene/?term=284904)(48.000) |
| [Palmidin C](http://www.megabionet.org/tcmid/ingredient/16547/) | [ESR1](http://www.ncbi.nlm.nih.gov/gene/?term=2099)(80.882)[HSD17B1](http://www.ncbi.nlm.nih.gov/gene/?term=3292)(23.000)[TFAP2C](http://www.ncbi.nlm.nih.gov/gene/?term=7022)(22.373)[TOX3](http://www.ncbi.nlm.nih.gov/gene/?term=27324)(22.373)[TGFB1](http://www.ncbi.nlm.nih.gov/gene/?term=7040)(22.373) [MED1](http://www.ncbi.nlm.nih.gov/gene/?term=5469)(22.373)[NKX3-1](http://www.ncbi.nlm.nih.gov/gene/?term=4824)(22.373)[ESR2](http://www.ncbi.nlm.nih.gov/gene/?term=2100)(22.373)[SHH](http://www.ncbi.nlm.nih.gov/gene/?term=6469)(22.373)[LEF1](http://www.ncbi.nlm.nih.gov/gene/?term=51176)(22.373) [FGFR2](http://www.ncbi.nlm.nih.gov/gene/?term=2263)(22.373)[PGR](http://www.ncbi.nlm.nih.gov/gene/?term=5241)(22.373)[WNT4](http://www.ncbi.nlm.nih.gov/gene/?term=54361)(22.373)[AREG](http://www.ncbi.nlm.nih.gov/gene/?term=374)(22.373)[GPER1](http://www.ncbi.nlm.nih.gov/gene/?term=2852)(22.373) [WNT5A](http://www.ncbi.nlm.nih.gov/gene/?term=7474)(22.373)[SOX9](http://www.ncbi.nlm.nih.gov/gene/?term=6662)(22.373)[VDR](http://www.ncbi.nlm.nih.gov/gene/?term=7421)(22.373)[TRIM24](http://www.ncbi.nlm.nih.gov/gene/?term=8805)(22.373) |
| [Stypandrol](http://www.megabionet.org/tcmid/ingredient/31944/) | [ESR1](http://www.ncbi.nlm.nih.gov/gene/?term=2099)(80.882)[HSD17B1](http://www.ncbi.nlm.nih.gov/gene/?term=3292)(23.000)[TFAP2C](http://www.ncbi.nlm.nih.gov/gene/?term=7022)(22.373)[TOX3](http://www.ncbi.nlm.nih.gov/gene/?term=27324)(22.373)[TGFB1](http://www.ncbi.nlm.nih.gov/gene/?term=7040)(22.373) [MED1](http://www.ncbi.nlm.nih.gov/gene/?term=5469)(22.373)[NKX3-1](http://www.ncbi.nlm.nih.gov/gene/?term=4824)(22.373)[ESR2](http://www.ncbi.nlm.nih.gov/gene/?term=2100)(22.373)[SHH](http://www.ncbi.nlm.nih.gov/gene/?term=6469)(22.373)[LEF1](http://www.ncbi.nlm.nih.gov/gene/?term=51176)(22.373) [FGFR2](http://www.ncbi.nlm.nih.gov/gene/?term=2263)(22.373)[PGR](http://www.ncbi.nlm.nih.gov/gene/?term=5241)(22.373)[WNT4](http://www.ncbi.nlm.nih.gov/gene/?term=54361)(22.373)[AREG](http://www.ncbi.nlm.nih.gov/gene/?term=374)(22.373)[GPER1](http://www.ncbi.nlm.nih.gov/gene/?term=2852)(22.373) [WNT5A](http://www.ncbi.nlm.nih.gov/gene/?term=7474)(22.373)[SOX9](http://www.ncbi.nlm.nih.gov/gene/?term=6662)(22.373)[VDR](http://www.ncbi.nlm.nih.gov/gene/?term=7421)(22.373)[TRIM24](http://www.ncbi.nlm.nih.gov/gene/?term=8805)(22.373) |
| [Progesterone](http://www.megabionet.org/tcmid/ingredient/17887/) | [CYP17A1](http://www.ncbi.nlm.nih.gov/gene/?term=1586)(686.000)[ESR1](http://www.ncbi.nlm.nih.gov/gene/?term=2099)(686.000)[PGR](http://www.ncbi.nlm.nih.gov/gene/?term=5241)(686.000)[OPRK1](http://www.ncbi.nlm.nih.gov/gene/?term=4986)(686.000)[NR3C2](http://www.ncbi.nlm.nih.gov/gene/?term=4306)(686.000) [AR](http://www.ncbi.nlm.nih.gov/gene/?term=367)(122.778)[PDE7B](http://www.ncbi.nlm.nih.gov/gene/?term=27115)(80.882)[PDE5A](http://www.ncbi.nlm.nih.gov/gene/?term=8654)(80.882)[PDE9A](http://www.ncbi.nlm.nih.gov/gene/?term=5152)(80.882)[PDE4A](http://www.ncbi.nlm.nih.gov/gene/?term=5141)(80.882) [RYR1](http://www.ncbi.nlm.nih.gov/gene/?term=6261)(80.882)[PDE3A](http://www.ncbi.nlm.nih.gov/gene/?term=5139)(80.882)[ADORA2A](http://www.ncbi.nlm.nih.gov/gene/?term=135)(80.882)[ITPR1](http://www.ncbi.nlm.nih.gov/gene/?term=3708)(80.882)[PDE6A](http://www.ncbi.nlm.nih.gov/gene/?term=5145)(80.882) [PRKDC](http://www.ncbi.nlm.nih.gov/gene/?term=5591)(80.882)[PIK3CD](http://www.ncbi.nlm.nih.gov/gene/?term=5293)(80.882)[PDE3B](http://www.ncbi.nlm.nih.gov/gene/?term=5140)(80.882)[PGD](http://www.ncbi.nlm.nih.gov/gene/?term=5226)(80.882)[PIK3CA](http://www.ncbi.nlm.nih.gov/gene/?term=5290)(80.882) [ADORA2B](http://www.ncbi.nlm.nih.gov/gene/?term=136)(80.882)[PDE1A](http://www.ncbi.nlm.nih.gov/gene/?term=5136)(80.882)[PDE4D](http://www.ncbi.nlm.nih.gov/gene/?term=5144)(80.882)[PIK3CB](http://www.ncbi.nlm.nih.gov/gene/?term=5291)(80.882)[ITPR2](http://www.ncbi.nlm.nih.gov/gene/?term=3709)(80.882) [PDE1B](http://www.ncbi.nlm.nih.gov/gene/?term=5153)(80.882)[PDE7A](http://www.ncbi.nlm.nih.gov/gene/?term=5150)(80.882)[ITPR3](http://www.ncbi.nlm.nih.gov/gene/?term=3710)(80.882)[POLA2](http://www.ncbi.nlm.nih.gov/gene/?term=23649)(80.882)[PDE4C](http://www.ncbi.nlm.nih.gov/gene/?term=5143)(80.882) [PDE10A](http://www.ncbi.nlm.nih.gov/gene/?term=10846)(80.882)[PDE1C](http://www.ncbi.nlm.nih.gov/gene/?term=5137)(80.882)[ATM](http://www.ncbi.nlm.nih.gov/gene/?term=472)(80.882)[PDE6B](http://www.ncbi.nlm.nih.gov/gene/?term=5158)(80.882)[PDE4B](http://www.ncbi.nlm.nih.gov/gene/?term=5142)(80.882) [PDE2A](http://www.ncbi.nlm.nih.gov/gene/?term=5138)(80.882)[ADORA1](http://www.ncbi.nlm.nih.gov/gene/?term=134)(80.882)[CYP19A1](http://www.ncbi.nlm.nih.gov/gene/?term=1588)(80.882)[PDE8B](http://www.ncbi.nlm.nih.gov/gene/?term=8622)(80.882)[NT5E](http://www.ncbi.nlm.nih.gov/gene/?term=4907)(80.882) [HDAC2](http://www.ncbi.nlm.nih.gov/gene/?term=3066)(80.882)[PDE6C](http://www.ncbi.nlm.nih.gov/gene/?term=5146)(80.882)[PDE11A](http://www.ncbi.nlm.nih.gov/gene/?term=50940)(80.882)[PDE8A](http://www.ncbi.nlm.nih.gov/gene/?term=5151)(80.882)[RINT1](http://www.ncbi.nlm.nih.gov/gene/?term=60561)(55.444) [RIPK1](http://www.ncbi.nlm.nih.gov/gene/?term=8737)(55.444)[PIK3R1](http://www.ncbi.nlm.nih.gov/gene/?term=5295)(55.444)[WNT4](http://www.ncbi.nlm.nih.gov/gene/?term=54361)(55.444)[TACR2](http://www.ncbi.nlm.nih.gov/gene/?term=6865)(55.444)[AMPD3](http://www.ncbi.nlm.nih.gov/gene/?term=272)(55.444) [CX3CR1](http://www.ncbi.nlm.nih.gov/gene/?term=1524)(55.444)[ADA](http://www.ncbi.nlm.nih.gov/gene/?term=100)(55.444)[HAP1](http://www.ncbi.nlm.nih.gov/gene/?term=9001)(55.444)[IDNK](http://www.ncbi.nlm.nih.gov/gene/?term=414328)(55.444)[SRD5A1](http://www.ncbi.nlm.nih.gov/gene/?term=6715)(48.000) [PRLR](http://www.ncbi.nlm.nih.gov/gene/?term=5618)(48.000)[NR3C1](http://www.ncbi.nlm.nih.gov/gene/?term=2908)(48.000)[ANXA1](http://www.ncbi.nlm.nih.gov/gene/?term=301)(48.000)[CREBBP](http://www.ncbi.nlm.nih.gov/gene/?term=1387)(26.373)[MTCH2](http://www.ncbi.nlm.nih.gov/gene/?term=23788)(26.373) [MED12](http://www.ncbi.nlm.nih.gov/gene/?term=9968)(26.373)[MVP](http://www.ncbi.nlm.nih.gov/gene/?term=9961)(26.373)[RELA](http://www.ncbi.nlm.nih.gov/gene/?term=5970)(26.373)[AKT1](http://www.ncbi.nlm.nih.gov/gene/?term=207)(26.373)[PAGR1](http://www.ncbi.nlm.nih.gov/gene/?term=79447)(26.373) [PAK6](http://www.ncbi.nlm.nih.gov/gene/?term=56924)(26.373)[VAV3](http://www.ncbi.nlm.nih.gov/gene/?term=10451)(26.373)[DDX5](http://www.ncbi.nlm.nih.gov/gene/?term=1655)(26.373)[KCNMB1](http://www.ncbi.nlm.nih.gov/gene/?term=3779)(26.373)[KCNN1](http://www.ncbi.nlm.nih.gov/gene/?term=3780)(26.373) [PPARGC1A](http://www.ncbi.nlm.nih.gov/gene/?term=10891)(26.373)[PRMT2](http://www.ncbi.nlm.nih.gov/gene/?term=3275)(26.373)[XBP1](http://www.ncbi.nlm.nih.gov/gene/?term=7494)(26.373)[FHL1](http://www.ncbi.nlm.nih.gov/gene/?term=2273)(26.373)[ITGB3BP](http://www.ncbi.nlm.nih.gov/gene/?term=23421)(26.373) [PIAS1](http://www.ncbi.nlm.nih.gov/gene/?term=8554)(26.373)[NCOR2](http://www.ncbi.nlm.nih.gov/gene/?term=9612)(26.373)[NFKB1](http://www.ncbi.nlm.nih.gov/gene/?term=4790)(26.373)[ZBTB17](http://www.ncbi.nlm.nih.gov/gene/?term=7709)(26.373)[ESRRG](http://www.ncbi.nlm.nih.gov/gene/?term=2104)(26.373) [ISL1](http://www.ncbi.nlm.nih.gov/gene/?term=3670)(26.373)[USF1](http://www.ncbi.nlm.nih.gov/gene/?term=7391)(26.373)[STUB1](http://www.ncbi.nlm.nih.gov/gene/?term=10273)(26.373)[NR0B2](http://www.ncbi.nlm.nih.gov/gene/?term=8431)(26.373)[MED20](http://www.ncbi.nlm.nih.gov/gene/?term=9477)(26.373) [SMAD4](http://www.ncbi.nlm.nih.gov/gene/?term=4089)(26.373)[MAPK1](http://www.ncbi.nlm.nih.gov/gene/?term=5594)(26.373)[SMARCD3](http://www.ncbi.nlm.nih.gov/gene/?term=6604)(26.373)[NELFB](http://www.ncbi.nlm.nih.gov/gene/?term=25920)(26.373)[HMGB2](http://www.ncbi.nlm.nih.gov/gene/?term=3148)(26.373) [BRCA1](http://www.ncbi.nlm.nih.gov/gene/?term=672)(26.373)[KLF9](http://www.ncbi.nlm.nih.gov/gene/?term=687)(26.373)[CDC25B](http://www.ncbi.nlm.nih.gov/gene/?term=994)(26.373)[RABAC1](http://www.ncbi.nlm.nih.gov/gene/?term=10567)(26.373)[PRDM2](http://www.ncbi.nlm.nih.gov/gene/?term=7799)(26.373) [PTEN](http://www.ncbi.nlm.nih.gov/gene/?term=5728)(26.373)[ACTG1](http://www.ncbi.nlm.nih.gov/gene/?term=71)(26.373)[EP300](http://www.ncbi.nlm.nih.gov/gene/?term=2033)(26.373)[KMT2D](http://www.ncbi.nlm.nih.gov/gene/?term=8085)(26.373)[NSD1](http://www.ncbi.nlm.nih.gov/gene/?term=64324)(26.373) [POU4F1](http://www.ncbi.nlm.nih.gov/gene/?term=5457)(26.373)[MED7](http://www.ncbi.nlm.nih.gov/gene/?term=9443)(26.373)[CYP21A2](http://www.ncbi.nlm.nih.gov/gene/?term=1589)(26.373)[NCOR1](http://www.ncbi.nlm.nih.gov/gene/?term=9611)(26.373)[TRIM28](http://www.ncbi.nlm.nih.gov/gene/?term=10155)(26.373) [MED1](http://www.ncbi.nlm.nih.gov/gene/?term=5469)(26.373)[LCK](http://www.ncbi.nlm.nih.gov/gene/?term=3932)(26.373)[CCNC](http://www.ncbi.nlm.nih.gov/gene/?term=892)(26.373)[CDK8](http://www.ncbi.nlm.nih.gov/gene/?term=1024)(26.373)[DDX17](http://www.ncbi.nlm.nih.gov/gene/?term=10521)(26.373) [MAP3K1](http://www.ncbi.nlm.nih.gov/gene/?term=4214)(26.373)[ESR2](http://www.ncbi.nlm.nih.gov/gene/?term=2100)(26.373)[REXO4](http://www.ncbi.nlm.nih.gov/gene/?term=57109)(26.373)[RPAIN](http://www.ncbi.nlm.nih.gov/gene/?term=84268)(26.373)[CITED1](http://www.ncbi.nlm.nih.gov/gene/?term=4435)(26.373) [MAPK14](http://www.ncbi.nlm.nih.gov/gene/?term=1432)(26.373)[JDP2](http://www.ncbi.nlm.nih.gov/gene/?term=122953)(26.373)[CEBPA](http://www.ncbi.nlm.nih.gov/gene/?term=1050)(26.373)[MTA2](http://www.ncbi.nlm.nih.gov/gene/?term=9219)(26.373)[PDLIM1](http://www.ncbi.nlm.nih.gov/gene/?term=9124)(26.373) [STAT5A](http://www.ncbi.nlm.nih.gov/gene/?term=6776)(26.373)[TP53](http://www.ncbi.nlm.nih.gov/gene/?term=7157)(26.373)[SLC30A9](http://www.ncbi.nlm.nih.gov/gene/?term=10463)(26.373)[ARHGEF15](http://www.ncbi.nlm.nih.gov/gene/?term=22899)(26.373)[OPRD1](http://www.ncbi.nlm.nih.gov/gene/?term=4985)(26.373) [CDO1](http://www.ncbi.nlm.nih.gov/gene/?term=1036)(26.373)[BAZ1B](http://www.ncbi.nlm.nih.gov/gene/?term=9031)(26.373)[KDM5A](http://www.ncbi.nlm.nih.gov/gene/?term=5927)(26.373)[KCNN2](http://www.ncbi.nlm.nih.gov/gene/?term=3781)(26.373)[WIPI1](http://www.ncbi.nlm.nih.gov/gene/?term=55062)(26.373) [SMAD3](http://www.ncbi.nlm.nih.gov/gene/?term=4088)(26.373)[CCNH](http://www.ncbi.nlm.nih.gov/gene/?term=902)(26.373)[KCNH6](http://www.ncbi.nlm.nih.gov/gene/?term=81033)(26.373)[NR0B1](http://www.ncbi.nlm.nih.gov/gene/?term=190)(26.373)[FHL2](http://www.ncbi.nlm.nih.gov/gene/?term=2274)(26.373) [MED17](http://www.ncbi.nlm.nih.gov/gene/?term=9440)(26.373)[PPP5C](http://www.ncbi.nlm.nih.gov/gene/?term=5536)(26.373)[TUBB](http://www.ncbi.nlm.nih.gov/gene/?term=203068)(26.373)[PNRC2](http://www.ncbi.nlm.nih.gov/gene/?term=55629)(26.373)[GADD45G](http://www.ncbi.nlm.nih.gov/gene/?term=10912)(26.373) [STAT3](http://www.ncbi.nlm.nih.gov/gene/?term=6774)(26.373)[MPG](http://www.ncbi.nlm.nih.gov/gene/?term=4350)(26.373)[CSNK2B](http://www.ncbi.nlm.nih.gov/gene/?term=1460)(26.373)[CCND1](http://www.ncbi.nlm.nih.gov/gene/?term=595)(26.373)[TDG](http://www.ncbi.nlm.nih.gov/gene/?term=6996)(26.373) [TBP](http://www.ncbi.nlm.nih.gov/gene/?term=6908)(26.373)[CHD9](http://www.ncbi.nlm.nih.gov/gene/?term=80205)(26.373)[PELP1](http://www.ncbi.nlm.nih.gov/gene/?term=27043)(26.373)[BCAR1](http://www.ncbi.nlm.nih.gov/gene/?term=9564)(26.373)[FKBP4](http://www.ncbi.nlm.nih.gov/gene/?term=2288)(26.373) [MMS19](http://www.ncbi.nlm.nih.gov/gene/?term=64210)(26.373)[PSMB9](http://www.ncbi.nlm.nih.gov/gene/?term=5698)(26.373)[HNF4A](http://www.ncbi.nlm.nih.gov/gene/?term=3172)(26.373)[DHODH](http://www.ncbi.nlm.nih.gov/gene/?term=1723)(26.373)[UBC](http://www.ncbi.nlm.nih.gov/gene/?term=7316)(26.373) [PROX1](http://www.ncbi.nlm.nih.gov/gene/?term=5629)(26.373)[POR](http://www.ncbi.nlm.nih.gov/gene/?term=5447)(26.373)[ESRRA](http://www.ncbi.nlm.nih.gov/gene/?term=2101)(26.373)[STRN](http://www.ncbi.nlm.nih.gov/gene/?term=6801)(26.373)[SVIL](http://www.ncbi.nlm.nih.gov/gene/?term=6840)(26.373) [PTMA](http://www.ncbi.nlm.nih.gov/gene/?term=5757)(26.373)[PIK3R3](http://www.ncbi.nlm.nih.gov/gene/?term=8503)(26.373)[AHR](http://www.ncbi.nlm.nih.gov/gene/?term=196)(26.373)[PRMT1](http://www.ncbi.nlm.nih.gov/gene/?term=3276)(26.373)[NCOA7](http://www.ncbi.nlm.nih.gov/gene/?term=135112)(26.373) [BARX1](http://www.ncbi.nlm.nih.gov/gene/?term=56033)(26.373)[NOS2](http://www.ncbi.nlm.nih.gov/gene/?term=4843)(26.373)[POU2F2](http://www.ncbi.nlm.nih.gov/gene/?term=5452)(26.373)[MAPK3](http://www.ncbi.nlm.nih.gov/gene/?term=5595)(26.373)[ZBTB16](http://www.ncbi.nlm.nih.gov/gene/?term=7704)(26.373) [RBM23](http://www.ncbi.nlm.nih.gov/gene/?term=55147)(26.373)[RFX4](http://www.ncbi.nlm.nih.gov/gene/?term=5992)(26.373)[UBE2I](http://www.ncbi.nlm.nih.gov/gene/?term=7329)(26.373)[CCNT1](http://www.ncbi.nlm.nih.gov/gene/?term=904)(26.373)[GRIP1](http://www.ncbi.nlm.nih.gov/gene/?term=23426)(26.373) [SORBS1](http://www.ncbi.nlm.nih.gov/gene/?term=10580)(26.373)[GABARAP](http://www.ncbi.nlm.nih.gov/gene/?term=11337)(26.373)[PPARGC1B](http://www.ncbi.nlm.nih.gov/gene/?term=133522)(26.373)[TRAM1](http://www.ncbi.nlm.nih.gov/gene/?term=23471)(26.373)[RPS6KA1](http://www.ncbi.nlm.nih.gov/gene/?term=6195)(26.373) [PSMC5](http://www.ncbi.nlm.nih.gov/gene/?term=5705)(26.373)[RLIM](http://www.ncbi.nlm.nih.gov/gene/?term=51132)(26.373)[TSC2](http://www.ncbi.nlm.nih.gov/gene/?term=7249)(26.373)[MTA1](http://www.ncbi.nlm.nih.gov/gene/?term=9112)(26.373)[SREBF1](http://www.ncbi.nlm.nih.gov/gene/?term=6720)(26.373) [THRA](http://www.ncbi.nlm.nih.gov/gene/?term=7067)(26.373)[SAFB](http://www.ncbi.nlm.nih.gov/gene/?term=6294)(26.373)[MED21](http://www.ncbi.nlm.nih.gov/gene/?term=9412)(26.373)[NR2C2](http://www.ncbi.nlm.nih.gov/gene/?term=7182)(26.373)[DRD2](http://www.ncbi.nlm.nih.gov/gene/?term=1813)(26.373) [HMGB1](http://www.ncbi.nlm.nih.gov/gene/?term=3146)(26.373)[JUN](http://www.ncbi.nlm.nih.gov/gene/?term=3725)(26.373)[SAFB2](http://www.ncbi.nlm.nih.gov/gene/?term=9667)(26.373)[CUL4B](http://www.ncbi.nlm.nih.gov/gene/?term=8450)(26.373)[RBFOX2](http://www.ncbi.nlm.nih.gov/gene/?term=23543)(26.373) [NCOA6](http://www.ncbi.nlm.nih.gov/gene/?term=23054)(26.373)[MED16](http://www.ncbi.nlm.nih.gov/gene/?term=10025)(26.373)[SRC](http://www.ncbi.nlm.nih.gov/gene/?term=6714)(26.373)[RNF4](http://www.ncbi.nlm.nih.gov/gene/?term=6047)(26.373)[PRPF6](http://www.ncbi.nlm.nih.gov/gene/?term=24148)(26.373) [NRIP1](http://www.ncbi.nlm.nih.gov/gene/?term=8204)(26.373)[SMAD2](http://www.ncbi.nlm.nih.gov/gene/?term=4087)(26.373)[UBE3A](http://www.ncbi.nlm.nih.gov/gene/?term=7337)(26.373)[HSP90AB1](http://www.ncbi.nlm.nih.gov/gene/?term=3326)(26.373)[POLR2D](http://www.ncbi.nlm.nih.gov/gene/?term=5433)(26.373) [RBBP5](http://www.ncbi.nlm.nih.gov/gene/?term=5929)(26.373)[EIF3I](http://www.ncbi.nlm.nih.gov/gene/?term=8668)(26.373)[FKBP5](http://www.ncbi.nlm.nih.gov/gene/?term=2289)(26.373)[SP1](http://www.ncbi.nlm.nih.gov/gene/?term=6667)(26.373)[NCOA3](http://www.ncbi.nlm.nih.gov/gene/?term=8202)(26.373) [DUT](http://www.ncbi.nlm.nih.gov/gene/?term=1854)(26.373)[ARNT](http://www.ncbi.nlm.nih.gov/gene/?term=405)(26.373)[TNFRSF14](http://www.ncbi.nlm.nih.gov/gene/?term=8764)(26.373)[SP3](http://www.ncbi.nlm.nih.gov/gene/?term=6670)(26.373)[TRIP4](http://www.ncbi.nlm.nih.gov/gene/?term=9325)(26.373) [ERBB2](http://www.ncbi.nlm.nih.gov/gene/?term=2064)(26.373)[YWHAQ](http://www.ncbi.nlm.nih.gov/gene/?term=10971)(26.373)[RBM39](http://www.ncbi.nlm.nih.gov/gene/?term=9584)(26.373)[HEXIM1](http://www.ncbi.nlm.nih.gov/gene/?term=10614)(26.373)[MNAT1](http://www.ncbi.nlm.nih.gov/gene/?term=4331)(26.373) [SMARCD1](http://www.ncbi.nlm.nih.gov/gene/?term=6602)(26.373)[COPS5](http://www.ncbi.nlm.nih.gov/gene/?term=10987)(26.373)[CCNE1](http://www.ncbi.nlm.nih.gov/gene/?term=898)(26.373)[CDKN1A](http://www.ncbi.nlm.nih.gov/gene/?term=1026)(26.373)[KCNH2](http://www.ncbi.nlm.nih.gov/gene/?term=3757)(26.373) [GNAI1](http://www.ncbi.nlm.nih.gov/gene/?term=2770)(26.373)[PPID](http://www.ncbi.nlm.nih.gov/gene/?term=5481)(26.373)[MED23](http://www.ncbi.nlm.nih.gov/gene/?term=9439)(26.373)[GPRASP1](http://www.ncbi.nlm.nih.gov/gene/?term=9737)(26.373)[POU2F1](http://www.ncbi.nlm.nih.gov/gene/?term=5451)(26.373) [BATF3](http://www.ncbi.nlm.nih.gov/gene/?term=55509)(26.373)[SMARCA2](http://www.ncbi.nlm.nih.gov/gene/?term=6595)(26.373)[IGF1R](http://www.ncbi.nlm.nih.gov/gene/?term=3480)(26.373)[MDM2](http://www.ncbi.nlm.nih.gov/gene/?term=4193)(26.373)[TAP1](http://www.ncbi.nlm.nih.gov/gene/?term=6890)(26.373) [MED6](http://www.ncbi.nlm.nih.gov/gene/?term=10001)(26.373)[UBR5](http://www.ncbi.nlm.nih.gov/gene/?term=51366)(26.373)[CAV1](http://www.ncbi.nlm.nih.gov/gene/?term=857)(26.373)[ACTA1](http://www.ncbi.nlm.nih.gov/gene/?term=58)(26.373)[YWHAH](http://www.ncbi.nlm.nih.gov/gene/?term=7533)(26.373) [JUND](http://www.ncbi.nlm.nih.gov/gene/?term=3727)(26.373)[PTPN1](http://www.ncbi.nlm.nih.gov/gene/?term=5770)(26.373)[JUNB](http://www.ncbi.nlm.nih.gov/gene/?term=3726)(26.373)[SOS1](http://www.ncbi.nlm.nih.gov/gene/?term=6654)(26.373)[ASH2L](http://www.ncbi.nlm.nih.gov/gene/?term=9070)(26.373) [MTA3](http://www.ncbi.nlm.nih.gov/gene/?term=57504)(26.373)[GADD45A](http://www.ncbi.nlm.nih.gov/gene/?term=1647)(26.373)[CYB5A](http://www.ncbi.nlm.nih.gov/gene/?term=1528)(26.373)[NR2F1](http://www.ncbi.nlm.nih.gov/gene/?term=7025)(26.373)[NCOA1](http://www.ncbi.nlm.nih.gov/gene/?term=8648)(26.373) [HSPA4](http://www.ncbi.nlm.nih.gov/gene/?term=3308)(26.373)[KCNN4](http://www.ncbi.nlm.nih.gov/gene/?term=3783)(26.373)[FLII](http://www.ncbi.nlm.nih.gov/gene/?term=2314)(26.373)[DRD3](http://www.ncbi.nlm.nih.gov/gene/?term=1814)(26.373)[THRB](http://www.ncbi.nlm.nih.gov/gene/?term=7068)(26.373) [PHB2](http://www.ncbi.nlm.nih.gov/gene/?term=11331)(26.373)[TRIM59](http://www.ncbi.nlm.nih.gov/gene/?term=286827)(26.373)[GTF2B](http://www.ncbi.nlm.nih.gov/gene/?term=2959)(26.373)[TRRAP](http://www.ncbi.nlm.nih.gov/gene/?term=8295)(26.373)[NPPA](http://www.ncbi.nlm.nih.gov/gene/?term=4878)(26.373) [MED10](http://www.ncbi.nlm.nih.gov/gene/?term=84246)(26.373)[BDNF](http://www.ncbi.nlm.nih.gov/gene/?term=627)(26.373)[TADA3](http://www.ncbi.nlm.nih.gov/gene/?term=10474)(26.373)[RGS3](http://www.ncbi.nlm.nih.gov/gene/?term=5998)(26.373)[CDK2](http://www.ncbi.nlm.nih.gov/gene/?term=1017)(26.373) [FOXO3](http://www.ncbi.nlm.nih.gov/gene/?term=2309)(26.373)[NCOA2](http://www.ncbi.nlm.nih.gov/gene/?term=10499)(26.373)[KCNMB2](http://www.ncbi.nlm.nih.gov/gene/?term=10242)(26.373)[CUEDC2](http://www.ncbi.nlm.nih.gov/gene/?term=79004)(26.373)[TUBA1B](http://www.ncbi.nlm.nih.gov/gene/?term=10376)(26.373) [BAG1](http://www.ncbi.nlm.nih.gov/gene/?term=573)(26.373)[KCNMB4](http://www.ncbi.nlm.nih.gov/gene/?term=27345)(26.373)[POLR1B](http://www.ncbi.nlm.nih.gov/gene/?term=84172)(26.373)[PIAS2](http://www.ncbi.nlm.nih.gov/gene/?term=9063)(26.373)[ACSL1](http://www.ncbi.nlm.nih.gov/gene/?term=2180)(26.373) [GNA15](http://www.ncbi.nlm.nih.gov/gene/?term=2769)(26.373)[PTPN6](http://www.ncbi.nlm.nih.gov/gene/?term=5777)(26.373)[PTGES3](http://www.ncbi.nlm.nih.gov/gene/?term=10728)(26.373)[FOXO4](http://www.ncbi.nlm.nih.gov/gene/?term=4303)(26.373)[CHUK](http://www.ncbi.nlm.nih.gov/gene/?term=1147)(26.373) [NR2C1](http://www.ncbi.nlm.nih.gov/gene/?term=7181)(26.373)[NOS3](http://www.ncbi.nlm.nih.gov/gene/?term=4846)(26.373)[RARA](http://www.ncbi.nlm.nih.gov/gene/?term=5914)(26.373)[NR2F6](http://www.ncbi.nlm.nih.gov/gene/?term=2063)(26.373)[TAF10](http://www.ncbi.nlm.nih.gov/gene/?term=6881)(26.373) [CDK7](http://www.ncbi.nlm.nih.gov/gene/?term=1022)(26.373)[CTNNB1](http://www.ncbi.nlm.nih.gov/gene/?term=1499)(26.373)[LDB1](http://www.ncbi.nlm.nih.gov/gene/?term=8861)(26.373)[PPP1CC](http://www.ncbi.nlm.nih.gov/gene/?term=5501)(26.373)[SLC9A3R1](http://www.ncbi.nlm.nih.gov/gene/?term=9368)(26.373) [CRIPAK](http://www.ncbi.nlm.nih.gov/gene/?term=285464)(26.373)[TIGD7](http://www.ncbi.nlm.nih.gov/gene/?term=91151)(26.373)[XRCC6](http://www.ncbi.nlm.nih.gov/gene/?term=2547)(26.373)[CALM2](http://www.ncbi.nlm.nih.gov/gene/?term=805)(26.373)[DDX54](http://www.ncbi.nlm.nih.gov/gene/?term=79039)(26.373) [MED13](http://www.ncbi.nlm.nih.gov/gene/?term=9969)(26.373)[HSP90AA1](http://www.ncbi.nlm.nih.gov/gene/?term=3320)(26.373)[RNF14](http://www.ncbi.nlm.nih.gov/gene/?term=9604)(26.373)[SHC1](http://www.ncbi.nlm.nih.gov/gene/?term=6464)(26.373)[KCNMA1](http://www.ncbi.nlm.nih.gov/gene/?term=3778)(26.373) [CEBPB](http://www.ncbi.nlm.nih.gov/gene/?term=1051)(26.373)[GADD45B](http://www.ncbi.nlm.nih.gov/gene/?term=4616)(26.373)[ATAD2](http://www.ncbi.nlm.nih.gov/gene/?term=29028)(26.373)[TCF20](http://www.ncbi.nlm.nih.gov/gene/?term=6942)(26.373)[PIAS3](http://www.ncbi.nlm.nih.gov/gene/?term=10401)(26.373) [MED14](http://www.ncbi.nlm.nih.gov/gene/?term=9282)(26.373)[MED24](http://www.ncbi.nlm.nih.gov/gene/?term=9862)(26.373)[FOXO1](http://www.ncbi.nlm.nih.gov/gene/?term=2308)(26.373)[KAT5](http://www.ncbi.nlm.nih.gov/gene/?term=10524)(26.373)[SMARCE1](http://www.ncbi.nlm.nih.gov/gene/?term=6605)(26.373) [CCNA2](http://www.ncbi.nlm.nih.gov/gene/?term=890)(26.373)[CALM3](http://www.ncbi.nlm.nih.gov/gene/?term=808)(26.373)[SMARCA4](http://www.ncbi.nlm.nih.gov/gene/?term=6597)(26.373)[RXRA](http://www.ncbi.nlm.nih.gov/gene/?term=6256)(26.373)[CSNK2A1](http://www.ncbi.nlm.nih.gov/gene/?term=1457)(26.373) [KCNH7](http://www.ncbi.nlm.nih.gov/gene/?term=90134)(26.373)[GTF2H1](http://www.ncbi.nlm.nih.gov/gene/?term=2965)(26.373)[PAK1](http://www.ncbi.nlm.nih.gov/gene/?term=5058)(26.373)[POU4F2](http://www.ncbi.nlm.nih.gov/gene/?term=5458)(26.373)[AKAP13](http://www.ncbi.nlm.nih.gov/gene/?term=11214)(26.373) [APEX2](http://www.ncbi.nlm.nih.gov/gene/?term=27301)(26.373)[MKNK2](http://www.ncbi.nlm.nih.gov/gene/?term=2872)(26.373)[EGFR](http://www.ncbi.nlm.nih.gov/gene/?term=1956)(26.373)[KIF1A](http://www.ncbi.nlm.nih.gov/gene/?term=547)(26.373)[KCNN3](http://www.ncbi.nlm.nih.gov/gene/?term=3782)(26.373) [TRIM25](http://www.ncbi.nlm.nih.gov/gene/?term=7706)(26.373)[GABARAPL1](http://www.ncbi.nlm.nih.gov/gene/?term=23710)(26.373)[WDR5](http://www.ncbi.nlm.nih.gov/gene/?term=11091)(26.373)[SNW1](http://www.ncbi.nlm.nih.gov/gene/?term=22938)(26.373)[DNTTIP2](http://www.ncbi.nlm.nih.gov/gene/?term=30836)(26.373) [KCNMB3](http://www.ncbi.nlm.nih.gov/gene/?term=27094)(26.373)[NR1H4](http://www.ncbi.nlm.nih.gov/gene/?term=9971)(26.373)[MGMT](http://www.ncbi.nlm.nih.gov/gene/?term=4255)(26.373)[BTF3](http://www.ncbi.nlm.nih.gov/gene/?term=689)(26.373)[CALM1](http://www.ncbi.nlm.nih.gov/gene/?term=801)(26.373) [ZNF398](http://www.ncbi.nlm.nih.gov/gene/?term=57541)(26.373)[NCOA4](http://www.ncbi.nlm.nih.gov/gene/?term=8031)(26.373)[TRIM24](http://www.ncbi.nlm.nih.gov/gene/?term=8805)(26.373)[RPS6KA3](http://www.ncbi.nlm.nih.gov/gene/?term=6197)(26.373)[PRKACA](http://www.ncbi.nlm.nih.gov/gene/?term=5566)(26.373) [BCAS2](http://www.ncbi.nlm.nih.gov/gene/?term=10286)(26.373)[ATP5E](http://www.ncbi.nlm.nih.gov/gene/?term=514)(26.373)[AKT2](http://www.ncbi.nlm.nih.gov/gene/?term=208)(26.373)[DAP3](http://www.ncbi.nlm.nih.gov/gene/?term=7818)(26.373)[GRB2](http://www.ncbi.nlm.nih.gov/gene/?term=2885)(26.373) [HDAC1](http://www.ncbi.nlm.nih.gov/gene/?term=3065)(23.000)[TFAP2C](http://www.ncbi.nlm.nih.gov/gene/?term=7022)(22.373)[HMGA2](http://www.ncbi.nlm.nih.gov/gene/?term=8091)(22.373)[TNFSF11](http://www.ncbi.nlm.nih.gov/gene/?term=8600)(22.373)[NT5C1A](http://www.ncbi.nlm.nih.gov/gene/?term=84618)(22.373) [ACTN3](http://www.ncbi.nlm.nih.gov/gene/?term=89)(22.373)[NOS1AP](http://www.ncbi.nlm.nih.gov/gene/?term=9722)(22.373)[TAC1](http://www.ncbi.nlm.nih.gov/gene/?term=6863)(22.373)[PHB](http://www.ncbi.nlm.nih.gov/gene/?term=5245)(22.373)[SIRT1](http://www.ncbi.nlm.nih.gov/gene/?term=23411)(22.373) [DGKI](http://www.ncbi.nlm.nih.gov/gene/?term=9162)(22.373)[FKBP1A](http://www.ncbi.nlm.nih.gov/gene/?term=2280)(22.373)[FURIN](http://www.ncbi.nlm.nih.gov/gene/?term=5045)(22.373)[TOX3](http://www.ncbi.nlm.nih.gov/gene/?term=27324)(22.373)[UTS2](http://www.ncbi.nlm.nih.gov/gene/?term=10911)(22.373) [CTR9](http://www.ncbi.nlm.nih.gov/gene/?term=9646)(22.373)[G6PD](http://www.ncbi.nlm.nih.gov/gene/?term=2539)(22.373)[GNAT1](http://www.ncbi.nlm.nih.gov/gene/?term=2779)(22.373)[SIX1](http://www.ncbi.nlm.nih.gov/gene/?term=6495)(22.373)[QDPR](http://www.ncbi.nlm.nih.gov/gene/?term=5860)(22.373) [TGFB1](http://www.ncbi.nlm.nih.gov/gene/?term=7040)(22.373)[ATP11C](http://www.ncbi.nlm.nih.gov/gene/?term=286410)(22.373)[KHDRBS1](http://www.ncbi.nlm.nih.gov/gene/?term=10657)(22.373)[PPP3CB](http://www.ncbi.nlm.nih.gov/gene/?term=5532)(22.373)[SYT2](http://www.ncbi.nlm.nih.gov/gene/?term=127833)(22.373) [SIX4](http://www.ncbi.nlm.nih.gov/gene/?term=51804)(22.373)[SIRT2](http://www.ncbi.nlm.nih.gov/gene/?term=22933)(22.373)[SPI1](http://www.ncbi.nlm.nih.gov/gene/?term=6688)(22.373)[HOMER1](http://www.ncbi.nlm.nih.gov/gene/?term=9456)(22.373)[SHANK3](http://www.ncbi.nlm.nih.gov/gene/?term=85358)(22.373) [TAS1R1](http://www.ncbi.nlm.nih.gov/gene/?term=80835)(22.373)[XRCC4](http://www.ncbi.nlm.nih.gov/gene/?term=7518)(22.373)[NKX3-1](http://www.ncbi.nlm.nih.gov/gene/?term=4824)(22.373)[UBE2B](http://www.ncbi.nlm.nih.gov/gene/?term=7320)(22.373)[HCN4](http://www.ncbi.nlm.nih.gov/gene/?term=10021)(22.373) [EDN1](http://www.ncbi.nlm.nih.gov/gene/?term=1906)(22.373)[CACNA1A](http://www.ncbi.nlm.nih.gov/gene/?term=773)(22.373)[GNAT3](http://www.ncbi.nlm.nih.gov/gene/?term=346562)(22.373)[HELB](http://www.ncbi.nlm.nih.gov/gene/?term=92797)(22.373)[COMT](http://www.ncbi.nlm.nih.gov/gene/?term=1312)(22.373) [SLC26A6](http://www.ncbi.nlm.nih.gov/gene/?term=65010)(22.373)[ADRA2A](http://www.ncbi.nlm.nih.gov/gene/?term=150)(22.373)[YWHAE](http://www.ncbi.nlm.nih.gov/gene/?term=7531)(22.373)[CETN2](http://www.ncbi.nlm.nih.gov/gene/?term=1069)(22.373)[POLA1](http://www.ncbi.nlm.nih.gov/gene/?term=5422)(22.373) [CRP](http://www.ncbi.nlm.nih.gov/gene/?term=1401)(22.373)[CETN1](http://www.ncbi.nlm.nih.gov/gene/?term=1068)(22.373)[NUDT9](http://www.ncbi.nlm.nih.gov/gene/?term=53343)(22.373)[PIK3CG](http://www.ncbi.nlm.nih.gov/gene/?term=5294)(22.373)[BMP2](http://www.ncbi.nlm.nih.gov/gene/?term=650)(22.373) [TNF](http://www.ncbi.nlm.nih.gov/gene/?term=7124)(22.373)[NEDD4](http://www.ncbi.nlm.nih.gov/gene/?term=4734)(22.373)[AGT](http://www.ncbi.nlm.nih.gov/gene/?term=183)(22.373)[FER](http://www.ncbi.nlm.nih.gov/gene/?term=2241)(22.373)[SHH](http://www.ncbi.nlm.nih.gov/gene/?term=6469)(22.373) [SERPINB3](http://www.ncbi.nlm.nih.gov/gene/?term=6317)(22.373)[FOXP3](http://www.ncbi.nlm.nih.gov/gene/?term=50943)(22.373)[CXCL13](http://www.ncbi.nlm.nih.gov/gene/?term=10563)(22.373)[ARID1A](http://www.ncbi.nlm.nih.gov/gene/?term=8289)(22.373)[FGF10](http://www.ncbi.nlm.nih.gov/gene/?term=2255)(22.373) [KCNB1](http://www.ncbi.nlm.nih.gov/gene/?term=3745)(22.373)[LEF1](http://www.ncbi.nlm.nih.gov/gene/?term=51176)(22.373)[PTK2B](http://www.ncbi.nlm.nih.gov/gene/?term=2185)(22.373)[LIG4](http://www.ncbi.nlm.nih.gov/gene/?term=3981)(22.373)[ADRBK1](http://www.ncbi.nlm.nih.gov/gene/?term=156)(22.373) [MAOB](http://www.ncbi.nlm.nih.gov/gene/?term=4129)(22.373)[SORCS3](http://www.ncbi.nlm.nih.gov/gene/?term=22986)(22.373)[ADRB1](http://www.ncbi.nlm.nih.gov/gene/?term=153)(22.373)[ADAM8](http://www.ncbi.nlm.nih.gov/gene/?term=101)(22.373)[VTI1A](http://www.ncbi.nlm.nih.gov/gene/?term=143187)(22.373) [SLC44A4](http://www.ncbi.nlm.nih.gov/gene/?term=80736)(22.373)[ADAP2](http://www.ncbi.nlm.nih.gov/gene/?term=55803)(22.373)[LRRC8A](http://www.ncbi.nlm.nih.gov/gene/?term=56262)(22.373)[FGFR2](http://www.ncbi.nlm.nih.gov/gene/?term=2263)(22.373)[AURKA](http://www.ncbi.nlm.nih.gov/gene/?term=6790)(22.373) [MCM3](http://www.ncbi.nlm.nih.gov/gene/?term=4172)(22.373)[VCP](http://www.ncbi.nlm.nih.gov/gene/?term=7415)(22.373)[GHRL](http://www.ncbi.nlm.nih.gov/gene/?term=51738)(22.373)[BCL11B](http://www.ncbi.nlm.nih.gov/gene/?term=64919)(22.373)[BCL2](http://www.ncbi.nlm.nih.gov/gene/?term=596)(22.373) [8-Mar](http://www.ncbi.nlm.nih.gov/gene/?term=220972)(22.373)[GAS6](http://www.ncbi.nlm.nih.gov/gene/?term=2621)(22.373)[C5](http://www.ncbi.nlm.nih.gov/gene/?term=727)(22.373)[PLN](http://www.ncbi.nlm.nih.gov/gene/?term=5350)(22.373)[DNM3](http://www.ncbi.nlm.nih.gov/gene/?term=26052)(22.373) [CHRNA3](http://www.ncbi.nlm.nih.gov/gene/?term=1136)(22.373)[TCF3](http://www.ncbi.nlm.nih.gov/gene/?term=6929)(22.373)[LONP1](http://www.ncbi.nlm.nih.gov/gene/?term=9361)(22.373)[XCL1](http://www.ncbi.nlm.nih.gov/gene/?term=6375)(22.373)[HIBADH](http://www.ncbi.nlm.nih.gov/gene/?term=11112)(22.373) [STAP1](http://www.ncbi.nlm.nih.gov/gene/?term=26228)(22.373)[HRC](http://www.ncbi.nlm.nih.gov/gene/?term=3270)(22.373)[UTS2R](http://www.ncbi.nlm.nih.gov/gene/?term=2837)(22.373)[SLC6A4](http://www.ncbi.nlm.nih.gov/gene/?term=6532)(22.373)[HMGCR](http://www.ncbi.nlm.nih.gov/gene/?term=3156)(22.373) [HIPK2](http://www.ncbi.nlm.nih.gov/gene/?term=28996)(22.373)[PPP3CA](http://www.ncbi.nlm.nih.gov/gene/?term=5530)(22.373)[RAPGEF2](http://www.ncbi.nlm.nih.gov/gene/?term=9693)(22.373)[TET1](http://www.ncbi.nlm.nih.gov/gene/?term=80312)(22.373)[C2CD5](http://www.ncbi.nlm.nih.gov/gene/?term=9847)(22.373) [CSF2](http://www.ncbi.nlm.nih.gov/gene/?term=1437)(22.373)[TALDO1](http://www.ncbi.nlm.nih.gov/gene/?term=6888)(22.373)[BDKRB2](http://www.ncbi.nlm.nih.gov/gene/?term=624)(22.373)[MMP28](http://www.ncbi.nlm.nih.gov/gene/?term=79148)(22.373)[HIF1A](http://www.ncbi.nlm.nih.gov/gene/?term=3091)(22.373) [KIT](http://www.ncbi.nlm.nih.gov/gene/?term=3815)(22.373)[INS](http://www.ncbi.nlm.nih.gov/gene/?term=3630)(22.373)[MTOR](http://www.ncbi.nlm.nih.gov/gene/?term=2475)(22.373)[WNT10B](http://www.ncbi.nlm.nih.gov/gene/?term=7480)(22.373)[SLC18A3](http://www.ncbi.nlm.nih.gov/gene/?term=6572)(22.373) [ABCC4](http://www.ncbi.nlm.nih.gov/gene/?term=10257)(22.373)[BNIP3](http://www.ncbi.nlm.nih.gov/gene/?term=664)(22.373)[ZFPM1](http://www.ncbi.nlm.nih.gov/gene/?term=161882)(22.373)[GATA3](http://www.ncbi.nlm.nih.gov/gene/?term=2625)(22.373)[AREG](http://www.ncbi.nlm.nih.gov/gene/?term=374)(22.373) [CRH](http://www.ncbi.nlm.nih.gov/gene/?term=1392)(22.373)[ZFP42](http://www.ncbi.nlm.nih.gov/gene/?term=132625)(22.373)[PIK3R6](http://www.ncbi.nlm.nih.gov/gene/?term=146850)(22.373)[PF4](http://www.ncbi.nlm.nih.gov/gene/?term=5196)(22.373)[ATP1A2](http://www.ncbi.nlm.nih.gov/gene/?term=477)(22.373) [AVPR2](http://www.ncbi.nlm.nih.gov/gene/?term=554)(22.373)[CAMK2D](http://www.ncbi.nlm.nih.gov/gene/?term=817)(22.373)[ADRA1B](http://www.ncbi.nlm.nih.gov/gene/?term=147)(22.373)[IL10](http://www.ncbi.nlm.nih.gov/gene/?term=3586)(22.373)[NEFL](http://www.ncbi.nlm.nih.gov/gene/?term=4747)(22.373) [UCN2](http://www.ncbi.nlm.nih.gov/gene/?term=90226)(22.373)[ATP1A1](http://www.ncbi.nlm.nih.gov/gene/?term=476)(22.373)[RYR3](http://www.ncbi.nlm.nih.gov/gene/?term=6263)(22.373)[PPP3R1](http://www.ncbi.nlm.nih.gov/gene/?term=5534)(22.373)[SPX](http://www.ncbi.nlm.nih.gov/gene/?term=80763)(22.373) [ATP2A1](http://www.ncbi.nlm.nih.gov/gene/?term=487)(22.373)[SMO](http://www.ncbi.nlm.nih.gov/gene/?term=6608)(22.373)[RAG1](http://www.ncbi.nlm.nih.gov/gene/?term=5896)(22.373)[RAB8B](http://www.ncbi.nlm.nih.gov/gene/?term=51762)(22.373)[DKK3](http://www.ncbi.nlm.nih.gov/gene/?term=27122)(22.373) [AHCYL1](http://www.ncbi.nlm.nih.gov/gene/?term=10768)(22.373)[AQP1](http://www.ncbi.nlm.nih.gov/gene/?term=358)(22.373)[TNFAIP3](http://www.ncbi.nlm.nih.gov/gene/?term=7128)(22.373)[TAC4](http://www.ncbi.nlm.nih.gov/gene/?term=255061)(22.373)[DPPA3](http://www.ncbi.nlm.nih.gov/gene/?term=359787)(22.373) [CASQ1](http://www.ncbi.nlm.nih.gov/gene/?term=844)(22.373)[FKBP1B](http://www.ncbi.nlm.nih.gov/gene/?term=2281)(22.373)[CDC42](http://www.ncbi.nlm.nih.gov/gene/?term=998)(22.373)[IL4](http://www.ncbi.nlm.nih.gov/gene/?term=3565)(22.373)[POLB](http://www.ncbi.nlm.nih.gov/gene/?term=5423)(22.373) [IDO1](http://www.ncbi.nlm.nih.gov/gene/?term=3620)(22.373)[ATP2B4](http://www.ncbi.nlm.nih.gov/gene/?term=493)(22.373)[PGLS](http://www.ncbi.nlm.nih.gov/gene/?term=25796)(22.373)[IGF2](http://www.ncbi.nlm.nih.gov/gene/?term=3481)(22.373)[HPS4](http://www.ncbi.nlm.nih.gov/gene/?term=89781)(22.373) [TKT](http://www.ncbi.nlm.nih.gov/gene/?term=7086)(22.373)[DRD1](http://www.ncbi.nlm.nih.gov/gene/?term=1812)(22.373)[ADAP1](http://www.ncbi.nlm.nih.gov/gene/?term=11033)(22.373)[EDA](http://www.ncbi.nlm.nih.gov/gene/?term=1896)(22.373)[RYR2](http://www.ncbi.nlm.nih.gov/gene/?term=6262)(22.373) [CD34](http://www.ncbi.nlm.nih.gov/gene/?term=947)(22.373)[LTA](http://www.ncbi.nlm.nih.gov/gene/?term=4049)(22.373)[SPR](http://www.ncbi.nlm.nih.gov/gene/?term=6697)(22.373)[GPER1](http://www.ncbi.nlm.nih.gov/gene/?term=2852)(22.373)[CBFA2T3](http://www.ncbi.nlm.nih.gov/gene/?term=863)(22.373) [CALHM1](http://www.ncbi.nlm.nih.gov/gene/?term=255022)(22.373)[WNT5A](http://www.ncbi.nlm.nih.gov/gene/?term=7474)(22.373)[SELP](http://www.ncbi.nlm.nih.gov/gene/?term=6403)(22.373)[XRCC6BP1](http://www.ncbi.nlm.nih.gov/gene/?term=91419)(22.373)[PARP10](http://www.ncbi.nlm.nih.gov/gene/?term=84875)(22.373) [HPRT1](http://www.ncbi.nlm.nih.gov/gene/?term=3251)(22.373)[AMICA1](http://www.ncbi.nlm.nih.gov/gene/?term=120425)(22.373)[TAS1R3](http://www.ncbi.nlm.nih.gov/gene/?term=83756)(22.373)[NOS1](http://www.ncbi.nlm.nih.gov/gene/?term=4842)(22.373)[TREM1](http://www.ncbi.nlm.nih.gov/gene/?term=54210)(22.373) [TAL1](http://www.ncbi.nlm.nih.gov/gene/?term=6886)(22.373)[ALAD](http://www.ncbi.nlm.nih.gov/gene/?term=210)(22.373)[PAWR](http://www.ncbi.nlm.nih.gov/gene/?term=5074)(22.373)[SOX9](http://www.ncbi.nlm.nih.gov/gene/?term=6662)(22.373)[BMP5](http://www.ncbi.nlm.nih.gov/gene/?term=653)(22.373) [PDGFB](http://www.ncbi.nlm.nih.gov/gene/?term=5155)(22.373)[RTN2](http://www.ncbi.nlm.nih.gov/gene/?term=6253)(22.373)[CD63](http://www.ncbi.nlm.nih.gov/gene/?term=967)(22.373)[CAMK2G](http://www.ncbi.nlm.nih.gov/gene/?term=818)(22.373)[LAMP2](http://www.ncbi.nlm.nih.gov/gene/?term=3920)(22.373) [NEFH](http://www.ncbi.nlm.nih.gov/gene/?term=4744)(22.373)[GLYR1](http://www.ncbi.nlm.nih.gov/gene/?term=84656)(22.373)[VDR](http://www.ncbi.nlm.nih.gov/gene/?term=7421)(22.373)[GNAS](http://www.ncbi.nlm.nih.gov/gene/?term=2778)(22.373)[NCBP2](http://www.ncbi.nlm.nih.gov/gene/?term=22916)(22.373) [TPM1](http://www.ncbi.nlm.nih.gov/gene/?term=7168)(22.373)[PIK3R5](http://www.ncbi.nlm.nih.gov/gene/?term=23533)(22.373)[PCSK6](http://www.ncbi.nlm.nih.gov/gene/?term=5046)(22.373)[REST](http://www.ncbi.nlm.nih.gov/gene/?term=5978)(22.373)[CRHR2](http://www.ncbi.nlm.nih.gov/gene/?term=1395)(22.373) [HCN2](http://www.ncbi.nlm.nih.gov/gene/?term=610)(22.373)[RAG2](http://www.ncbi.nlm.nih.gov/gene/?term=5897)(22.373)[NFX1](http://www.ncbi.nlm.nih.gov/gene/?term=4799)(22.373)[TAS1R2](http://www.ncbi.nlm.nih.gov/gene/?term=80834)(22.373) |
| [Rhapontisterone](http://www.megabionet.org/tcmid/ingredient/18735/) | [SRD5A1](http://www.ncbi.nlm.nih.gov/gene/?term=6715)(48.000)[CYP17A1](http://www.ncbi.nlm.nih.gov/gene/?term=1586)(48.000)[ESR1](http://www.ncbi.nlm.nih.gov/gene/?term=2099)(48.000)[PTGER1](http://www.ncbi.nlm.nih.gov/gene/?term=5731)(48.000)[PTGER4](http://www.ncbi.nlm.nih.gov/gene/?term=5734)(48.000) [PGR](http://www.ncbi.nlm.nih.gov/gene/?term=5241)(48.000)[AR](http://www.ncbi.nlm.nih.gov/gene/?term=367)(48.000)[NR3C1](http://www.ncbi.nlm.nih.gov/gene/?term=2908)(48.000)[PTGIR](http://www.ncbi.nlm.nih.gov/gene/?term=5739)(48.000)[OPRK1](http://www.ncbi.nlm.nih.gov/gene/?term=4986)(48.000) [PTGER2](http://www.ncbi.nlm.nih.gov/gene/?term=5732)(48.000)[PTGER3](http://www.ncbi.nlm.nih.gov/gene/?term=5733)(48.000)[PTGFR](http://www.ncbi.nlm.nih.gov/gene/?term=5737)(48.000)[ANXA1](http://www.ncbi.nlm.nih.gov/gene/?term=301)(48.000)[NR3C2](http://www.ncbi.nlm.nih.gov/gene/?term=4306)(48.000) |
| [Serotonine](http://www.megabionet.org/tcmid/ingredient/25361/) | [SMPD1](http://www.ncbi.nlm.nih.gov/gene/?term=6609)(373.000)[HTR2A](http://www.ncbi.nlm.nih.gov/gene/?term=3356)(373.000)[CHRM3](http://www.ncbi.nlm.nih.gov/gene/?term=1131)(373.000)[CHRM1](http://www.ncbi.nlm.nih.gov/gene/?term=1128)(373.000)[HTR1B](http://www.ncbi.nlm.nih.gov/gene/?term=3351)(373.000) [HTR6](http://www.ncbi.nlm.nih.gov/gene/?term=3362)(373.000)[HTR2C](http://www.ncbi.nlm.nih.gov/gene/?term=3358)(373.000)[HTR1D](http://www.ncbi.nlm.nih.gov/gene/?term=3352)(373.000)[CHRM5](http://www.ncbi.nlm.nih.gov/gene/?term=1133)(373.000)[HRH4](http://www.ncbi.nlm.nih.gov/gene/?term=59340)(373.000) [HRH1](http://www.ncbi.nlm.nih.gov/gene/?term=3269)(373.000)[CALY](http://www.ncbi.nlm.nih.gov/gene/?term=50632)(373.000)[HTR1E](http://www.ncbi.nlm.nih.gov/gene/?term=3354)(373.000)[HTR3A](http://www.ncbi.nlm.nih.gov/gene/?term=3359)(373.000)[CHRM2](http://www.ncbi.nlm.nih.gov/gene/?term=1129)(373.000) [CHRM4](http://www.ncbi.nlm.nih.gov/gene/?term=1132)(373.000)[SLC6A3](http://www.ncbi.nlm.nih.gov/gene/?term=6531)(140.500)[ADRA1A](http://www.ncbi.nlm.nih.gov/gene/?term=148)(140.500)[ADRB2](http://www.ncbi.nlm.nih.gov/gene/?term=154)(140.500)[ADRA2A](http://www.ncbi.nlm.nih.gov/gene/?term=150)(140.500) [TH](http://www.ncbi.nlm.nih.gov/gene/?term=7054)(140.500)[ADRB1](http://www.ncbi.nlm.nih.gov/gene/?term=153)(140.500)[TPH1](http://www.ncbi.nlm.nih.gov/gene/?term=7166)(140.500)[SLC18A1](http://www.ncbi.nlm.nih.gov/gene/?term=6570)(140.500)[ADRA1D](http://www.ncbi.nlm.nih.gov/gene/?term=146)(140.500) [DRD2](http://www.ncbi.nlm.nih.gov/gene/?term=1813)(140.500)[PAH](http://www.ncbi.nlm.nih.gov/gene/?term=5053)(140.500)[SLC6A4](http://www.ncbi.nlm.nih.gov/gene/?term=6532)(140.500)[SLC18A2](http://www.ncbi.nlm.nih.gov/gene/?term=6571)(140.500)[DRD4](http://www.ncbi.nlm.nih.gov/gene/?term=1815)(140.500) [HTR7](http://www.ncbi.nlm.nih.gov/gene/?term=3363)(140.500)[HTR1A](http://www.ncbi.nlm.nih.gov/gene/?term=3350)(140.500)[ADRA1B](http://www.ncbi.nlm.nih.gov/gene/?term=147)(140.500)[ADRA2C](http://www.ncbi.nlm.nih.gov/gene/?term=152)(140.500)[DBH](http://www.ncbi.nlm.nih.gov/gene/?term=1621)(140.500) [DRD3](http://www.ncbi.nlm.nih.gov/gene/?term=1814)(140.500)[DRD5](http://www.ncbi.nlm.nih.gov/gene/?term=1816)(140.500)[NOS3](http://www.ncbi.nlm.nih.gov/gene/?term=4846)(140.500)[DRD1](http://www.ncbi.nlm.nih.gov/gene/?term=1812)(140.500)[ADRB3](http://www.ncbi.nlm.nih.gov/gene/?term=155)(140.500) [ADRA2B](http://www.ncbi.nlm.nih.gov/gene/?term=151)(140.500)[SLC6A2](http://www.ncbi.nlm.nih.gov/gene/?term=6530)(140.500)[CALR](http://www.ncbi.nlm.nih.gov/gene/?term=811)(114.143)[TPH2](http://www.ncbi.nlm.nih.gov/gene/?term=121278)(114.143)[CHRNA4](http://www.ncbi.nlm.nih.gov/gene/?term=1137)(114.143) [CHRNB3](http://www.ncbi.nlm.nih.gov/gene/?term=1142)(114.143)[NQO2](http://www.ncbi.nlm.nih.gov/gene/?term=4835)(114.143)[MTNR1A](http://www.ncbi.nlm.nih.gov/gene/?term=4543)(114.143)[HTR2B](http://www.ncbi.nlm.nih.gov/gene/?term=3357)(114.143)[ESR1](http://www.ncbi.nlm.nih.gov/gene/?term=2099)(114.143) [CHRNA7](http://www.ncbi.nlm.nih.gov/gene/?term=1139)(114.143)[MTNR1B](http://www.ncbi.nlm.nih.gov/gene/?term=4544)(114.143)[ASMT](http://www.ncbi.nlm.nih.gov/gene/?term=438)(114.143)[GSK3B](http://www.ncbi.nlm.nih.gov/gene/?term=2932)(114.143)[MPO](http://www.ncbi.nlm.nih.gov/gene/?term=4353)(114.143) [CHRNA10](http://www.ncbi.nlm.nih.gov/gene/?term=57053)(114.143)[CHRNA3](http://www.ncbi.nlm.nih.gov/gene/?term=1136)(114.143)[CHRNB2](http://www.ncbi.nlm.nih.gov/gene/?term=1141)(114.143)[GRIN2B](http://www.ncbi.nlm.nih.gov/gene/?term=2904)(114.143)[CHRNA2](http://www.ncbi.nlm.nih.gov/gene/?term=1135)(114.143) [CHRNA6](http://www.ncbi.nlm.nih.gov/gene/?term=8973)(114.143)[HTR1F](http://www.ncbi.nlm.nih.gov/gene/?term=3355)(114.143)[IMPA2](http://www.ncbi.nlm.nih.gov/gene/?term=3613)(114.143)[GRIA3](http://www.ncbi.nlm.nih.gov/gene/?term=2892)(114.143)[IMPA1](http://www.ncbi.nlm.nih.gov/gene/?term=3612)(114.143) [CALM2](http://www.ncbi.nlm.nih.gov/gene/?term=805)(114.143)[GSTP1](http://www.ncbi.nlm.nih.gov/gene/?term=2950)(114.143)[CHAT](http://www.ncbi.nlm.nih.gov/gene/?term=1103)(114.143)[CHRNA9](http://www.ncbi.nlm.nih.gov/gene/?term=55584)(114.143)[CHRFAM7A](http://www.ncbi.nlm.nih.gov/gene/?term=89832)(114.143) [CALM3](http://www.ncbi.nlm.nih.gov/gene/?term=808)(114.143)[CYP19A1](http://www.ncbi.nlm.nih.gov/gene/?term=1588)(114.143)[CHRNA5](http://www.ncbi.nlm.nih.gov/gene/?term=1138)(114.143)[CHRNB4](http://www.ncbi.nlm.nih.gov/gene/?term=1143)(114.143)[CALM1](http://www.ncbi.nlm.nih.gov/gene/?term=801)(114.143) [EPX](http://www.ncbi.nlm.nih.gov/gene/?term=8288)(114.143)[WARS2](http://www.ncbi.nlm.nih.gov/gene/?term=10352)(48.000)[WARS](http://www.ncbi.nlm.nih.gov/gene/?term=7453)(48.000)[SCN11A](http://www.ncbi.nlm.nih.gov/gene/?term=11280)(25.857)[SCN2B](http://www.ncbi.nlm.nih.gov/gene/?term=6327)(25.857) [PTGDR2](http://www.ncbi.nlm.nih.gov/gene/?term=11251)(25.857)[CACNB1](http://www.ncbi.nlm.nih.gov/gene/?term=782)(25.857)[CACNA2D1](http://www.ncbi.nlm.nih.gov/gene/?term=781)(25.857)[GRIA4](http://www.ncbi.nlm.nih.gov/gene/?term=2893)(25.857)[GPT2](http://www.ncbi.nlm.nih.gov/gene/?term=84706)(25.857) [CACNA1H](http://www.ncbi.nlm.nih.gov/gene/?term=8912)(25.857)[HTR4](http://www.ncbi.nlm.nih.gov/gene/?term=3360)(25.857)[HTR5A](http://www.ncbi.nlm.nih.gov/gene/?term=3361)(25.857)[ABAT](http://www.ncbi.nlm.nih.gov/gene/?term=18)(25.857)[SCN1A](http://www.ncbi.nlm.nih.gov/gene/?term=6323)(25.857) [SCN3B](http://www.ncbi.nlm.nih.gov/gene/?term=55800)(25.857)[KCNQ3](http://www.ncbi.nlm.nih.gov/gene/?term=3786)(25.857)[SLC12A2](http://www.ncbi.nlm.nih.gov/gene/?term=6558)(25.857)[GABRA2](http://www.ncbi.nlm.nih.gov/gene/?term=2555)(25.857)[MAPK1](http://www.ncbi.nlm.nih.gov/gene/?term=5594)(25.857) [CACNG2](http://www.ncbi.nlm.nih.gov/gene/?term=10369)(25.857)[GABRD](http://www.ncbi.nlm.nih.gov/gene/?term=2563)(25.857)[SLC12A7](http://www.ncbi.nlm.nih.gov/gene/?term=10723)(25.857)[SLC12A5](http://www.ncbi.nlm.nih.gov/gene/?term=57468)(25.857)[PLA2G2A](http://www.ncbi.nlm.nih.gov/gene/?term=5320)(25.857) [GABRB1](http://www.ncbi.nlm.nih.gov/gene/?term=2560)(25.857)[GRIA1](http://www.ncbi.nlm.nih.gov/gene/?term=2890)(25.857)[ADORA2A](http://www.ncbi.nlm.nih.gov/gene/?term=135)(25.857)[SCN3A](http://www.ncbi.nlm.nih.gov/gene/?term=6328)(25.857)[GLRA2](http://www.ncbi.nlm.nih.gov/gene/?term=2742)(25.857) [KCNJ3](http://www.ncbi.nlm.nih.gov/gene/?term=3760)(25.857)[GABRG3](http://www.ncbi.nlm.nih.gov/gene/?term=2567)(25.857)[NTRK1](http://www.ncbi.nlm.nih.gov/gene/?term=4914)(25.857)[OPRD1](http://www.ncbi.nlm.nih.gov/gene/?term=4985)(25.857)[SLC22A6](http://www.ncbi.nlm.nih.gov/gene/?term=9356)(25.857) [KCNH6](http://www.ncbi.nlm.nih.gov/gene/?term=81033)(25.857)[SCN7A](http://www.ncbi.nlm.nih.gov/gene/?term=6332)(25.857)[VCAM1](http://www.ncbi.nlm.nih.gov/gene/?term=7412)(25.857)[GLO1](http://www.ncbi.nlm.nih.gov/gene/?term=2739)(25.857)[GRIN3B](http://www.ncbi.nlm.nih.gov/gene/?term=116444)(25.857) [CACNA2D2](http://www.ncbi.nlm.nih.gov/gene/?term=9254)(25.857)[KCNA1](http://www.ncbi.nlm.nih.gov/gene/?term=3736)(25.857)[GRIN2A](http://www.ncbi.nlm.nih.gov/gene/?term=2903)(25.857)[PTGIS](http://www.ncbi.nlm.nih.gov/gene/?term=5740)(25.857)[SCN5A](http://www.ncbi.nlm.nih.gov/gene/?term=6331)(25.857) [KCNJ6](http://www.ncbi.nlm.nih.gov/gene/?term=3763)(25.857)[PPARG](http://www.ncbi.nlm.nih.gov/gene/?term=5468)(25.857)[SCN10A](http://www.ncbi.nlm.nih.gov/gene/?term=6336)(25.857)[CALML3](http://www.ncbi.nlm.nih.gov/gene/?term=810)(25.857)[GABRE](http://www.ncbi.nlm.nih.gov/gene/?term=2564)(25.857) [PIK3R3](http://www.ncbi.nlm.nih.gov/gene/?term=8503)(25.857)[ALDH5A1](http://www.ncbi.nlm.nih.gov/gene/?term=7915)(25.857)[MAOB](http://www.ncbi.nlm.nih.gov/gene/?term=4129)(25.857)[SCN2A](http://www.ncbi.nlm.nih.gov/gene/?term=6326)(25.857)[GABRA3](http://www.ncbi.nlm.nih.gov/gene/?term=2556)(25.857) [GABRR2](http://www.ncbi.nlm.nih.gov/gene/?term=2570)(25.857)[SLC29A1](http://www.ncbi.nlm.nih.gov/gene/?term=2030)(25.857)[CACNA1S](http://www.ncbi.nlm.nih.gov/gene/?term=779)(25.857)[ADORA2B](http://www.ncbi.nlm.nih.gov/gene/?term=136)(25.857)[PIK3R1](http://www.ncbi.nlm.nih.gov/gene/?term=5295)(25.857) [GABRG1](http://www.ncbi.nlm.nih.gov/gene/?term=2565)(25.857)[HRH3](http://www.ncbi.nlm.nih.gov/gene/?term=11255)(25.857)[GABRP](http://www.ncbi.nlm.nih.gov/gene/?term=2568)(25.857)[NR3C1](http://www.ncbi.nlm.nih.gov/gene/?term=2908)(25.857)[SIGMAR1](http://www.ncbi.nlm.nih.gov/gene/?term=10280)(25.857) [GRIA2](http://www.ncbi.nlm.nih.gov/gene/?term=2891)(25.857)[GABRA4](http://www.ncbi.nlm.nih.gov/gene/?term=2557)(25.857)[HDAC9](http://www.ncbi.nlm.nih.gov/gene/?term=9734)(25.857)[P2RY12](http://www.ncbi.nlm.nih.gov/gene/?term=64805)(25.857)[PTGIR](http://www.ncbi.nlm.nih.gov/gene/?term=5739)(25.857) [OPRK1](http://www.ncbi.nlm.nih.gov/gene/?term=4986)(25.857)[GABRB2](http://www.ncbi.nlm.nih.gov/gene/?term=2561)(25.857)[CACNA1B](http://www.ncbi.nlm.nih.gov/gene/?term=774)(25.857)[CACNA1C](http://www.ncbi.nlm.nih.gov/gene/?term=775)(25.857)[MAOA](http://www.ncbi.nlm.nih.gov/gene/?term=4128)(25.857) [SCN9A](http://www.ncbi.nlm.nih.gov/gene/?term=6335)(25.857)[CACNA1D](http://www.ncbi.nlm.nih.gov/gene/?term=776)(25.857)[GABRR1](http://www.ncbi.nlm.nih.gov/gene/?term=2569)(25.857)[PANX1](http://www.ncbi.nlm.nih.gov/gene/?term=24145)(25.857)[OPRM1](http://www.ncbi.nlm.nih.gov/gene/?term=4988)(25.857) [PTGR2](http://www.ncbi.nlm.nih.gov/gene/?term=145482)(25.857)[HTR3D](http://www.ncbi.nlm.nih.gov/gene/?term=200909)(25.857)[KCNH2](http://www.ncbi.nlm.nih.gov/gene/?term=3757)(25.857)[GRIN2C](http://www.ncbi.nlm.nih.gov/gene/?term=2905)(25.857)[GABBR2](http://www.ncbi.nlm.nih.gov/gene/?term=9568)(25.857) [SCN4A](http://www.ncbi.nlm.nih.gov/gene/?term=6329)(25.857)[SCN4B](http://www.ncbi.nlm.nih.gov/gene/?term=6330)(25.857)[GABRQ](http://www.ncbi.nlm.nih.gov/gene/?term=55879)(25.857)[GPT](http://www.ncbi.nlm.nih.gov/gene/?term=2875)(25.857)[SLC12A6](http://www.ncbi.nlm.nih.gov/gene/?term=9990)(25.857) [HRH2](http://www.ncbi.nlm.nih.gov/gene/?term=3274)(25.857)[PTGS1](http://www.ncbi.nlm.nih.gov/gene/?term=5742)(25.857)[AOC3](http://www.ncbi.nlm.nih.gov/gene/?term=8639)(25.857)[PGRMC1](http://www.ncbi.nlm.nih.gov/gene/?term=10857)(25.857)[ORM1](http://www.ncbi.nlm.nih.gov/gene/?term=5004)(25.857) [PTGS2](http://www.ncbi.nlm.nih.gov/gene/?term=5743)(25.857)[ADORA3](http://www.ncbi.nlm.nih.gov/gene/?term=140)(25.857)[GABRB3](http://www.ncbi.nlm.nih.gov/gene/?term=2562)(25.857)[KCND2](http://www.ncbi.nlm.nih.gov/gene/?term=3751)(25.857)[KCNJ5](http://www.ncbi.nlm.nih.gov/gene/?term=3762)(25.857) [GRIN3A](http://www.ncbi.nlm.nih.gov/gene/?term=116443)(25.857)[SLC22A8](http://www.ncbi.nlm.nih.gov/gene/?term=9376)(25.857)[HTR3B](http://www.ncbi.nlm.nih.gov/gene/?term=9177)(25.857)[GABRA5](http://www.ncbi.nlm.nih.gov/gene/?term=2558)(25.857)[SLC29A2](http://www.ncbi.nlm.nih.gov/gene/?term=3177)(25.857) [HTR3E](http://www.ncbi.nlm.nih.gov/gene/?term=285242)(25.857)[GABRR3](http://www.ncbi.nlm.nih.gov/gene/?term=200959)(25.857)[ACADSB](http://www.ncbi.nlm.nih.gov/gene/?term=36)(25.857)[GABRA6](http://www.ncbi.nlm.nih.gov/gene/?term=2559)(25.857)[SLC12A1](http://www.ncbi.nlm.nih.gov/gene/?term=6557)(25.857) [SCN8A](http://www.ncbi.nlm.nih.gov/gene/?term=6334)(25.857)[GABRA1](http://www.ncbi.nlm.nih.gov/gene/?term=2554)(25.857)[TSPO](http://www.ncbi.nlm.nih.gov/gene/?term=706)(25.857)[SCN1B](http://www.ncbi.nlm.nih.gov/gene/?term=6324)(25.857)[SLC22A11](http://www.ncbi.nlm.nih.gov/gene/?term=55867)(25.857) [KCNQ2](http://www.ncbi.nlm.nih.gov/gene/?term=3785)(25.857)[CACNB2](http://www.ncbi.nlm.nih.gov/gene/?term=783)(25.857)[PIK3R2](http://www.ncbi.nlm.nih.gov/gene/?term=5296)(25.857)[GABRG2](http://www.ncbi.nlm.nih.gov/gene/?term=2566)(25.857)[PPARA](http://www.ncbi.nlm.nih.gov/gene/?term=5465)(25.857) [GRIN2D](http://www.ncbi.nlm.nih.gov/gene/?term=2906)(25.857)[ADORA1](http://www.ncbi.nlm.nih.gov/gene/?term=134)(25.857)[ANXA1](http://www.ncbi.nlm.nih.gov/gene/?term=301)(25.857)[GABBR1](http://www.ncbi.nlm.nih.gov/gene/?term=2550)(25.857)[OGDH](http://www.ncbi.nlm.nih.gov/gene/?term=4967)(25.857) [KCNH7](http://www.ncbi.nlm.nih.gov/gene/?term=90134)(25.857)[TAAR1](http://www.ncbi.nlm.nih.gov/gene/?term=134864)(25.857)[NTRK2](http://www.ncbi.nlm.nih.gov/gene/?term=4915)(25.857)[ORM2](http://www.ncbi.nlm.nih.gov/gene/?term=5005)(25.857)[KCNJ9](http://www.ncbi.nlm.nih.gov/gene/?term=3765)(25.857) [HDAC2](http://www.ncbi.nlm.nih.gov/gene/?term=3066)(25.857)[SLC12A4](http://www.ncbi.nlm.nih.gov/gene/?term=6560)(25.857)[GRIK2](http://www.ncbi.nlm.nih.gov/gene/?term=2898)(25.857)[KCND3](http://www.ncbi.nlm.nih.gov/gene/?term=3752)(25.857)[HTR3C](http://www.ncbi.nlm.nih.gov/gene/?term=170572)(25.857) [GLRA1](http://www.ncbi.nlm.nih.gov/gene/?term=2741)(25.857)[GRIN1](http://www.ncbi.nlm.nih.gov/gene/?term=2902)(25.857)[CACNG1](http://www.ncbi.nlm.nih.gov/gene/?term=786)(25.857) |
| [Physcion](http://www.megabionet.org/tcmid/ingredient/17233/) | [SOAT1](http://www.ncbi.nlm.nih.gov/gene/?term=6646)(23.000)[MTTP](http://www.ncbi.nlm.nih.gov/gene/?term=4547)(23.000)[CNR2](http://www.ncbi.nlm.nih.gov/gene/?term=1269)(23.000)[CNR1](http://www.ncbi.nlm.nih.gov/gene/?term=1268)(23.000)[SOAT2](http://www.ncbi.nlm.nih.gov/gene/?term=8435)(23.000) [DRD2](http://www.ncbi.nlm.nih.gov/gene/?term=1813)(22.373)[PAWR](http://www.ncbi.nlm.nih.gov/gene/?term=5074)(22.373) |
| [3-Carboxy-4-Hydroxy-Phenoxy Glucoside](http://www.megabionet.org/tcmid/ingredient/3174/) | This compound doesn't have any potential target with score larger than 20. |
| [Cumic Acid](http://www.megabionet.org/tcmid/ingredient/30835/) | [PLAT](http://www.ncbi.nlm.nih.gov/gene/?term=5327)(80.882)[HCAR2](http://www.ncbi.nlm.nih.gov/gene/?term=338442)(80.882)[THBD](http://www.ncbi.nlm.nih.gov/gene/?term=7056)(80.882)[PPARG](http://www.ncbi.nlm.nih.gov/gene/?term=5468)(80.882)[QPRT](http://www.ncbi.nlm.nih.gov/gene/?term=23475)(80.882) [BCL2](http://www.ncbi.nlm.nih.gov/gene/?term=596)(80.882)[FABP2](http://www.ncbi.nlm.nih.gov/gene/?term=2169)(80.882)[CFTR](http://www.ncbi.nlm.nih.gov/gene/?term=1080)(80.882)[HCAR3](http://www.ncbi.nlm.nih.gov/gene/?term=8843)(80.882)[PTGS1](http://www.ncbi.nlm.nih.gov/gene/?term=5742)(80.882) [PTGS2](http://www.ncbi.nlm.nih.gov/gene/?term=5743)(80.882)[NNMT](http://www.ncbi.nlm.nih.gov/gene/?term=4837)(80.882)[APOA2](http://www.ncbi.nlm.nih.gov/gene/?term=336)(55.444)[TCAF1](http://www.ncbi.nlm.nih.gov/gene/?term=9747)(55.444)[IL1B](http://www.ncbi.nlm.nih.gov/gene/?term=3553)(55.444) [IL13](http://www.ncbi.nlm.nih.gov/gene/?term=3596)(55.444)[C1QTNF3](http://www.ncbi.nlm.nih.gov/gene/?term=114899)(55.444)[KCNE2](http://www.ncbi.nlm.nih.gov/gene/?term=9992)(55.444)[ANXA1](http://www.ncbi.nlm.nih.gov/gene/?term=301)(55.444)[SERPINB7](http://www.ncbi.nlm.nih.gov/gene/?term=8710)(55.444) [AKR1C1](http://www.ncbi.nlm.nih.gov/gene/?term=1645)(48.000)[PDXP](http://www.ncbi.nlm.nih.gov/gene/?term=57026)(22.373)[NDRG2](http://www.ncbi.nlm.nih.gov/gene/?term=57447)(22.373)[ASPDH](http://www.ncbi.nlm.nih.gov/gene/?term=554235)(22.373)[ATOH1](http://www.ncbi.nlm.nih.gov/gene/?term=474)(22.373) [SLC4A7](http://www.ncbi.nlm.nih.gov/gene/?term=9497)(22.373)[NOX1](http://www.ncbi.nlm.nih.gov/gene/?term=27035)(22.373)[NAMPT](http://www.ncbi.nlm.nih.gov/gene/?term=10135)(22.373)[RGCC](http://www.ncbi.nlm.nih.gov/gene/?term=28984)(22.373)[SIRT1](http://www.ncbi.nlm.nih.gov/gene/?term=23411)(22.373) [NGFR](http://www.ncbi.nlm.nih.gov/gene/?term=4804)(22.373)[FNDC5](http://www.ncbi.nlm.nih.gov/gene/?term=252995)(22.373)[RAB11FIP5](http://www.ncbi.nlm.nih.gov/gene/?term=26056)(22.373)[NR1H3](http://www.ncbi.nlm.nih.gov/gene/?term=10062)(22.373)[ADAMTS20](http://www.ncbi.nlm.nih.gov/gene/?term=80070)(22.373) [SIRT2](http://www.ncbi.nlm.nih.gov/gene/?term=22933)(22.373)[OSBPL8](http://www.ncbi.nlm.nih.gov/gene/?term=114882)(22.373)[CA7](http://www.ncbi.nlm.nih.gov/gene/?term=766)(22.373)[MYF5](http://www.ncbi.nlm.nih.gov/gene/?term=4617)(22.373)[ACMSD](http://www.ncbi.nlm.nih.gov/gene/?term=130013)(22.373) [AVPR1A](http://www.ncbi.nlm.nih.gov/gene/?term=552)(22.373)[DNAJC3](http://www.ncbi.nlm.nih.gov/gene/?term=5611)(22.373)[ANO9](http://www.ncbi.nlm.nih.gov/gene/?term=338440)(22.373)[SHOX2](http://www.ncbi.nlm.nih.gov/gene/?term=6474)(22.373)[KITLG](http://www.ncbi.nlm.nih.gov/gene/?term=4254)(22.373) [FASLG](http://www.ncbi.nlm.nih.gov/gene/?term=356)(22.373)[ITGB3](http://www.ncbi.nlm.nih.gov/gene/?term=3690)(22.373)[TP53](http://www.ncbi.nlm.nih.gov/gene/?term=7157)(22.373)[PRKDC](http://www.ncbi.nlm.nih.gov/gene/?term=5591)(22.373)[METRNL](http://www.ncbi.nlm.nih.gov/gene/?term=284207)(22.373) [MAPK9](http://www.ncbi.nlm.nih.gov/gene/?term=5601)(22.373)[ADAMTS9](http://www.ncbi.nlm.nih.gov/gene/?term=56999)(22.373)[SLC26A6](http://www.ncbi.nlm.nih.gov/gene/?term=65010)(22.373)[FXYD1](http://www.ncbi.nlm.nih.gov/gene/?term=5348)(22.373)[STX3](http://www.ncbi.nlm.nih.gov/gene/?term=6809)(22.373) [OXER1](http://www.ncbi.nlm.nih.gov/gene/?term=165140)(22.373)[PNPLA2](http://www.ncbi.nlm.nih.gov/gene/?term=57104)(22.373)[MYF6](http://www.ncbi.nlm.nih.gov/gene/?term=4618)(22.373)[MEF2C](http://www.ncbi.nlm.nih.gov/gene/?term=4208)(22.373)[PTGIS](http://www.ncbi.nlm.nih.gov/gene/?term=5740)(22.373) [RUNX1](http://www.ncbi.nlm.nih.gov/gene/?term=861)(22.373)[CLN3](http://www.ncbi.nlm.nih.gov/gene/?term=1201)(22.373)[PNP](http://www.ncbi.nlm.nih.gov/gene/?term=4860)(22.373)[AURKA](http://www.ncbi.nlm.nih.gov/gene/?term=6790)(22.373)[HEG1](http://www.ncbi.nlm.nih.gov/gene/?term=57493)(22.373) [HSH2D](http://www.ncbi.nlm.nih.gov/gene/?term=84941)(22.373)[ITGAV](http://www.ncbi.nlm.nih.gov/gene/?term=3685)(22.373)[SCRIB](http://www.ncbi.nlm.nih.gov/gene/?term=23513)(22.373)[MYOD1](http://www.ncbi.nlm.nih.gov/gene/?term=4654)(22.373)[MCL1](http://www.ncbi.nlm.nih.gov/gene/?term=4170)(22.373) [CNTF](http://www.ncbi.nlm.nih.gov/gene/?term=1270)(22.373)[PTPN2](http://www.ncbi.nlm.nih.gov/gene/?term=5771)(22.373)[S100A8](http://www.ncbi.nlm.nih.gov/gene/?term=6279)(22.373)[ALOX5AP](http://www.ncbi.nlm.nih.gov/gene/?term=241)(22.373)[TCF3](http://www.ncbi.nlm.nih.gov/gene/?term=6929)(22.373) [SRC](http://www.ncbi.nlm.nih.gov/gene/?term=6714)(22.373)[AVP](http://www.ncbi.nlm.nih.gov/gene/?term=551)(22.373)[BAX](http://www.ncbi.nlm.nih.gov/gene/?term=581)(22.373)[IFNG](http://www.ncbi.nlm.nih.gov/gene/?term=3458)(22.373)[SEC24B](http://www.ncbi.nlm.nih.gov/gene/?term=10427)(22.373) [SLC6A4](http://www.ncbi.nlm.nih.gov/gene/?term=6532)(22.373)[TCAF2](http://www.ncbi.nlm.nih.gov/gene/?term=285966)(22.373)[BDKRB2](http://www.ncbi.nlm.nih.gov/gene/?term=624)(22.373)[NR1H2](http://www.ncbi.nlm.nih.gov/gene/?term=7376)(22.373)[NEUROD2](http://www.ncbi.nlm.nih.gov/gene/?term=4761)(22.373) [INS](http://www.ncbi.nlm.nih.gov/gene/?term=3630)(22.373)[NGF](http://www.ncbi.nlm.nih.gov/gene/?term=4803)(22.373)[RAB11FIP3](http://www.ncbi.nlm.nih.gov/gene/?term=9727)(22.373)[ANAPC2](http://www.ncbi.nlm.nih.gov/gene/?term=29882)(22.373)[HAAO](http://www.ncbi.nlm.nih.gov/gene/?term=23498)(22.373) [WNT11](http://www.ncbi.nlm.nih.gov/gene/?term=7481)(22.373)[CDKN2A](http://www.ncbi.nlm.nih.gov/gene/?term=1029)(22.373)[ALOX15B](http://www.ncbi.nlm.nih.gov/gene/?term=247)(22.373)[CAV3](http://www.ncbi.nlm.nih.gov/gene/?term=859)(22.373)[HTT](http://www.ncbi.nlm.nih.gov/gene/?term=3064)(22.373) [SLC26A3](http://www.ncbi.nlm.nih.gov/gene/?term=1811)(22.373)[CDC20](http://www.ncbi.nlm.nih.gov/gene/?term=991)(22.373)[NUDT12](http://www.ncbi.nlm.nih.gov/gene/?term=83594)(22.373)[DNAJA3](http://www.ncbi.nlm.nih.gov/gene/?term=9093)(22.373)[ZEB2](http://www.ncbi.nlm.nih.gov/gene/?term=9839)(22.373) [IL6](http://www.ncbi.nlm.nih.gov/gene/?term=3569)(22.373)[RET](http://www.ncbi.nlm.nih.gov/gene/?term=5979)(22.373)[BCL2L1](http://www.ncbi.nlm.nih.gov/gene/?term=598)(22.373)[COL1A1](http://www.ncbi.nlm.nih.gov/gene/?term=1277)(22.373)[BDNF](http://www.ncbi.nlm.nih.gov/gene/?term=627)(22.373) [NR1D1](http://www.ncbi.nlm.nih.gov/gene/?term=9572)(22.373)[IFI6](http://www.ncbi.nlm.nih.gov/gene/?term=2537)(22.373)[ABCA1](http://www.ncbi.nlm.nih.gov/gene/?term=19)(22.373)[KMO](http://www.ncbi.nlm.nih.gov/gene/?term=8564)(22.373)[NAPRT](http://www.ncbi.nlm.nih.gov/gene/?term=93100)(22.373) [SLC34A1](http://www.ncbi.nlm.nih.gov/gene/?term=6569)(22.373)[MECOM](http://www.ncbi.nlm.nih.gov/gene/?term=2122)(22.373)[ABHD5](http://www.ncbi.nlm.nih.gov/gene/?term=51099)(22.373)[SNCA](http://www.ncbi.nlm.nih.gov/gene/?term=6622)(22.373)[ADIPOQ](http://www.ncbi.nlm.nih.gov/gene/?term=9370)(22.373) [CPLX2](http://www.ncbi.nlm.nih.gov/gene/?term=10814)(22.373)[PPARA](http://www.ncbi.nlm.nih.gov/gene/?term=5465)(22.373)[CA2](http://www.ncbi.nlm.nih.gov/gene/?term=760)(22.373)[ALOX15](http://www.ncbi.nlm.nih.gov/gene/?term=246)(22.373)[PAWR](http://www.ncbi.nlm.nih.gov/gene/?term=5074)(22.373) [MYOG](http://www.ncbi.nlm.nih.gov/gene/?term=4656)(22.373)[S100A9](http://www.ncbi.nlm.nih.gov/gene/?term=6280)(22.373)[RAB11FIP1](http://www.ncbi.nlm.nih.gov/gene/?term=80223)(22.373)[KYNU](http://www.ncbi.nlm.nih.gov/gene/?term=8942)(22.373)[FABP3](http://www.ncbi.nlm.nih.gov/gene/?term=2170)(22.373) [SEC61B](http://www.ncbi.nlm.nih.gov/gene/?term=10952)(22.373)[NR1H4](http://www.ncbi.nlm.nih.gov/gene/?term=9971)(22.373)[ABCG1](http://www.ncbi.nlm.nih.gov/gene/?term=9619)(22.373)[PRDM16](http://www.ncbi.nlm.nih.gov/gene/?term=63976)(22.373)[RAG2](http://www.ncbi.nlm.nih.gov/gene/?term=5897)(22.373) [CCM2L](http://www.ncbi.nlm.nih.gov/gene/?term=140706)(22.373) |
| [Cyanin](http://www.megabionet.org/tcmid/ingredient/4450/) | This compound doesn't have any potential target with score larger than 20. |
| [Sennoside A](http://www.megabionet.org/tcmid/ingredient/19734/) | This compound doesn't have any potential target with score larger than 20. |
| [M-Cresol](http://www.megabionet.org/tcmid/ingredient/4231/) | [GABRA2](http://www.ncbi.nlm.nih.gov/gene/?term=2555)(80.882)[GABRD](http://www.ncbi.nlm.nih.gov/gene/?term=2563)(80.882)[PDE3A](http://www.ncbi.nlm.nih.gov/gene/?term=5139)(80.882)[GABRB1](http://www.ncbi.nlm.nih.gov/gene/?term=2560)(80.882)[GABRG3](http://www.ncbi.nlm.nih.gov/gene/?term=2567)(80.882) [TNF](http://www.ncbi.nlm.nih.gov/gene/?term=7124)(80.882)[GABRE](http://www.ncbi.nlm.nih.gov/gene/?term=2564)(80.882)[SCN2A](http://www.ncbi.nlm.nih.gov/gene/?term=6326)(80.882)[GABRA3](http://www.ncbi.nlm.nih.gov/gene/?term=2556)(80.882)[GABRG1](http://www.ncbi.nlm.nih.gov/gene/?term=2565)(80.882) [GABRP](http://www.ncbi.nlm.nih.gov/gene/?term=2568)(80.882)[GABRA4](http://www.ncbi.nlm.nih.gov/gene/?term=2557)(80.882)[GABRB2](http://www.ncbi.nlm.nih.gov/gene/?term=2561)(80.882)[ACHE](http://www.ncbi.nlm.nih.gov/gene/?term=43)(80.882)[SCN4A](http://www.ncbi.nlm.nih.gov/gene/?term=6329)(80.882) [GABRQ](http://www.ncbi.nlm.nih.gov/gene/?term=55879)(80.882)[GABRB3](http://www.ncbi.nlm.nih.gov/gene/?term=2562)(80.882)[GABRA5](http://www.ncbi.nlm.nih.gov/gene/?term=2558)(80.882)[GABRA6](http://www.ncbi.nlm.nih.gov/gene/?term=2559)(80.882)[GABRA1](http://www.ncbi.nlm.nih.gov/gene/?term=2554)(80.882) [PDE4B](http://www.ncbi.nlm.nih.gov/gene/?term=5142)(80.882)[BCHE](http://www.ncbi.nlm.nih.gov/gene/?term=590)(80.882)[GABRG2](http://www.ncbi.nlm.nih.gov/gene/?term=2566)(80.882)[PDE3B](http://www.ncbi.nlm.nih.gov/gene/?term=5140)(55.444)[PDE4D](http://www.ncbi.nlm.nih.gov/gene/?term=5144)(55.444) [COLQ](http://www.ncbi.nlm.nih.gov/gene/?term=8292)(55.444)[LTA](http://www.ncbi.nlm.nih.gov/gene/?term=4049)(55.444)[RXRA](http://www.ncbi.nlm.nih.gov/gene/?term=6256)(55.444)[SCN1A](http://www.ncbi.nlm.nih.gov/gene/?term=6323)(23.000)[SCN3A](http://www.ncbi.nlm.nih.gov/gene/?term=6328)(23.000) [HMGA2](http://www.ncbi.nlm.nih.gov/gene/?term=8091)(22.373)[PDE5A](http://www.ncbi.nlm.nih.gov/gene/?term=8654)(22.373)[APOA2](http://www.ncbi.nlm.nih.gov/gene/?term=336)(22.373)[SLC5A7](http://www.ncbi.nlm.nih.gov/gene/?term=60482)(22.373)[SEMA4D](http://www.ncbi.nlm.nih.gov/gene/?term=10507)(22.373) [NRG1](http://www.ncbi.nlm.nih.gov/gene/?term=3084)(22.373)[CD74](http://www.ncbi.nlm.nih.gov/gene/?term=972)(22.373)[HPX](http://www.ncbi.nlm.nih.gov/gene/?term=3263)(22.373)[SIRT2](http://www.ncbi.nlm.nih.gov/gene/?term=22933)(22.373)[MIF](http://www.ncbi.nlm.nih.gov/gene/?term=4282)(22.373) [DMGDH](http://www.ncbi.nlm.nih.gov/gene/?term=29958)(22.373)[NKX3-1](http://www.ncbi.nlm.nih.gov/gene/?term=4824)(22.373)[SIX3](http://www.ncbi.nlm.nih.gov/gene/?term=6496)(22.373)[HCN4](http://www.ncbi.nlm.nih.gov/gene/?term=10021)(22.373)[FASLG](http://www.ncbi.nlm.nih.gov/gene/?term=356)(22.373) [LGALS3](http://www.ncbi.nlm.nih.gov/gene/?term=3958)(22.373)[KLF4](http://www.ncbi.nlm.nih.gov/gene/?term=9314)(22.373)[LILRB1](http://www.ncbi.nlm.nih.gov/gene/?term=10859)(22.373)[NCKAP1L](http://www.ncbi.nlm.nih.gov/gene/?term=3071)(22.373)[CRP](http://www.ncbi.nlm.nih.gov/gene/?term=1401)(22.373) [TLR3](http://www.ncbi.nlm.nih.gov/gene/?term=7098)(22.373)[EPO](http://www.ncbi.nlm.nih.gov/gene/?term=2056)(22.373)[MAP2K5](http://www.ncbi.nlm.nih.gov/gene/?term=5607)(22.373)[EIF2AK1](http://www.ncbi.nlm.nih.gov/gene/?term=27102)(22.373)[IL1B](http://www.ncbi.nlm.nih.gov/gene/?term=3553)(22.373) [SHH](http://www.ncbi.nlm.nih.gov/gene/?term=6469)(22.373)[RIPK1](http://www.ncbi.nlm.nih.gov/gene/?term=8737)(22.373)[FGF10](http://www.ncbi.nlm.nih.gov/gene/?term=2255)(22.373)[PRSS12](http://www.ncbi.nlm.nih.gov/gene/?term=8492)(22.373)[SLC44A4](http://www.ncbi.nlm.nih.gov/gene/?term=80736)(22.373) [ADORA2B](http://www.ncbi.nlm.nih.gov/gene/?term=136)(22.373)[ALDH7A1](http://www.ncbi.nlm.nih.gov/gene/?term=501)(22.373)[FADD](http://www.ncbi.nlm.nih.gov/gene/?term=8772)(22.373)[FGFR2](http://www.ncbi.nlm.nih.gov/gene/?term=2263)(22.373)[ANGPT1](http://www.ncbi.nlm.nih.gov/gene/?term=284)(22.373) [AURKA](http://www.ncbi.nlm.nih.gov/gene/?term=6790)(22.373)[WNT2](http://www.ncbi.nlm.nih.gov/gene/?term=7472)(22.373)[YBX3](http://www.ncbi.nlm.nih.gov/gene/?term=8531)(22.373)[IL1RN](http://www.ncbi.nlm.nih.gov/gene/?term=3557)(22.373)[PLN](http://www.ncbi.nlm.nih.gov/gene/?term=5350)(22.373) [DNM3](http://www.ncbi.nlm.nih.gov/gene/?term=26052)(22.373)[FCER2](http://www.ncbi.nlm.nih.gov/gene/?term=2208)(22.373)[WNT2B](http://www.ncbi.nlm.nih.gov/gene/?term=7482)(22.373)[IFNG](http://www.ncbi.nlm.nih.gov/gene/?term=3458)(22.373)[HRC](http://www.ncbi.nlm.nih.gov/gene/?term=3270)(22.373) [SLC6A4](http://www.ncbi.nlm.nih.gov/gene/?term=6532)(22.373)[TGFB2](http://www.ncbi.nlm.nih.gov/gene/?term=7042)(22.373)[RAPGEF2](http://www.ncbi.nlm.nih.gov/gene/?term=9693)(22.373)[TIRAP](http://www.ncbi.nlm.nih.gov/gene/?term=114609)(22.373)[ROCK2](http://www.ncbi.nlm.nih.gov/gene/?term=9475)(22.373) [ADIRF](http://www.ncbi.nlm.nih.gov/gene/?term=10974)(22.373)[BDKRB2](http://www.ncbi.nlm.nih.gov/gene/?term=624)(22.373)[ASCL1](http://www.ncbi.nlm.nih.gov/gene/?term=429)(22.373)[ENPP6](http://www.ncbi.nlm.nih.gov/gene/?term=133121)(22.373)[CAMK2D](http://www.ncbi.nlm.nih.gov/gene/?term=817)(22.373) [IL10](http://www.ncbi.nlm.nih.gov/gene/?term=3586)(22.373)[CDH8](http://www.ncbi.nlm.nih.gov/gene/?term=1006)(22.373)[NCOA1](http://www.ncbi.nlm.nih.gov/gene/?term=8648)(22.373)[IL6](http://www.ncbi.nlm.nih.gov/gene/?term=3569)(22.373)[ROCK1](http://www.ncbi.nlm.nih.gov/gene/?term=6093)(22.373) [FNTA](http://www.ncbi.nlm.nih.gov/gene/?term=2339)(22.373)[HPN](http://www.ncbi.nlm.nih.gov/gene/?term=3249)(22.373)[KMT2A](http://www.ncbi.nlm.nih.gov/gene/?term=4297)(22.373)[NOD2](http://www.ncbi.nlm.nih.gov/gene/?term=64127)(22.373)[BMP4](http://www.ncbi.nlm.nih.gov/gene/?term=652)(22.373) [AHCY](http://www.ncbi.nlm.nih.gov/gene/?term=191)(22.373)[APOA1](http://www.ncbi.nlm.nih.gov/gene/?term=335)(22.373)[ARHGEF2](http://www.ncbi.nlm.nih.gov/gene/?term=9181)(22.373)[IL4](http://www.ncbi.nlm.nih.gov/gene/?term=3565)(22.373)[AGRN](http://www.ncbi.nlm.nih.gov/gene/?term=375790)(22.373) [IKBKB](http://www.ncbi.nlm.nih.gov/gene/?term=3551)(22.373)[IDO1](http://www.ncbi.nlm.nih.gov/gene/?term=3620)(22.373)[CTNNB1](http://www.ncbi.nlm.nih.gov/gene/?term=1499)(22.373)[CHKA](http://www.ncbi.nlm.nih.gov/gene/?term=1119)(22.373)[PDE2A](http://www.ncbi.nlm.nih.gov/gene/?term=5138)(22.373) [CHDH](http://www.ncbi.nlm.nih.gov/gene/?term=55349)(22.373)[SOX9](http://www.ncbi.nlm.nih.gov/gene/?term=6662)(22.373)[CAMK2G](http://www.ncbi.nlm.nih.gov/gene/?term=818)(22.373)[FAS](http://www.ncbi.nlm.nih.gov/gene/?term=355)(22.373)[GRIN1](http://www.ncbi.nlm.nih.gov/gene/?term=2902)(22.373) [HCN2](http://www.ncbi.nlm.nih.gov/gene/?term=610)(22.373) |
| [Rhamnetin](http://www.megabionet.org/tcmid/ingredient/18664/) | This compound doesn't have any potential target with score larger than 20. |
| [Palmidin A](http://www.megabionet.org/tcmid/ingredient/16545/) | [CHRM3](http://www.ncbi.nlm.nih.gov/gene/?term=1131)(23.000)[CHRM1](http://www.ncbi.nlm.nih.gov/gene/?term=1128)(23.000)[CHRM2](http://www.ncbi.nlm.nih.gov/gene/?term=1129)(23.000)[CHRM4](http://www.ncbi.nlm.nih.gov/gene/?term=1132)(23.000) |
| [Isoeruboside B](http://www.megabionet.org/tcmid/ingredient/31350/) | [CXCR4](http://www.ncbi.nlm.nih.gov/gene/?term=7852)(48.000) |
| [Stylopine](http://www.megabionet.org/tcmid/ingredient/20400/) | [CHRM3](http://www.ncbi.nlm.nih.gov/gene/?term=1131)(48.000)[DRD2](http://www.ncbi.nlm.nih.gov/gene/?term=1813)(48.000)[ACHE](http://www.ncbi.nlm.nih.gov/gene/?term=43)(48.000)[SLC18A2](http://www.ncbi.nlm.nih.gov/gene/?term=6571)(48.000)[CHRNA2](http://www.ncbi.nlm.nih.gov/gene/?term=1135)(48.000) [HTR3A](http://www.ncbi.nlm.nih.gov/gene/?term=3359)(48.000)[BCHE](http://www.ncbi.nlm.nih.gov/gene/?term=590)(48.000)[CHRM2](http://www.ncbi.nlm.nih.gov/gene/?term=1129)(48.000)[HTR2A](http://www.ncbi.nlm.nih.gov/gene/?term=3356)(23.000)[CHRNA4](http://www.ncbi.nlm.nih.gov/gene/?term=1137)(23.000) [CHRNB3](http://www.ncbi.nlm.nih.gov/gene/?term=1142)(23.000)[SULT1E1](http://www.ncbi.nlm.nih.gov/gene/?term=6783)(23.000)[CHRM1](http://www.ncbi.nlm.nih.gov/gene/?term=1128)(23.000)[CHRNA7](http://www.ncbi.nlm.nih.gov/gene/?term=1139)(23.000)[HTR2C](http://www.ncbi.nlm.nih.gov/gene/?term=3358)(23.000) [CHRNA10](http://www.ncbi.nlm.nih.gov/gene/?term=57053)(23.000)[CHRNA3](http://www.ncbi.nlm.nih.gov/gene/?term=1136)(23.000)[CHRNB2](http://www.ncbi.nlm.nih.gov/gene/?term=1141)(23.000)[HRH1](http://www.ncbi.nlm.nih.gov/gene/?term=3269)(23.000)[CHRNA6](http://www.ncbi.nlm.nih.gov/gene/?term=8973)(23.000) [CHAT](http://www.ncbi.nlm.nih.gov/gene/?term=1103)(23.000)[CHRNA9](http://www.ncbi.nlm.nih.gov/gene/?term=55584)(23.000)[CHRFAM7A](http://www.ncbi.nlm.nih.gov/gene/?term=89832)(23.000)[CYP19A1](http://www.ncbi.nlm.nih.gov/gene/?term=1588)(23.000)[CHRNA5](http://www.ncbi.nlm.nih.gov/gene/?term=1138)(23.000) [CHRNB4](http://www.ncbi.nlm.nih.gov/gene/?term=1143)(23.000) |
| [Meldenin](http://www.megabionet.org/tcmid/ingredient/13654/) | [PLAT](http://www.ncbi.nlm.nih.gov/gene/?term=5327)(23.000)[PDE4A](http://www.ncbi.nlm.nih.gov/gene/?term=5141)(23.000)[PTGER1](http://www.ncbi.nlm.nih.gov/gene/?term=5731)(23.000)[PDE4D](http://www.ncbi.nlm.nih.gov/gene/?term=5144)(23.000)[NR3C1](http://www.ncbi.nlm.nih.gov/gene/?term=2908)(23.000) [PTGIR](http://www.ncbi.nlm.nih.gov/gene/?term=5739)(23.000)[PDE4C](http://www.ncbi.nlm.nih.gov/gene/?term=5143)(23.000)[PDE4B](http://www.ncbi.nlm.nih.gov/gene/?term=5142)(23.000) |
| [Strictinin](http://www.megabionet.org/tcmid/ingredient/20373/) | This compound doesn't have any potential target with score larger than 20. |
| [Disenecionyl Cis-Khellactone](http://www.megabionet.org/tcmid/ingredient/25817/) | This compound doesn't have any potential target with score larger than 20. |
| [Canadine](http://www.megabionet.org/tcmid/ingredient/3057/) | [CHRM3](http://www.ncbi.nlm.nih.gov/gene/?term=1131)(48.000)[DRD2](http://www.ncbi.nlm.nih.gov/gene/?term=1813)(48.000)[ACHE](http://www.ncbi.nlm.nih.gov/gene/?term=43)(48.000)[SLC18A2](http://www.ncbi.nlm.nih.gov/gene/?term=6571)(48.000)[CHRNA2](http://www.ncbi.nlm.nih.gov/gene/?term=1135)(48.000) [HTR3A](http://www.ncbi.nlm.nih.gov/gene/?term=3359)(48.000)[BCHE](http://www.ncbi.nlm.nih.gov/gene/?term=590)(48.000)[CHRM2](http://www.ncbi.nlm.nih.gov/gene/?term=1129)(48.000)[CYBA](http://www.ncbi.nlm.nih.gov/gene/?term=1535)(23.000)[CHRNA4](http://www.ncbi.nlm.nih.gov/gene/?term=1137)(23.000) [RAC2](http://www.ncbi.nlm.nih.gov/gene/?term=5880)(23.000)[RAC1](http://www.ncbi.nlm.nih.gov/gene/?term=5879)(23.000)[OPRD1](http://www.ncbi.nlm.nih.gov/gene/?term=4985)(23.000)[NCF4](http://www.ncbi.nlm.nih.gov/gene/?term=4689)(23.000)[CHRNA7](http://www.ncbi.nlm.nih.gov/gene/?term=1139)(23.000) [CYBB](http://www.ncbi.nlm.nih.gov/gene/?term=1536)(23.000)[NCF1](http://www.ncbi.nlm.nih.gov/gene/?term=653361)(23.000)[SIGMAR1](http://www.ncbi.nlm.nih.gov/gene/?term=10280)(23.000)[NCF2](http://www.ncbi.nlm.nih.gov/gene/?term=4688)(23.000)[OPRK1](http://www.ncbi.nlm.nih.gov/gene/?term=4986)(23.000) [CHRNA3](http://www.ncbi.nlm.nih.gov/gene/?term=1136)(23.000)[RAC3](http://www.ncbi.nlm.nih.gov/gene/?term=5881)(23.000)[CHRNB2](http://www.ncbi.nlm.nih.gov/gene/?term=1141)(23.000)[SLC6A4](http://www.ncbi.nlm.nih.gov/gene/?term=6532)(23.000)[OPRM1](http://www.ncbi.nlm.nih.gov/gene/?term=4988)(23.000) [PGRMC1](http://www.ncbi.nlm.nih.gov/gene/?term=10857)(23.000)[GRIN3A](http://www.ncbi.nlm.nih.gov/gene/?term=116443)(23.000)[CHRFAM7A](http://www.ncbi.nlm.nih.gov/gene/?term=89832)(23.000)[CHRNB4](http://www.ncbi.nlm.nih.gov/gene/?term=1143)(23.000)[SLC6A2](http://www.ncbi.nlm.nih.gov/gene/?term=6530)(23.000) [TLR4](http://www.ncbi.nlm.nih.gov/gene/?term=7099)(22.373)[ELANE](http://www.ncbi.nlm.nih.gov/gene/?term=1991)(22.373)[CTSG](http://www.ncbi.nlm.nih.gov/gene/?term=1511)(22.373)[SLC18A1](http://www.ncbi.nlm.nih.gov/gene/?term=6570)(22.373)[CEND1](http://www.ncbi.nlm.nih.gov/gene/?term=51286)(22.373) [SLC22A1](http://www.ncbi.nlm.nih.gov/gene/?term=6580)(22.373)[ADRA1B](http://www.ncbi.nlm.nih.gov/gene/?term=147)(22.373)[WLS](http://www.ncbi.nlm.nih.gov/gene/?term=79971)(22.373)[CHRNA9](http://www.ncbi.nlm.nih.gov/gene/?term=55584)(22.373) |
| [Cinnamic Acid](http://www.megabionet.org/tcmid/ingredient/23095/) | [RXRB](http://www.ncbi.nlm.nih.gov/gene/?term=6257)(80.882)[PPARG](http://www.ncbi.nlm.nih.gov/gene/?term=5468)(80.882)[RXRG](http://www.ncbi.nlm.nih.gov/gene/?term=6258)(80.882)[AGTR1](http://www.ncbi.nlm.nih.gov/gene/?term=185)(80.882)[RXRA](http://www.ncbi.nlm.nih.gov/gene/?term=6256)(80.882) [APOA2](http://www.ncbi.nlm.nih.gov/gene/?term=336)(55.444)[IL1B](http://www.ncbi.nlm.nih.gov/gene/?term=3553)(55.444)[TNF](http://www.ncbi.nlm.nih.gov/gene/?term=7124)(55.444)[IL13](http://www.ncbi.nlm.nih.gov/gene/?term=3596)(55.444)[ACTN2](http://www.ncbi.nlm.nih.gov/gene/?term=88)(55.444) [C1QTNF1](http://www.ncbi.nlm.nih.gov/gene/?term=114897)(22.373)[ANG](http://www.ncbi.nlm.nih.gov/gene/?term=283)(22.373)[NR1H3](http://www.ncbi.nlm.nih.gov/gene/?term=10062)(22.373)[OSBPL8](http://www.ncbi.nlm.nih.gov/gene/?term=114882)(22.373)[ADRB2](http://www.ncbi.nlm.nih.gov/gene/?term=154)(22.373) [ITGB3](http://www.ncbi.nlm.nih.gov/gene/?term=3690)(22.373)[RXFP4](http://www.ncbi.nlm.nih.gov/gene/?term=339403)(22.373)[FOXA1](http://www.ncbi.nlm.nih.gov/gene/?term=3169)(22.373)[STX3](http://www.ncbi.nlm.nih.gov/gene/?term=6809)(22.373)[OXER1](http://www.ncbi.nlm.nih.gov/gene/?term=165140)(22.373) [PNPLA2](http://www.ncbi.nlm.nih.gov/gene/?term=57104)(22.373)[EIF2AK1](http://www.ncbi.nlm.nih.gov/gene/?term=27102)(22.373)[AGT](http://www.ncbi.nlm.nih.gov/gene/?term=183)(22.373)[MAS1](http://www.ncbi.nlm.nih.gov/gene/?term=4142)(22.373)[BMP6](http://www.ncbi.nlm.nih.gov/gene/?term=654)(22.373) [SERPINF2](http://www.ncbi.nlm.nih.gov/gene/?term=5345)(22.373)[AGTRAP](http://www.ncbi.nlm.nih.gov/gene/?term=57085)(22.373)[ITGAV](http://www.ncbi.nlm.nih.gov/gene/?term=3685)(22.373)[PLA2G1B](http://www.ncbi.nlm.nih.gov/gene/?term=5319)(22.373)[PTPN2](http://www.ncbi.nlm.nih.gov/gene/?term=5771)(22.373) [S100A8](http://www.ncbi.nlm.nih.gov/gene/?term=6279)(22.373)[ALOX5AP](http://www.ncbi.nlm.nih.gov/gene/?term=241)(22.373)[TCF3](http://www.ncbi.nlm.nih.gov/gene/?term=6929)(22.373)[NR1H2](http://www.ncbi.nlm.nih.gov/gene/?term=7376)(22.373)[NMUR2](http://www.ncbi.nlm.nih.gov/gene/?term=56923)(22.373) [ACE](http://www.ncbi.nlm.nih.gov/gene/?term=1636)(22.373)[AGTR2](http://www.ncbi.nlm.nih.gov/gene/?term=186)(22.373)[ZNF536](http://www.ncbi.nlm.nih.gov/gene/?term=9745)(22.373)[DNAJA3](http://www.ncbi.nlm.nih.gov/gene/?term=9093)(22.373)[REN](http://www.ncbi.nlm.nih.gov/gene/?term=5972)(22.373) [COL1A1](http://www.ncbi.nlm.nih.gov/gene/?term=1277)(22.373)[NR1D1](http://www.ncbi.nlm.nih.gov/gene/?term=9572)(22.373)[NOTCH1](http://www.ncbi.nlm.nih.gov/gene/?term=4851)(22.373)[RFK](http://www.ncbi.nlm.nih.gov/gene/?term=55312)(22.373)[ABCA1](http://www.ncbi.nlm.nih.gov/gene/?term=19)(22.373) [WDR77](http://www.ncbi.nlm.nih.gov/gene/?term=79084)(22.373)[ABHD5](http://www.ncbi.nlm.nih.gov/gene/?term=51099)(22.373)[RARA](http://www.ncbi.nlm.nih.gov/gene/?term=5914)(22.373)[ADIPOQ](http://www.ncbi.nlm.nih.gov/gene/?term=9370)(22.373)[PPARA](http://www.ncbi.nlm.nih.gov/gene/?term=5465)(22.373) [PAWR](http://www.ncbi.nlm.nih.gov/gene/?term=5074)(22.373)[S100A9](http://www.ncbi.nlm.nih.gov/gene/?term=6280)(22.373)[VDR](http://www.ncbi.nlm.nih.gov/gene/?term=7421)(22.373)[FABP3](http://www.ncbi.nlm.nih.gov/gene/?term=2170)(22.373)[EGFR](http://www.ncbi.nlm.nih.gov/gene/?term=1956)(22.373) [ABCG1](http://www.ncbi.nlm.nih.gov/gene/?term=9619)(22.373) |
| [Tetrahydropalmatine](http://www.megabionet.org/tcmid/ingredient/21061/) | [ACHE](http://www.ncbi.nlm.nih.gov/gene/?term=43)(122.778)[CHRNA2](http://www.ncbi.nlm.nih.gov/gene/?term=1135)(122.778)[HTR3A](http://www.ncbi.nlm.nih.gov/gene/?term=3359)(122.778)[CHRM2](http://www.ncbi.nlm.nih.gov/gene/?term=1129)(122.778)[CYBA](http://www.ncbi.nlm.nih.gov/gene/?term=1535)(80.882) [CHRNA4](http://www.ncbi.nlm.nih.gov/gene/?term=1137)(80.882)[RAC2](http://www.ncbi.nlm.nih.gov/gene/?term=5880)(80.882)[RAC1](http://www.ncbi.nlm.nih.gov/gene/?term=5879)(80.882)[OPRD1](http://www.ncbi.nlm.nih.gov/gene/?term=4985)(80.882)[NCF4](http://www.ncbi.nlm.nih.gov/gene/?term=4689)(80.882) [CHRNA7](http://www.ncbi.nlm.nih.gov/gene/?term=1139)(80.882)[CYBB](http://www.ncbi.nlm.nih.gov/gene/?term=1536)(80.882)[NCF1](http://www.ncbi.nlm.nih.gov/gene/?term=653361)(80.882)[SIGMAR1](http://www.ncbi.nlm.nih.gov/gene/?term=10280)(80.882)[NCF2](http://www.ncbi.nlm.nih.gov/gene/?term=4688)(80.882) [OPRK1](http://www.ncbi.nlm.nih.gov/gene/?term=4986)(80.882)[CHRNA3](http://www.ncbi.nlm.nih.gov/gene/?term=1136)(80.882)[RAC3](http://www.ncbi.nlm.nih.gov/gene/?term=5881)(80.882)[CHRNB2](http://www.ncbi.nlm.nih.gov/gene/?term=1141)(80.882)[SLC6A4](http://www.ncbi.nlm.nih.gov/gene/?term=6532)(80.882) [OPRM1](http://www.ncbi.nlm.nih.gov/gene/?term=4988)(80.882)[PGRMC1](http://www.ncbi.nlm.nih.gov/gene/?term=10857)(80.882)[GRIN3A](http://www.ncbi.nlm.nih.gov/gene/?term=116443)(80.882)[CHRFAM7A](http://www.ncbi.nlm.nih.gov/gene/?term=89832)(80.882)[CHRNB4](http://www.ncbi.nlm.nih.gov/gene/?term=1143)(80.882) [SLC6A2](http://www.ncbi.nlm.nih.gov/gene/?term=6530)(80.882)[TLR4](http://www.ncbi.nlm.nih.gov/gene/?term=7099)(55.444)[ELANE](http://www.ncbi.nlm.nih.gov/gene/?term=1991)(55.444)[CTSG](http://www.ncbi.nlm.nih.gov/gene/?term=1511)(55.444)[SLC18A1](http://www.ncbi.nlm.nih.gov/gene/?term=6570)(55.444) [CEND1](http://www.ncbi.nlm.nih.gov/gene/?term=51286)(55.444)[SLC22A1](http://www.ncbi.nlm.nih.gov/gene/?term=6580)(55.444)[ADRA1B](http://www.ncbi.nlm.nih.gov/gene/?term=147)(55.444)[WLS](http://www.ncbi.nlm.nih.gov/gene/?term=79971)(55.444)[CHRNA9](http://www.ncbi.nlm.nih.gov/gene/?term=55584)(55.444) [CHRM3](http://www.ncbi.nlm.nih.gov/gene/?term=1131)(48.000)[DRD2](http://www.ncbi.nlm.nih.gov/gene/?term=1813)(48.000)[SLC18A2](http://www.ncbi.nlm.nih.gov/gene/?term=6571)(48.000)[BCHE](http://www.ncbi.nlm.nih.gov/gene/?term=590)(48.000)[ADRA1A](http://www.ncbi.nlm.nih.gov/gene/?term=148)(23.000) [HTR2A](http://www.ncbi.nlm.nih.gov/gene/?term=3356)(23.000)[CHRM1](http://www.ncbi.nlm.nih.gov/gene/?term=1128)(23.000)[HTR2B](http://www.ncbi.nlm.nih.gov/gene/?term=3357)(23.000)[ADRA2A](http://www.ncbi.nlm.nih.gov/gene/?term=150)(23.000)[HTR6](http://www.ncbi.nlm.nih.gov/gene/?term=3362)(23.000) [HTR2C](http://www.ncbi.nlm.nih.gov/gene/?term=3358)(23.000)[ADRA1D](http://www.ncbi.nlm.nih.gov/gene/?term=146)(23.000)[CHRM5](http://www.ncbi.nlm.nih.gov/gene/?term=1133)(23.000)[HRH4](http://www.ncbi.nlm.nih.gov/gene/?term=59340)(23.000)[HRH1](http://www.ncbi.nlm.nih.gov/gene/?term=3269)(23.000) [KCNH2](http://www.ncbi.nlm.nih.gov/gene/?term=3757)(23.000)[HRH2](http://www.ncbi.nlm.nih.gov/gene/?term=3274)(23.000)[HTR1A](http://www.ncbi.nlm.nih.gov/gene/?term=3350)(23.000)[ADRA2C](http://www.ncbi.nlm.nih.gov/gene/?term=152)(23.000)[CHRM4](http://www.ncbi.nlm.nih.gov/gene/?term=1132)(23.000) [ADRA2B](http://www.ncbi.nlm.nih.gov/gene/?term=151)(23.000)[ACTN3](http://www.ncbi.nlm.nih.gov/gene/?term=89)(22.373)[GPM6B](http://www.ncbi.nlm.nih.gov/gene/?term=2824)(22.373)[HMOX1](http://www.ncbi.nlm.nih.gov/gene/?term=3162)(22.373)[TAC1](http://www.ncbi.nlm.nih.gov/gene/?term=6863)(22.373) [MC2R](http://www.ncbi.nlm.nih.gov/gene/?term=4158)(22.373)[SLITRK6](http://www.ncbi.nlm.nih.gov/gene/?term=84189)(22.373)[UTS2](http://www.ncbi.nlm.nih.gov/gene/?term=10911)(22.373)[NAV2](http://www.ncbi.nlm.nih.gov/gene/?term=89797)(22.373)[PDE3A](http://www.ncbi.nlm.nih.gov/gene/?term=5139)(22.373) [SCT](http://www.ncbi.nlm.nih.gov/gene/?term=6343)(22.373)[TUSC2](http://www.ncbi.nlm.nih.gov/gene/?term=11334)(22.373)[RHOA](http://www.ncbi.nlm.nih.gov/gene/?term=387)(22.373)[AVPR1A](http://www.ncbi.nlm.nih.gov/gene/?term=552)(22.373)[ADORA2A](http://www.ncbi.nlm.nih.gov/gene/?term=135)(22.373) [HCN4](http://www.ncbi.nlm.nih.gov/gene/?term=10021)(22.373)[PHKG2](http://www.ncbi.nlm.nih.gov/gene/?term=5261)(22.373)[OXT](http://www.ncbi.nlm.nih.gov/gene/?term=5020)(22.373)[CACNA1A](http://www.ncbi.nlm.nih.gov/gene/?term=773)(22.373)[CCL5](http://www.ncbi.nlm.nih.gov/gene/?term=6352)(22.373) [APLP1](http://www.ncbi.nlm.nih.gov/gene/?term=333)(22.373)[LAMP1](http://www.ncbi.nlm.nih.gov/gene/?term=3916)(22.373)[PPP1R9B](http://www.ncbi.nlm.nih.gov/gene/?term=84687)(22.373)[YWHAE](http://www.ncbi.nlm.nih.gov/gene/?term=7531)(22.373)[SUMO1](http://www.ncbi.nlm.nih.gov/gene/?term=7341)(22.373) [HTR1B](http://www.ncbi.nlm.nih.gov/gene/?term=3351)(22.373)[CNR2](http://www.ncbi.nlm.nih.gov/gene/?term=1269)(22.373)[NOXA1](http://www.ncbi.nlm.nih.gov/gene/?term=10811)(22.373)[MC1R](http://www.ncbi.nlm.nih.gov/gene/?term=4157)(22.373)[MC4R](http://www.ncbi.nlm.nih.gov/gene/?term=4160)(22.373) [IL1B](http://www.ncbi.nlm.nih.gov/gene/?term=3553)(22.373)[AGT](http://www.ncbi.nlm.nih.gov/gene/?term=183)(22.373)[CRHBP](http://www.ncbi.nlm.nih.gov/gene/?term=1393)(22.373)[F2R](http://www.ncbi.nlm.nih.gov/gene/?term=2149)(22.373)[MTNR1B](http://www.ncbi.nlm.nih.gov/gene/?term=4544)(22.373) [FGF10](http://www.ncbi.nlm.nih.gov/gene/?term=2255)(22.373)[EGR1](http://www.ncbi.nlm.nih.gov/gene/?term=1958)(22.373)[NOXO1](http://www.ncbi.nlm.nih.gov/gene/?term=124056)(22.373)[ADAM8](http://www.ncbi.nlm.nih.gov/gene/?term=101)(22.373)[SLC44A4](http://www.ncbi.nlm.nih.gov/gene/?term=80736)(22.373) [FLOT1](http://www.ncbi.nlm.nih.gov/gene/?term=10211)(22.373)[NPY5R](http://www.ncbi.nlm.nih.gov/gene/?term=4889)(22.373)[VCP](http://www.ncbi.nlm.nih.gov/gene/?term=7415)(22.373)[GHRL](http://www.ncbi.nlm.nih.gov/gene/?term=51738)(22.373)[TBR1](http://www.ncbi.nlm.nih.gov/gene/?term=10716)(22.373) [PTPRO](http://www.ncbi.nlm.nih.gov/gene/?term=5800)(22.373)[OXTR](http://www.ncbi.nlm.nih.gov/gene/?term=5021)(22.373)[PAX2](http://www.ncbi.nlm.nih.gov/gene/?term=5076)(22.373)[CACNA1C](http://www.ncbi.nlm.nih.gov/gene/?term=775)(22.373)[F2RL1](http://www.ncbi.nlm.nih.gov/gene/?term=2150)(22.373) [UTS2R](http://www.ncbi.nlm.nih.gov/gene/?term=2837)(22.373)[ACVR2A](http://www.ncbi.nlm.nih.gov/gene/?term=92)(22.373)[RAPGEF2](http://www.ncbi.nlm.nih.gov/gene/?term=9693)(22.373)[TIRAP](http://www.ncbi.nlm.nih.gov/gene/?term=114609)(22.373)[AIF1](http://www.ncbi.nlm.nih.gov/gene/?term=199)(22.373) [GHRH](http://www.ncbi.nlm.nih.gov/gene/?term=2691)(22.373)[HCRT](http://www.ncbi.nlm.nih.gov/gene/?term=3060)(22.373)[RAB3B](http://www.ncbi.nlm.nih.gov/gene/?term=5865)(22.373)[CNR1](http://www.ncbi.nlm.nih.gov/gene/?term=1268)(22.373)[HIF1A](http://www.ncbi.nlm.nih.gov/gene/?term=3091)(22.373) [INS](http://www.ncbi.nlm.nih.gov/gene/?term=3630)(22.373)[ACE](http://www.ncbi.nlm.nih.gov/gene/?term=1636)(22.373)[SLC18A3](http://www.ncbi.nlm.nih.gov/gene/?term=6572)(22.373)[BNIP3](http://www.ncbi.nlm.nih.gov/gene/?term=664)(22.373)[DRD4](http://www.ncbi.nlm.nih.gov/gene/?term=1815)(22.373) [CRH](http://www.ncbi.nlm.nih.gov/gene/?term=1392)(22.373)[GLI3](http://www.ncbi.nlm.nih.gov/gene/?term=2737)(22.373)[PLXNB3](http://www.ncbi.nlm.nih.gov/gene/?term=5365)(22.373)[CAV3](http://www.ncbi.nlm.nih.gov/gene/?term=859)(22.373)[NEFL](http://www.ncbi.nlm.nih.gov/gene/?term=4747)(22.373) [UCN2](http://www.ncbi.nlm.nih.gov/gene/?term=90226)(22.373)[IL6](http://www.ncbi.nlm.nih.gov/gene/?term=3569)(22.373)[RNF207](http://www.ncbi.nlm.nih.gov/gene/?term=388591)(22.373)[TACR2](http://www.ncbi.nlm.nih.gov/gene/?term=6865)(22.373)[PTGS2](http://www.ncbi.nlm.nih.gov/gene/?term=5743)(22.373) [NRP1](http://www.ncbi.nlm.nih.gov/gene/?term=8829)(22.373)[NPPA](http://www.ncbi.nlm.nih.gov/gene/?term=4878)(22.373)[MC5R](http://www.ncbi.nlm.nih.gov/gene/?term=4161)(22.373)[AQP1](http://www.ncbi.nlm.nih.gov/gene/?term=358)(22.373)[MC3R](http://www.ncbi.nlm.nih.gov/gene/?term=4159)(22.373) [TAC4](http://www.ncbi.nlm.nih.gov/gene/?term=255061)(22.373)[TNR](http://www.ncbi.nlm.nih.gov/gene/?term=7143)(22.373)[NOS3](http://www.ncbi.nlm.nih.gov/gene/?term=4846)(22.373)[EPHB1](http://www.ncbi.nlm.nih.gov/gene/?term=2047)(22.373)[SNCA](http://www.ncbi.nlm.nih.gov/gene/?term=6622)(22.373) [DRD1](http://www.ncbi.nlm.nih.gov/gene/?term=1812)(22.373)[PDE2A](http://www.ncbi.nlm.nih.gov/gene/?term=5138)(22.373)[CD34](http://www.ncbi.nlm.nih.gov/gene/?term=947)(22.373)[KCNMA1](http://www.ncbi.nlm.nih.gov/gene/?term=3778)(22.373)[POU4F3](http://www.ncbi.nlm.nih.gov/gene/?term=5459)(22.373) [CBFA2T3](http://www.ncbi.nlm.nih.gov/gene/?term=863)(22.373)[CALCA](http://www.ncbi.nlm.nih.gov/gene/?term=796)(22.373)[PINK1](http://www.ncbi.nlm.nih.gov/gene/?term=65018)(22.373)[NOS1](http://www.ncbi.nlm.nih.gov/gene/?term=4842)(22.373)[TREM1](http://www.ncbi.nlm.nih.gov/gene/?term=54210)(22.373) [GJA5](http://www.ncbi.nlm.nih.gov/gene/?term=2702)(22.373)[PDGFB](http://www.ncbi.nlm.nih.gov/gene/?term=5155)(22.373)[CARTPT](http://www.ncbi.nlm.nih.gov/gene/?term=9607)(22.373)[NEFH](http://www.ncbi.nlm.nih.gov/gene/?term=4744)(22.373)[ATP8B1](http://www.ncbi.nlm.nih.gov/gene/?term=5205)(22.373) [KISS1](http://www.ncbi.nlm.nih.gov/gene/?term=3814)(22.373)[GRM7](http://www.ncbi.nlm.nih.gov/gene/?term=2917)(22.373)[FOS](http://www.ncbi.nlm.nih.gov/gene/?term=2353)(22.373)[CRHR2](http://www.ncbi.nlm.nih.gov/gene/?term=1395)(22.373)[GRIN1](http://www.ncbi.nlm.nih.gov/gene/?term=2902)(22.373) [HCN2](http://www.ncbi.nlm.nih.gov/gene/?term=610)(22.373) |
| [Alpha-Ylangene](http://www.megabionet.org/tcmid/ingredient/23748/) | [KCND1](http://www.ncbi.nlm.nih.gov/gene/?term=3750)(80.882)[KCNA3](http://www.ncbi.nlm.nih.gov/gene/?term=3738)(80.882)[PRKAB1](http://www.ncbi.nlm.nih.gov/gene/?term=5564)(80.882)[ADH1A](http://www.ncbi.nlm.nih.gov/gene/?term=124)(80.882)[KCNA10](http://www.ncbi.nlm.nih.gov/gene/?term=3744)(80.882) [GAMT](http://www.ncbi.nlm.nih.gov/gene/?term=2593)(80.882)[KCNC3](http://www.ncbi.nlm.nih.gov/gene/?term=3748)(80.882)[KCNA1](http://www.ncbi.nlm.nih.gov/gene/?term=3736)(80.882)[KCNA2](http://www.ncbi.nlm.nih.gov/gene/?term=3737)(80.882)[TPO](http://www.ncbi.nlm.nih.gov/gene/?term=7173)(80.882) [CAT](http://www.ncbi.nlm.nih.gov/gene/?term=847)(80.882)[KCNB1](http://www.ncbi.nlm.nih.gov/gene/?term=3745)(80.882)[ADH1B](http://www.ncbi.nlm.nih.gov/gene/?term=125)(80.882)[DLG4](http://www.ncbi.nlm.nih.gov/gene/?term=1742)(80.882)[KCNC2](http://www.ncbi.nlm.nih.gov/gene/?term=3747)(80.882) [KCNC1](http://www.ncbi.nlm.nih.gov/gene/?term=3746)(80.882)[ADH1C](http://www.ncbi.nlm.nih.gov/gene/?term=126)(80.882)[KCNB2](http://www.ncbi.nlm.nih.gov/gene/?term=9312)(80.882)[KCNA5](http://www.ncbi.nlm.nih.gov/gene/?term=3741)(80.882)[RNASE1](http://www.ncbi.nlm.nih.gov/gene/?term=6035)(80.882) [KCND2](http://www.ncbi.nlm.nih.gov/gene/?term=3751)(80.882)[ALDH2](http://www.ncbi.nlm.nih.gov/gene/?term=217)(80.882)[KCNA4](http://www.ncbi.nlm.nih.gov/gene/?term=3739)(80.882)[KCNA7](http://www.ncbi.nlm.nih.gov/gene/?term=3743)(80.882)[KCNA6](http://www.ncbi.nlm.nih.gov/gene/?term=3742)(80.882) [KCND3](http://www.ncbi.nlm.nih.gov/gene/?term=3752)(80.882)[GUCY1B3](http://www.ncbi.nlm.nih.gov/gene/?term=2983)(55.444)[GATM](http://www.ncbi.nlm.nih.gov/gene/?term=2628)(55.444)[KCNK4](http://www.ncbi.nlm.nih.gov/gene/?term=50801)(55.444)[IYD](http://www.ncbi.nlm.nih.gov/gene/?term=389434)(55.444) [KCNQ1](http://www.ncbi.nlm.nih.gov/gene/?term=3784)(55.444)[RDH11](http://www.ncbi.nlm.nih.gov/gene/?term=51109)(48.000)[RBP3](http://www.ncbi.nlm.nih.gov/gene/?term=5949)(48.000)[RETSAT](http://www.ncbi.nlm.nih.gov/gene/?term=54884)(48.000)[CYP17A1](http://www.ncbi.nlm.nih.gov/gene/?term=1586)(48.000) [RDH13](http://www.ncbi.nlm.nih.gov/gene/?term=112724)(48.000)[DHRS3](http://www.ncbi.nlm.nih.gov/gene/?term=9249)(48.000)[RDH5](http://www.ncbi.nlm.nih.gov/gene/?term=5959)(48.000)[ESR1](http://www.ncbi.nlm.nih.gov/gene/?term=2099)(48.000)[PGR](http://www.ncbi.nlm.nih.gov/gene/?term=5241)(48.000) [ALDH1A3](http://www.ncbi.nlm.nih.gov/gene/?term=220)(48.000)[RDH12](http://www.ncbi.nlm.nih.gov/gene/?term=145226)(48.000)[OPRK1](http://www.ncbi.nlm.nih.gov/gene/?term=4986)(48.000)[DHRS4](http://www.ncbi.nlm.nih.gov/gene/?term=10901)(48.000)[RBP1](http://www.ncbi.nlm.nih.gov/gene/?term=5947)(48.000) [ALDH1A1](http://www.ncbi.nlm.nih.gov/gene/?term=216)(48.000)[RLBP1](http://www.ncbi.nlm.nih.gov/gene/?term=6017)(48.000)[RDH14](http://www.ncbi.nlm.nih.gov/gene/?term=57665)(48.000)[RDH8](http://www.ncbi.nlm.nih.gov/gene/?term=50700)(48.000)[LRAT](http://www.ncbi.nlm.nih.gov/gene/?term=9227)(48.000) [ALDH1A2](http://www.ncbi.nlm.nih.gov/gene/?term=8854)(48.000)[NR3C2](http://www.ncbi.nlm.nih.gov/gene/?term=4306)(48.000)[FBP1](http://www.ncbi.nlm.nih.gov/gene/?term=2203)(22.373)[DGKI](http://www.ncbi.nlm.nih.gov/gene/?term=9162)(22.373)[ADH7](http://www.ncbi.nlm.nih.gov/gene/?term=131)(22.373) [NFIB](http://www.ncbi.nlm.nih.gov/gene/?term=4781)(22.373)[CYGB](http://www.ncbi.nlm.nih.gov/gene/?term=114757)(22.373)[NRXN3](http://www.ncbi.nlm.nih.gov/gene/?term=9369)(22.373)[LRRC4B](http://www.ncbi.nlm.nih.gov/gene/?term=94030)(22.373)[AQP8](http://www.ncbi.nlm.nih.gov/gene/?term=343)(22.373) [ARX](http://www.ncbi.nlm.nih.gov/gene/?term=170302)(22.373)[IGF1](http://www.ncbi.nlm.nih.gov/gene/?term=3479)(22.373)[SHANK3](http://www.ncbi.nlm.nih.gov/gene/?term=85358)(22.373)[ACY3](http://www.ncbi.nlm.nih.gov/gene/?term=91703)(22.373)[ASPA](http://www.ncbi.nlm.nih.gov/gene/?term=443)(22.373) [NRXN1](http://www.ncbi.nlm.nih.gov/gene/?term=9378)(22.373)[EDN1](http://www.ncbi.nlm.nih.gov/gene/?term=1906)(22.373)[OXT](http://www.ncbi.nlm.nih.gov/gene/?term=5020)(22.373)[MAGI2](http://www.ncbi.nlm.nih.gov/gene/?term=9863)(22.373)[APOE](http://www.ncbi.nlm.nih.gov/gene/?term=348)(22.373) [SOX15](http://www.ncbi.nlm.nih.gov/gene/?term=6665)(22.373)[CNTNAP4](http://www.ncbi.nlm.nih.gov/gene/?term=85445)(22.373)[RNASE4](http://www.ncbi.nlm.nih.gov/gene/?term=6038)(22.373)[RAB3A](http://www.ncbi.nlm.nih.gov/gene/?term=5864)(22.373)[MC4R](http://www.ncbi.nlm.nih.gov/gene/?term=4160)(22.373) [IL1B](http://www.ncbi.nlm.nih.gov/gene/?term=3553)(22.373)[GRIN2A](http://www.ncbi.nlm.nih.gov/gene/?term=2903)(22.373)[SLC17A7](http://www.ncbi.nlm.nih.gov/gene/?term=57030)(22.373)[CYP11A1](http://www.ncbi.nlm.nih.gov/gene/?term=1583)(22.373)[SCN5A](http://www.ncbi.nlm.nih.gov/gene/?term=6331)(22.373) [SCN10A](http://www.ncbi.nlm.nih.gov/gene/?term=6336)(22.373)[SPARC](http://www.ncbi.nlm.nih.gov/gene/?term=6678)(22.373)[SORCS3](http://www.ncbi.nlm.nih.gov/gene/?term=22986)(22.373)[RNASE8](http://www.ncbi.nlm.nih.gov/gene/?term=122665)(22.373)[KCNIP2](http://www.ncbi.nlm.nih.gov/gene/?term=30819)(22.373) [GPX7](http://www.ncbi.nlm.nih.gov/gene/?term=2882)(22.373)[CRLF1](http://www.ncbi.nlm.nih.gov/gene/?term=9244)(22.373)[FADD](http://www.ncbi.nlm.nih.gov/gene/?term=8772)(22.373)[SNTG2](http://www.ncbi.nlm.nih.gov/gene/?term=54221)(22.373)[SCGB1A1](http://www.ncbi.nlm.nih.gov/gene/?term=7356)(22.373) [OXTR](http://www.ncbi.nlm.nih.gov/gene/?term=5021)(22.373)[ALDH1B1](http://www.ncbi.nlm.nih.gov/gene/?term=219)(22.373)[LEP](http://www.ncbi.nlm.nih.gov/gene/?term=3952)(22.373)[NLGN1](http://www.ncbi.nlm.nih.gov/gene/?term=22871)(22.373)[CACNA1D](http://www.ncbi.nlm.nih.gov/gene/?term=776)(22.373) [RAPGEF2](http://www.ncbi.nlm.nih.gov/gene/?term=9693)(22.373)[DARS](http://www.ncbi.nlm.nih.gov/gene/?term=1615)(22.373)[ANK3](http://www.ncbi.nlm.nih.gov/gene/?term=288)(22.373)[KCNE5](http://www.ncbi.nlm.nih.gov/gene/?term=23630)(22.373)[PPARD](http://www.ncbi.nlm.nih.gov/gene/?term=5467)(22.373) [NKX2-1](http://www.ncbi.nlm.nih.gov/gene/?term=7080)(22.373)[MIP](http://www.ncbi.nlm.nih.gov/gene/?term=4284)(22.373)[RYR3](http://www.ncbi.nlm.nih.gov/gene/?term=6263)(22.373)[ZPR1](http://www.ncbi.nlm.nih.gov/gene/?term=8882)(22.373)[ALDH3B1](http://www.ncbi.nlm.nih.gov/gene/?term=221)(22.373) [NPPA](http://www.ncbi.nlm.nih.gov/gene/?term=4878)(22.373)[STX1A](http://www.ncbi.nlm.nih.gov/gene/?term=6804)(22.373)[PAXBP1](http://www.ncbi.nlm.nih.gov/gene/?term=94104)(22.373)[PAX7](http://www.ncbi.nlm.nih.gov/gene/?term=5081)(22.373)[ALDH3B2](http://www.ncbi.nlm.nih.gov/gene/?term=222)(22.373) [RNASE2](http://www.ncbi.nlm.nih.gov/gene/?term=6036)(22.373)[NRXN2](http://www.ncbi.nlm.nih.gov/gene/?term=9379)(22.373)[ADORA1](http://www.ncbi.nlm.nih.gov/gene/?term=134)(22.373)[ADH4](http://www.ncbi.nlm.nih.gov/gene/?term=127)(22.373)[GJA5](http://www.ncbi.nlm.nih.gov/gene/?term=2702)(22.373) [FAS](http://www.ncbi.nlm.nih.gov/gene/?term=355)(22.373)[DAB2IP](http://www.ncbi.nlm.nih.gov/gene/?term=153090)(22.373)[CYP2E1](http://www.ncbi.nlm.nih.gov/gene/?term=1571)(22.373)[ACY1](http://www.ncbi.nlm.nih.gov/gene/?term=95)(22.373) |
